# Newly synthesised oxime and lactone derivatives from *Dipterocarpus alatus* dipterocarpol as anti-diabetic inhibitors: experimental bioassay-based evidence and theoretical computation-based prediction†

**DOI:** 10.1039/d1ra04461c

**Published:** 2021-11-04

**Authors:** Tran Thi Phuong Thao, Thanh Q. Bui, Nguyen Thi Thanh Hai, Lam K. Huynh, Phan Tu Quy, Nguyen Chi Bao, Nguyen Thi Dung, Nguyen Linh Chi, Tran Van Loc, Irina E. Smirnova, Anastasiya V. Petrova, Pham Thi Ninh, Tran Van Sung, Nguyen Thi Ai Nhung

**Affiliations:** Institute of Chemistry, Vietnam Academy of Science and Technology (VAST) 18 Hoang Quoc Viet Road, Cau Giay Ha Noi Vietnam tranvansungvhh@gmail.com; Graduate University of Science and Technology, VAST 18 Hoang Quoc Viet Road, Cau Giay Ha Noi Vietnam; Department of Chemistry, University of Sciences, Hue University Hue City Vietnam ntanhung@hueuni.edu.vn; International University Quarter 6, Linh Trung Ward, Thu Duc District Ho Chi Minh City Vietnam; Department of Natural Sciences & Technology, Tay Nguyen University Buon Ma Thuot Vietnam; Hue University Hue City Vietnam; Ufa Institute of Chemistry-Subdivision of the Ufa Federal Research Centre of the Russian Academy of Sciences Prospekt Oktyabrya 71 Ufa Russian Federation; Vietnam National University Ho Chi Minh City Vietnam

## Abstract

*Dipterocarpus alatus*-derived products are expected to exhibit anti-diabetes properties. Natural dipterocarpol (1) was isolated from *Dipterocarpus alatus* collected in Quang Nam province, Vietnam; afterwards, 20 derivatives including 13 oxime esters (2 and 3a–3m) and 7 lactones (4, 5, 6a–6e) were semi-synthesised. Their inhibitory effects towards diabetes-related proteins were investigated experimentally (α-glucosidase) and computationally (3W37, 3AJ7, and PTP1B). Except for compound 2, the other 19 compounds (3a–3m, 4, 5, and 6a–6d) are reported for the first time, which were modified at positions C-3, C-24 and C-25 of the dipterocarpol *via* imidation, esterification, oxidative cleavage and lactonisation reactions. A framework based on docking-QSARIS combination was proposed to predict the inhibitory behaviour of the ligand-protein complexes. Enzyme assays revealed the most effective α-glucosidase inhibitors, which follow the order 5 (IC_50_ of 2.73 ± 0.05 μM) > 6c (IC_50_ of 4.62 ± 0.12 μM) > 6e (IC_50_ of 7.31 ± 0.11 μM), and the computation-based analysis confirmed this, *i.e.*, 5 (mass: 416.2 amu; polarisability: 52.4 Å^3^; DS: −14.9 kcal mol^−1^) > 6c (mass: 490.1 amu; polarisability: 48.8 Å^3^; DS: −13.7 kcal mol^−1^) > 6e (mass: 549.2 amu; polarisability: 51.6 Å^3^; DS: −15.2 kcal mol^−1^). Further theoretical justifications predicted 5 and 6c as versatile anti-diabetic inhibitors. The experimental results encourage next stages for the development of anti-diabetic drugs and the computational strategy invites more relevant work for validation.

## Abstract 2

*Dipterocarpus alatus*-derived products are expected to exhibit anti-diabetes properties.

## Graphical abstract

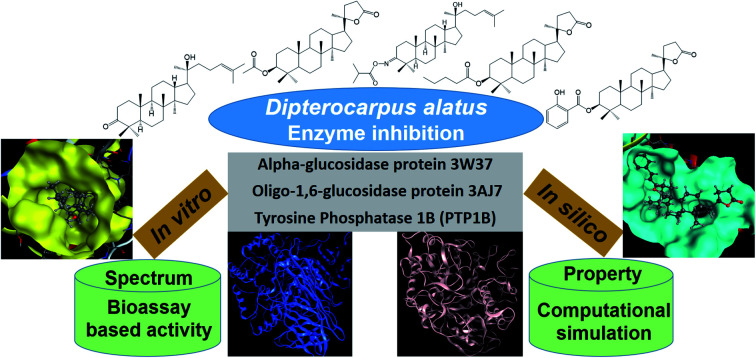

## 1. Introduction

Diabetes mellitus (DM) is increasing in humans worldwide, especially in middle-income countries. According to The World Health Organization (WHO), diabetes was directly responsible for about 1.5 million deaths in 2019 and it is predicted that the increasing death toll will cause this disorder to be the seventh leading cause of death by 2030.^1^ It has also been evidenced that cardiovascular disease, blindness, kidney failure, stroke, and limb amputation are related to this disorder.^2^ In particular, the noninsulin-dependent disorder, *viz.*, type 2 DM, accounts for 90–95% of the total DM patients.^3^ The mechanism of type 2 DM is well known, stemming from the ineffective use of insulin in the body or insufficient insulin production in the pancreas of patients.

**Fig. 1:**
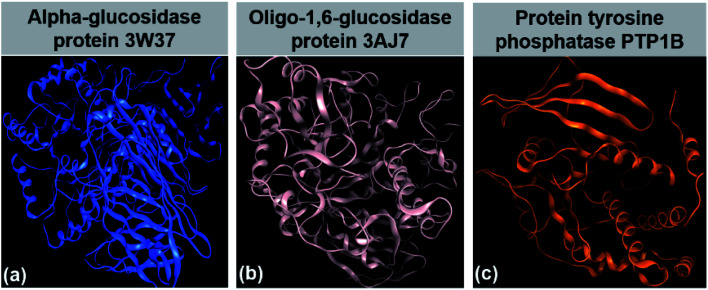
(a) α-Glucosidase protein 3W37. (b) Oligo-1,6-glucosidase protein 3W37. (c) Protein tyrosine phosphatase 1B PTP1B.

Inhibition of glucose- and insulin-based enzymes is the main strategy for the therapeutic treatment of type 2 diabetes. The former attempts to reduce gut glucose absorption by controlling postprandial hyperglycemia by mitigating the activity of glucosidases; meanwhile, the latter involves insulin signaling regulation by compensating defects in insulin secretion and insulin action.^4^ Firstly, α-glucosidase, an exoenzyme found in animals, plants, bacteria, and fungi, breaks down starch and disaccharides to glucose. A study suggested that this enzyme can only yield monosaccharides by catalysing the hydrolysis of α-(1→4) and α-(1→6) bonds,^5^ confirming the sources of α-glucosidase from sugar beet seeds.^6^ Data on the α-glucosidase crystal structure can be found at the Worldwide Protein Data Bank under entry PDB-3W37 (DOI: 10.2210/pdb3W37/pdb). Secondly, oligo-1,6-glucosidase, also known as isomaltase, is a debranching endoenzyme, hydrolysing only the α-1,6 linkage in starch and glycogen to produce sugars with an α-configuration.^7^ Besides some bacterial species such as *Bacillus cereus*, oligo-1,6-glucosidase is present mainly in the animal kingdom.^8^ In humans, it can be found on the small intestine brush border.^9^ Protein structural data of isomaltase can be download directly from the Worldwide Protein Data Bank database under entry PDB-3AJ7 (DOI: 10.2210/pdb3AJ7/pdb). Thirdly, protein tyrosine phosphatase 1B (PTP1B) is a major glucose-homeostasis and energy-metabolism regulator, and thus regarded as an attractive drug target for therapeutic intervention in type 2 diabetes and obesity.^10^ It blocks insulin receptor substrate-1 and dephosphorylate phosphotyrosine residues, thus causing insulin insensitivity or even cut-off of intracellular insulin signaling.^11^ Conversely, regarding the leptin signaling pathway, this protein binds and dephosphorylates leptin receptor Janus kinase 2 (JAK2), thereby resulting in the malfunctioning of energy balance.^11^ The PTP1B structure has already been well determined and its information is available for public reference at UniProtKB, archived under entry ID: UniProtKB-A0A0U1XP67. Therefore, 3W37, 3AJ7, and PTP1B (Fig. 1) are considered highly promising drug targets for the effective treatment of type 2 diabetes. In principle, multiple inhibition of the protein tyrosine phosphatase 1B (PTP1B) and glycoside hydrolase proteins (3W37 and 3AJ7) is a promising strategy to suppress hyperglycemia and improve insulin sensitisation simultaneously.

**Fig. 2:**
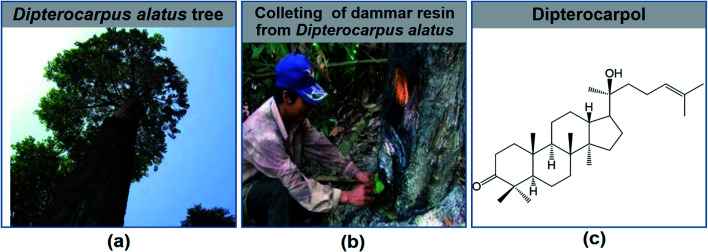
(a) *Dipterocarpus alatus* tree. (b) Collection of dammar resin from *Dipterocarpus alatus*. (c) Structure of dipterocarpol.

Natural products are considered highly valuable for drug discovery due to their diverse structures and biological activities. In particular, triterpenes belong to a large group of naturally occurring compounds derived from the precursor squalene.^12^ Dammarane is a tetracyclic triterpene, existing as the main components in dammar resin^13,14^ and ginseng.^15^ The basic skeleton of dammarane is comprised of a tetracyclic moiety and a side chain at C-17.^16,17^ Recently, the number of publications and patents on the anti-diabetic activity of dammarane triterpenoids has increased rapidly.^18–21^ Besides, clinical trials and animal model experiments have proven the ability of dammarane triterpenoids to prevent and treat diabetes by lowering the blood glucose, regulating lipid metabolism, and increasing insulin sensitivity.^22^ Thus, considering the advantage of the diversity and novelty of the dammarane structure, a series of chemical modifications of leading compounds has been achieved. Consequently, many synthetic dammarane derivatives have been reported to improve hyperglycemia, glucose tolerance and α-glucosidase inhibition.^22–25^ Dipterocarpol, a dammarane-type triterpenoid, is the main metabolite of the resin from *Dipterocarpus alatus* (Dipterocarpaceae), a tropical plant growing in countries in South East Asia such as Cambodia, Laos, Thailand, the Philippines and Vietnam^26^ (Fig. 2). It was reported that dipterocarpol and its derivatives exhibit antituberculosis,^27^ antimicrobial,^28^ hepatoprotective,^29^ and cytotoxic activity.^30^

**Fig. 3:**
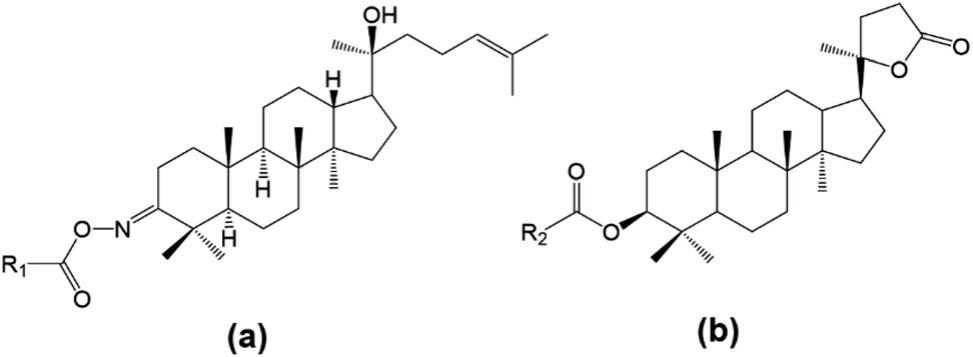
Designed structures of dipterocarpol derivatives.

In this work, two different approaches to modify the structure of dipterocarpol are proposed based on their advantages reported in the literature. The modification of the ring at C-3 of dipterocarpol to obtain oxime and its acyl derivatives (Fig. 3a) was designed. The activating group ketone at C-3 can be easily converted to oxime, followed by *O*-acylation to obtain acyloxyimino compounds (–CR=N–O–COOR). This type of compound (containing acyloxyimino functional groups) is known to exhibit important biological activities, *e.g.*, antiviral, anticancer and anti-diabetes.^31^ According to our knowledge, several studies on the synthesis of oxime esters derived from lupane or oleane triterpenoids have been carried out;^31^ nevertheless, similar transformation regarding dammarane triterpenoids has not reported to date. Another modification was at the side chain, focusing on the formation of a lactone ring, instead of a long-chain linear of dipterocarpol (Fig. 3b). Previous studies showed that the location of a lactone ring at C-17 of some dammarane derivatives helps promote the antihyperglycemic effects significantly.^20,25,32^ Besides, a series of ester derivatives at C-3 was also introduced.

*In silico* research has been developed extensively in an attempt to accurately predict molecular behaviour at the biological scale. In particular, molecular dynamics gives a view of the dynamic “evolution” of atoms and molecules in a system, whose trajectories are determined by numerically solving Newton's equations of motion for a system of interacting particles. This technique is widely known as one of the most powerful computer-aided tools for pharmacophore development and drug design. However, its downside of long computation times, high cost and significant power consumption highly limit its applicability. Conversely, although successfully complementing the dynamics-related disadvantage by introducing a view of static inhibitory stability, the molecular docking technique is based on a precursor condition that the ligands are already placed within a certain radius to the targeted protein, and thus there is a lack of information on their preceding transport in or interaction with plasmatic components in biological media. Accordingly, a strategy based on the combination of the static docking technique and molecular dynamics has been proposed in an attempt to retrieve meaningful models for ligand–protein inhibition.^33–35^ Nevertheless, the cost of its implementation is still considered high in low-based research institutes. QSARIS,^36^ which was released by SciVision Biotech Inc., is a leading-edge database system for predicting the physiochemical properties of lead compounds in drug and agrochemical research. This software promises sufficient compensation for the information lost in the pre-docking presumption. Therefore, a QSARIS-docking combination can be a potential approach for the prediction of ligand–protein inhibition, in general.

Herein, both experimental and theoretical studies were conducted. The isolation of dipterocarpol and semi-synthesis of its twenty derivatives were carried out on *Dipterocarpus alatus* from Vietnam. Their α-glucosidase inhibition was investigated by enzyme assays. The experimental evidence collected was used to retrieve a comprehensive theoretical model framed by molecular docking simulation coupled with QSARIS-based analysis. The experiment-theory retrieval was applied to all the diabetes-related protein structures.

## 2. Methodology

### 2.1. Experiment

#### 2.1.1. Materials and characterisation

Reagents and solvents were purchased from commercial suppliers and used without further purification. Column chromatography was performed using silica gel from Merck (Kieselgel 60, 230–400 mesh). Analytical thin-layer chromatography (TLC) was carried out on pre-coated silica gel 60 F_254_ sheets (0.25 mm, Merck). Nuclear magnetic resonance (NMR) spectroscopic data was acquired on a Bruker Advance III spectrometer (Switzerland) with the chemical shift in ppm using tetramethylsilane (TMS) as the internal standard. Electrospray ionization mass spectrometry (ESI-MS) was performed on an Agilent 1100 mass spectrometer from the USA. Fourier-transform infrared (FT-IR) spectroscopy was performed on a PerkinElmer Spectrum Two (UK). High-resolution electrospray ionization mass spectra (HR-ESI-MS) were recorded on a SCEIX X500R QTOF (USA). TLC spots were visually observed under UV light (254 and 365 nm) and by dipping in vanillin/H_2_SO_4_ reagent followed by heating.

#### 2.1.2. Isolation and synthesis

##### Isolation of dipterocarpol (compound 1) from *Dipterocarpus alatus*

The resin of *Dipterocarpus alatus* (4.0 kg) was collected in Dai Thach commune, Dai Loc district, Quang Nam province, Vietnam in April, 2018. The plant sample was identified taxonomically by Mst. Nguyen The Anh, Institute of Chemistry, Vietnam Academy of Science and Technology, Hanoi, Vietnam. Water (2 L) was added to the resin, and successively extracted with *n*-hexane (4 × 2 L) and CH_2_Cl_2_ (3 × 2 L) at room temperature. The extracts were combined and evaporated under reduced pressure to obtain the *n*-hexane and dichloromethane extracts, respectively. The dichloromethane extract was dipped in MeOH (2 L, for 5 h). Finally, the solvent of the MeOH extract was removed under reduced pressure to obtain a white solid, which was recrystalised in EtOH to yield dipterocarpol (80 g).

**Scheme 1:**
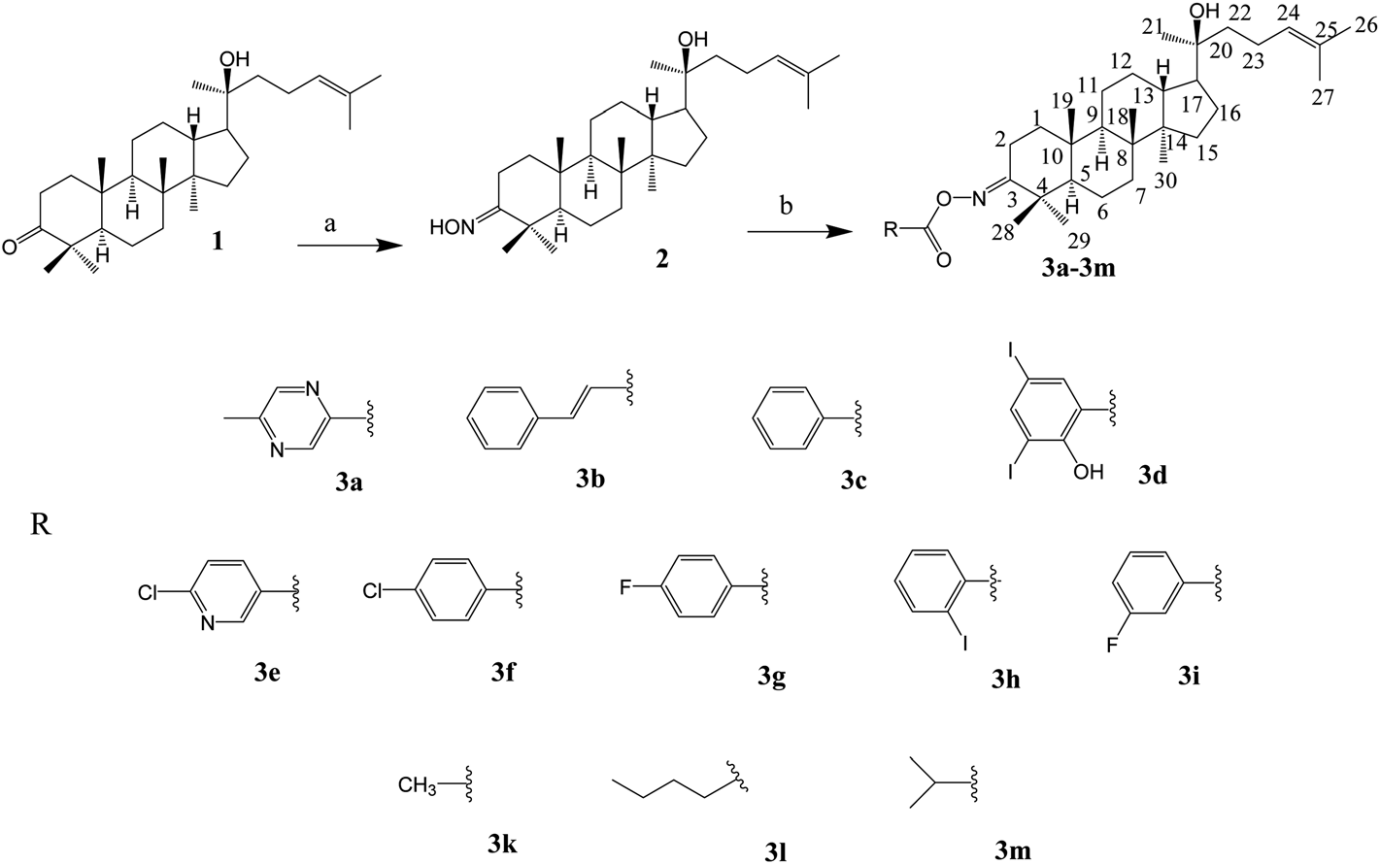
Synthesis of oxime ester derivatives at C-3 of dipterocarpol. (a) NH_2_OH·HCl, TEA, C_2_H_5_OH, 24 h, rt (83%). (b) Synthesis of 3a–3i: DCC, DMAP, RCOOH, CH_2_Cl_2_, 5 h, rt (48–80%); 3k: pyridine, Ac_2_O, 24 h, rt (81%); 3l, 3m: RCOCl, CH_2_Cl_2_, 19 h, rt (80–83%).

The synthesis of the oxime esters is summarised in Scheme 1.

##### Synthesis of oxime 2

A solution of compound 1 (500 mg, 1.13 mmol), hydroxylamine hydrochloride (142 mg, 2.03 mmol) and triethylamine (0.1 mL) in ethanol (3 mL) was stirred at room temperature for 24 h. The solvent was removed under vacuum and water (20 mL) was added to the residue, followed by extraction with CH_2_Cl_2_ (3 × 20 mL). The CH_2_Cl_2_ extracts were combined, dried over anhydrous Na_2_SO_4_, and the solvent evaporated to obtain a white solid. The solid was purified by silica gel column chromatography (*n*-hexane/EtOAc 9 : 1) to obtain compound 2 (yield: 430 mg, 83.0%).

##### General procedures for the synthesis of oxime esters 3a–3i

To a solution of compound 2 (40 mg, 0.087 mmol, 1 eq.), DCC (36 mg, 0.174 mmol, 2 eq.) and DMAP (7.42 mg, 0.06 mmol) in CH_2_Cl_2_ (5 mL) were added with carboxylic acid (0.174 mmol, 2 eq.). The reaction mixture was stirred at room temperature for 5 h, and then filtered to separate the solution from the solid. The solution was evaporated under vacuum, followed by the addition of water to yield a white solid, which was purified by silica gel column chromatography (*n*-hexane/EtOAc) to attain compounds 3a–3i (48–80% yield).

##### Synthesis of oxime ester 3k

To a solution of compound 2 (40 mg, 0.087 mmol) in pyridine (1 mL), acetic anhydride (0.3 mL) was added. The reaction mixture was stirred at room temperature for 24 h. The solvent was removed under reduced pressure to obtain a white residue. The residue was purified on a silica gel column (*n*-hexane/EtOAc 87 : 13) to yield 35 mg 3k (80.6%).

##### General procedures for the synthesis of oxime esters 3l and 3m

To a solution of compound 2 (40 mg, 0.087 mmol) in CH_2_Cl_2_ (2.5 mL), valeryl chloride (0.015 mL, 1.25 eq. for compound 3l) or isobutyryl chloride (0.013 mL, 1.25 eq. for compound 3m) was added. The reaction mixture was stirred at room temperature for 19 h, and then the solvent was evaporated under reduced pressure. The white crude product was purified by silica gel column chromatography (*n*-hexane/EtOAc 9 : 1) to afford compound 3l (39 mg, 83%) or compound 3m (38 mg, 82%).

**Scheme 2:**
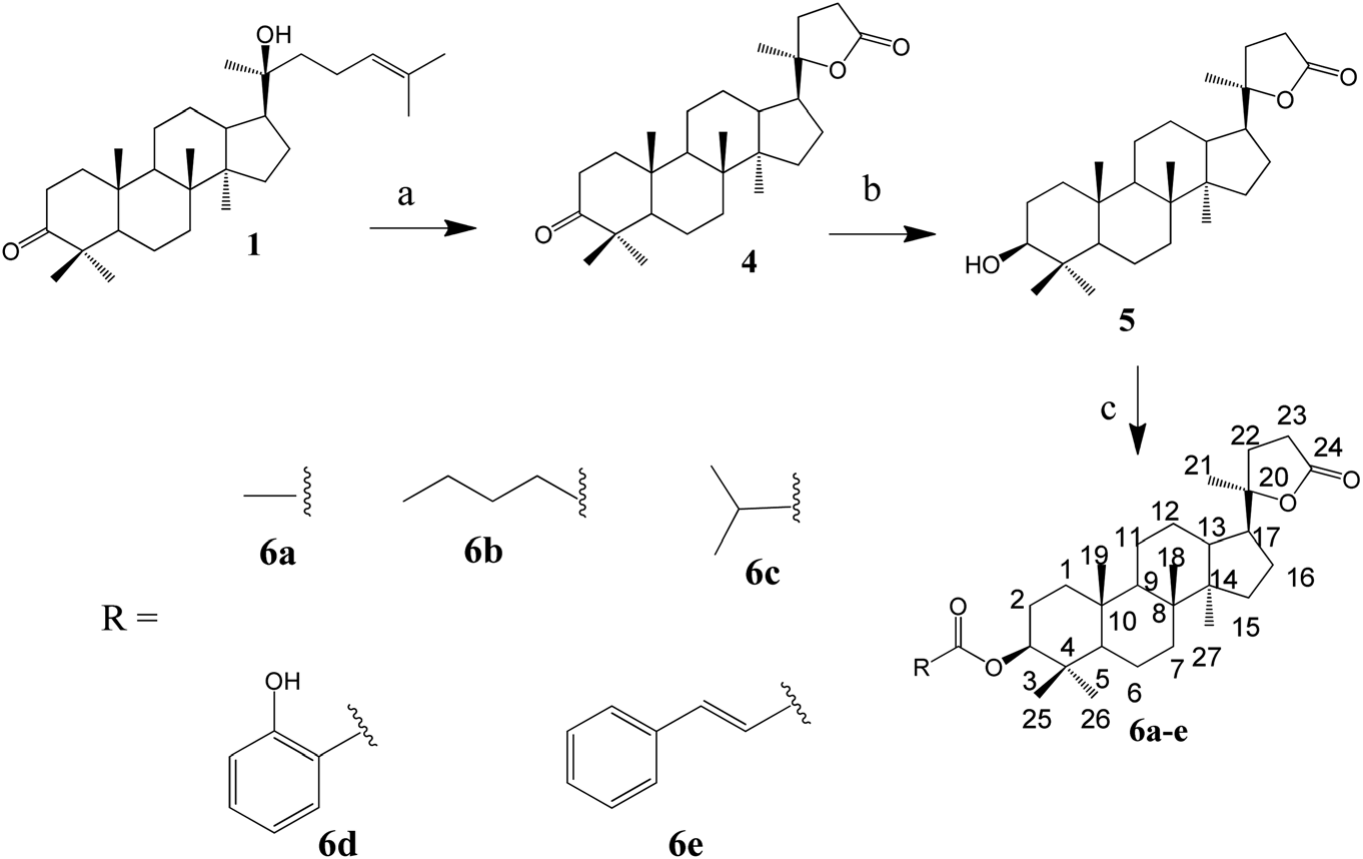
Synthesis of lactone derivatives of dipterocarpol. (a) CrO_3_, CH_3_COOH, H_2_O, 40 min, rt (85.5%). (b) NaBH_4_, MeOH, 20 min, rt (74%). (c) Synthesis of 6a: Ac_2_O, pyridine, 24 h, rt (73%); 6b and 6c: RCOCl, CH_2_Cl_2_, 20 h, rt (60–75%); 6d and 6e: RCOOH, DCC, DMAP, CH_2_Cl_2_, rt, 48 h for 6d (66%) or reflux, 28 h for 6e (61%).

The synthesis of the lactone derivatives is summarised in Scheme 2.

##### Synthesis of compound 4

A mixture of CrO_3_ (2.45 g, 16.2 mmol, 2.8 eq.) in CH_3_COOH (5 mL) and H_2_O (3 mL) was added dropwise to a solution of compound 1 (2.5 g, 5.65 mmol, 1 eq.) in CH_3_COOH (20 mL) at 5 °C. The reaction mixture was allowed to warm up to room temperature and further stirred for 30 min. A solution of 10% NaCl (50 mL) was added to the reaction mixture, followed by extraction with EtOAc (3 × 50 mL). The extract was evaporated under vacuum to yield a white solid, which was purified by silica gel column chromatography (*n*-hexane/acetone 9 : 1) to yield compound 4 (2.0 g, 4 mmol, 85.5%).

##### Synthesis of compound 5

To a solution of compound 4 (700 mg, 1.7 mmol, 1 eq.) in MeOH (7 mL) at 5 °C, NaBH_4_ (260 mg, 6.84 mmol, 4 eq.) was added slowly. The reaction mixture was allowed to warm up to room temperature and further stirred for 20 min. Acetone (5 mL) was added, and the reaction mixture was continuously stirred for an additional 5 min. Cold water (5 mL) was added to the reaction, followed by evaporation. Water (30 mL) was added to the residue, and then extracted with EtOAc (3 × 30 mL). The solvent was removed to give a white powder, which was purified by silica gel column chromatography (*n*-hexane/EtOAc 9 : 1) to yield compound 5 (520 mg, 74%).

##### Synthesis of compound 6a

To a solution of compound 5 (20 mg, 0.048 mmol) in pyridine (1 mL), acetic anhydride (0.3 mL) was added. The reaction mixture was stirred at room temperature for 24 h. The solvent was removed under reduced pressure to obtain a white residue. The residue was purified by silica gel column chromatography (*n*-hexane/EtOAc 9 : 1) to give compound 6a (16 mg, 73%).

##### General procedure for the synthesis of compounds 6b and 6c

To a solution of compound 5 (20 mg, 0.048 mmol, 1 eq.) in CH_2_Cl_2_ (2.5 mL), valeryl chloride (0.015 mL, 2.5 eq., for compound 6b) or isobutyryl chloride (0.013 mL, 2.5 eq., for compound 6c) was added. The reaction mixture was stirred at room temperature for 20 h, and then the solvent evaporated under reduced pressure. The obtained white crude product was purified by Sephadex LH-20 column chromatography (*n*-hexane/CH_2_Cl_2_/MeOH 1/1/2) to obtain the respective compound 6b (18 mg, 75%) or compound 6c (14 mg, 60%).

##### General procedures for the synthesis of compounds 6d and 6e

To a solution of compound 5 (20 mg, 0.048 mmol, 1 eq.), DCC (20 mg, 0.096 mmol, 2.0 eq.) and DMAP (6 mg, 0.048 mmol, 1.0 eq.) in 5 mL CH_2_Cl_2_ were added with carboxylic acid (2 eq., 0.096 mmol). The reaction mixture was stirred for 48 h at room temperature (for compound 6d) or refluxed for 28 h (for compound 6e). The white precipitate was filtered. The remaining solution was evaporated under vacuum, and water was added and extracted with EtOAc (3 × 15 mL). The solvent was removed from the extract, and the remaining residue was subjected to a silica gel column (*n*-hexane/EtOAc) to obtain compound 6d (17 mg, 66%) or 6e (16 mg, 61%).

#### 2.1.3. α-Glucosidase inhibitory assay

The bioassay was carried out on 96 well-plates (200 μL per well). A solution of the test samples in 10% dimethyl sulfoxide (DMSO) was diluted further to concentrations of 1024, 256, 64, 16, 4, and 1 μg mL^−1^. Each concentration of the test samples (10 μL) was incubated at 37 °C with phosphate buffer 100 mM (pH 6.8, 40 μL), α-glucosidase 0.4 U mL^−1^ (25 μL), and *p*-nitrophenyl α-d-glucopyranoside 2.5 mM (25 μL). The reaction was stopped after 30 min by adding 100 μL of 0.1 M Na_2_CO_3_. The enzyme activities were measured based on the absorbance at *λ*_max_ 405 nm of the test samples. The control assay was prepared using the same procedure, except the test samples were replaced with distilled water; meanwhile, the activity of the reference was tested by replacing the sample with acarbose.^37^ Percentage inhibition was calculated as follows:^38^α-Glucosidase inhibition (%) = [Abs_(control)_ − Abs_(sample)_]/Abs_(control)_ × 100%

### 2.2. Computation

#### 2.2.1. Molecular docking simulation

The MOE 2015.10 software was used for the molecular docking simulation. Its requisite input includes structural information of the docking participants, *i.e.*, 21 *Dipterocarpus alatus*-derived ligands and 3 diabetes-related protein structures. The former is dipterocarpol (1), 13 oxime esters (2 and 3a–3m), and 7 lactones (4, 5, and 6a–6e) and the latter is glycoside–hydrolase proteins (3W37 and 3AJ7) and protein tyrosine phosphatase 1B (PTP1B). The formation of each ligand–protein inhibitory structure was primarily evaluated using a variety of output parameters, *e.g.*, docking score (DS) energy, root-mean-square deviation (RMSD), interaction types, and distance between ligands and proteins. A typical docking procedure followed three steps.^39–42^

##### Pre-docking preparation

Structural information of three proteins 3W37, 3AJ7, and PTP1B was obtained from the Worldwide Protein Data Bank and UniProtKB: α-glucosidase protein 3W37 (DOI: 10.2210/pdb3W37/pdb), oligo-1,6-glucosidase protein 3AJ7 (DOI: 10.2210/pdb3AJ7/pdb), and tyrosine phosphatase protein 1B PTP1B (entry: UniProtKB-A0A0U1XP67). Sequence Editor in the MOE 2015.10 program was used to remove the water molecules in the protein structure. The Quickprep tool was then used to prepare the structure of the proteins and their 3D protonation, configured as: tethered ligand–receptor with the strength of 5000 and refinement of 0.0001 kcal mol^−1^ Å^−1^. The active zones of the proteins were determined based on the possible interactability between their amino acids and the inhibitory ligands within a radius of 4.5 Å. The protein structural data obtained was saved in the *.pdb format. The ligands were under energy optimisation *via* Conj Grad and Termination. The configuration was set with an energy change of 0.0001 kcal mol^−1^; maximum number of interactions of 1000; and a modified Gasteiger–Huckel charge.

##### Docking investigation

After input preparation, intermolecular interaction simulation was performed on MOE 2015.10 and the complex structures were saved in the *.sdf format. The docking simulation parameters were configured as follows: the number of poses retained for further inhibition analysis = 10; the maximum number of solutions per iteration = 1000; and the maximum number of solutions per fragmentation = 200.

##### Post-docking analysis

The docking score (DS) predicts the binding affinity of ligands and their targeted protein in the site–site distance. The conformation of the docked complexes was visualised on 2D and 3D planes. The interactions formed in the active sites between the ligands and amino acids in the site–site distance of their targeted protein were also analysed. They include hydrogen bonds, ion bonds, arene–arene (π–π), cation–arene (cations–π), and van der Waals interactions detected during the simulation. In addition, root-mean-square deviation (RMSD) calculated provides information on the rigidity of the conformational binding of the docked complexes.

#### 2.2.2. Physicochemical and pharmaceutical compatibility

The docking parameters including DS_average_ (kcal mol^−1^), molecular mass (Da), polarizability (Å^3^) and volume or size (Å), and dispersion coefficients (log *P* and log *S*) were achieved with the Gasteiger–Marsili method using the QSARIS system.^43^ They were then pre-screened in an attempt to evaluate their oral pharmacological compatibility. This was based on Lipinski's rule of five, a well-known set of indicators to predict drug-likeness.^44^ According to Lipinski's criteria, a good membrane-permeable molecule should satisfy the following requirements: (1) molecular mass of <500 Da; (2) no more than 5 groups for hydrogen bonds; (3) no more than 10 groups receiving hydrogen bonds; and (4) log *P* value of less than +5 (log *P* < 5).^45,46^

## 3. Results and discussion

### 3.1. Experimentation

The synthesis of the oxime ester and lactone derivatives from dipterocarpol is schematically described in Schemes 1 and 2, respectively. Firstly, dipterocarpol was allowed to react with hydroxylamine hydrochloride/triethylamine in ethanol for 24 h to obtain oxime 2 in 83% yield. This product was then acylated with carboxylic acids (3a–3i), chloride acids (3l and 3m) or acetic anhydride/pyridine (3k) to afford a series of oxime ester 3a–3m (Scheme 1). It was noted that in the case of anhydride or chloride acid, if the reaction was prolonged for more than 24 h, diester derivatives at C-3 (major) and C-20 (minor) were formed.

To the best of our knowledge, there was only one study reported on the oxidative cleavage at the double bond (C-24 and C-25) of dipterocarpol. The authors used ozone/CH_2_Cl_2_ or ozone/MeOH to obtain hemiacetal or acetal, respectively.^21^ In our case, other oxidative cleavage reagents were utilised such as CrO_3_, SeO_2_, KMnO_4_. Consequently, the use of CrO_3_/CH_3_COOH (Jones oxidation) afforded the lactone products (compound 4) under mild conditions (room temperature, 40 min) in good yield (85.5%). Other oxidative reagents gave several unwanted products, which could not be purified after the reaction. Under Jones oxidation, the aldehyde formed at C-24 was further oxidised to carboxylic acid, which continuously underwent an intramolecular reaction with the hydroxyl group at C-20 to form lactone 4 in acidic condition during oxidation. The reduction of the ketone group at C-3 of compound 4 to hydroxyl group was carried out with NaBH_4_ in MeOH for 20 min. Compound 5 was obtained in 74% yield. Due to the steric hindrance in the β-site of the A-ring of 4, the BH_4_^−^ anion can only approach the C-3-keto group from the α-site, and thus the 3β-hydroxyl product 5 is the major product. Compound 5 was the only product obtained after column chromatography of the reduction mixture. Further, 5 was reacted with anhydric acid/pyridine to produce 6a, with chloride acid to give 6b and 6c, and with carboxylic acid/DCC/DMAP to obtain 6d and 6e in good yields.

### 3.2. Characteristics

#### 3.2.1. Spectroscopic data

##### Compound 1 (dipterocarpol)

White needles, mp 128.4–130.3 °C; C_30_H_50_O_2,_ ESI-MS (*m*/*z*): 443.2 [M + H]^+^, 425.2 [M + H–H_2_O]^+^; ^1^H NMR (500 MHz, CDCl_3_, *δ*_H_, ppm), (*J*, Hz): 5.13–5.10 (1H, m), 2.53–2.39 (2H, m), 2.08–2.02 (2H, m), 1.94–1.89 (1H, m), 1.85–1.83 (1H, m), 1.76–1.71 (2H, m), 1.69 (3H, s), 1.62 (3H, s), 1.59–1.23 (16H), 1.15 (3H, s), 1.08 (3H, s), 1.03 (3H, s), 1.00 (3H, s), 0.94 (3H, s), 0.89 (3H, s). ^13^C NMR (125 MHz, CDCl_3_, *δ*_C_, ppm): 218.08, 131.63, 124.69, 75.36, 55.38, 50.26, 50.02, 49.84, 47.42, 42.40, 40.47, 40.29, 39.89, 36.84, 34.56, 34.11, 31.15, 27.53, 26.71, 25.72, 25.48, 24.80, 22.56, 22.04, 21.00, 19.66, 17.70, 16.35, 16.00, 15.22.

##### Compound 2 [20(*S*)-hydroxydammar-24-ene-3-oxime]

White amorphous powder, C_30_H_51_NO_2_, ESI-MS (*m*/*z*): 458.3 [M + H]^+^, 440.2 [M + H–H_2_O]^+^; ^1^H-NMR (500 MHz, CDCl_3_, *δ*_H_, ppm), (*J*, Hz): 5.12 (t, 1H, *J* = 7.0), 2.97–2.92 (1H, m), 2.31–2.25 (1H, m), 2.07–2.02 (2H, m), 1.84–1.78 (2H, m), 1.75–1.72 (2H, m), 1.68 (3H, s), 1.62 (3H, s), 1.14 (3H, s), 1.14 (3H, s), 1.05 (3H, s), 0.99 (3H, s), 0.94 (3H, s), 0.86 (3H, s). ^13^C-NMR (125 MHz, CDCl_3_, *δ*_C_, ppm): 167.40, 131.62, 124.74, 75.41, 56.03, 50.31, 50.30, 49.87, 42.37, 40.53, 40.43, 40.37, 39.08, 37.20, 34.86, 31.16, 27.56, 27.41, 25.73, 25.47, 24.81, 22.88, 22.58, 21.84, 19.07, 17.72, 17.03, 16.37, 15.89, 15.41.

##### Compound 3a {20(*S*)-hydroxy dammar-24-ene-3-oximyl-[5-methyl-3,6-diazine-1-carboxylate]}

White amorphous powder, C_36_H_55_N_3_O_3_, HR-ESI-MS (*m*/*z*): 600.4285 [M + Na]^+^ (calculated for C_36_H_55_N_3_O_3_Na 600.4245); ^1^H-NMR (500 MHz, CDCl_3_, *δ*_H_, ppm), (*J*, Hz): 9.18 (1H, d, *J* = 1.5), 8.58 (1H, d, *J* = 1.5), 5.13–5.10 (1H, m), 2.98–2.93 (1H, m), 2.67 (3H, s), 2.61–2.54 (1H, m), 2.07–2.02 (2H, m), 1.68 (3H, s), 1.56 (3H, s), 1.33 (3H, s), 1.21 (3H, s), 1.14 (3H, s), 1.00 (3H, s), 0.97 (3H, s), 0.88 (3H, s). ^13^C-NMR (125 MHz, CDCl_3_, *δ*_C_, ppm): 177.84, 162.08, 157.72, 145.27, 144.33, 140.69, 131.65, 124.71, 75.38, 55.93, 50.28, 49.89, 42.35, 41.89, 40.47, 40.42, 39.47, 37.17, 34.73, 33.98, 31.15, 27.51, 27.48, 25.74, 25.51, 24.81, 22.70, 22.58, 21.97, 21.94, 20.18, 19.03, 17.71, 16.36, 16.21, 15.37.

##### Compound 3b [20(*S*)-hydroxydammar-24-ene-3-oximyl cinnamate]

White amorphous powder, C_39_H_57_NO_3_, HR-ESI-MS (*m*/*z*): 610.4251 [M + Na]^+^ (calculated for C_39_H_57_NO_3_Na 610.4236), ESI-MS (*m*/*z*): 588.1 [M + H]^+^; ^1^H-NMR (500 MHz, CDCl_3_, *δ*_H_, ppm), (*J*, Hz): 7.76 (1H, d, *J* = 16.0), 7.56–7.54 (2H, m), 7.39–7.36 (3H, m), 6.59 (1H, d, *J* = 16.0), 5.12 (1H, t, *J* = 7.0), 2.89–2.83 (1H, m), 2.49–2.48 (1H, m), 2.06–2.03 (2H, m), 1.67 (3H, s), 1.60 (3H, s), 1.26 (3H, s), 1.18 (3H, s), 1.14 (3H, s), 1.06 (3H, s), 0.99 (3H, s), 0.87 (3H, s). ^13^C-NMR (125 MHz, CDCl_3_, *δ*_C_, ppm): 175.74, 165.25, 147.71, 134.52, 131.61, 130.38, 128.90, 128.15, 124.71, 116.43, 75.36, 55.95, 50.27, 49.87, 42.33, 41.60, 40.46, 40.40, 39.45, 39.16, 37.14, 34.73, 31.13, 31.03, 27.51, 27.45, 26.34, 25.72, 25.49, 24.79, 22.68, 22.57, 21.91, 19.74, 19.03, 17.70, 16.34, 16.17, 15.36.

##### Compound 3c [20(*S*)-hydroxydammar-24-ene-3-oximyl-benzoate]

White amorphous powder, C_37_H_55_NO_3_, HR-ESI-MS (*m*/*z*): 584.4167 [M + Na]^+^ (calculated for C_37_H_55_NO_3_Na 584.4142), ESI-MS (*m*/*z*): 562.2 [M + H]^+^; ^1^H-NMR (500 MHz, CDCl_3_, *δ*_H_, ppm), (*J*, Hz): 8.05 (2H, d, *J* = 7.5), 7.56 (1H, t, *J* = 7.5), 7.45 (2H, t, *J* = 7.5), 5.12 (1H, t, *J* = 7.5), 2.97–2.92 (1H, m), 2.58–2.51 (1H, m), 2.06–2.03 (2H, m), 1.68 (3H, s), 1.62 (3H, s), 1.33 (3H, s), 1.23 (3H, s), 1.14 (3H, s), 1.00 (3H, s), 0.97 (3H, s), 0.83 (3H, s). ^13^C-NMR (125 MHz, CDCl_3_, *δ*_C_, ppm): 176.48, 164.25, 132.94, 131.59, 129.79, 129.50, 128.44, 124.71, 75.35, 55.95, 50.26, 49.87, 42.33, 41.70, 40.45, 40.40, 39.47, 37.16, 34.73, 31.58, 31.14, 27.50, 27.47, 25.72, 25.49, 24.79, 22.72, 22.57, 21.92, 19.98, 19.03, 17.69, 16.34, 16.16, 15.36.

##### Compound 3d {20(*S*)-hydroxydammar-24-ene-3-oximyl-[2-hydroxy-3,5-diiodobenzoate]}

White amorphous powder, C_37_H_53_NO_4_I_2_, HR-ESI-MS (*m*/*z*): 852.1983 [M + Na]^+^ (calculated for C_37_H_53_NO_4_I_2_Na 852.1962), ESI-MS (*m*/*z*): 828.0 [M–H]^−^; ^1^H-NMR (500 MHz, CDCl_3_, *δ*_H_, ppm), (*J*, Hz): 11.65 (1H, s, OH), 8.21 (1H, d, *J* = 2.5), 8.07 (1H, d, *J* = 2.5), 5.13–5.10 (1H, m), 2.92–2.87 (1H, m), 2.60–2.54 (1H, m), 2.06–2.03 (2H, m), 1.75 (3H, s), 1.65 (3H, s), 1.29 (3H, s), 1.21 (3H, s), 1.14 (3H, s), 1.00 (3H, s), 0.98 (3H, s), 0.88 (3H, s). ^13^C-NMR (125 MHz, CDCl_3_, *δ*_C_, ppm): 178.20, 166.54, 160.49, 152.17, 137.72, 131.65, 124.68, 113.61, 87.23, 80.62, 75.35, 55.93, 50.26, 49.87, 42.32, 42.02, 40.43, 40.40, 39.44, 37.17, 34.68, 31.13, 27.47, 27.44, 25.72, 25.53, 24.79, 22.72, 22.57, 21.94, 20.34, 19.00, 17.70, 16.34, 16.17, 15.36.

##### Compound 3e {20(*S*)-hydroxydammar-24-ene-3-oximyl-[6-chloronicotinoate]}

White amorphous powder, C_36_H_53_ClN_2_O_3_, HR-ESI-MS (*m*/*z*): 619.3785 [M + Na]^+^ (calculated for C_36_H_53_ClN_2_O_3_Na 619.3759), ESI-MS (*m*/*z*): 598.2 [M + H]^+^; ^1^H-NMR (500 MHz, CDCl_3_, *δ*_H_, ppm), (*J*, Hz): 9.00 (1H, d, *J* = 2.0), 8.26 (1H, dd, *J* = 8.0, 2.0), 7.44 (1H, d, *J* = 8.0), 5.12 (1H, d, *J* = 7.0), 2.92–2.87 (1H, m), 2.58–2.52 (1H, m), 2.06–2.04 (2H, m), 1.75 (3H, s), 1.66 (3H, s), 1.33 (3H, s), 1.23 (3H, s), 1.14 (3H, s), 1.00 (3H, s), 0.87 (3H, s). ^13^C-NMR (125 MHz, CDCl_3_, *δ*_C_, ppm): 177.35, 162.14, 155.70, 150.79, 139.55, 131.58, 124.79, 124.70, 124.37, 75.31, 55.91, 50.25, 49.84, 42.30, 41.85, 40.47, 40.38, 39.41, 37.15, 34.69, 31.13, 27.46, 25.72, 25.49, 24.78, 22.69, 22.56, 21.92, 20.12, 19.01, 17.70, 16.33, 16.17, 15.35.

##### Compound 3f {20(*S*)-hydroxydammar-24-ene-3-oximyl-[4-chlorobenzoate]}

White amorphous powder, C_37_H_54_ClNO_3_, HR-ESI-MS (*m*/*z*): 630.3467 [M + Cl]^−^ (calculated for C_37_H_54_Cl_2_NO_3_ 630.3486); ^1^H-NMR (500 MHz, CDCl_3_, *δ*_H_, ppm), (*J*, Hz): 7.98 (2H, d, *J* = 8.5), 7.43 (2H, d, *J* = 8.5), 5.13–5.10 (1H, m), 2.94–2.89 (1H, m), 2.57–2.50 (1H, m), 2.06–2.03 (2H, m), 1.67 (3H, s), 1.62 (3H, s), 1.33 (3H, s), 1.24 (3H, s), 1.22 (3H, s), 1.08 (3H, s), 1.06 (3H, s), 0.87 (3H, s). ^13^C-NMR (125 MHz, CDCl_3_, *δ*_C_, ppm): 176.73, 163.41, 139.43, 131.60, 130.86, 128.83, 128.23, 124.71, 75.34, 55.94, 50.26, 49.89, 42.33, 41.75, 40.45, 40.41, 39.45, 37.17, 34.73, 31.13, 27.48, 25.71, 25.51, 24.79, 22.72, 22.57, 21.93, 20.02, 19.02, 17.69, 16.34, 16.16, 15.36.

##### Compound 3g {20(*S*)-hydroxydammar-24-ene-3-oximyl-[4-fluorobenzoate]}

White amorphous powder, C_37_H_54_NO_3_F, HR-ESI-MS (*m*/*z*): 602.4125 [M + Na]^+^ (calculated for C_37_H_54_NO_3_FNa 602.4097); ^1^H-NMR (500 MHz, CDCl_3_, *δ*_H_, ppm), (*J*, Hz): 8.07 (2H, dd, *J* = 8.0, 2.0), 7.12 (2H, dd, *J* = 8.0, 2.0), 5.12 (1H, t, *J* = 7.0), 2.94–2.91 (1H, m), 2.59–2.51 (1H, m), 2.06–2.03 (2H, m), 1.75 (3H, s), 1.71 (3H, s), 1.32 (3H, s), 1.20 (3H, s), 1.14 (3H, s), 1.00 (3H, s), 0.97 (3H, s), 0.87 (3H, s). ^13^C-NMR (125 MHz, CDCl_3_, *δ*_C_, ppm): 176.58, 166.76, 163.30, 132.05, 131.97, 131.59, 126.00, 125.97, 124.71, 115.55, 75.33, 55.93, 50.26, 49.87, 42.32, 41.72, 40.45, 40.40, 39.45, 37.16, 34.72, 31.13, 27.49, 27.47, 25.72, 25.50, 24.79, 22.71, 22.57, 21.92, 20.00, 19.02, 17.69, 16.34, 16.16, 15.35.

##### Compound 3h {20(*S*)-hydroxydammar-24-ene-3-oximyl-[2-iodobenzoate]}

White amorphous powder, C_37_H_54_NO_3_I, HR-ESI-MS (*m*/*z*): 710.3213 [M + Na]^+^ (calculated for C_37_H_54_NO_3_INa 710.3170); ^1^H-NMR (500 MHz, CDCl_3_, *δ*_H_, ppm), (*J*, Hz): 7.97 (1H, dd, *J* = 8.0, 1.0), 7.73 (1H, dd, *J* = 8.0, 2.0), 7.42–7.39 (1H, m), 7.17–7.13 (1H, m), 5.13–5.10 (1H, m), 2.96–2.92 (1H, m), 2.58–2.52 (1H, m), 2.06–2.03 (2H, m), 1.68 (3H, s), 1.62 (3H, s), 1.30 (3H, s), 1.22 (3H, s), 1.19 (3H, s), 0.99 (3H, s), 0.96 (3H, s), 0.83 (3H, s). ^13^C-NMR (125 MHz, CDCl_3_, *δ*_C_, ppm): 176.66, 164.81, 140.96, 135.84, 132.35, 131.54, 130.66, 127.84, 124.70, 93.60, 75.30, 55.96, 50.26, 50.25, 49.86, 42.31, 41.75, 40.45, 40.39, 39.56, 37.15, 34.72, 31.12, 27.48, 27.41, 25.69, 25.49, 24.78, 22.62, 22.55, 21.91, 20.23, 19.00, 17.67, 16.34, 16.21, 15.34.

##### Compound 3i {20(*S*)-hydroxydammar-24-ene-3-oximyl-[3-fluorobenzoate]}

White amorphous powder, C_37_H_54_FNO_3_, HRESI-MS (*m*/*z*): 602.3981 [M + Na]^+^ (calculated for C_37_H_54_FNO_3_Na 602.3985); ^1^H-NMR (500 MHz, CDCl_3_, *δ*_H_, ppm), (*J*, Hz): 7.85 (1H, dd, *J* = 7.5, 1.0), 7.72 (1H, dd, *J* = 7.5, 4.0), 7.45–7.41 (1H, m), 7.29–7.25 (1H, m), 5.13–5.10 (1H, m), 2.95–2.90 (1H, m), 2.58–2.52 (1H, m), 2.06–2.03 (2H, m), 1.68 (3H, s), 1.62 (3H, s), 1.32 (3H, s), 1.21 (3H, s), 1.14 (3H, s), 1.10 (3H, s), 0.97 (3H, s), 0.87 (3H, s). ^13^C-NMR (125 MHz, CDCl_3_, *δ*_C_, ppm): 176.75, 163.51, 161.54, 131.93, 131.87, 131.50, 130.11, 130.05, 125.20, 124.69, 75.26, 55.89, 50.22, 49.83, 42.28, 41.70, 40.43, 40.36, 39.40, 37.12, 34.68, 31.09, 27.45, 25.67, 25.45, 24.75, 22.67, 22.53, 21.89, 19.98, 18.98, 17.64, 16.30, 16.11, 15.31.

##### Compound 3k [20(*S*)-hydroxydammar-24-ene-3-oximyl acetate]

White amorphous powder, C_32_H_53_NO_3_, HR-ESI-MS (*m*/*z*): 522.4045 [M + Na]^+^ (calculated for C_32_H_53_NO_3_Na 522.3997); ^1^H-NMR (500 MHz, CDCl_3_, *δ*_H_, ppm), (*J*, Hz): 5.13–5.10 (1H, m), 2.86–2.81 (1H, m), 2.45–2.39 (1H, m), 2.17 
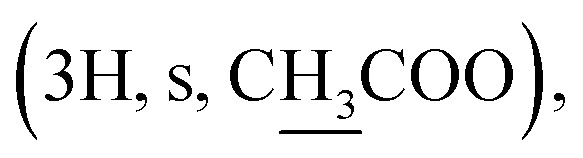
 2.06–2.03 (2H, m), 1.67 (3H, s), 1.57 (3H, s), 1.23 (3H, s), 1.14 (3H, s), 1.13 (3H, s), 0.98 (3H, s), 0.94 (3H, s), 0.86 (3H, s). ^13^C-NMR (125 MHz, CDCl_3_, *δ*_C_, ppm): 174.86, 169.85, 131.57, 124.70, 75.34, 55.89, 50.25, 49.84, 42.31, 41.39, 40.47, 40.38, 39.35, 37.09, 34.71, 31.12, 27.49, 27.40, 25.71, 25.47, 24.78, 22.62, 22.56, 21.89, 20.00, 19.52, 19.00, 17.69, 16.32, 16.13, 15.34.

##### Compound 3l [20(*S*)-hydroxydammar-24-ene-3-oximyl pentanoate]

White amorphous powder, C_35_H_59_NO_3_, HR-ESI-MS (*m*/*z*): 542.4588 [M + H]^+^ (calculated for C_35_H_60_NO_3_ 542.4567); ^1^H-NMR (500 MHz, CDCl_3_, *δ*_H_, ppm), (*J*, Hz): 5.13–5.10 (1H, m), 2.84–2.79 (1H, m), 2.42 (2H, t, *J* = 7.5, OCOCH_2_), 2.41–2.37 (1H, m), 2.07–2.01 (2H, m), 1.70 (3H, s), 1.62 (3H, s), 1.24 (3H, s), 1.15 (3H, s), 1.14 (3H, s), 0.98 (3H, s), 0.94 (3H, s), 0.92 (3H, t, *J* = 7.5), 0.86 (3H, s). ^13^C-NMR (125 MHz, CDCl_3_, *δ*_C_, ppm): 175.07, 172.07, 131.56, 124.71, 75.33, 55.90, 50.25, 49.85, 42.32, 41.45, 40.46, 40.38, 39.37, 37.10, 34.72, 32.92, 31.12, 27.49, 25.47, 24.78, 22.63, 22.56, 22.31, 21.89, 19.55, 19.00, 17.68, 16.32, 16.11, 15.34, 13.69.

##### Compound 3m [20(*S*)-hydroxydammar-24-ene-3-oximyl isobutyrate]

White amorphous powder, C_34_H_57_NO_3_, HR-ESI-MS (*m*/*z*): 550.4378 [M + Na]^+^ (calculated for C_34_H_57_NO_3_Na 550.4330), ESI-MS (*m*/*z*): 528.2 [M + H]^+^; ^1^H-NMR (500 MHz, CDCl_3_, *δ*_H_, ppm), (*J*, Hz): 5.13–5.10 (1H, m), 2.85–2.79 (1H, m), 2.72–2.66 (1H, m), 2.43–2.37 (1H, m), 2.07–2.03 (2H, m), 1.69 (3H, s), 1.62 (3H, s), 1.25 (3H, s), 1.23 (6H, d, *J* = 7.0), 1.14 (6H, brs), 0.98 (3H, s), 0.94 (3H, s), 0.86 (3H, s). ^13^C-NMR (125 MHz, CDCl_3_, *δ*_C_, ppm): 175.59, 174.79, 131.61, 124.70, 75.39, 55.93, 50.25, 49.86, 42.32, 41.53, 40.44, 40.39, 39.39, 37.11, 34.73, 33.19, 31.12, 27.49, 27.33, 25.71, 25.48, 24.79, 22.65, 22.56, 21.89, 19.54, 19.06, 19.03, 18.98, 17.69, 16.32, 16.10, 15.35.

##### Compound 4 {17-[(5α-methyl)-3,4*H*-furane-2-one]-25,26,27-tris-nor-dammar-3-one}

White needles, mp 118.4–120.3 °C; C_27_H_42_O_3_, HR-ESI-MS (*m*/*z*): 415.3226 [M + H]^+^ (calculated for C_27_H_43_O_3_ 415.3206), ESI-MS (*m*/*z*): 415.1 [M + H]^+^, 397.1 [M–H_2_O + H]^+^; ^1^H-NMR (500 MHz, CDCl_3_, *δ*_H_, ppm), (*J*, Hz): 2.55–2.50 (1H, m, H-23a), 2.49–2.47 (1H, m, H-22a), 2.45–2.41 (1H, m, H-23b), 2.38–2.35 (1H, m, H-2a), 2.06–2.04 (1H, m, H-17), 1.30 (3H, s, H-21), 1.01 (3H, s, H-25), 0.97 (3H, s, H-26), 0.93 (3H, s, H-18), 0.87 (3H, s, H-19), 0.83 (3H, s, H-27). ^13^C-NMR (125 MHz, CDCl_3_, *δ*_C_, ppm): 217.88 (C-3), 176.69 (C-24, C̲O lactone), 90.02 (C-20), 55.34 (C-5), 50.15, 49.93 (C-9), 49.30 (C-17), 47.40, 43.31 (C-13), 40.28, 39.85 36.83, 34.53, 34.07, 31.18, 31.09, 29.16, 26.83, 26.70, 25.49, 25.02, 21.93, 21.02, 19.61, 16.16, 15.98, 15.19.

##### Compound 5 {3β-hydroxy-17-[(5α-methyl)-3,4*H*-furane-2-one]-25,26,27-tris-nor-dammarane}

White needles, mp 195.0–196.0 °C, C_27_H_44_O_3_, HR-ESI-MS (*m*/*z*): 417.3378 [M + H]^+^ (calculated for C_27_H_45_O_3_ 417.3363); ^1^H-NMR (500 MHz, CDCl_3_, *δ*_H_, ppm), (*J*, Hz): 3.19 (1H, dd, *J* = 11.5, 5.0), 2.68–2.62 (1H, m), 2.57–2.50 (1H, m), 2.15–2.08 (1H, m), 2.01–1.90 (2H, m), 1.37 (3H, s), 0.98 (3H, s), 0.97 (3H, s), 0.88 (3H, s), 0.85 (3H, s), 0.77 (3H, s). ^13^C-NMR (125 MHz, CDCl_3_, *δ*_C_, ppm): 176.75, 90.14, 78.93, 55.85, 50.58, 50.18, 49.37, 43.20, 40.38, 39.04, 38.98, 37.14, 35.21, 31.21, 31.10, 29.20, 28.00, 27.40, 26.82, 25.44, 25.05, 21.43, 18.25, 16.25, 16.18, 15.48, 15.35.

##### Compound 6a {17-[(5α-methyl)-3,4*H*-furane-2-one]-3β-(*O*-acetyl)-25,26,27-tris-nor-dammarane}

White needles, mp 248.0–249.0 °C, C_29_H_46_O_4_, HR-ESI-MS (*m*/*z*): 481.3357 [M + Na]^+^ (calculated for C_29_H_46_O_4_Na 481.3294), ESI-MS (*m*/*z*): 459.1 [M + H]^+^; ^1^H-NMR (500 MHz, CDCl_3_, *δ*_H_, ppm), (*J*, Hz): 4.48 (1H, dd, *J* = 11.0, 5.5), 2.68–2.62 (1H, m), 2.57–2.50 (1H, m), 2.14–2.10 (1H, m), 2.04 
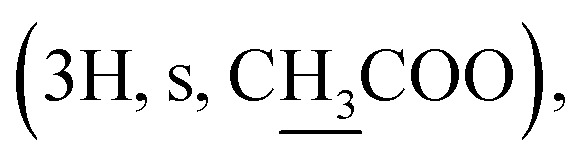
 2.00–1.90 (2H, m), 1.37 (3H, s), 0.96 (3H, s), 0.88 (3H, s), 0.86 (3H, s), 0.84 (6H, brs). ^13^C-NMR (125 MHz, CDCl_3_, *δ*_C_, ppm): 176.75, 170.98, 90.10, 80.86, 55.93, 50.50, 50.16, 49.36, 43.18, 40.39, 38.71, 37.90, 37.05, 35.14, 31.22, 31.19, 29.18, 27.96, 26.80, 25.37, 25.04, 23.68, 21.43, 21.29, 18.13, 16.48, 16.24, 16.22, 15.48.

##### Compound 6b {17-[(5α-methyl)-3,4*H*-furane-2-one]-3β-(*O*-pentanoyl)-25,26,27-tris-nor-dammarane}

White needles, mp 134.0–135.0 °C, C_32_H_52_O_4_, HR-ESI-MS (*m*/*z*): 523.3892 [M + Na]^+^ (calculated for C_32_H_52_O_4_Na 523.3858), ESI-MS (*m*/*z*): 501.3 [M + H]^+^; ^1^H-NMR (500 MHz, CDCl_3_, *δ*_H_, ppm), (*J*, Hz): 4.48 (1H, dd, *J* = 11.0, 5.0), 2.65–2.60 (1H, m), 2.56–2.50 (1H, m), 2.29 (2H, t, *J* = 7.5), 2.14–2.10 (1H, m), 2.01–1.90 (2H, m), 1.37 (3H, s), 0.96 (3H, s), 0.91 (3H, t, *J* = 7.5), 0.88 (3H, s), 0.86 (3H, s), 0.85 (6H, brs). ^13^C-NMR (125 MHz, CDCl_3_, *δ*_C_, ppm): 176.73, 173.64, 90.09, 80.50, 55.93, 50.48, 50.17, 49.37, 43.19, 40.40, 38.70, 37.94, 37.06, 35.14, 34.56, 31.22, 31.19, 29.17, 27.97, 27.23, 26.80, 25.36, 25.04, 23.72, 22.29, 21.44, 18.13, 16.54, 16.23, 15.48, 13.70.

##### Compound 6c (17-[(5α-methyl)-3,4*H*-furane-2-one]-3β-(*O*-isobutyryl)-25,26,27-tris-nor-dammarane)

White needle crystals, mp 206.0–207.0 °C; C_31_H_50_O_4_, HR-ESI-MS (*m*/*z*): 509.3683 [M + Na]^+^ (calculated for C_31_H_50_O_4_Na 509.3671), ESI-MS (*m*/*z*): 487.2 [M + H]^+^; ^1^H-NMR (500 MHz, CDCl_3_, *δ*_H_, ppm), (*J*, Hz): 4.48–4.44 (1H, m), 2.68–2.62 (1H, m), 2.57–2.50 (1H, m), 2.14–2.10 (1H, m), 2.01–1.90 (1H, m), 1.39 (3H, s), 1.17 (3H, d, *J* = 7.5), 1.16 (3H, d, *J* = 7.5), 0.96 (3H, s), 0.88 (3H, s), 0.87 (3H, s), 0.86 (3H, s), 0.84 (3H, s). ^13^C-NMR (125 MHz, CDCl_3_, *δ*_C_, ppm): 176.79, 176.73, 90.09, 80.27, 55.93, 50.48, 50.18, 49.37, 43.19, 40.40, 38.67, 38.06, 37.07, 35.14, 34.47, 31.23, 31.20, 29.17, 27.95, 26.80, 25.36, 25.04, 23.64, 21.44, 19.18, 18.93, 18.11, 16.54, 16.25, 16.22, 15.48.

##### Compound 6d {17-[(5α-methyl)-3,4*H*-furane-2-one]-3β-(*O*-salycyloyl)-25,26,27-tris-nor-dammarane}

White needles, mp 186.0–187.0 °C; C_34_H_48_O_5_, HR-ESI-MS (*m*/*z*): 559.3519 [M + Na]^+^ (calculated for C_34_H_48_O_5_Na 559.3494), ESI-MS (*m*/*z*): 537.1 [M + H]^+^; ^1^H-NMR (500 MHz, CDCl_3_, *δ*_H_, ppm), (*J*, Hz): 7.83 (1H, dd, *J* = 8.0, 2.0), 7.46–7.42 (1H, dt, *J* = 7.5, 1.5), 6.98 (1H, dd, *J* = 8.0, 1.0), 6.87 (dt, *J* = 8.0, 1.0), 5.29 (1H, s, OH), 4.77–4.74 (1H, m), 2.67–2.61 (1H, m), 2.57–2.54 (1H, m), 2.13–2.09 (1H, m), 1.36 (3H, s), 1.01 (3H, s), 0.98 (3H, s), 0.93 (3H, s), 0.92 (3H, s), 0.90 (3H, s). ^13^C-NMR (125 MHz, CDCl_3_, *δ*_C_, ppm): 176.70, 169.90, 161.74, 135.44, 129.76, 119.04, 117.58, 113.09, 90.06, 82.27, 56.00, 50.51, 50.19, 49.37, 43.19, 40.43, 38.70, 38.30, 37.11, 35.13, 31.20, 29.69, 29.17, 28.14, 26.81, 25.41, 25.04, 23.71, 21.48, 18.15, 16.73, 16.25, 15.51.

##### Compound 6e {17-[(5α-methyl)-3,4*H*-furane-2-one]-3β-(*O*-cinnamoyl)-25,26,27-tris-nor-dammarane}

White crystals, mp 236.0–238.0 °C; C_36_H_50_O_4_, HR-ESI-MS (*m*/*z*): 583.5987 [M + H + 2H_2_O]^+^ (calculated for C_36_H_55_O_6_ 583.3999), ^1^H-NMR (500 MHz, CDCl_3_, *δ*_H_, ppm), (*J*, Hz): 7.65 (1H, d, *J* = 16.0), 7.53–7.51 (2H, m), 7.39–7.37 (3H, m), 6.43 (1H, d, *J* = 16.0), 4.62 (1H, dd, *J* = 11.0, 5.0), 2.64–2.61 (1H, m), 2.57–2.54 (1H, m), 2.13–2.00 (1H, m), 1.50 (3H, s), 1.36 (3H, s), 0.97 (3H, s), 0.93 (3H, s), 0.90 (6H, brs). ^13^C-NMR (125 MHz, CDCl_3_, *δ*_C_, ppm): 176.73, 166.81, 144.29, 134.59, 130.12, 128.85, 128.03, 118.88, 90.09, 80.94, 55.98, 50.52, 50.19, 49.38, 43.21, 40.42, 38.76, 38.17, 37.10, 35.16, 31.25, 31.20, 29.18, 28.04, 26.82, 25.36, 25.05, 23.80, 21.46, 18.16, 16.65, 16.25, 15.50.

#### 3.2.2. Structural elucidation

Compound 1 was isolated from *Dipterocarpus alatus* as colourless needles. The NMR spectral data of this compound is consistent with the reported data.^27,30^ This compound was used as the starting material for the synthesis of the oxime and lactone derivatives.

Instead of a ketone signal (*δ*_C_ 218.08) in the ^13^C NMR spectrum of 1, a signal at *δ*_C_ 167.40, which is characteristic of an oxime carbon at C-3, was observed for compound 2. The formation of oxime esters 3a–3m was confirmed by the appearance of carbonyl ester groups in the range of *δ*_C_ 178.02–174.48 in their ^13^C NMR spectra. The signals representing the aromatic carbons of compounds 3a–3i are resonated at *δ*_C_ 163.03–124.37 and *δ*_H_ 9.17–7.12 in their NMR spectra. The ^1^H NMR spectrum of compound 3k provides an additional methyl signal at *δ*_H_ 2.17 
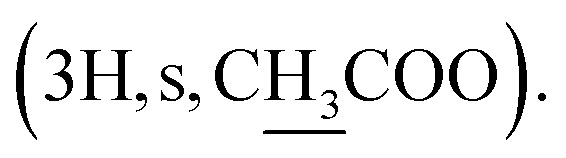
 The signals at *δ*_H_ 2.42 
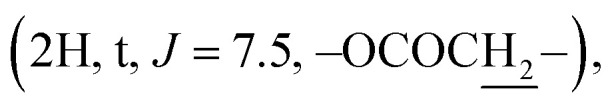
 and 0.92 
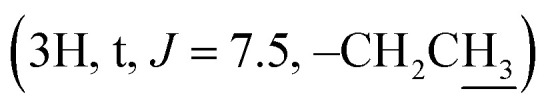
 demonstrate the presence of a pentanoyl group in compound 3l. The presence of an isobutyryl group is inferred by the signal at *δ*_H_ 1.23 (6H, d, *J* = 6.0) in the ^1^H NMR spectrum of compound 3m. The NMR spectra of compound 4 indicate the disappearance of the olefine bond at C-24 and C-25 and the two methyl groups at C-26, C-27, compared with compound 1. The presence of a carbonyl lactone at C-24 (*δ*_C_ 176.69), together with the much more down-field chemical shift of the tertiary oxygenated carbon (*δ*_C_ 90.02) suggests the formation of a lactone ring at the side chain of the dipterocarpol skeleton in compound 4. The structure of 4 was further confirmed by 2D NMR (COSY, HSQC, HMBC, and NOESY) spectra. The HMBC correlations between H-23 (*δ*_H_ 2.55–2.50), H-22 (*δ*_H_ 2.49–2.47)/C-24 (*δ*_C_ 176.69), C-20 (*δ*_C_ 90.02), H-21 (*δ*_H_ 1.30)/C-20 (*δ*_C_ 90.02) and C-17 (49.30) confirm the location of the lactone ring at C-17. The orientation of the methyl group (C-21) was established as α by the NOESY correlation between H-17 (*δ*_H_ 2.06–2.04) and H-21 (*δ*_H_ 1.30).

The ^1^H NMR spectrum of compound 5 presents a hydroxymethine group at *δ*_H_ 3.19 (1H, dd, *J* = 11.5, 5.0, H-3). The large coupling constant (*J* = 11.5) suggests the axial orientation of proton H-3 and the β-configuration of the hydroxyl group at C-3. An oxygenated methine carbon (*δ*_C_ 78.93) was observed in the ^13^C NMR spectrum of compound 5, instead of a ketone group (*δ*_C_ 217.88) in compound 4. The ^13^C NMR spectra of compounds 6a–6e contain additional signals of carbonyl ester in the range of *δ*_C_ 176.63–170.98. The signals at *δ*_C_ 144.29–113.09 and *δ*_H_ 7.83–6.43 indicate the presence of aromatic rings in compounds 6d and 6e. A methyl carbonyl ester at *δ*_H_ 2.04 
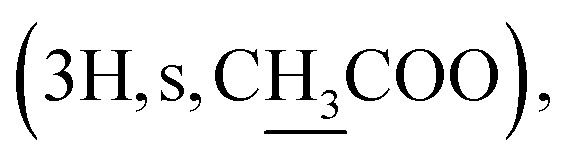
 a pentanoyl group at *δ*_H_ 2.29 
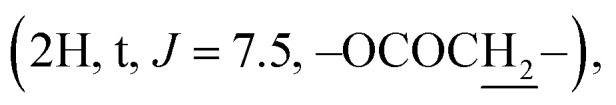
 and an isobutyryl group at *δ*_H_ 1.17 (3H, d, *J* = 7.5), 1.16 (3H, d, *J* = 7.5) were determined from the ^1^H NMR spectra of compounds 6a, 6b and 6c, respectively.

To the best of our knowledge, the twelve oxime esters and seven lactones are new dammarane derivatives that have not been reported in the literature to date. The analysis of the HR-ESIMS spectra data of all these newly synthesised compounds provided in Section 3.2 (characteristics) also further confirms the elucidation of their structures.

### 3.3. Enzyme inhibition

#### 3.3.1. Experimental assays of α-glucosidase inhibition

Twenty-one compounds (1, 2, 3a–3m, 4, 5, and 6a–6e) were evaluated for their ability to inhibit α-glucosidase *in vitro*, and the corresponding results are given in Table 1.

**Table 1:** Assay-based inhibition of the investigated compounds (dipterocarpol and its derivatives) towards α-glucosidase^a^

Number	Sample/compound	IC_50_ value ± SD (μM)
1	1	264.91 ± 9.64
2	2	36.94 ± 2.80
3	3a	NA
4	3b	33.12 ± 5.37
5	3c	16.38 ± 1.00
6	3d	NA
7	3e	36.60 ± 1.26
8	3f	23.98 ± 1.29
9	3g	NA
10	3h	NA
11	3i	46.79 ± 5.33
12	3k	59.15 ± 2.08
13	3l	43.51 ± 1.68
14	3m	NA
15	4	NA
16	5	2.73 ± 0.05
17	6a	220.5 ± 4.77
18	6b	31.95 ± 0.69
19	6c	4.62 ± 0.12
20	6d	228.09 ± 4.62
21	6e	7.31 ± 0.11

a Acarbose (IC_50_ = 183.44 ± 3.78 μM) and NA: no activity (IC_50_ > 390 μM).

The starting material dipterocarpol (1) was also tested for comparison with its synthetic derivatives. Acarbose was used as the standard compound in this assay. The results showed that dipterocarpol possesses weak activity (IC_50_ 264.91 μM) compared with acarbose (IC_50_ 183.44 μM). Nevertheless, most of its oxime ester derivatives (2, 3b, 3c, 3e, 3f, 3i, 3k, and 3l) exhibited better the bio-activity than the standard compound acarbose towards α-glucosidase, as shown by their IC_50_ values of 16.38–59.15 μM. The benzoate derivative (3c) is the most active compound in the oxime ester series with an IC_50_ of 16.38 μM. It is followed by the parachlorobenzoate (3f, IC_50_ of 23.98 μM) and cinnamate (3b, IC_50_ of 33.12 μM) derivatives. The substitution of monoiodine (3h) and diodine benzoate (3d) led to the loss of their activity. Compound 3i with *meta*-fluorobenzoate exhibited good activity with an IC_50_ value of 46.79 μM. However, the installation of *para*-fluorobenzoate (3g) resulted in no α-glucosidase inhibition. The above-mentioned inhibitory effect suggests that the presence of the halogen substitutions in the aromatic rings increase the overall inhibition in the order of Cl > F > I. The heterocyclic oxime ester with a nicotinic ring (3e) showed good activity with an IC_50_ of 36.60 μM; in contrast, the pyrazine derivative was observed to be inactive. The acetate (3k) and pentanoate (3l) derivatives were found to exhibit good activity with the corresponding IC_50_ of 59.15 and 43.51 μM, respectively. The results suggest that the chain length of the alkoxy substitution may improve the α-glucosidase inhibitory activity. The isobutyrate (3m) oxime ester was inactive in this test. This means that the bulkiness of the alkoxy oxime ester group seems to lower the activity overall.

Compound 4 with a lactone ring located at C-17 was inactive to the α-glucosidase inhibition; however, the reduction of the ketone to hydroxyl group at C-3 led to potential inhibitory activity. Compound 5 with a free hydroxyl group was registered as the most active compound (IC_50_ of 2.73 μM) among the ester derivatives, as shown in Scheme 2. This evidence suggests that the hydroxyl group at C-3 plays an important role in α-glucosidase inhibition. Sufficiently increasing the chain length and bulkiness of the alkanoate moieties may enhance the activity. This was evidenced by the increase in activity, which followed the order of: 6a (IC_50_ 220.5 μM) < 6b (IC_50_ 31.95 μM) < 6c (IC_50_ 4.62 μM) (Table 1). The cinnamate derivative showed significantly better activity than the salicylate one. This observation suggests that the electron density of the benzene rings may have a negative correlation with the inhibitory activity of the host molecules towards α-glucosidase. Also, the dose–response curves of the most active inhibitors, *i.e.*, 5, 6c, and 6e, are provided in the ESI (Section 3; pages 203–205†).

In summary, it is obvious that the most significant inhibitors towards α-glucosidase, which correspond to IC_50_ values lower than 10 μM, follow the order of 5 > 6c > 6e. In particular, 5 demonstrates pronounced efficacy with the corresponding value of 2.73 ± 0.05 μM, accounting for the concentration required for 50% inhibition of the enzyme. This is followed by 3c > 3f > 6b > 3b > 3e > 2, whose IC_50_ values are in the range of 10–40 μM, and this considered highly effective. This group includes a mildly effective inhibitor, namely 3e, which is expected to be very different according to the docking-based simulation. Also, the experiments found that 3a, 3d, 3g, 3h, 3m, and 4 exhibit no inhibition activity towards α-glucosidase; nevertheless, the simulation gave the opposite prediction given 3d. The inconsistencies observed are readdressed and discussed in detail later in this report.

#### 3.3.2. Computational simulation of diabetes-related protein inhibitability

**Fig. 4:**
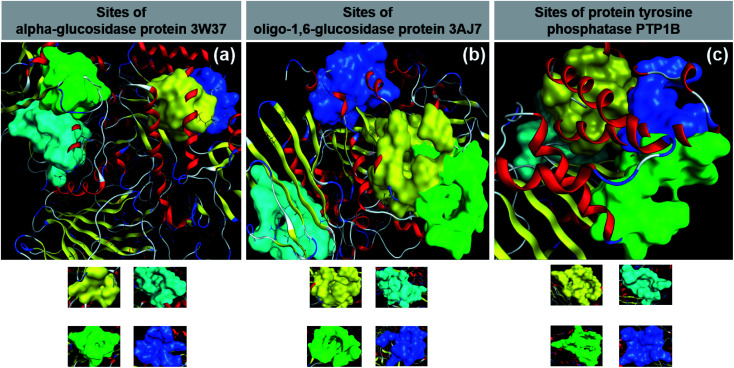
Quaternary structures of proteins (a) 3W37, (b) 3AJ7 and (c) PTP1B with their approachable sites by investigated compounds (1, 2, 3a–3m, 4, 5, and 6a–6e): site 1 (yellow), site 2 (cyan), site 3 (green), and site 4 (blue).

The static interaction between the inhibitory agents (*i.e.*, structure of the *Dipterocarpus alatus*-related compounds) and the targeted proteins (*viz.*, 3W37, 3AJ7, and PTP1B) was simulated using the molecular docking technique. The quaternary structure of the proteins and their approachable sites (by the ligands) are shown in Fig. 4. The docking results are summarised in Table 2, including the primary parameters, namely, the docking score (DS) energy, root-mean-square deviation (RMSD), number of hydrogen bonds, and number of van der Waals interactions. Detailed data is provided in the ESI (Sections 2.1–2.3; pages 194–202†). Overall, pre-screening of the two most important parameters,^39–41,47^*i.e.*, the DS values (representing static stability of an inhibitory system) and number of hydrogen bonds (representing the effectiveness of intermolecular binding, thus inducing conformational changes), indicated that 3W37, 3AJ7, and PTP1B are likely to be most susceptible at site 2, site 1, and site 1, respectively. As an exception, 6e is predicted to exhibit the highest inhibition towards PTP1B if in its site 2. The corresponding figures selected for further in-depth analysis are presented in bold.

**Table 2:** Docking-based inhibitability of the investigated ligands (1, 2, 3a–3m, 4, 5, and 6a–6e) towards the protein structures (3W37, 3AJ7 and PTP1B)^a^

C	3W37										3AJ7										PTP1B									
	Site 1		Site 2				Site 3		Site 4		Site 1				Site 2		Site 3		Site 4		Site 1				Site 2		Site 3		Site 4	
	E	H	E	H	V	R	E	H	E	H	E	H	V	R	E	H	E	H	E	H	E	H	V	R	E	H	E	H	E	H
1	−8.1	1	−10.9	2	14	1.24	−9.2	2	−9.8	2	−10.2	2	15	1.24	−9.4	1	−8.7	1	−9.3	1	−10.5	1	10	1.19	−8.2	0	−9.1	1	−7.4	0
2	−11.2	3	−12.9	4	5	1.67	−9.8	3	−10.4	3	−11.9	2	18	1.67	−8.7	1	−8.2	0	−10.6	1	−13.8	3	7	1.17	−11.4	2	−10.8	1	−9.7	1
3a	−8.1	1	−9.7	2	17	1.89	−8.7	1	−9.1	1	−9.5	3	27	1.89	−8.2	2	−7.3	1	−7.9	1	−9.3	2	11	1.25	−7.2	1	−8.1	1	−7.3	0
3b	−12.1	2	−13.4	3	13	1.78	−11.7	2	−10.9	1	−12.2	3	20	1.78	−9.8	1	−9.4	1	−11.7	2	−11.5	2	10	1.98	−8.0	0	−7.6	0	−9.3	1
3c	−8.2	1	−10.6	2	11	1.29	−9.0	2	−9.3	2	−11.8	3	25	1.29	−10.1	2	−8.4	1	−9.6	2	−9.6	2	6	1.92	−8.8	1	−7.5	1	−6.7	0
3d	−10.9	3	−12.3	4	13	1.29	−11.3	2	−11.8	3	−10.9	3	20	1.29	−8.2	1	−9.1	2	−9.5	2	−13.7	8	8	1.89	−11.9	4	−12.3	3	−12.7	4
3e	−12.4	3	−13.8	6	11	1.12	−11.6	3	−12.6	4	−15.3	5	22	1.12	−12.6	3	−13.1	3	−11.9	2	−12.9	4	8	1.43	−10.7	2	−11.3	3	−9.6	2
3f	−7.9	2	−10.5	2	19	1.94	−8.3	1	−9.5	1	−12.2	3	21	1.94	−9.7	1	−10.1	1	−11.3	2	−10.1	3	8	1.75	−8.9	1	−9.1	2	−7.5	1
3g	−7.6	1	−9.8	2	13	2.05	−8.0	1	−7.8	1	−9.9	2	22	2.05	−7.1	1	−6.8	0	−8.2	1	−9.0	3	8	1.90	−8.4	2	−7.6	1	−8.1	1
3h	−8.2	1	−9.9	2	15	1.92	−8.3	1	−8.7	2	−10.4	2	13	1.92	−9.7	1	−8.3	1	−9.6	1	−9.4	3	5	1.36	−7.0	1	−8.4	1	−8.9	2
3i	−10.6	3	−11.3	4	13	1.81	−9.6	2	−10.2	3	−11.7	2	23	1.81	−10.2	1	−9.3	1	−8.0	0	−10.3	3	9	1.14	−9.0	1	−8.9	1	−9.4	2
3k	−9.8	2	−11.6	3	12	1.03	−8.9	1	−10.1	2	−12.7	5	14	1.03	−10.8	2	−11.0	3	−9.5	2	−9.7	2	7	1.35	−7.6	1	−8.0	1	−7.3	0
3l	−7.9	1	−10.9	2	15	1.70	−8.2	1	−9.0	1	−9.6	1	14	1.70	−7.2	0	−6.9	0	−8.1	1	−9.9	2	11	1.60	−8.1	1	−7.9	1	−8.7	1
3m	−7.7	0	−9.1	1	14	1.95	−7.3	0	−8.2	1	−10.3	3	21	1.95	−8.5	1	−9.0	2	−7.6	1	−9.3	1	9	1.34	−6.9	0	−7.8	1	−6.3	0
4	−7.5	1	−8.5	1	7	1.87	−6.7	0	−7.1	0	−10.2	3	14	1.87	−9.2	1	−8.1	1	−7.5	1	−11.2	4	6	1.23	−9.2	2	−8.1	1	−10.7	2
5	−10.2	1	−14.9	3	9	1.30	−11.3	2	−11.9	2	−15.9	4	13	1.30	−11.9	2	−12.7	2	−13.5	3	−13.4	4	7	1.37	−10.2	1	−8.9	1	−9.7	2
6a	−8.1	0	−9.7	1	14	1.19	−8.8	1	−7.9	0	−11.6	3	19	1.19	−9.2	2	−8.9	1	−10.2	2	−9.8	2	8	1.40	−7.3	1	−6.9	0	−7.0	0
6b	−9.3	1	−10.1	2	14	1.22	−8.9	1	−9.6	1	−10.6	2	20	1.22	−8.8	1	−7.9	1	−7.6	0	−11.5	4	9	1.85	−10.1	2	−10.9	2	−9.5	1
6c	−11.9	1	−13.2	2	14	1.12	−10.7	1	−11.3	2	−13.7	3	21	1.12	−11.3	2	−9.1	1	−10.6	2	−15.8	4	9	1.99	−12.1	2	−10.8	2	−10.2	1
6d	−10.8	2	−13.7	4	10	1.31	−11.4	2	−12.1	2	−12.5	4	20	1.31	−9.7	2	−10.5	2	−10.9	3	−10.7	3	5	1.70	−9.2	1	−8.9	1	−9.7	2
6e	−12.6	3	−15.2	4	11	0.31	−11.2	2	−12.1	2	−14.4	3	16	0.31	−10.8	1	−9.3	1	−8.1	1	−10.3	2	11	1.54	−8.9	1	−12.2	3	−9.5	1

a C: compound; E: docking score energy (kcal mol^−1^); H: hydrogen bonds; V: van der Waals interactions; and R: root-mean-square deviation (Å).

Regarding the investigated structure representing α-glucosidase, *viz.*, 3W37, the computational simulation is considered to be in good agreement with the experimental results collected from the bioassays. The docking-based computation predicted that the ligands with the highest inhibitability follow the order of 6e (DS −15.2 kcal mol^−1^) > 5 (DS −14.9 kcal mol^−1^) > 3e (DS −13.8 kcal mol^−1^) = 6d (DS −13.7 kcal mol^−1^) > 3b (DS −13.4 kcal mol^−1^) = 6c (DS −13.2 kcal mol^−1^). The significance is highly justified by the values calculated using a similar computing configuration for three commercialised drugs, *i.e.*, acarbose (DS −14.2 kcal mol^−1^), voglibose (DS −11.1 kcal mol^−1^), and miglitol (DS −12.7 kcal mol^−1^), which were reported in our previous study.^47^ The corresponding RMSD values for the derivatives are all under 2 Å, indicating a biologically rigid conformation.^48^ Especially, that of 6e is 0.31 Å, meaning a significantly short average distance between its (backbone) atoms, and thus it can possibly be viewed as a quasi-crystallised structure. All the compounds were determined to be effective α-glucosidase inhibitors based on the assay experiments, except for 3e, which exhibited a moderate IC_50_ of 36.60 ± 1.26 μM *in vitro* although it was predicted to have the third lowest DS energy *in silico*. Furthermore, if the intermolecular interaction ability is considered, this ligand can create 6 strong hydrogen bonds, storing a total Gibbs free energy of −10.3 kcal mol^−1^ (ESI: Section 2.1 and Table S1†), with the in-pose amino acids of its targeted protein structure and should be highly conducive to the stability of the 3e-3W37 complex. Although observed in-discrete, this experiment-theory inconsistency is still notable, thus requiring further explanation. Alternatively, the compounds showing unnoticeable inhibition towards the enzyme in the *in vitro* investigation, *viz.*, 3a, 3g, 3h, 3m, and 4, are also expected to form unstable ligand-protein complexes according to the static *in silico* study, given their significantly low DS values (over −10 kcal mol^−1^). This excludes 3d, whose IC_50_ value is over 390 μM (*i.e.*, no activity) but its corresponding DS value is −12.3 kcal mol^−1^ (*i.e.*, considerable stability). In addition, 6d was experimentally demonstrated to be a weak α-glucosidase inhibitor (IC_50_ of 228.09 ± 4.62 μM) but computationally predicted to exhibit good affinity towards the 3W37 structure (DS of −13.7 kcal mol^−1^). Thus, these discrepancies also require appropriate explanations. Otherwise, the docking-based static models correspond well with the assay-based inhibition experiments.

However, due to the information lost from the pre-docking conditions, we performed a QSARIS-based analysis on the physicochemical properties of the studied ligands with reference to Lipinski's rule of five. The results are summarised in Table 3.

**Table 3:** QSARIS-based physicochemical properties of the studied ligands (1, 2, 3a–3m, 4, 5, and 6a–6e) docked with the protein structures (3W37, 3AJ7 and PTP1B)

Ligand (compound)	DS_average_ (kcal mol^−1^)	Mass (amu)	Polarizability (Å^3^)	Dispersion coefficients		Hydrogen bond (3W37/3AJ7/PTP1B)	
				Log *P*	Log *S*	H-Acceptor	H-Donor
1	−10.5	442.8	33.7	6.2	−5.6	1/1/1	1/1/0
2	−12.9	458.0	38.7	4.2	−5.4	2/1/3	2/1/0
3a	−9.5	577.9	29.6	9.0	−9.2	1/1/1	1/2/1
3b	−12.4	589.0	36.8	4.7	−5.3	2/1/0	0/1/1
3c	−10.7	562.3	39.2	3.3	−5.1	1/1/2	1/1/0
3d	−12.3	827.9	30.1	11.6	−12.1	3/2/5	0/1/0
3e	−14.0	598.4	36.9	4.1	−5.2	2/1/2	1/1/0
3f	−10.9	597.9	41.0	3.7	−5.0	1/0/3	0/3/0
3g	−9.6	598.7	32.8	9.1	−9.6	1/1/3	1/1/0
3h	−9.9	687.4	31.7	9.8	−10.2	1/0/2	1/1/1
3i	−11.1	578.3	32.8	5.3	−6.6	4/0/3	0/2/0
3k	−11.3	499.5	33.5	4.9	−5.4	2/2/1	1/2/1
3l	−10.1	542.8	34.8	4.6	−5.3	1/0/2	1/0/0
3m	−9.6	528.6	31.1	8.6	−8.4	1/2/0	0/1/0
4	−10.0	416.8	29.8	6.4	−6.6	1/2/3	0/0/1
5	−14.7	416.2	52.4	3.1	−3.8	3/2/4	0/0/0
6a	−10.4	458.9	33.3	4.5	−5.7	1/1/2	0/0/0
6b	−10.7	501.2	37.2	3.9	−4.8	2/1/3	0/1/1
6c	−14.2	490.1	48.8	3.6	−3.7	1/3/3	1/0/0
6d	−12.3	537.8	37.3	5.1	−5.7	2/3/2	1/1/1
6e	−13.9	549.2	51.6	3.8	−3.5	3/1/1	0/0/1

The first criterion regarding molecular mass (<500 amu) can be used to explain the docking-assay inconsistency. The corresponding values for ligands 3d and 3e are 827.9 and 598.4 amu, respectively. Their significant mass implies that even though they can exhibit inhibition towards the 3W37 structure when in the pose, their heavy mass is more likely to deter their transport from the medium of either the *in vitro* experiments or real physiological environment. A similar approach can explain the mild discrepancy observed in the case of 6d due to its slightly heavy mass of 537.8 amu, *cf.*, Lipinski's criterion. In contrast, although having a lower-bound mass weight (mass of 461.8 amu), 4 exhibited no inhibition activity (IC_50_ > 390 μM) in the bioassay-based experiments, which is likely because of its low in-pose stability (DS of −8.5 kcal mol^−1^). Otherwise, most of the assay-based inactive compounds, *viz.*, 3a, 3d, 3g, 3h, and 3m, also possess a molecular mass over 500 amu, thus justifying this correlation. However, although 6e is predicted by the static model in the docking simulation to be the most promising inhibitor (DS of −15.2 kcal mol^−1^) towards α-glucosidase, the experimental observations reveal that it is only third-ranked (IC_50_ of 7.31 ± 0.11 μM), after 5 (IC_50_ of 2.73 ± 0.05 μM) and 6c (IC_50_ of 4.62 ± 0.12 μM). In this case, the differences can be explained based on the slightly high mass of 6e, *i.e.* 549.2 amu, compromising its capability to form a highly stable 6e-3W37 inhibitory system, but not prohibitively. Besides molecular mass, according to Lipinski's criteria, a good membrane-permeable molecule should satisfy the requirements of no more than 5 groups for hydrogen bonds; no more than 10 groups receiving hydrogen bonds; and a value of log *P* of less than +5 (log *P* < 5). Hence, it is obvious that the three most promising candidates, *viz.*5, 6c, and 6e, are ready for further development in pharmaceutical applications, especially for oral administration. In addition, their polarisability constants are significant, *i.e.* 52.4 Å^3^ for 5, 48.8 Å^3^ for 6c, and Å^3^ 51.6 for 6e, likely inducing the formation of molecular dielectric moments.^49^ This property is thought to be highly conducive to both inhibitory effects towards a protein structure given that the polypeptide molecule is made of polarised amino acids and fluidity in hydrophilic environments of the body.

In summary, the molecular docking technique coupled with analysis of the QSARIS-based physicochemical properties can provide a reasonably accurate model to predict ligand-protein inhibitability in general, and dammarane-α-glucosidase inhibition in particular. The latter determines the mobility of the ligands, whereas the former provides the stability of the ligand–protein complexes. This theoretical model suggests that the *Dipterocarpus alatus*-derived compounds are promising drug-like inhibitors towards α-glucosidase, following the order of 5 (mass: 416.2 amu; polarisability: 52.4 Å^3^; DS: −14.9 kcal mol^−1^) > 6c (mass: 490.1 amu; polarisability: 48.8 Å^3^; DS: −13.7 kcal mol^−1^) > 6e (mass: 549.2 amu; polarisability: 51.6 Å^3^; DS: −15.2 kcal mol^−1^), which is in excellent agreement with the experiments. However, this model is still considered premature, thus requiring more solid-evidenced validations. Molecular dynamics simulation can monitor the simultaneous “behaviour” of all atoms in the systems, thus validating the mechanism of inhibition, and surface plasmon resonance can identify the correct ligand-protein complex structures.

Relatively, the inhibitability of the studied ligands towards the other diabetes-related protein structures, *i.e.*, 3AJ7 and PTP1B, was predicted without experimental evidence. Firstly, the former is still expected to be most vulnerable at its site 1 and with the strongest interactions derived by 5 (DS: −15.9 kcal mol^−1^), 6c (DS: −13.7 kcal mol^−1^), and 6e (DS: −14.4 kcal mol^−1^). Although the 3e-3AJ7 inhibitory system has favourable stability, given its DS value of −15.3 kcal mol^−1^, number of hydrogen bonds of 5, and number of van der Waals interactions of 22, its formation its expected to be significantly hindered due to the heavy mass (598.4 amu) and low polarisability (36.9 Å^3^) of the ligand. Similarly, the formation of the 2-PTP1B and 3d-PTP1B complexes, especially the latter, is almost unexpected despite the favourable stability of their intermolecular interaction. Besides, the dispersion coefficient (log *P*) of 3d is 11.6, which is much higher that the Lipinski's threshold, suggesting its insignificant dispersity. Meanwhile, 6e does not seem to be a promising inhibitor towards the PTP1B structure given its corresponding moderate DS values, regardless of the inhibited sites. Therefore, the preliminary analysis suggests that 5 and 6c are versatile inhibitors regarding all the diabetes-related protein structures, *i.e.*, 3W37, 3AJ7, and PTP1B. The theoretical implications can be verified by appropriate experimental investigations, *e.g.*, enzyme inhibition assays or clinical trials.

**Fig. 5:**
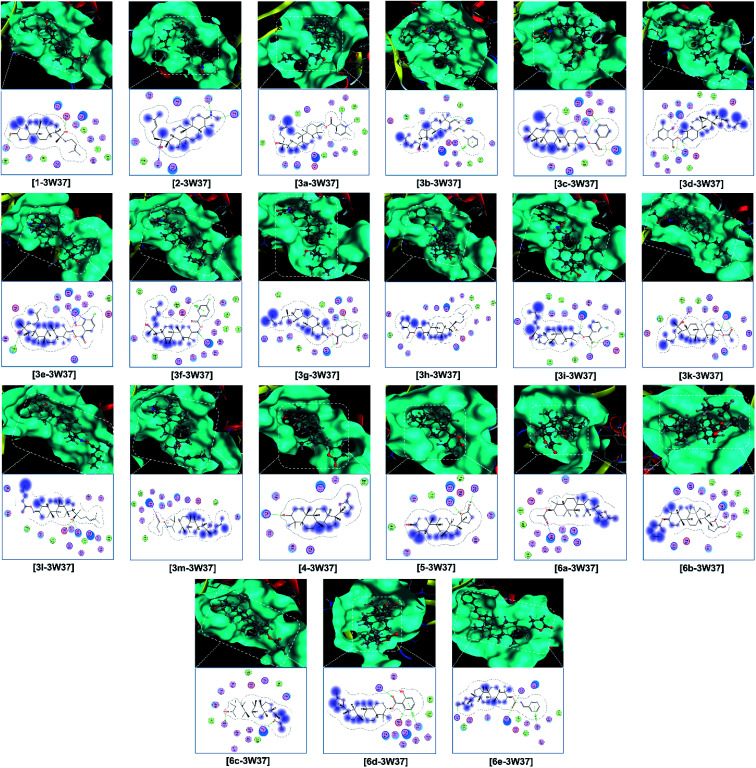
Visual presentation and in-pose interaction map of ligand–3W37 inhibitory complexes.

**Fig. 6:**
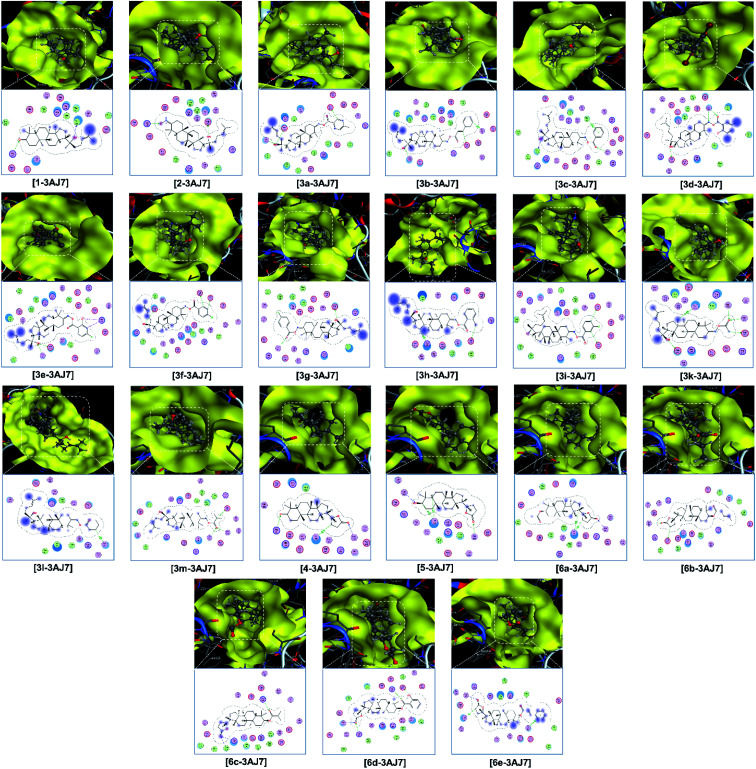
Visual presentation and in-pose interaction maps of the ligand–3AJ7 inhibitory complexes.

**Fig. 7:**
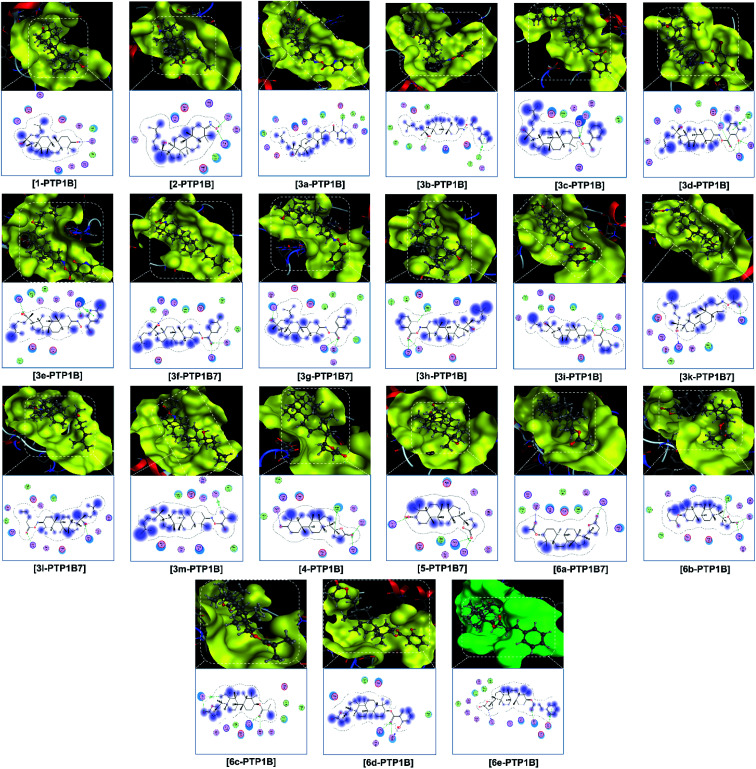
Visual presentation and in-pose interaction maps of the ligand–PTP1B7 inhibitory complexes.

Finally, the 3D morphology and 2D interaction map of all the ligand-protein inhibitory complexes are visually presented in Fig. 5–7. In the design of specific protein inhibitors, the size- and shape-compatibility between the ligands and the protein sites is also important. Although the *Dipterocarpus alatus*-derived compounds are projected to be size-fitting (from the 3D observation) and shape-complementary (by 2D continuous proximity contours) with the in-pose topographical features of the diabetes-related protein structures, the sites are unlikely to be conducive to either the entry of significantly larger candidates or simultaneous inhibition. Therefore, a size- and medical-reference for the further development of pharmaceutical medicine are also given. In addition, the configurations of the in-pose interactions were projected in detail.

## 4. Conclusions

This study demonstrated the capability of *Dipterocarpus alatus*-based derivatives as effective inhibitors towards α-glucosidase and proposed a new strategy to predict the ligand-protein inhibitability. Dipterocarpol (1) was isolated from *Dipterocarpus alatus* and 20 derivatives (2, 3a–m, 4, 5, and 6a–e) were obtained from semi-synthesis. Except for 2, the synthetic derivatives are new compounds. According to the experimental assay-based research, the most effective inhibitors towards α-glucosidase follow the order of 5 (IC_50_ of 2.73 ± 0.05 μM) > 6c (IC_50_ of 4.62 ± 0.12 μM) > 6e (IC_50_ of 7.31 ± 0.11 μM). Considering the computational docking-QSARIS analysis, the corresponding order is 5 (mass: 416.2 amu; polarisability: 52.4 Å^3^; DS: −14.9 kcal mol^−1^) > 6c (mass: 490.1 amu; polarisability: 48.8 Å^3^; DS: −13.7 kcal mol^−1^) > 6e (mass: 549.2 amu; polarisability: 51.6 Å^3^; DS −15.2 kcal mol^−1^). These results indicate that the enzyme inhibition activity (observed from experiments) of a ligand highly correlates with its physicochemical properties (calculated by QSARIS and referenced to Lipinski's rule of five) and the ligand–protein static stability (simulated by molecular docking technique). The extended simulations predicted 5 and 6c to be versatile inhibitors regarding all the diabetes-related protein structures, *viz.*3W37, 3AJ7, and PTP1B. These results can encourage further investigations to develop alternative anti-diabetic drugs, and the prediction framework of ligand–protein inhibitability may attract more work for validation, *e.g.*, surface plasmon resonance and molecular dynamics simulation.

## Conflicts of interest

There are no conflicts to declare.

## Supplementary Material

RA-011-D1RA04461C-s001
