# Supplementary material for: Newly synthesised oxime and lactone derivatives from Dipterocarpus alatus dipterocarpol as anti-diabetic inhibitors: experimental bioassay-based evidence and theoretical computation-based prediction
Source: RSC Adv. 2021 Nov 4;11(57):35765–82. doi: 10.1039/d1ra04461c (PMC9043233; doi:10.1039/d1ra04461c)
Supplement: RA-011-D1RA04461C-s001 [file RA-011-D1RA04461C-s001.pdf]

Electronic Supplementary Material (ESI) for RSC Advances.
This journal is © The Royal Society of Chemistry 2021

**SUPPORTING INFORMATION**

# New-synthesised oxime and lactone derivatives from *Dipterocarpus alatus* dipterocarpol as anti-diabetic inhibitors: experimental bioassay-based evidence and theoretical computation-based prediction

**Tran Thi Phuong Thao[1,2§], Thanh Q. Bui[3§], Nguyen Thi Thanh Hai[3], Lam K. Huynh[4], Phan Tu Quy[5], Nguyen Chi Bao[6], Nguyen Thi Dung[1], Nguyen Linh Chi[1,2], Tran Van Loc[1,2], Irina E. Smirnova[7], Anastasiya V. Petrova[7], Pham Thi Ninh[1,2], Tran Van Sung[1,2]*, Nguyen Thi Ai Nhung[3]***

[1]*Institute of Chemistry, Vietnam Academy of Science and Technology (VAST), 18 Hoang Quoc Viet Road, Cau Giay, Ha Noi, Vietnam.*
[2]*Graduate University of Science and Technology, VAST, 18 Hoang Quoc Viet Road, Cau Giay, Ha Noi, Vietnam.*
[3]*Department of Chemistry, University of Sciences, Hue University, Hue City, Vietnam.*
[4]*International University, Quarter 6, Linh Trung Ward, Thu Duc District, Ho Chi Minh City, Vietnam.*
[5]*Department of Natural Sciences & Technology, Tay Nguyen University, Buon Ma Thuot, Vietnam.*
[6]*Hue University, Hue City, Vietnam.*
[7]*Ufa Institute of Chemistry-Subdivision of the Ufa Federal Research Centre of the Russian Academy of Sciences, Ufa, prospekt Oktyabrya 71, Russian Federation.*

[§]These authors contributed equally to this work.

*Correspondence to:
Tran Van Sung (E-mail: tranvansungvhh@gmail.com)
Nguyen Thi Ai Nhung (E-mail: ntanhung@hueuni.edu.vn)

| Section | Content | Page |
|---|---|---|
| **1** | **EXPERIMENTAL CHARACTERISATION** | 3 |
| 1.1 | Compound 1 | 3 |
| 1.2 | Compound 2 | 11 |
| 1.3 | Compound 3a | 20 |
| 1.4 | Compound 3b | 27 |
| 1.5 | Compound 3c | 35 |
| 1.6 | Compound 3d | 43 |
| 1.7 | Compound 3e | 53 |
| 1.8 | Compound 3f | 61 |
| 1.9 | Compound 3g | 68 |
| 1.10 | Compound 3h | 76 |
| 1.11 | Compound 3i | 83 |
| 1.12 | Compound 3k | 90 |
| 1.13 | Compound 3l | 97 |
| 1.14 | Compound 3m | 104 |
| 1.15 | Compound 4 | 112 |
| 1.16 | Compound 5 | 137 |
| 1.17 | Compound 6a | 145 |
| 1.18 | Compound 6b | 154 |
| 1.19 | Compound 6c | 165 |
| 1.20 | Compound 6d | 175 |
| 1.21 | Compound 6e | 185 |
| **2** | **COMPUTATIONAL SIMULATION** | 194 |
| 2.1 | In-detail data of ligand-3W37 inhibitory complexes | 194 |
| 2.2 | In-detail data of ligand-3AJ7 inhibitory complexes | 197 |
| 2.3 | In-detail data of ligand-PTP1B inhibitory complexes | 200 |
| **3** | **DOSE RESPONSE CURVE OF THE MOST POTENT COMPOUNDS** | 203 |
| 3.1 | Dose response curve of compound 5 | 203 |
| 3.2 | Dose response curve of compound 6c | 204 |
| 3.3 | Dose response curve of compound 6e | 205 |

## 1. EXPERIMENTAL CHARACTERISATION

### 1.1. Compound 1

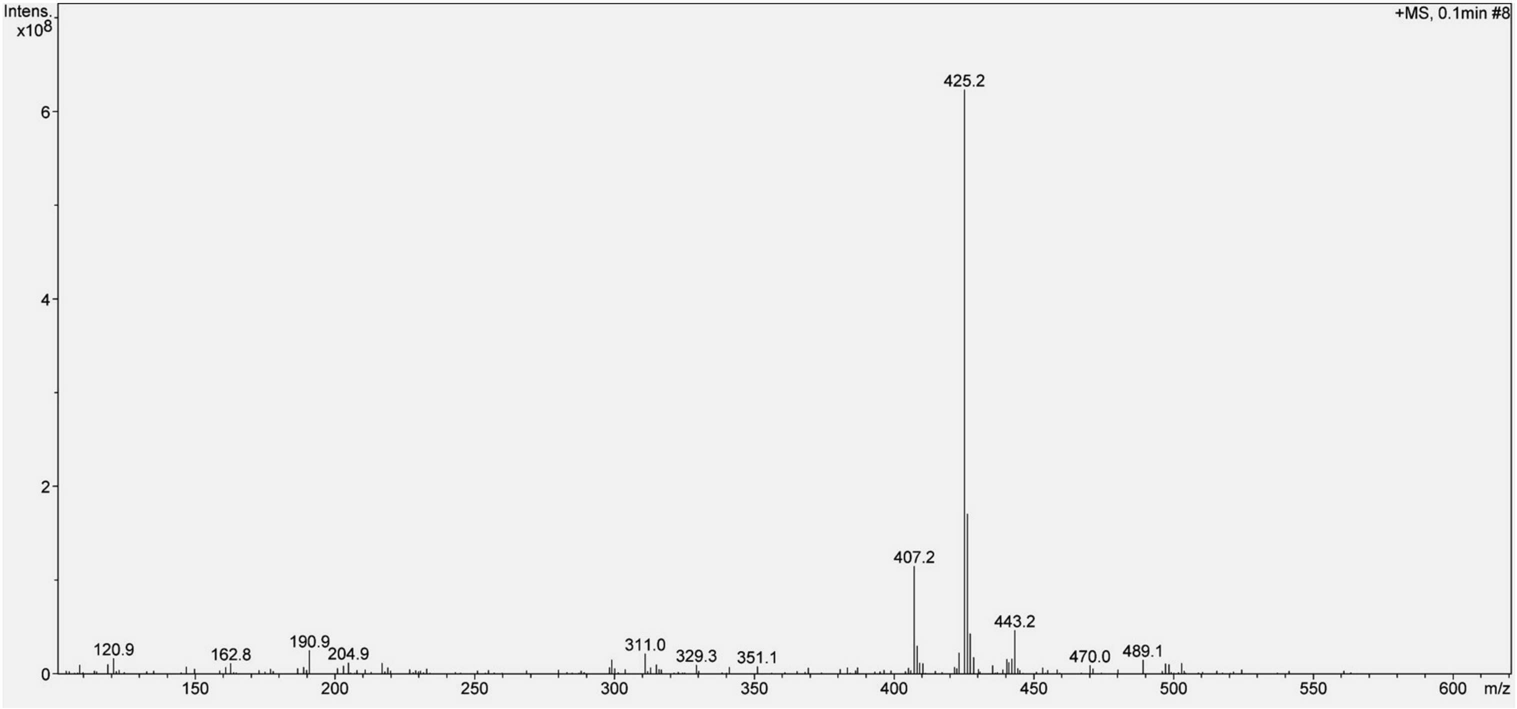


(+)-ESI–MS spectrum of compound **1**

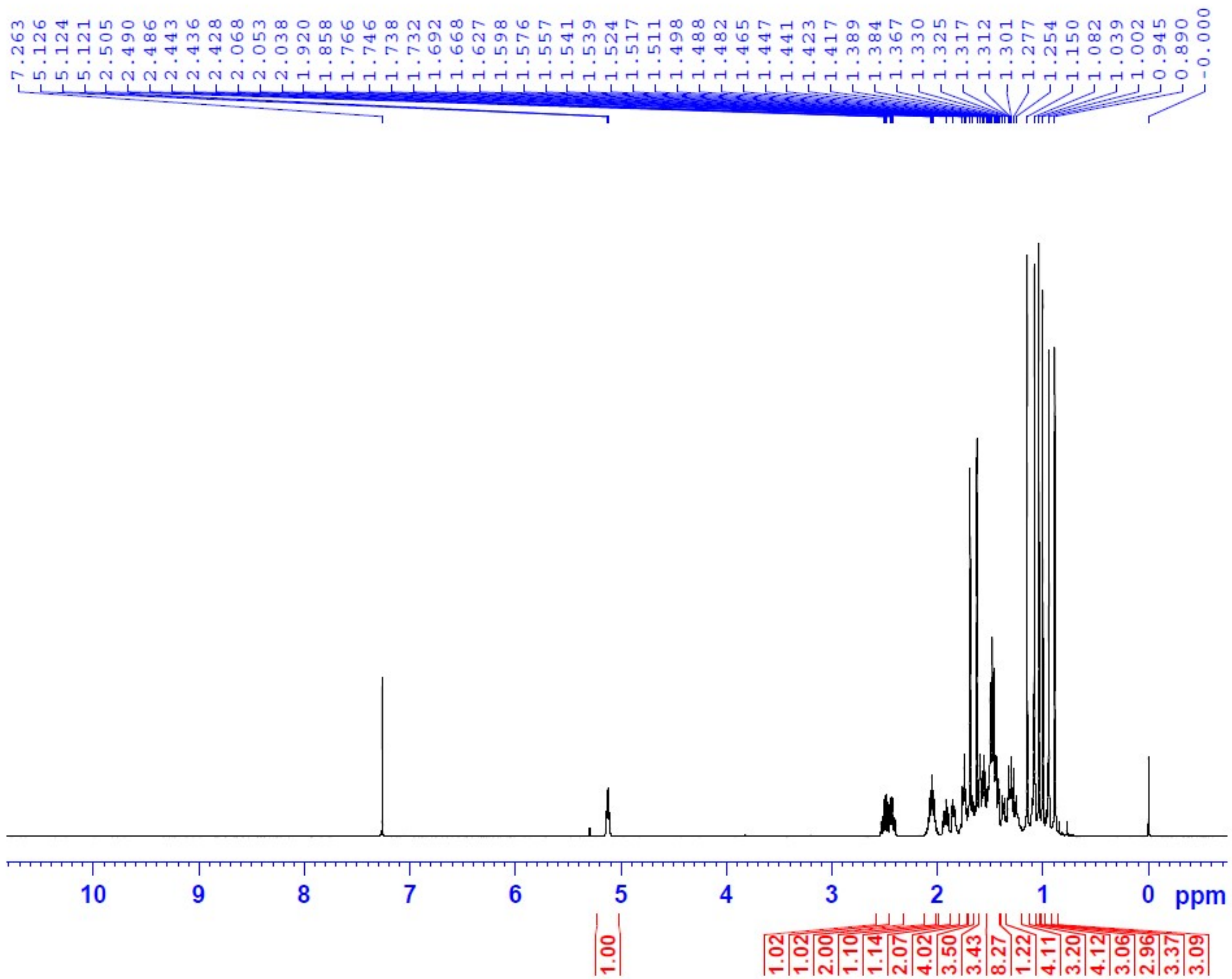


$^{1}$H-NMR spectrum of compound **1**

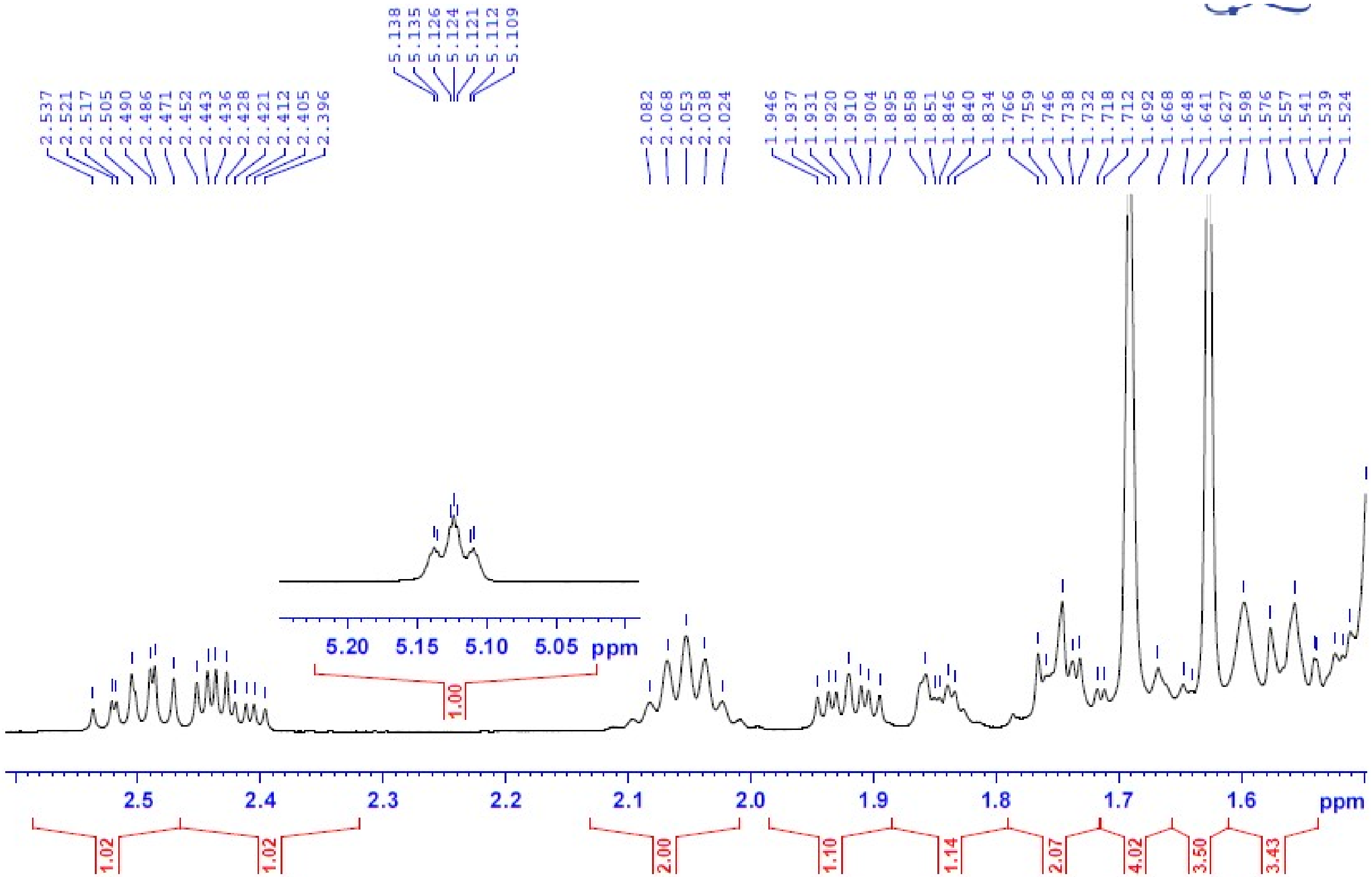
2.537 2.521 2.517 2.505 2.490 2.486 2.471 2.452 2.443 2.436 2.428 2.421 2.412 2.405 2.396
5.138 5.135 5.126 5.124 5.121 5.112 5.109
2.082 2.068 2.053 2.038 2.024
1.946 1.937 1.931 1.920 1.910 1.904 1.895 1.858 1.851 1.846 1.840 1.834 1.766 1.759 1.746 1.738 1.732 1.718 1.712 1.692 1.668 1.648 1.641 1.627 1.598 1.576 1.557 1.541 1.539 1.524
5.20 5.15 5.10 5.05 ppm
1.00
2.5 2.4 2.3 2.2 2.1 2.0 1.9 1.8 1.7 1.6 ppm
1.02 1.02 2.00 1.10 1.14 2.07 4.02 3.50 3.43

$^{1}$H-NMR spectrum of compound **1** (extension)

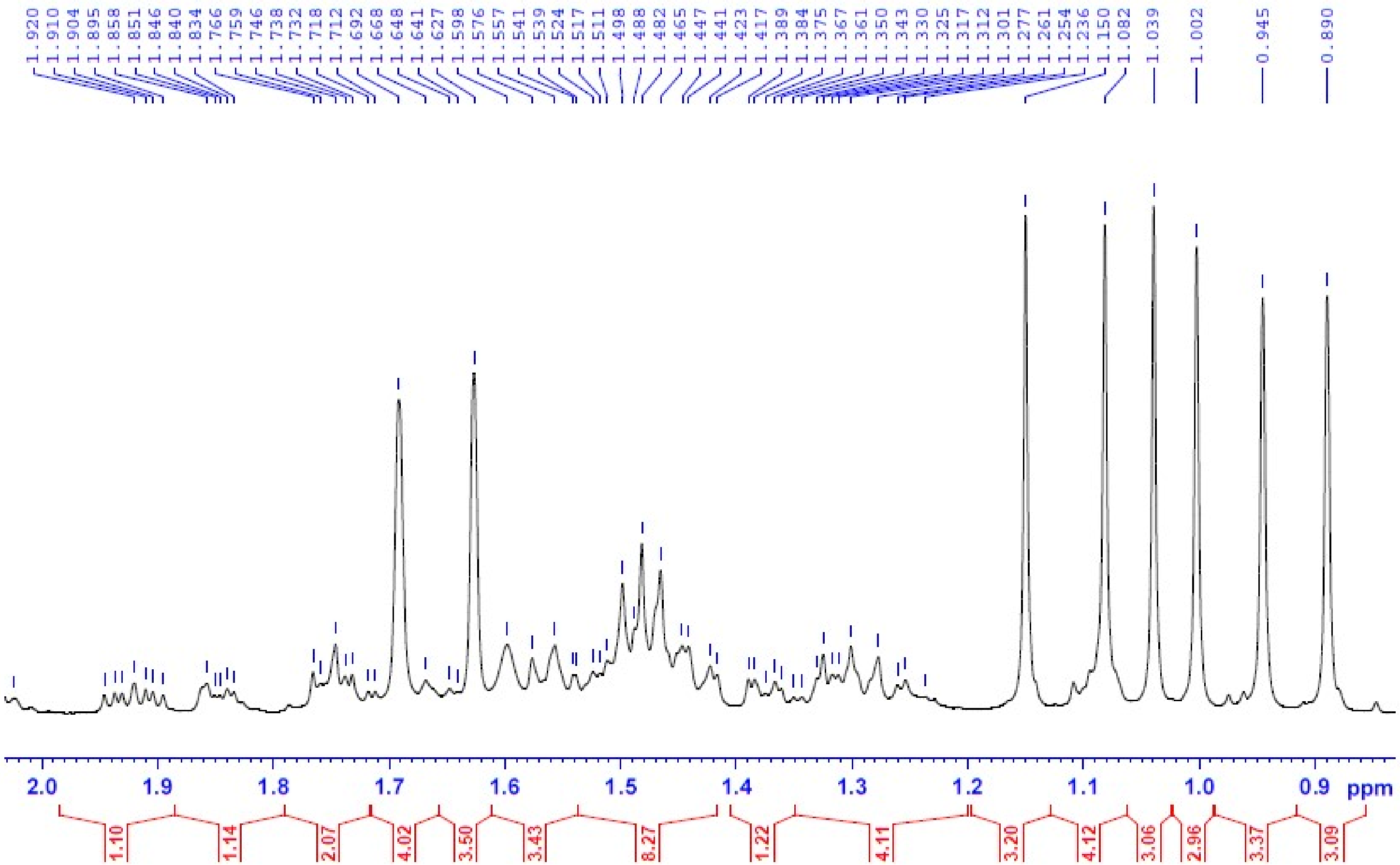

1.920
1.910
1.904
1.895
1.858
1.851
1.846
1.840
1.834
1.766
1.759
1.746
1.738
1.732
1.718
1.712
1.692
1.668
1.648
1.641
1.627
1.598
1.576
1.557
1.541
1.539
1.524
1.517
1.511
1.498
1.488
1.482
1.465
1.447
1.441
1.423
1.417
1.389
1.384
1.375
1.367
1.361
1.350
1.343
1.330
1.325
1.317
1.312
1.301
1.277
1.261
1.254
1.236
1.150
1.082
1.039
1.002
0.945
0.890
2.0
1.9
1.8
1.7
1.6
1.5
1.4
1.3
1.2
1.1
1.0
0.9
ppm
1.10
1.14
2.07
4.02
3.50
3.43
8.27
1.22
4.11
3.20
4.12
3.06
2.96
3.37
3.09

$^{1}$H-NMR spectrum of compound **1** (extension)

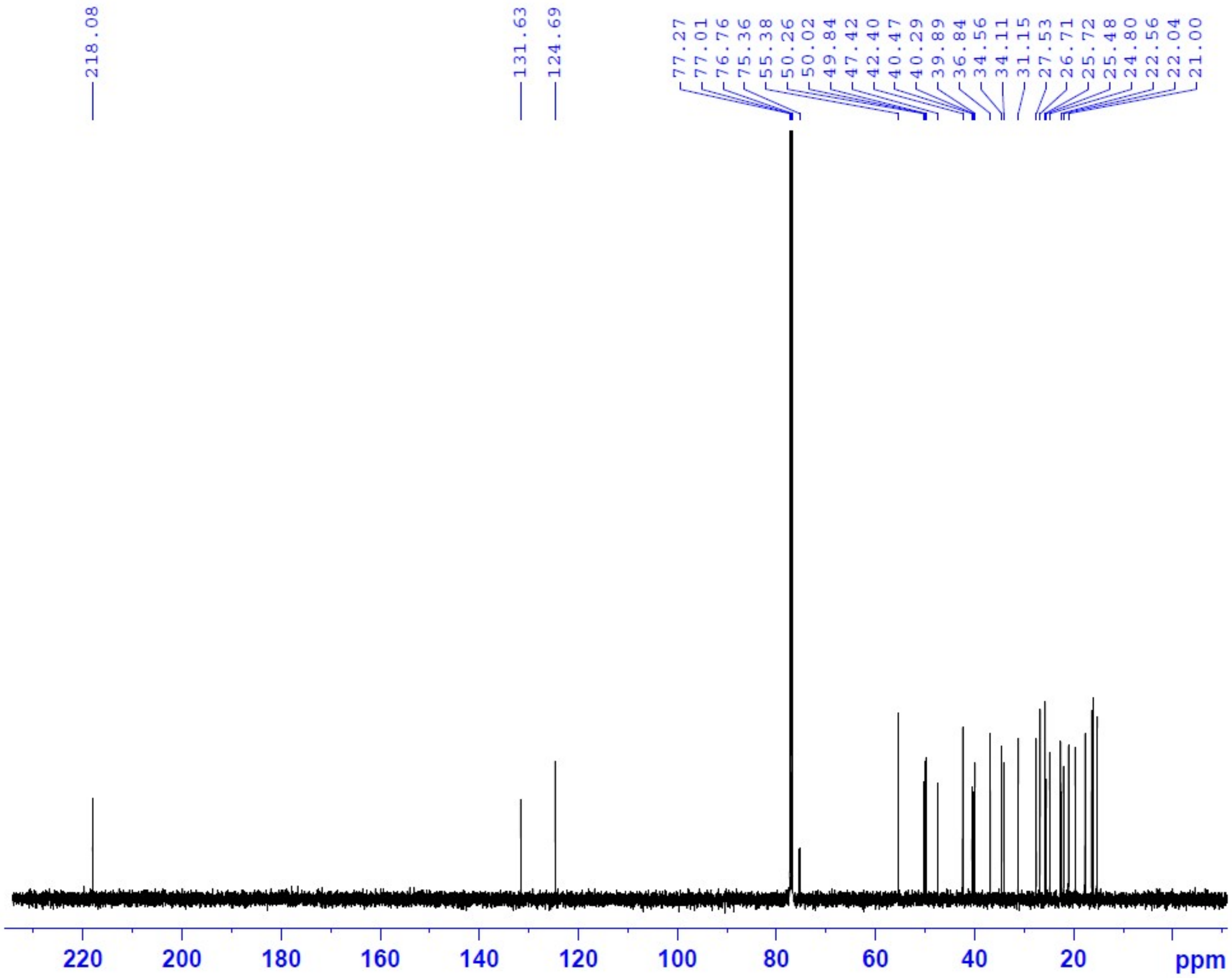


$^{13}$C-NMR spectrum of compound **1**

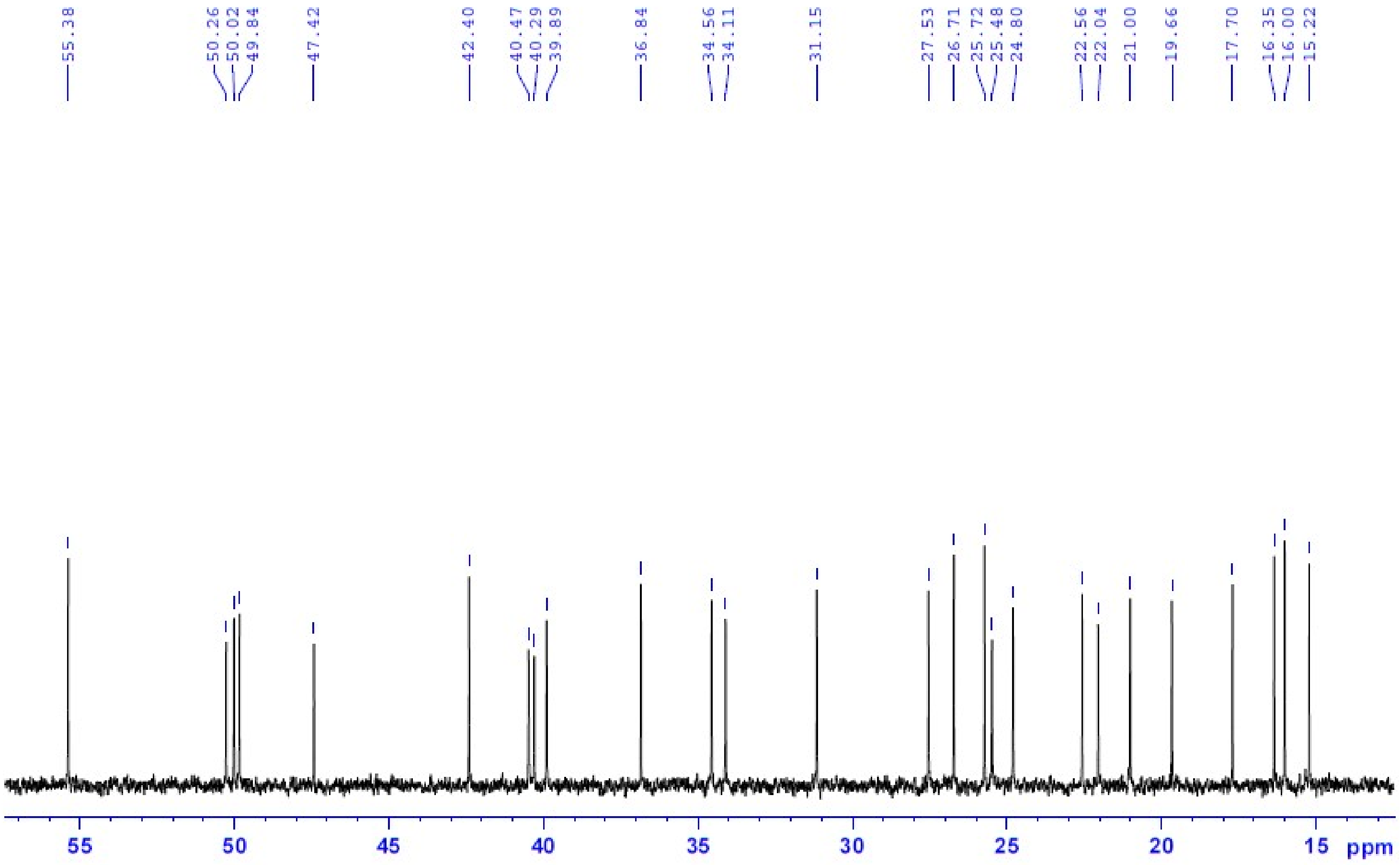
55.38
50.26
50.02
49.84
47.42
42.40
40.47
40.29
39.89
36.84
34.56
34.11
31.15
27.53
26.71
25.72
25.48
24.80
22.56
22.04
21.00
19.66
17.70
16.35
16.00
15.22
55
50
45
40
35
30
25
20
15
ppm

$^{13}$C-NMR spectrum of compound **1** (extension)

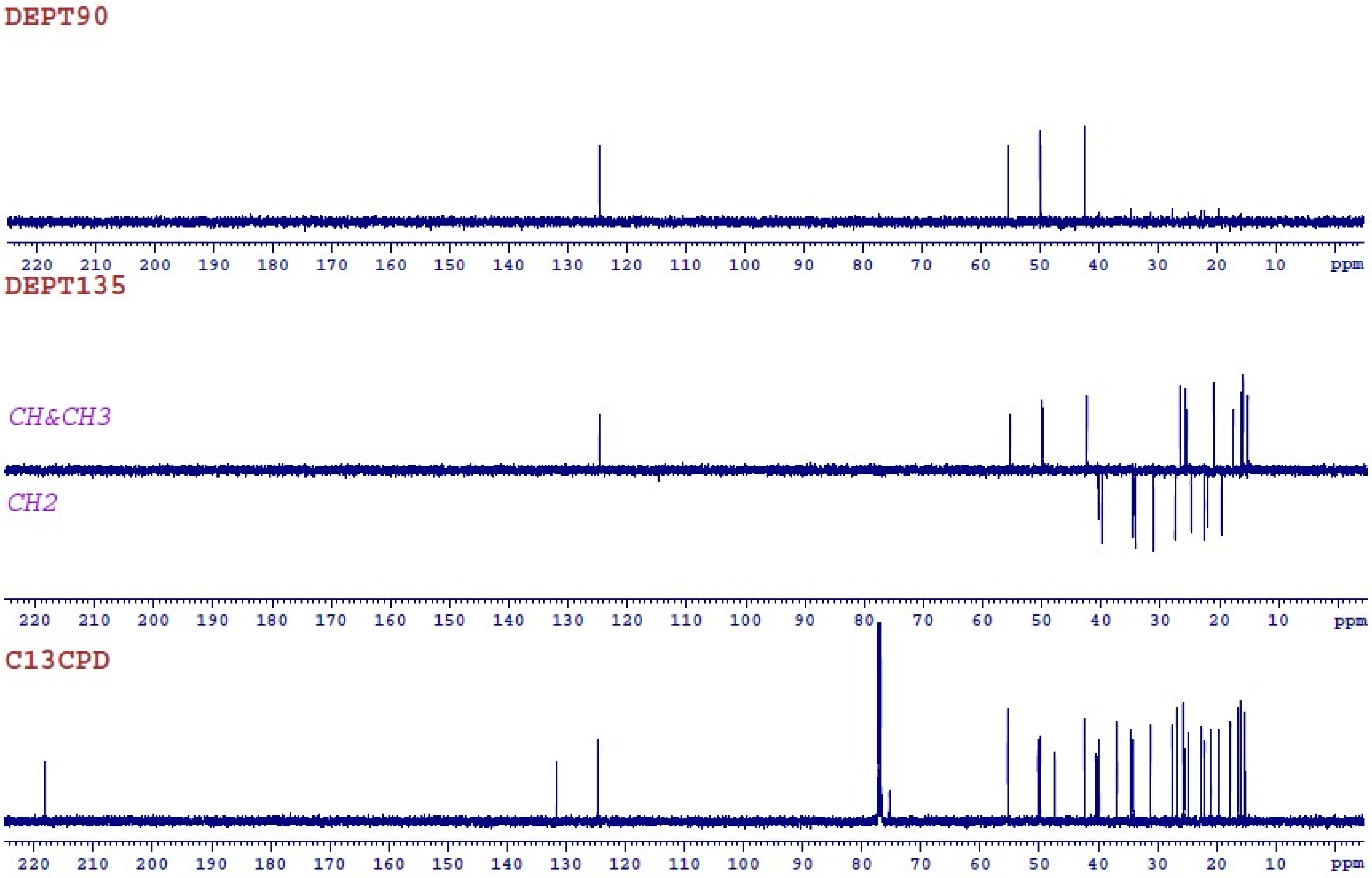


DEPT spectrum of compound **1**

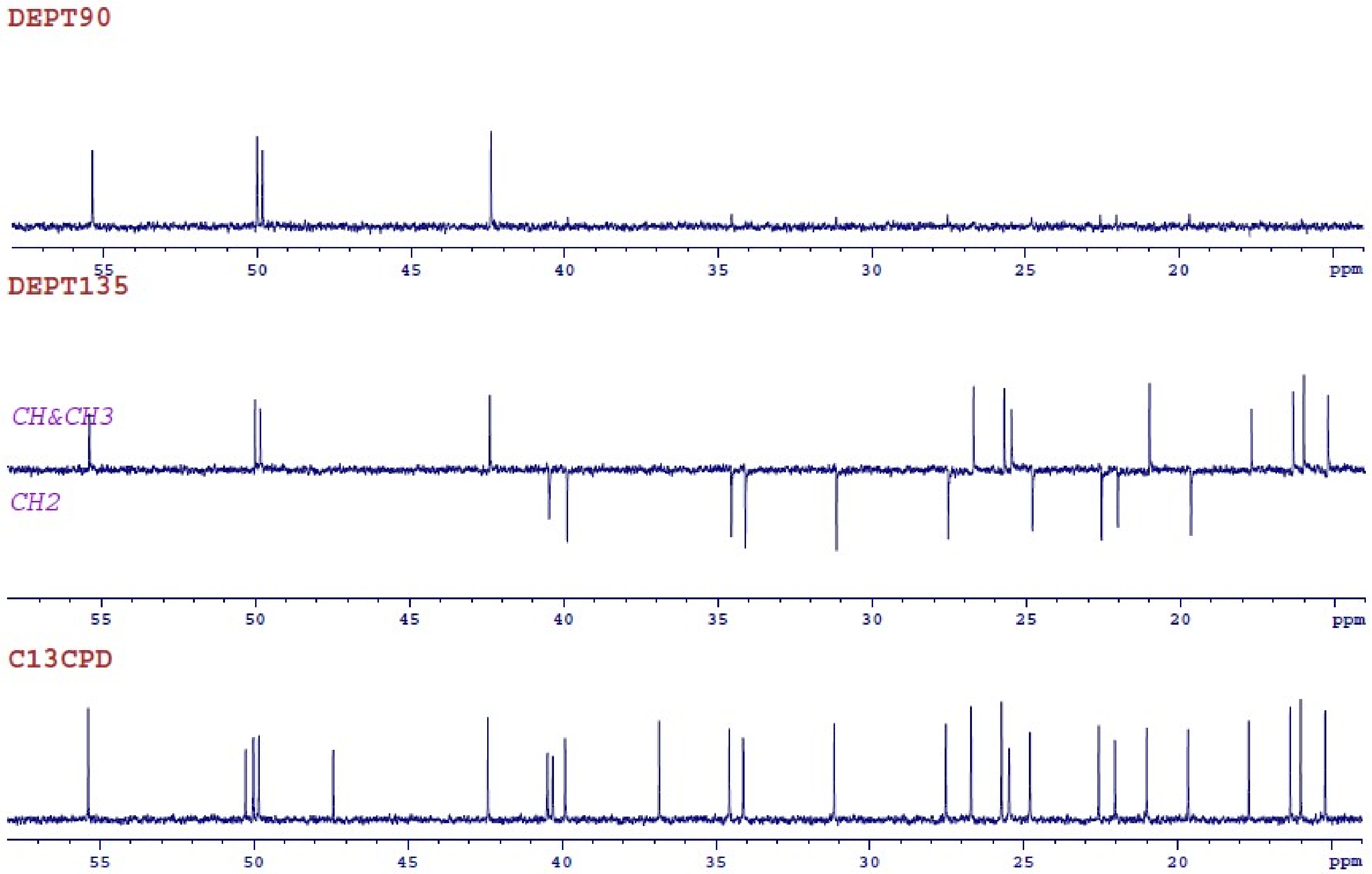


DEPT spectrum of compound **1** (extension)

**1.2. Compound 2**

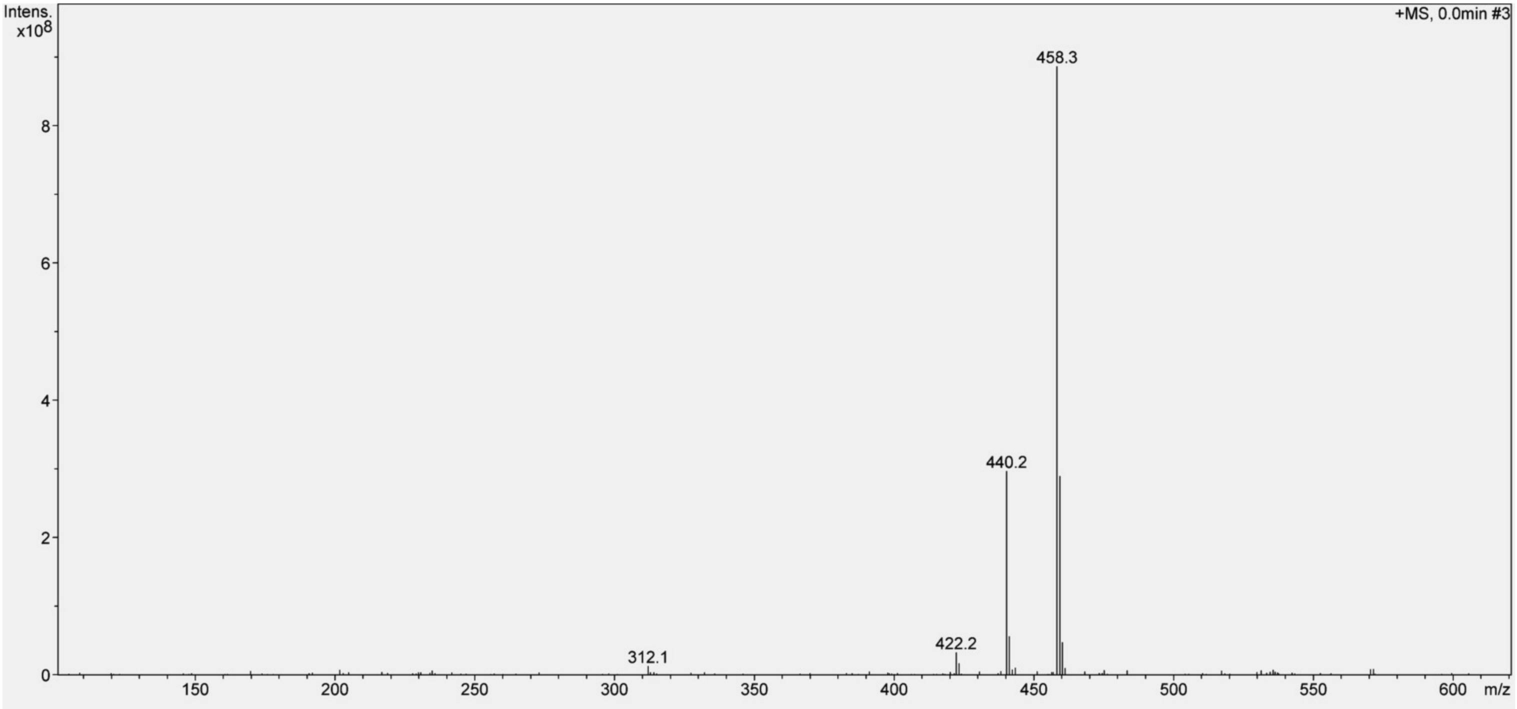


(+)-ESI–MS spectrum of compound **2**

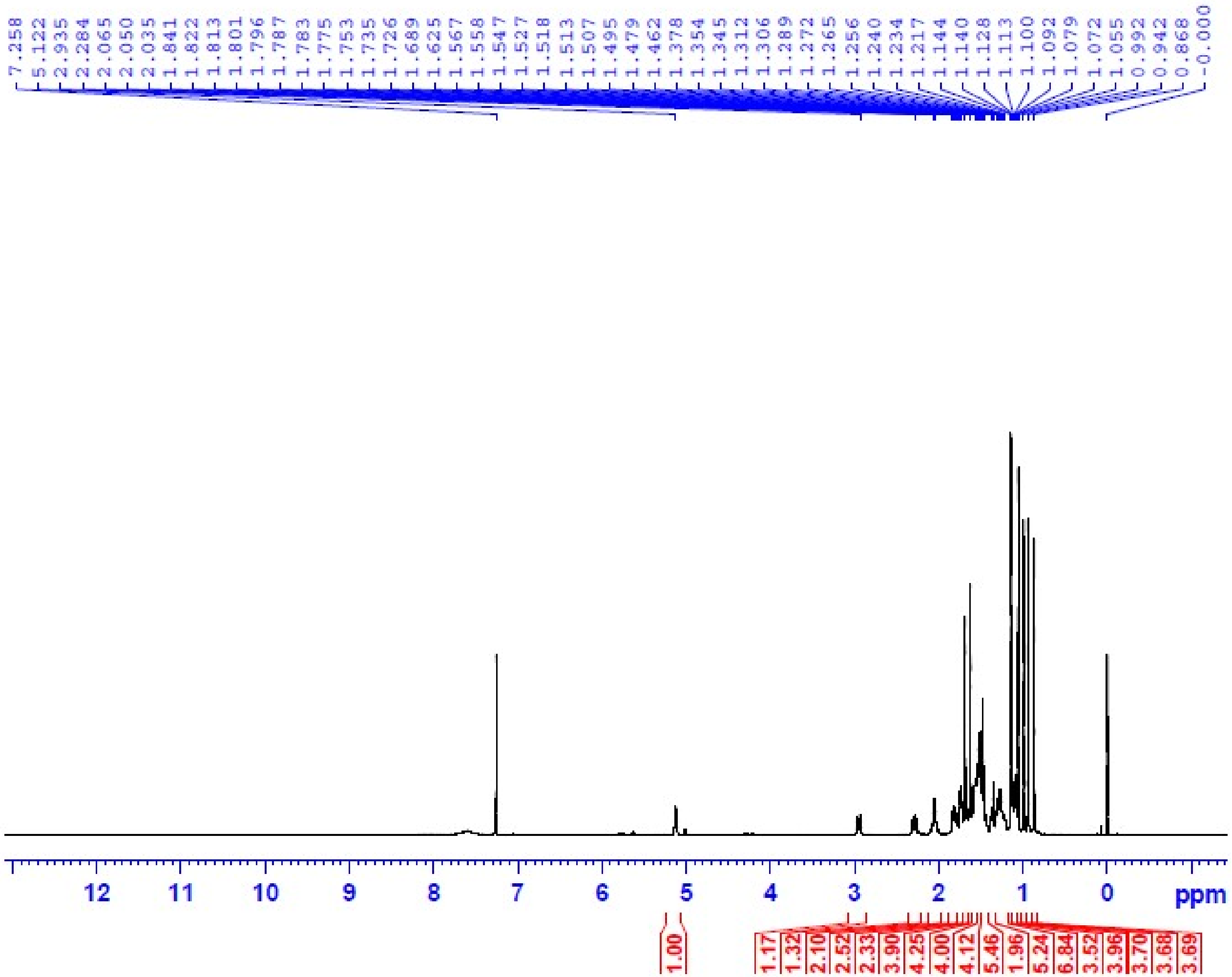


$^{1}$H-NMR spectrum of compound **2**

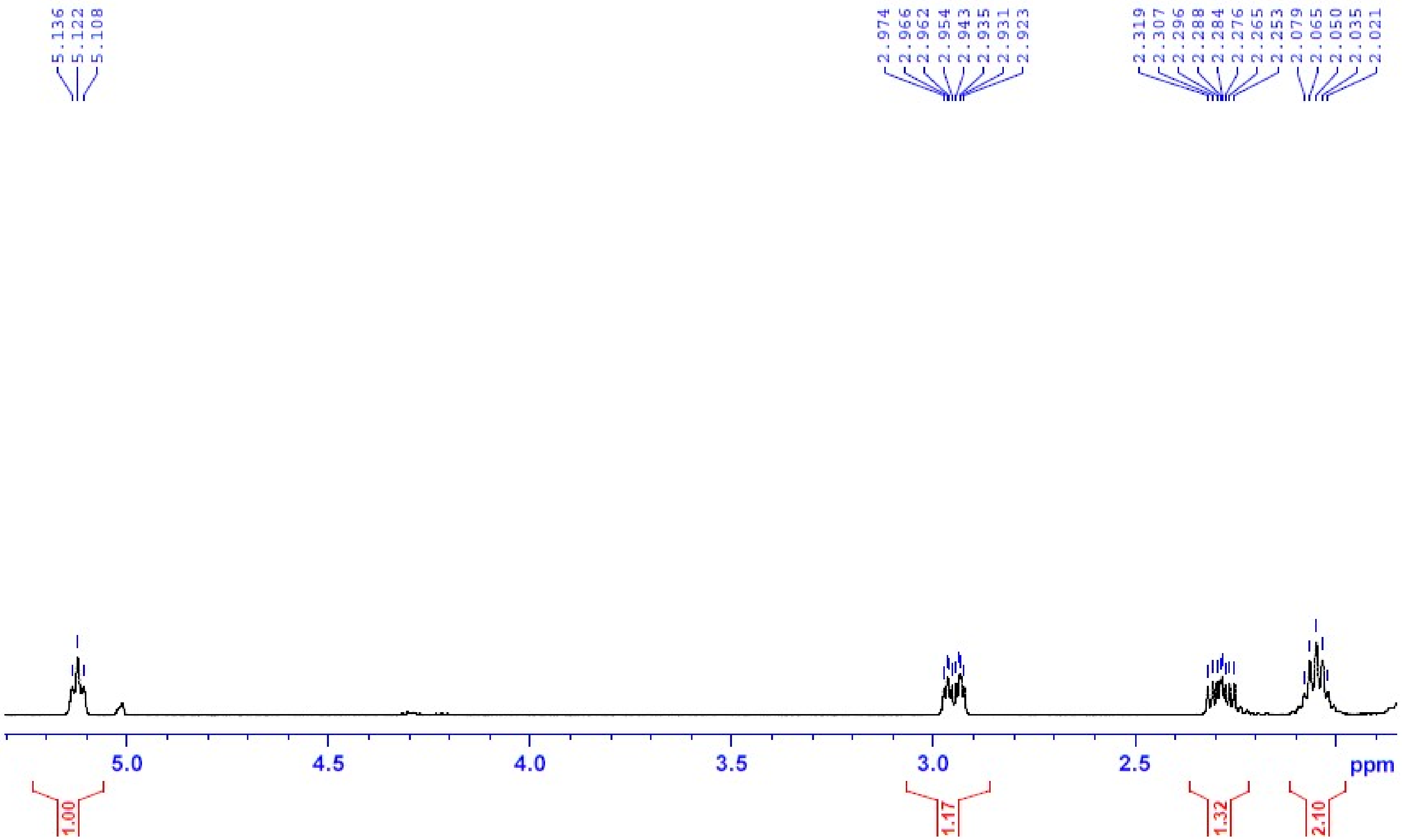

5.136
5.122
5.108
2.974
2.966
2.962
2.954
2.943
2.935
2.931
2.923
2.319
2.307
2.296
2.288
2.284
2.276
2.265
2.253
2.079
2.065
2.050
2.035
2.021
5.0
4.5
4.0
3.5
3.0
2.5
ppm
1.00
1.17
1.32
2.10

$^{1}$H-NMR spectrum of compound **2** (extension)

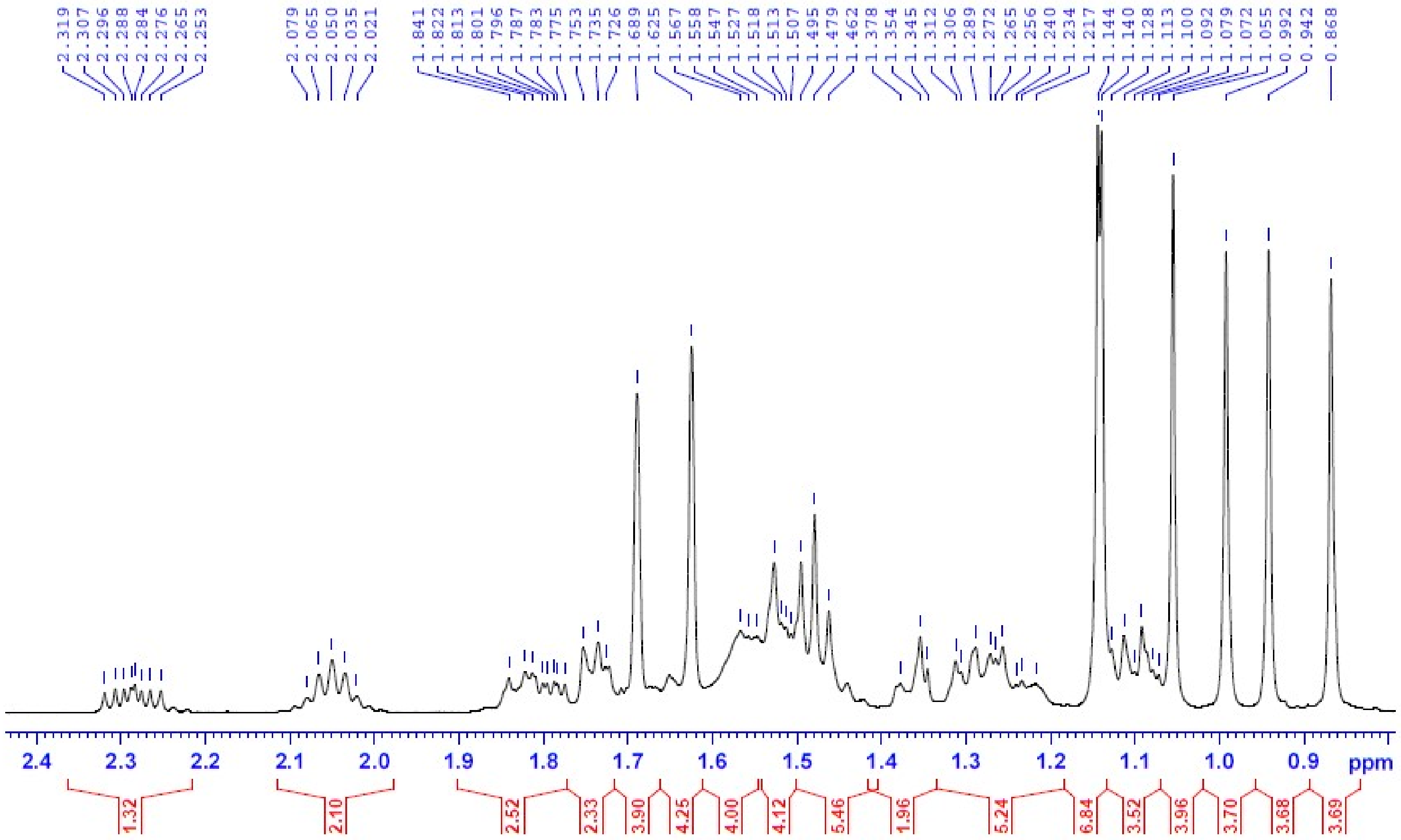

$^1$H-NMR spectrum of compound **2** (extension)

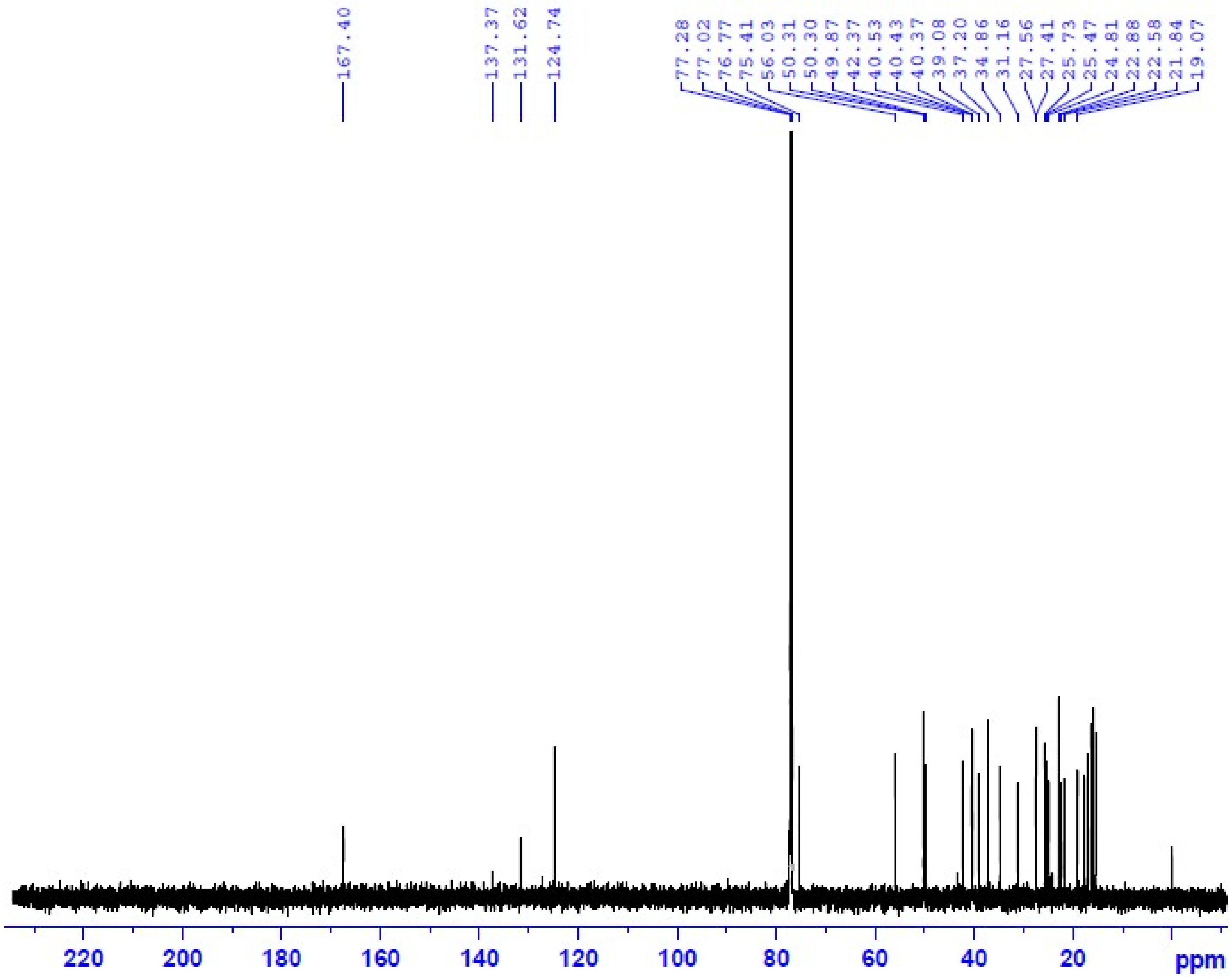


$^{13}$C-NMR spectrum of compound **2**

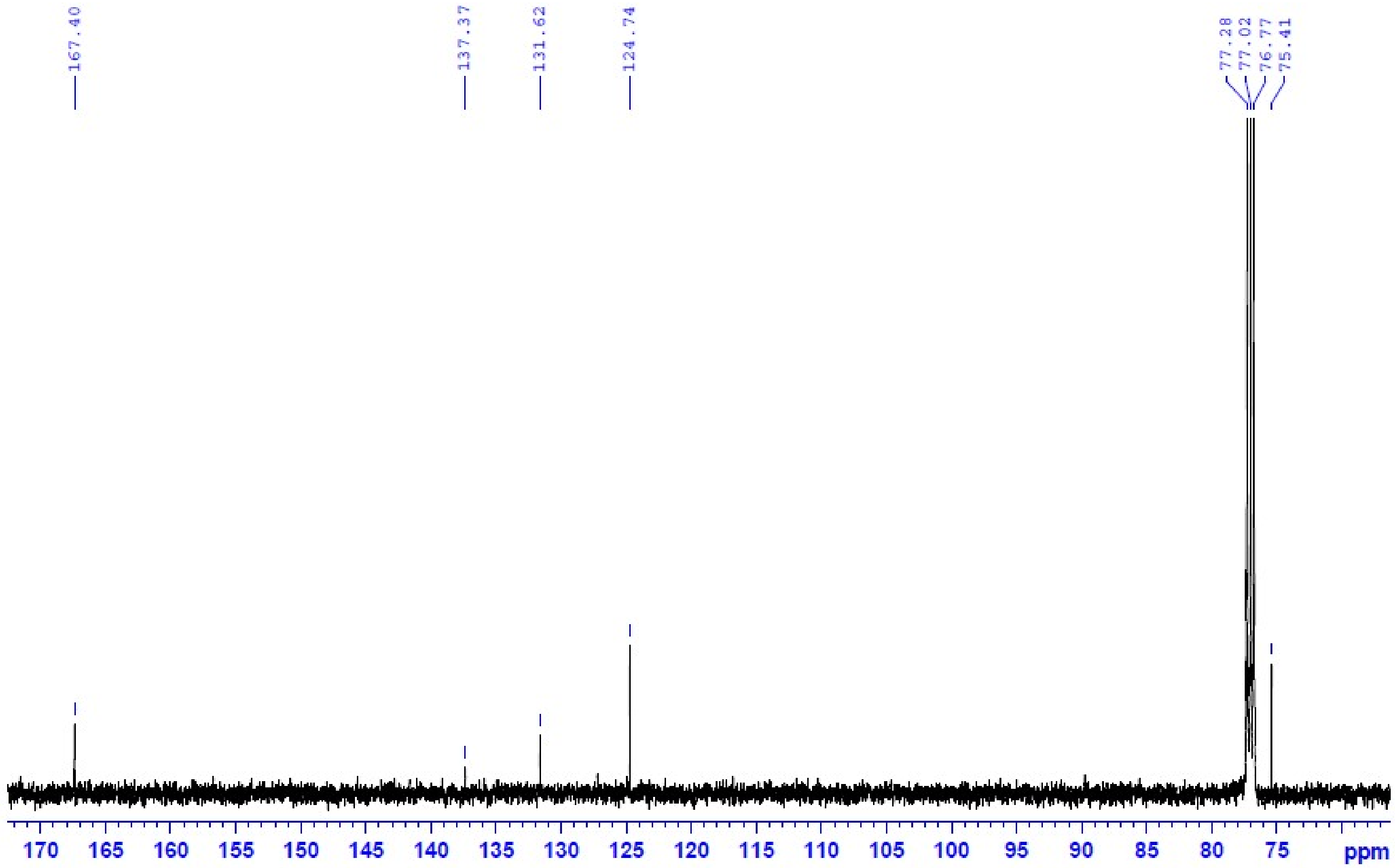


$^{13}C$-NMR spectrum of compound **2** (extension)

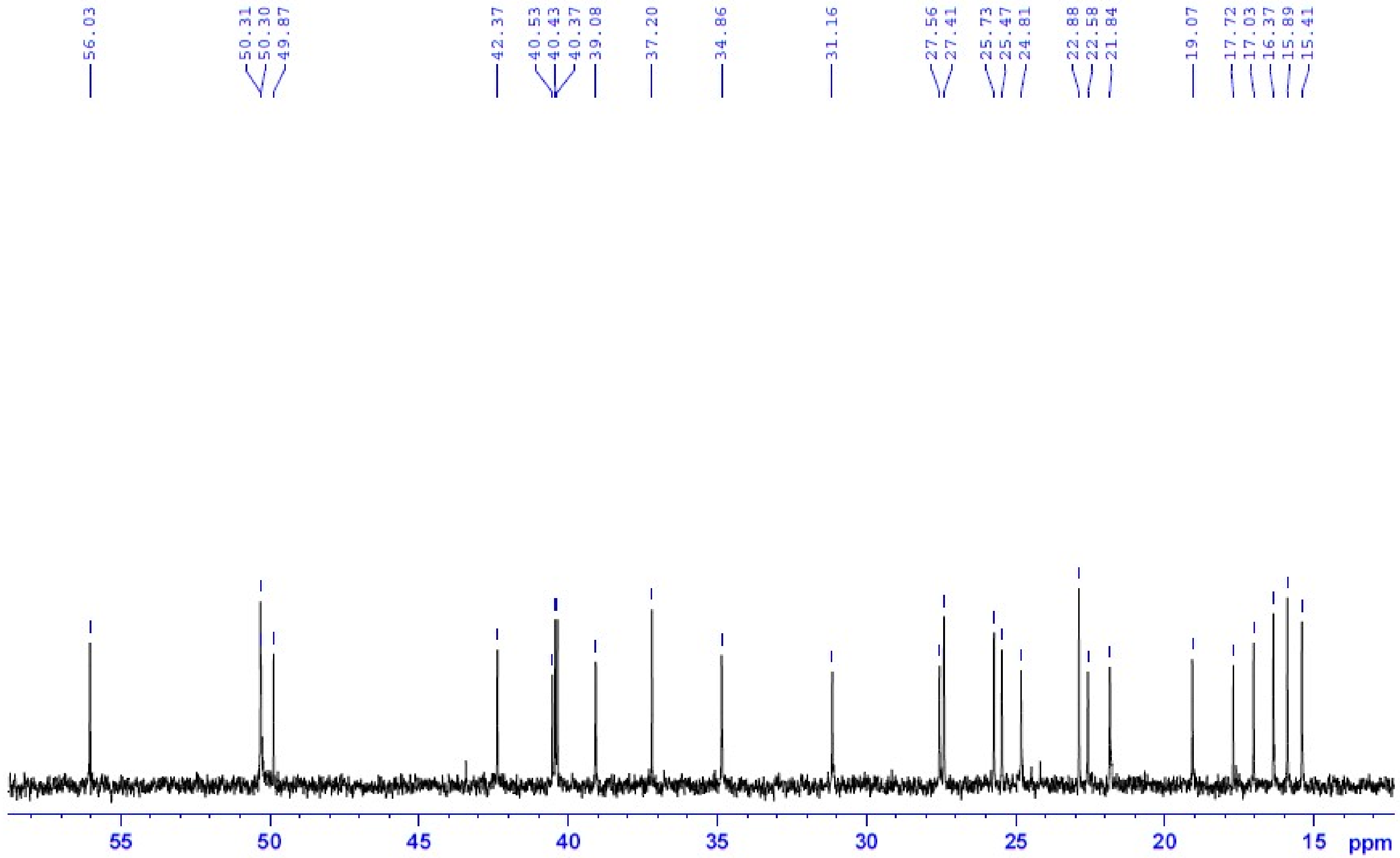
56.03
50.31
50.30
49.87
42.37
40.53
40.43
40.37
39.08
37.20
34.86
31.16
27.56
27.41
25.73
25.47
24.81
22.88
22.58
21.84
19.07
17.72
17.03
16.37
15.89
15.41
55
50
45
40
35
30
25
20
15
ppm

$^{13}$C-NMR spectrum of compound **2** (extension)

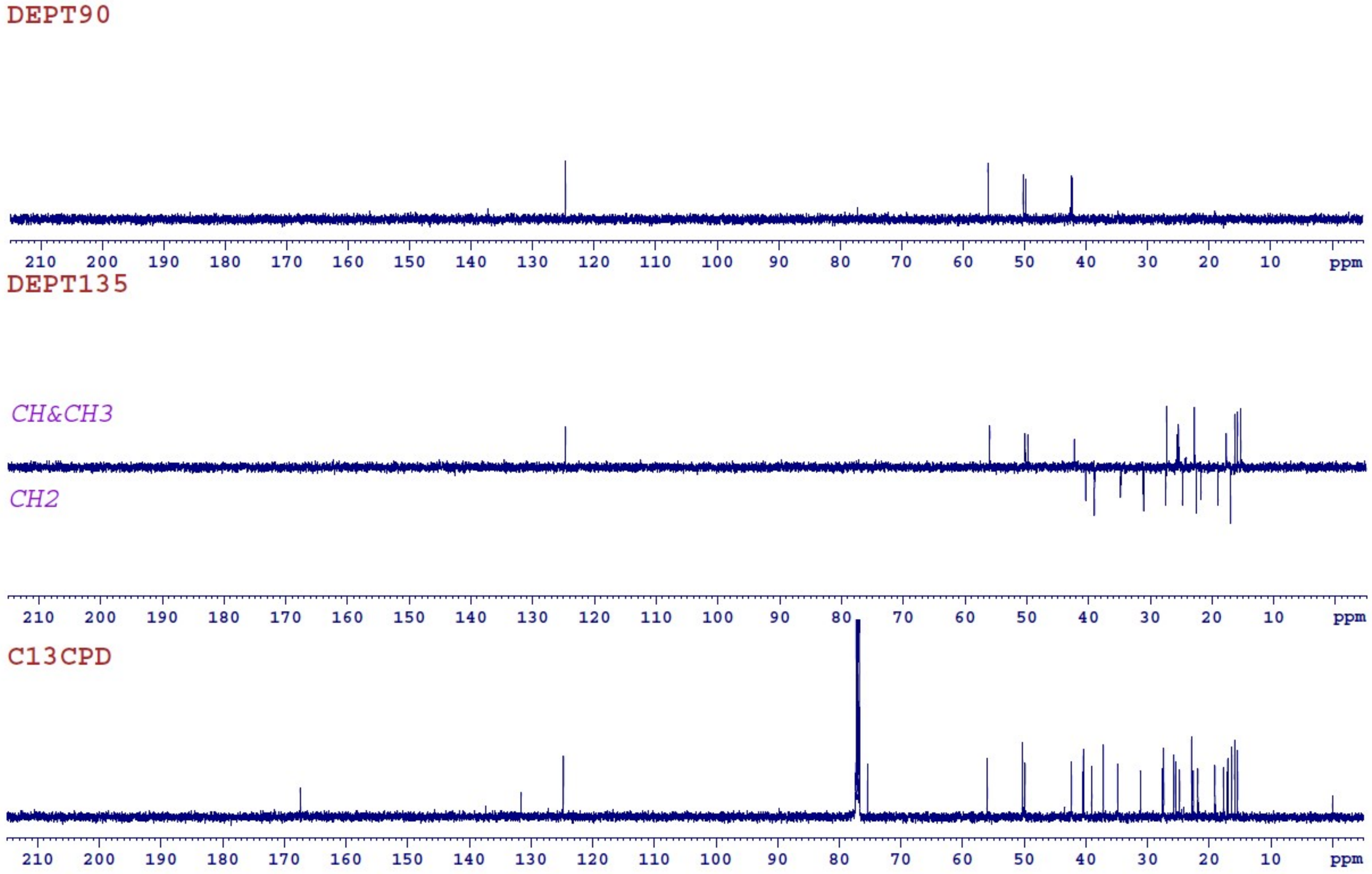


DEPT spectrum of compound **2**

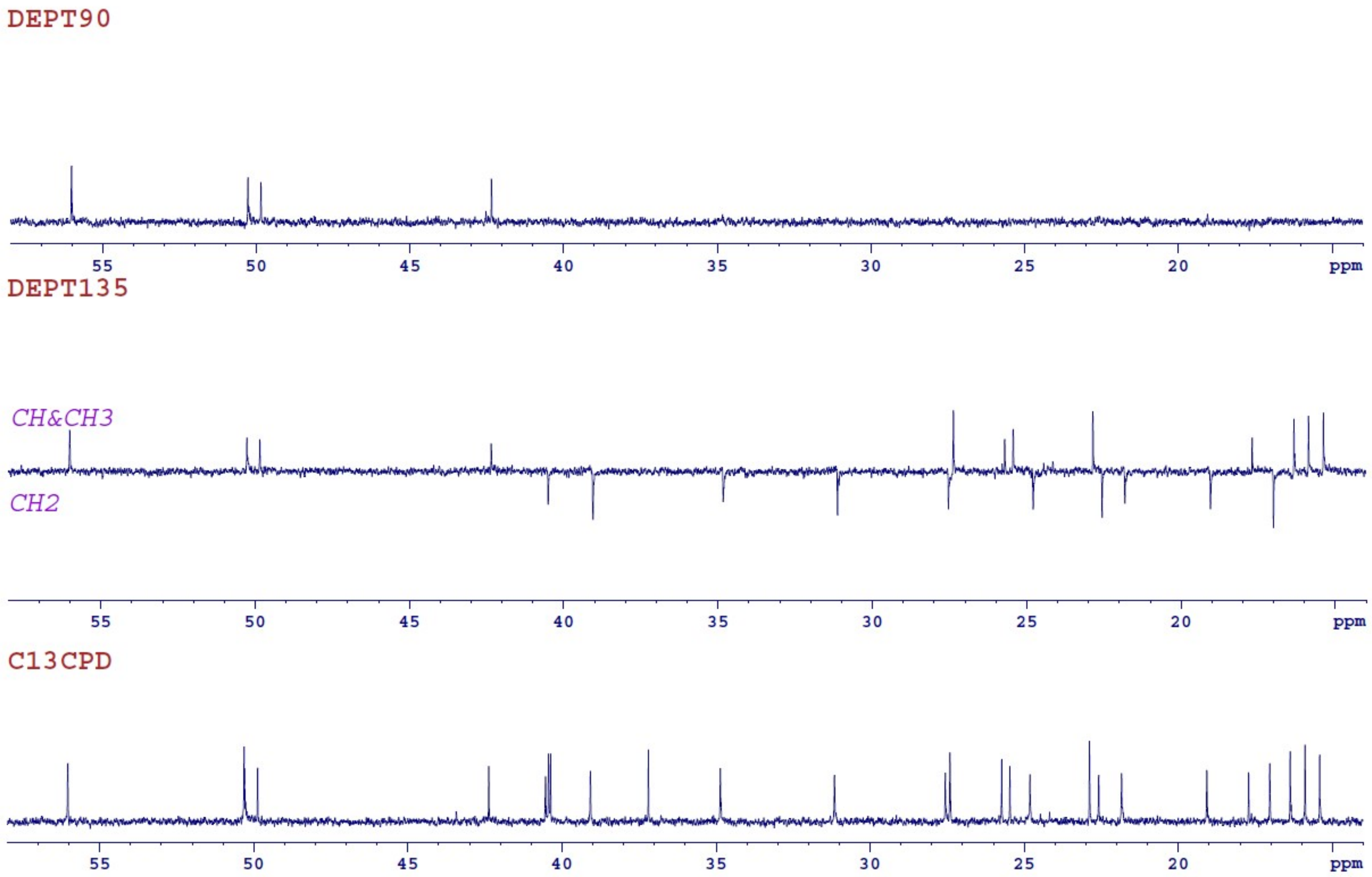


DEPT spectrum of compound **2** (extension)

## 1.3. Compound 3a

***Sample name: DNPyrazin***
***Operator: Le Anh VHH***
***Method: +IDA TOF MS/MS***
***Date: 2021.04.23***

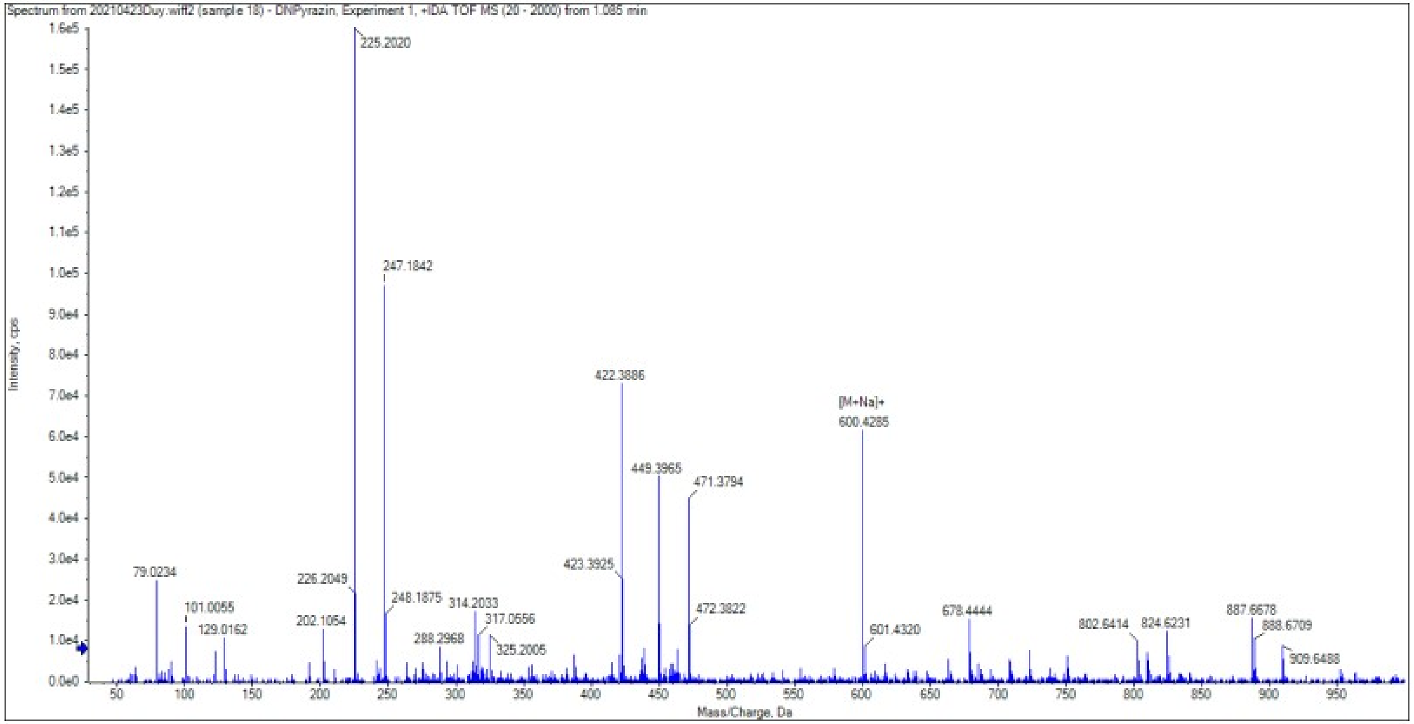


| Hit | Formula | m/z | RDB | ppm | MS Rank | MSMS ppm | MSMS Rank | Found |
|---|---|---|---|---|---|---|---|---|
| 1 | C36H55N3O3 | 600.42456 | 11.0 | 4.5 | 1 | | | NA/NA |

(+)-HR-ESI-MS spectrum of compound **3a**

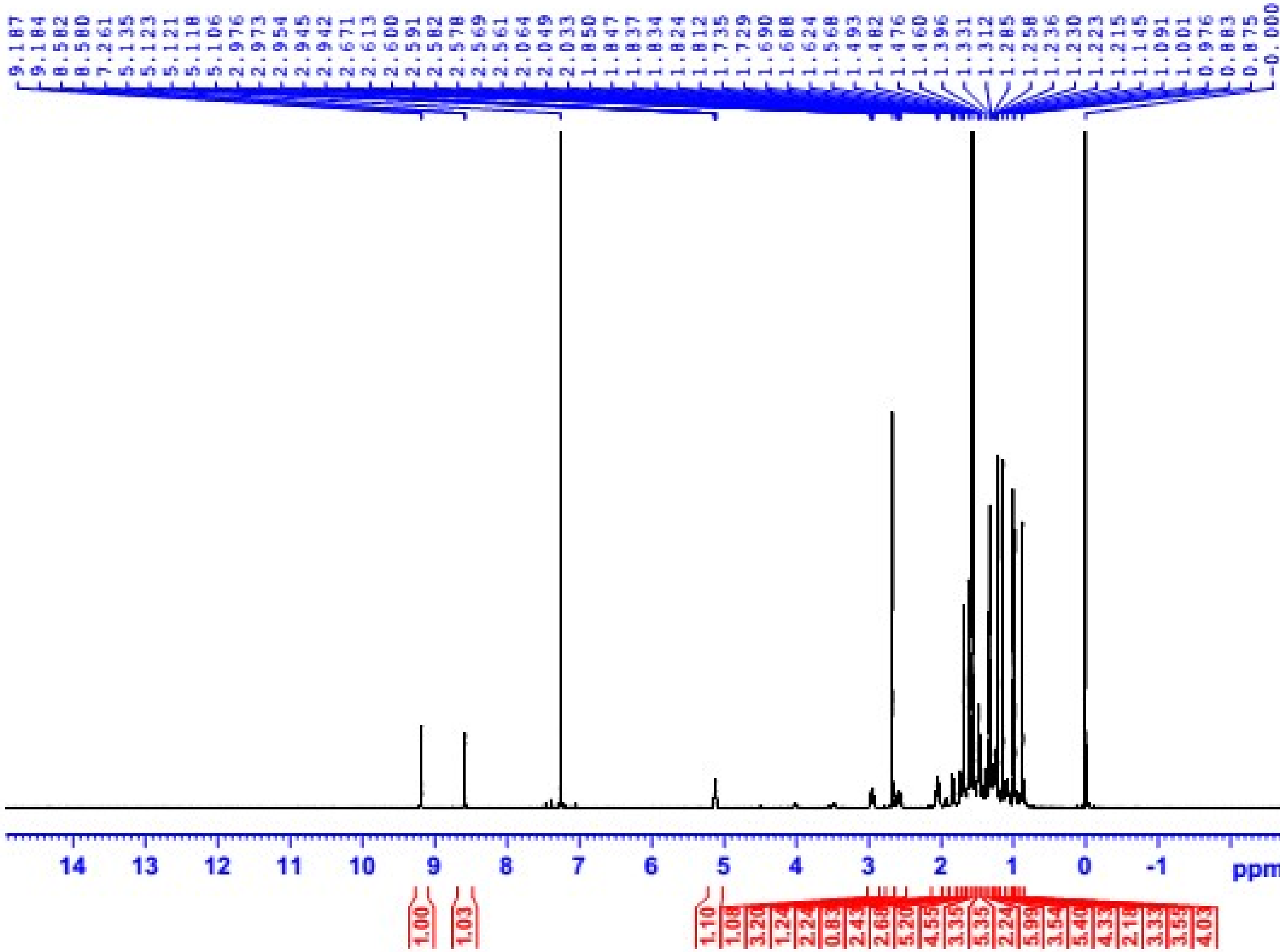

$^{1}$H-NMR spectrum of compound **3a**

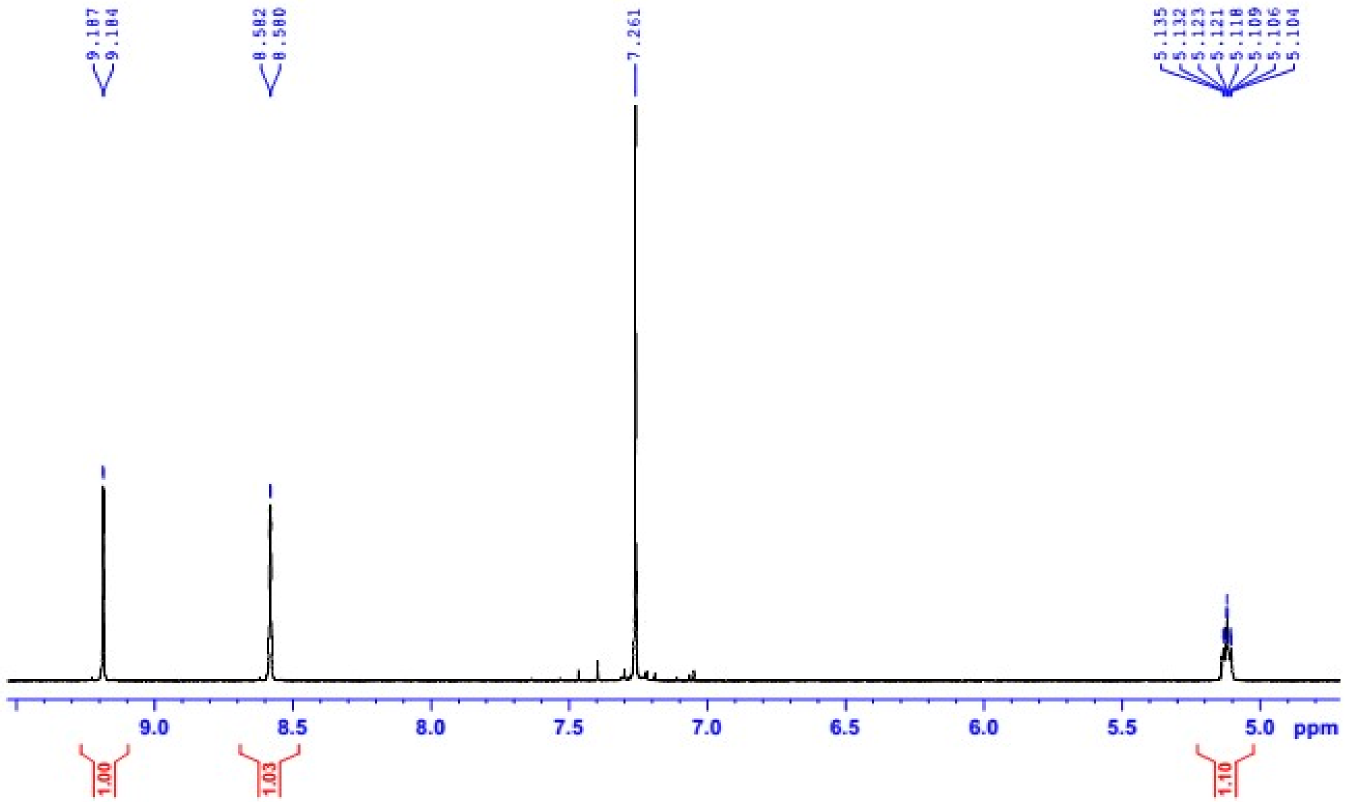


$^{1}$H-NMR spectrum of compound **3a** (extension)

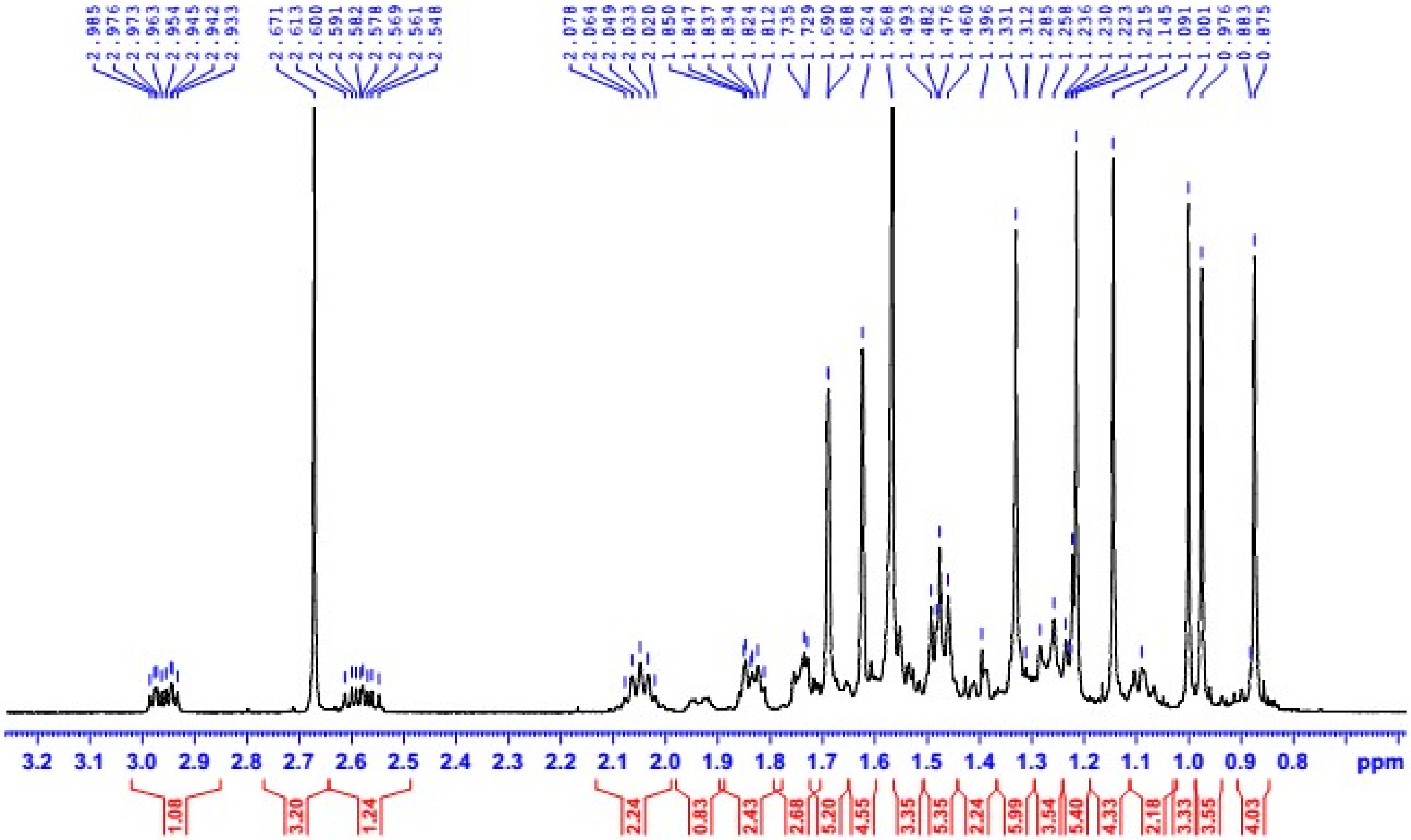


$^1$H-NMR spectrum of compound **3a** (extension)

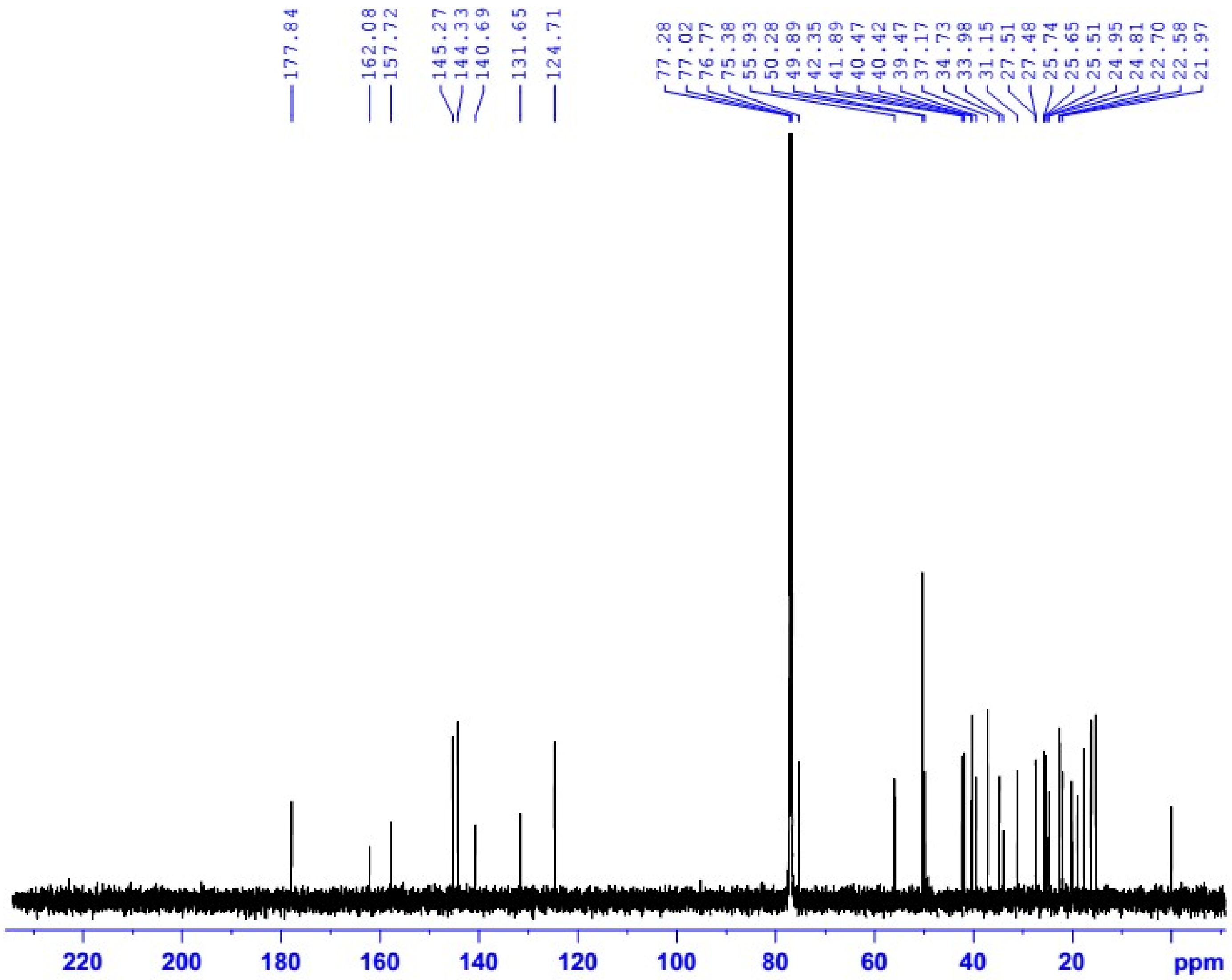


$^{13}$C-NMR spectrum of compound **3a**

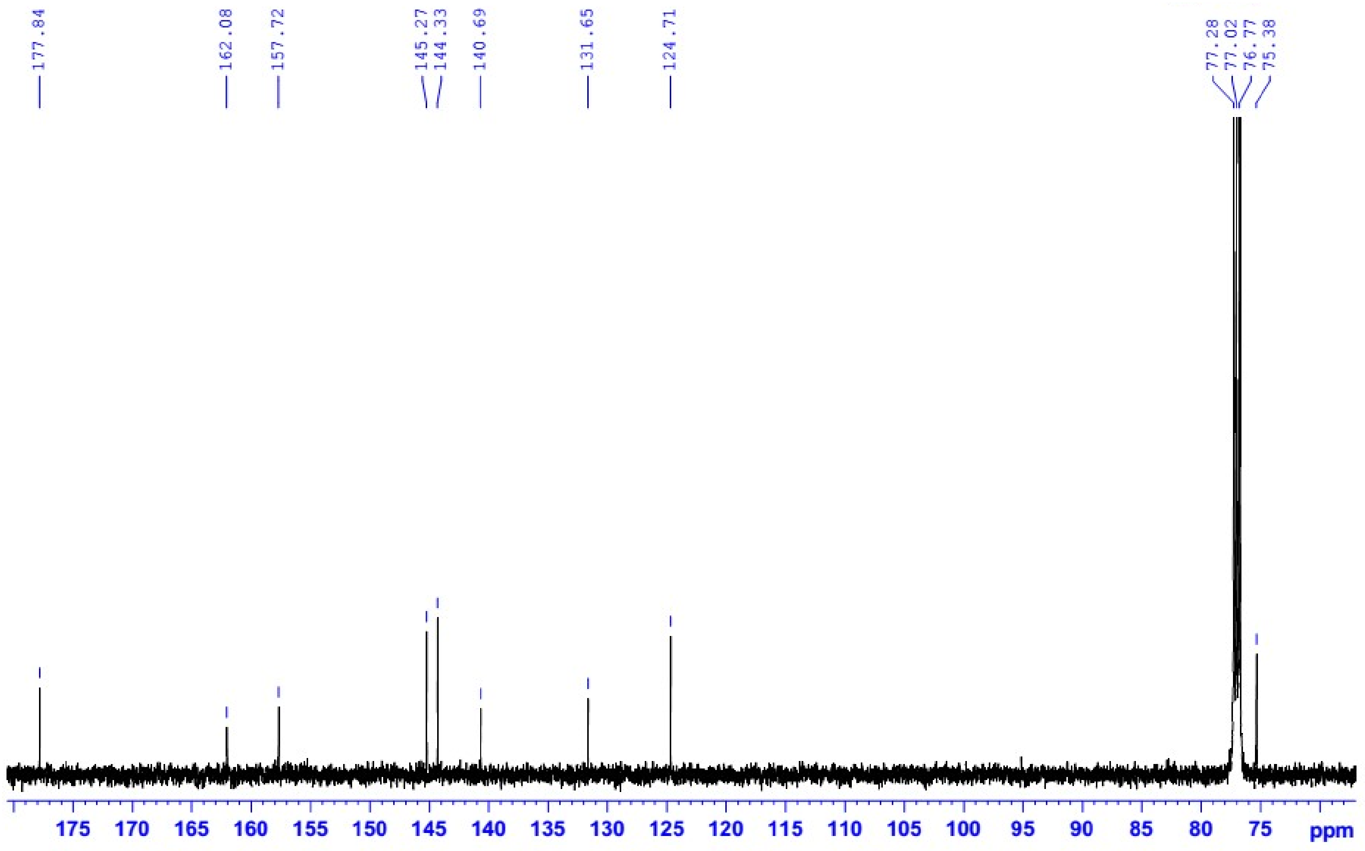


$^{13}C$-NMR spectrum of compound **3a** (extension)

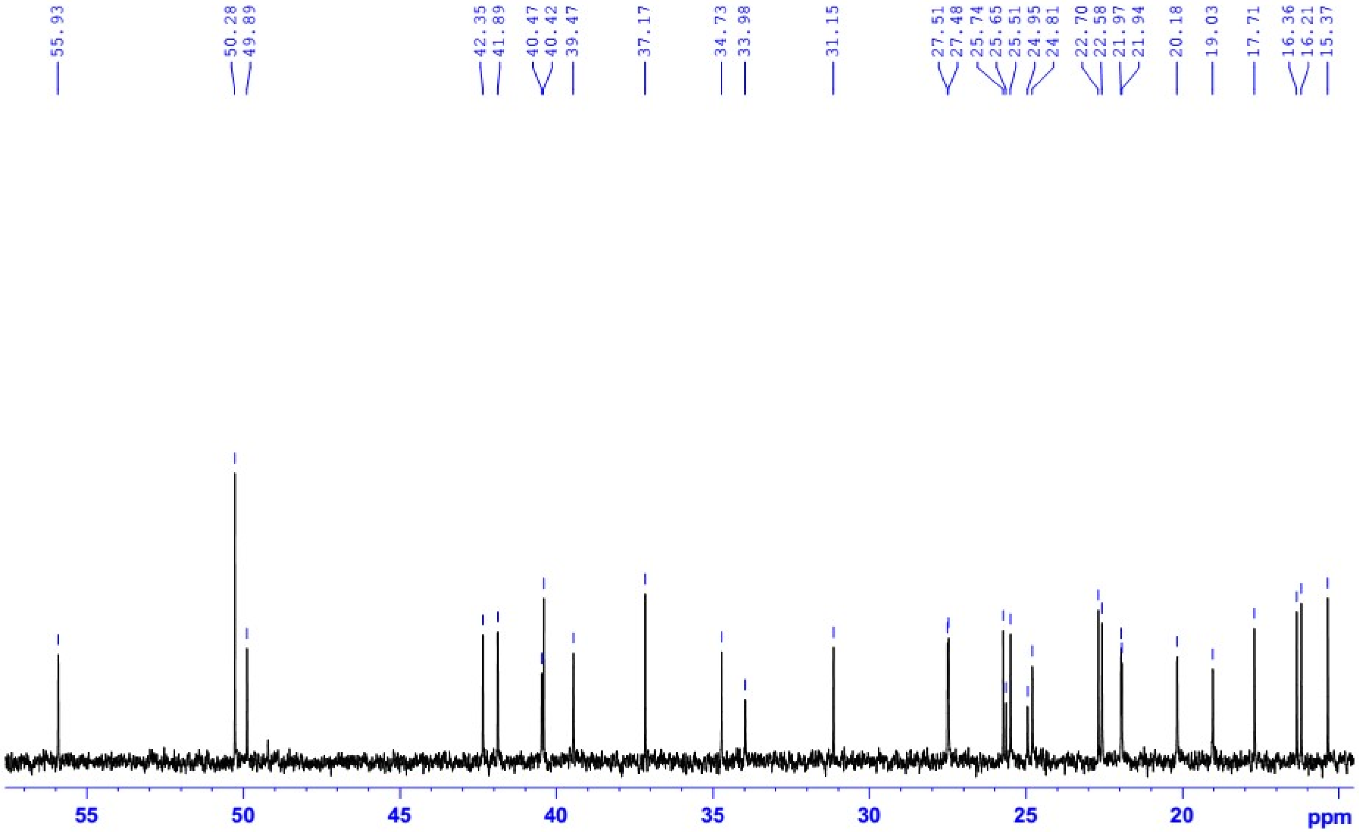


$^{13}$C-NMR spectrum of compound **3a** (extension)

## 1.4. Compound 3b

***Sample name: DNcinnamic***
***Operator: Le Anh VHH***
***Method: +IDA TOF MS/MS***
***Date: 2021.04.23***

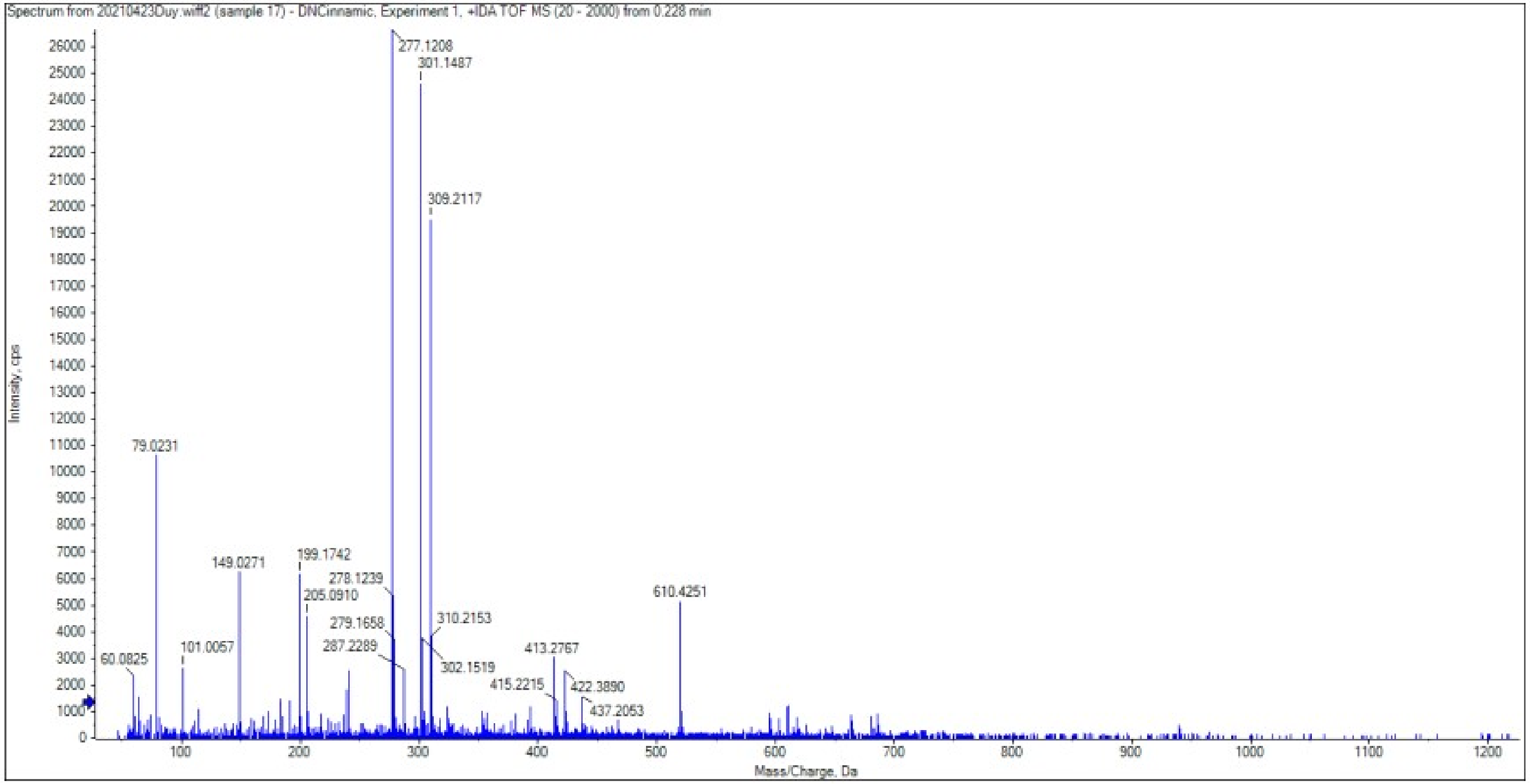


| Hit | Formula | m/z | RDB | ppm | MS Rank | MSMS ppm | MSMS Rank | Found |
|---|---|---|---|---|---|---|---|---|
| 1 | C39H57NO3 | 610.42361 | 12 | 2.4 | 1 | | | NA/NA |

(+)-HR-ESI-MS spectrum of compound **3b**

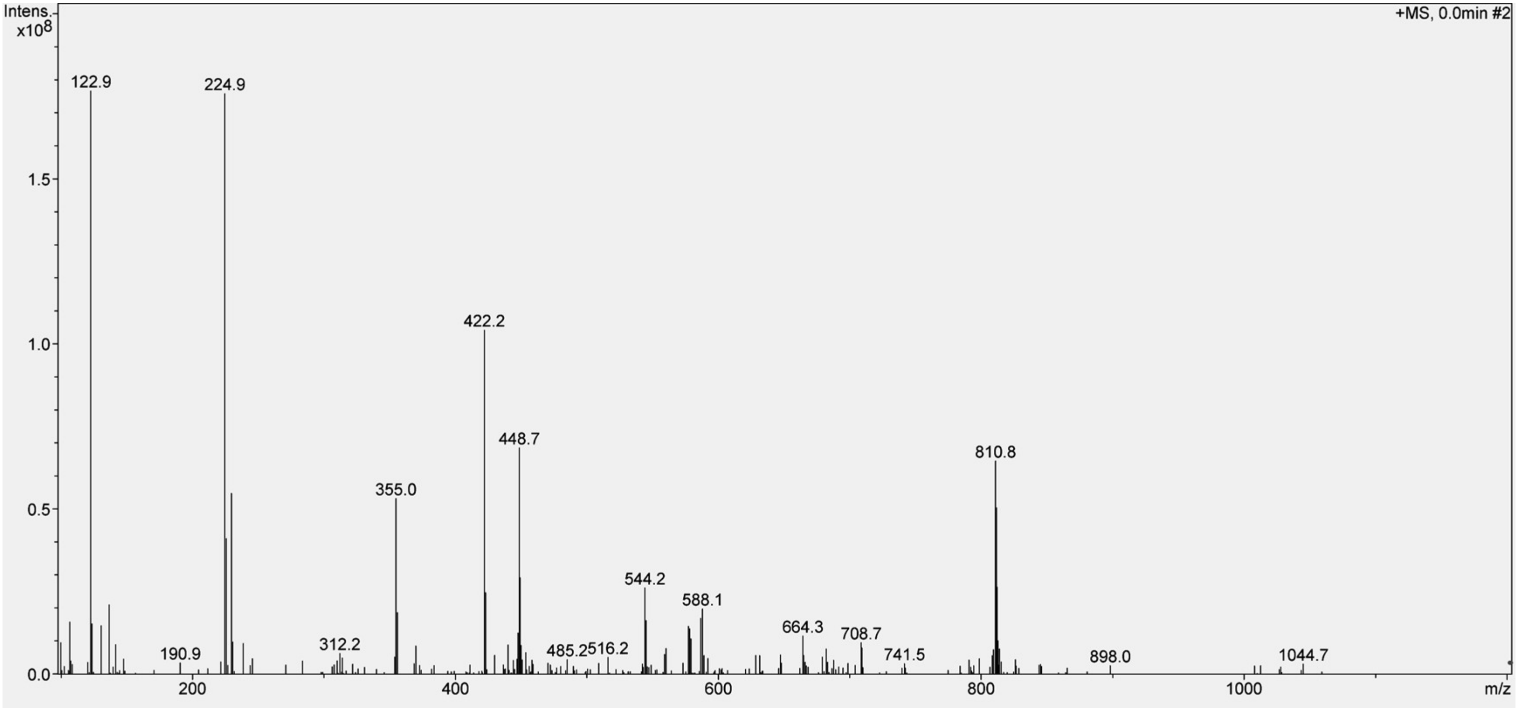


(+)-ESI–MS spectrum of compound **3b**

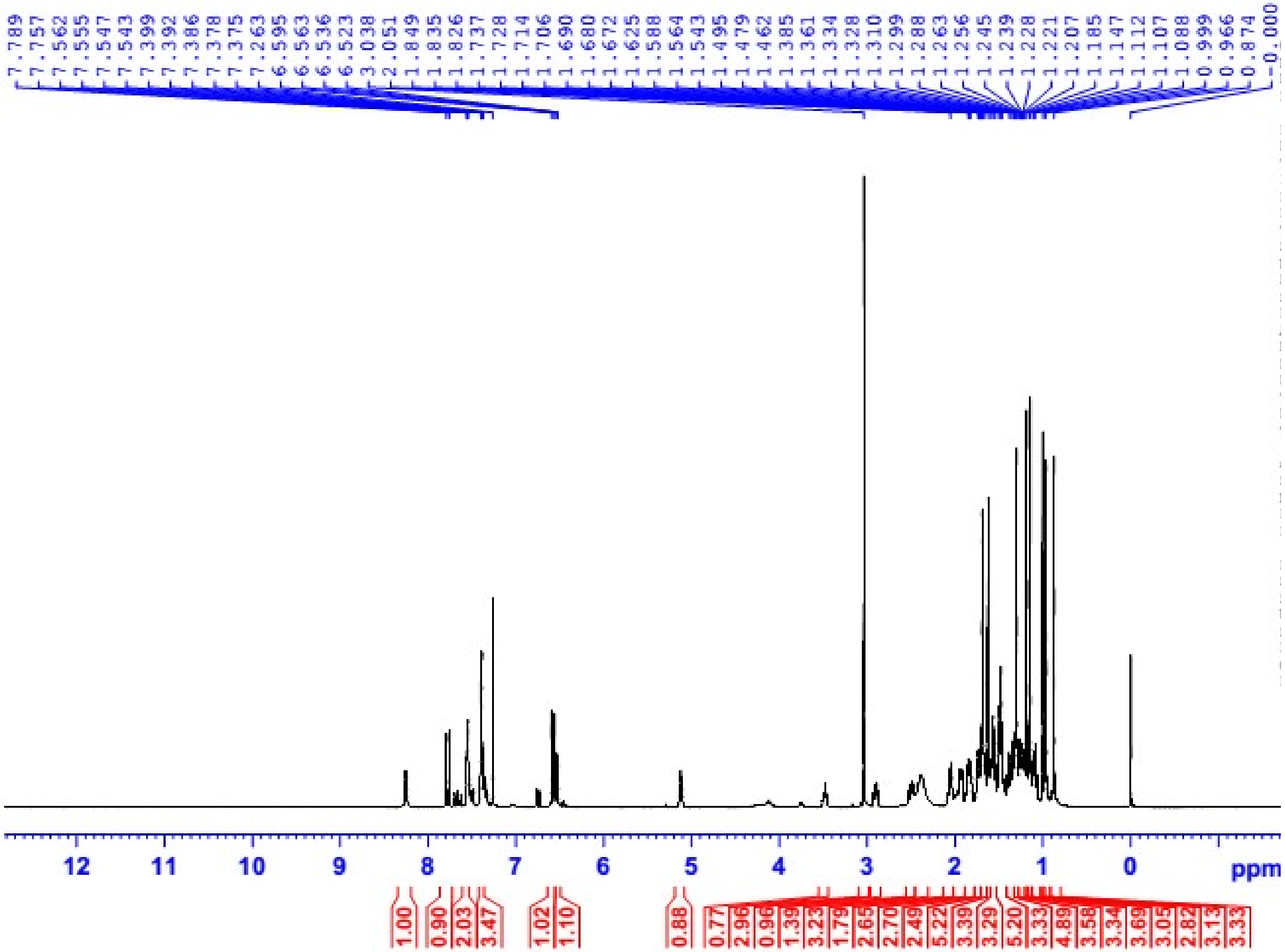

$^{1}$H-NMR spectrum of compound **3b**

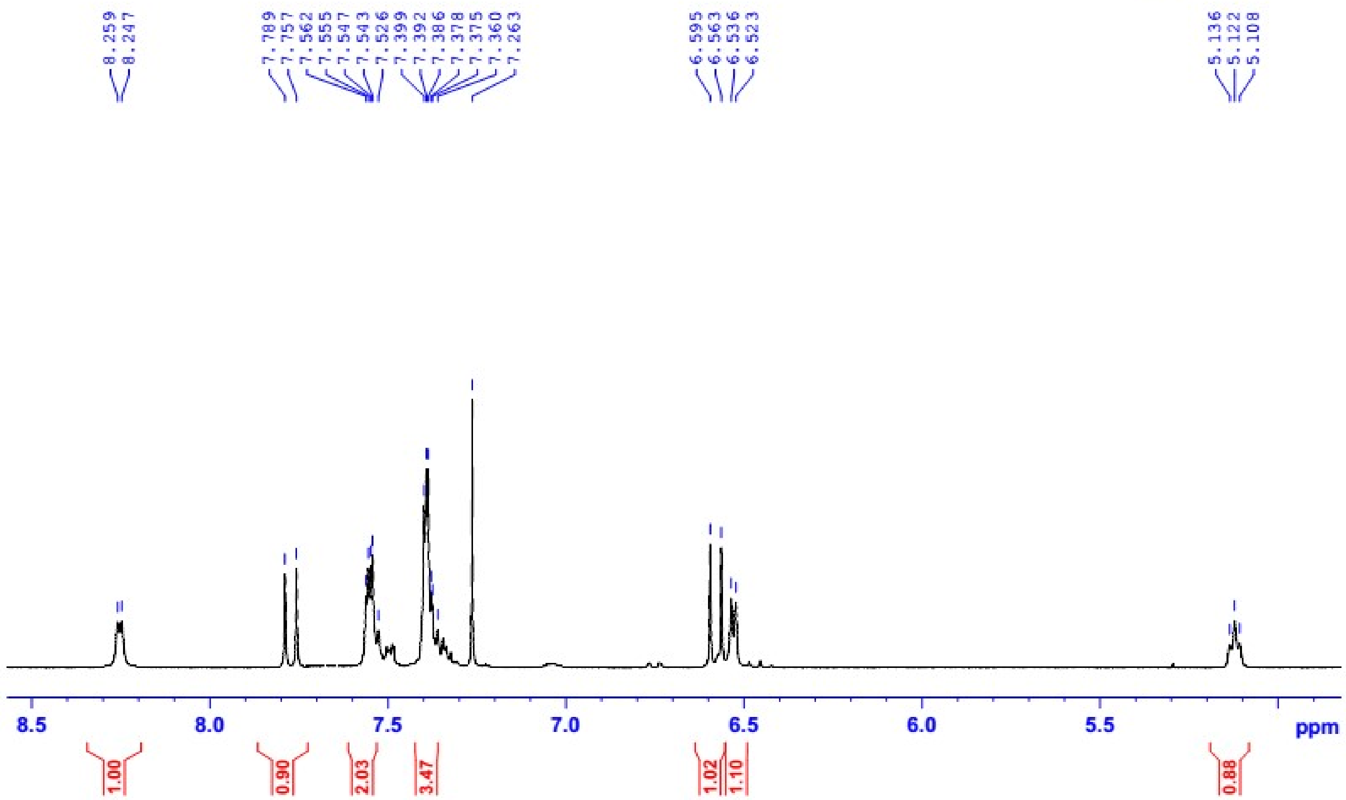


$^{1}$H-NMR spectrum of compound **3b** (extension)

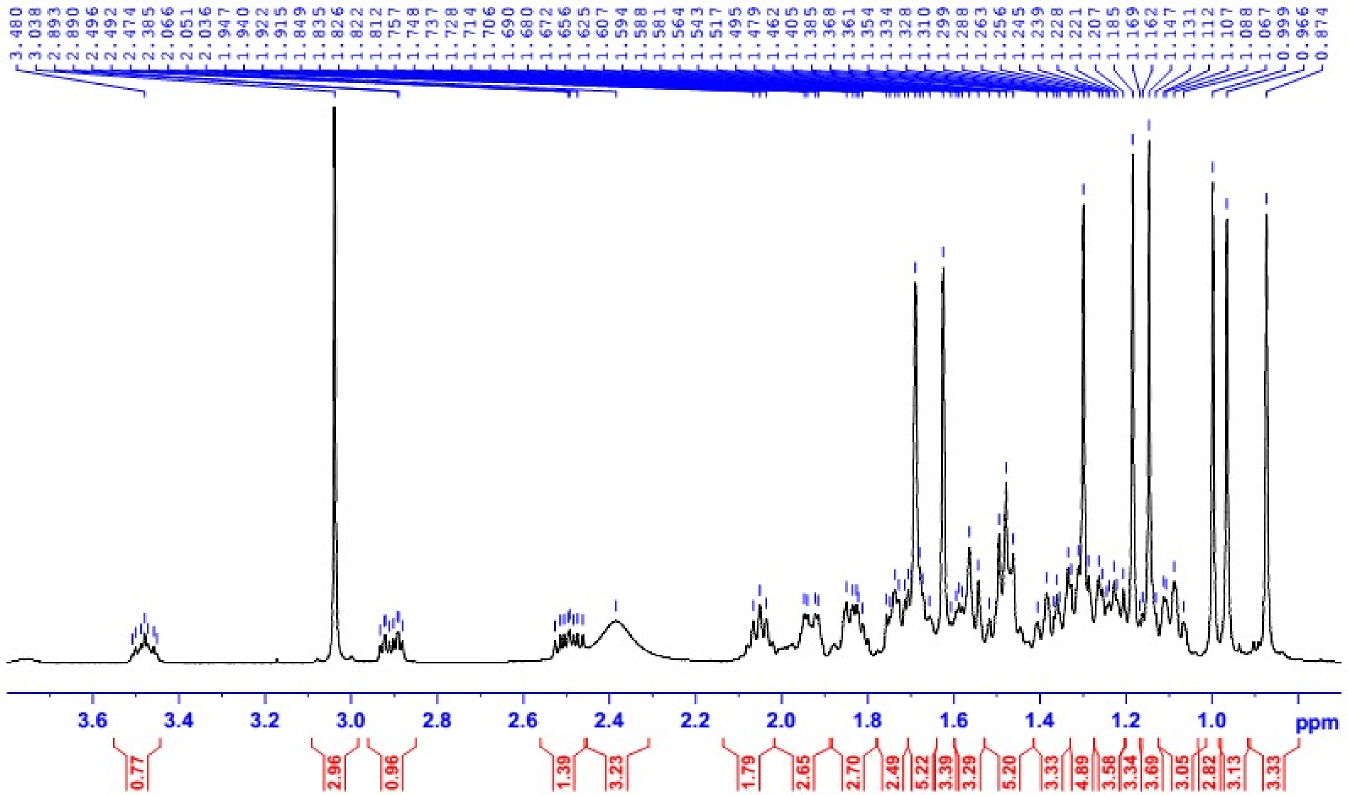


$^{1}$H-NMR spectrum of compound **3b** (extension)

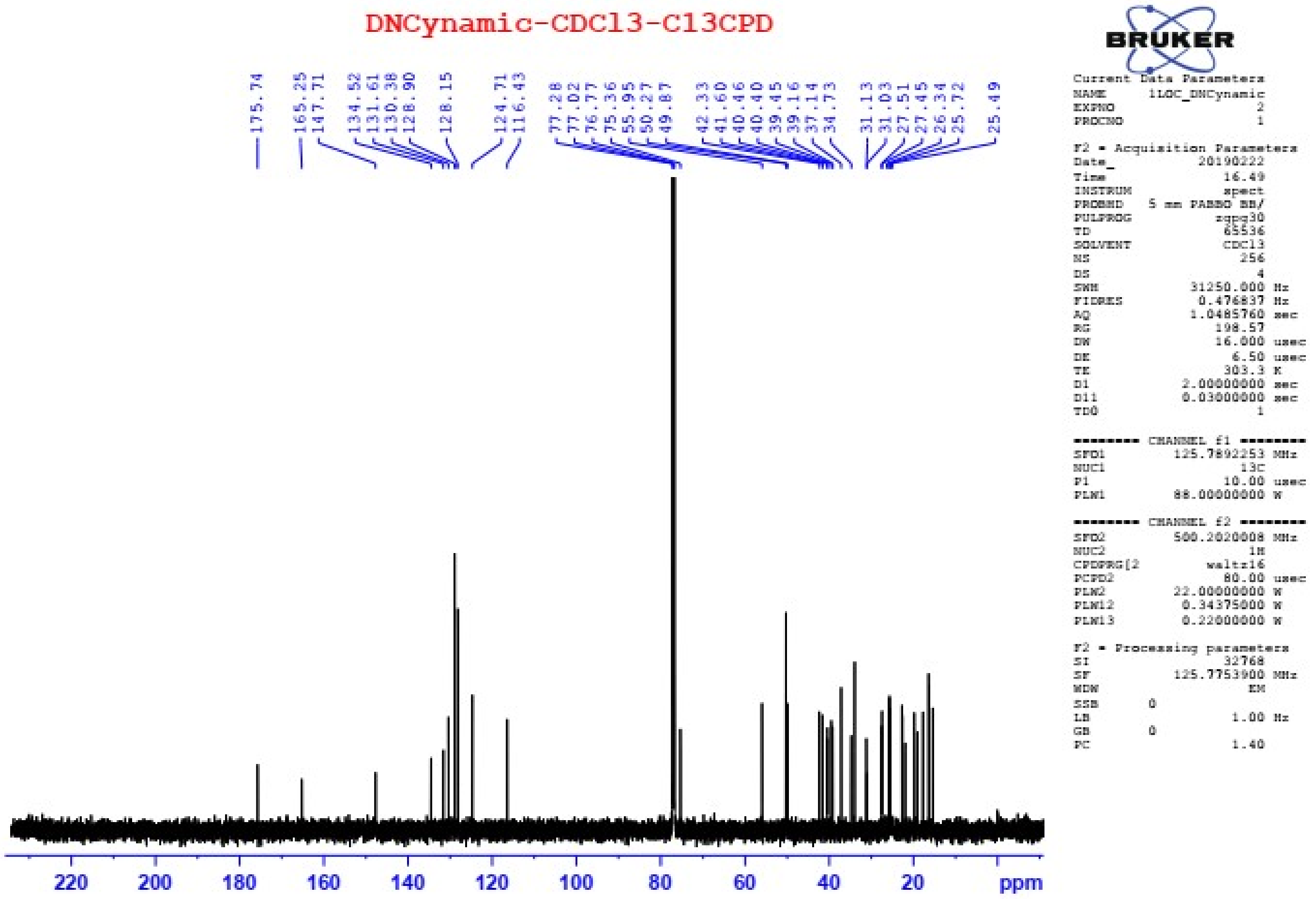


$^{13}$C-NMR spectrum of compound **3b**

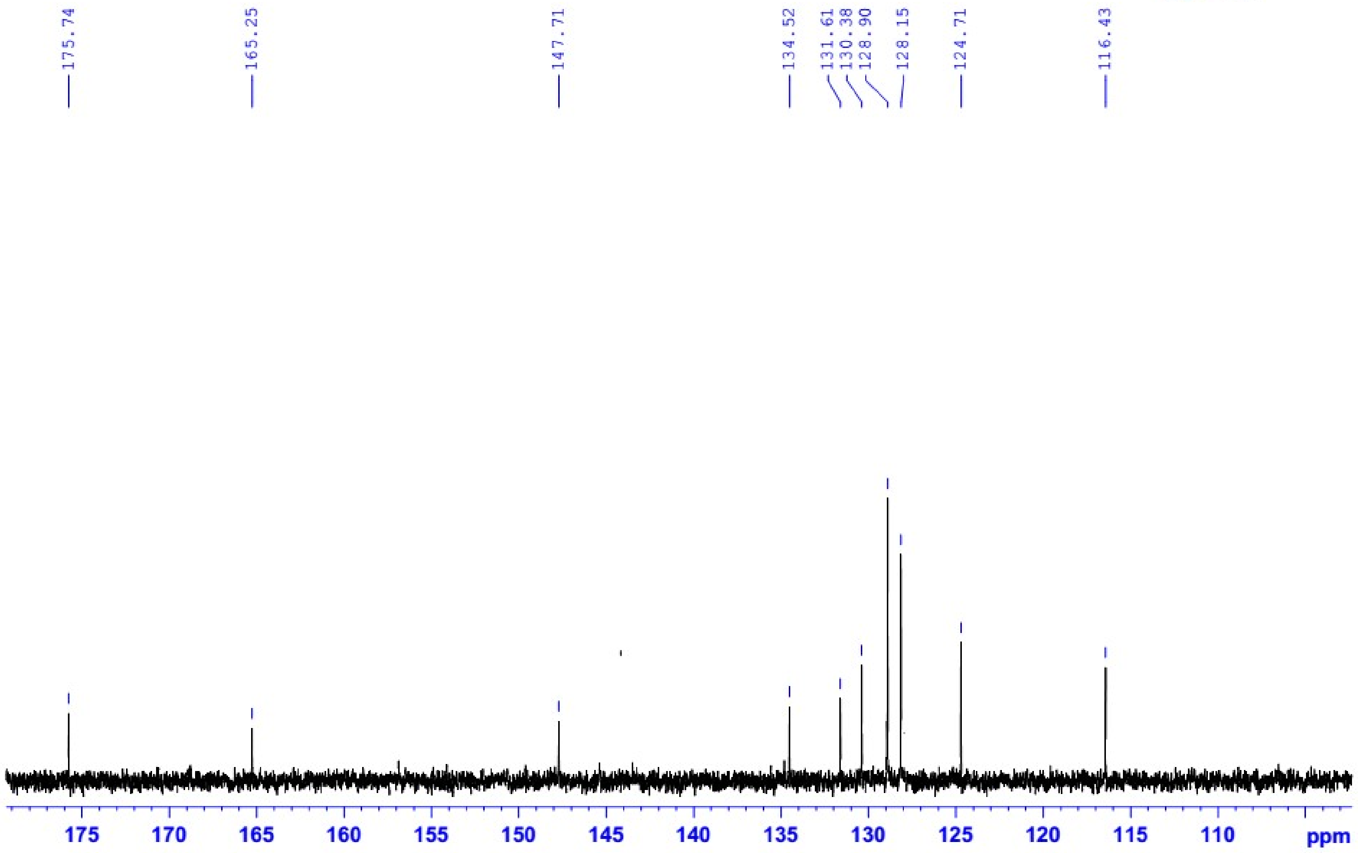


$^{13}$C-NMR spectrum of compound **3b** (extension)

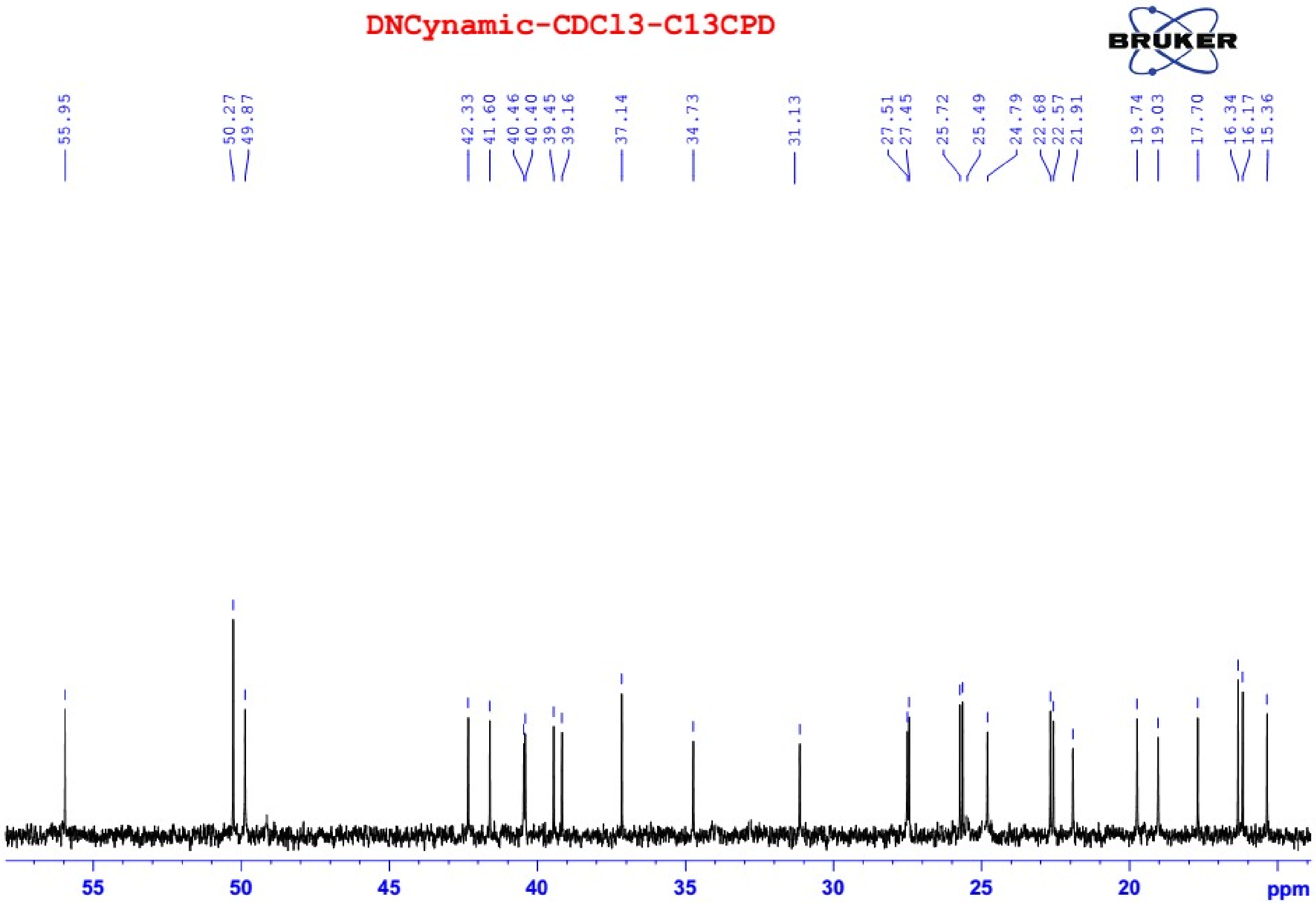


$^{13}$C-NMR spectrum of compound **3b** (extension)

## 1.5. Compound 3c

***Sample name: DNBenzoic***
***Operator: Le Anh VHH***
***Method: +IDA TOF MS/MS***
***Date: 2021.04.23***

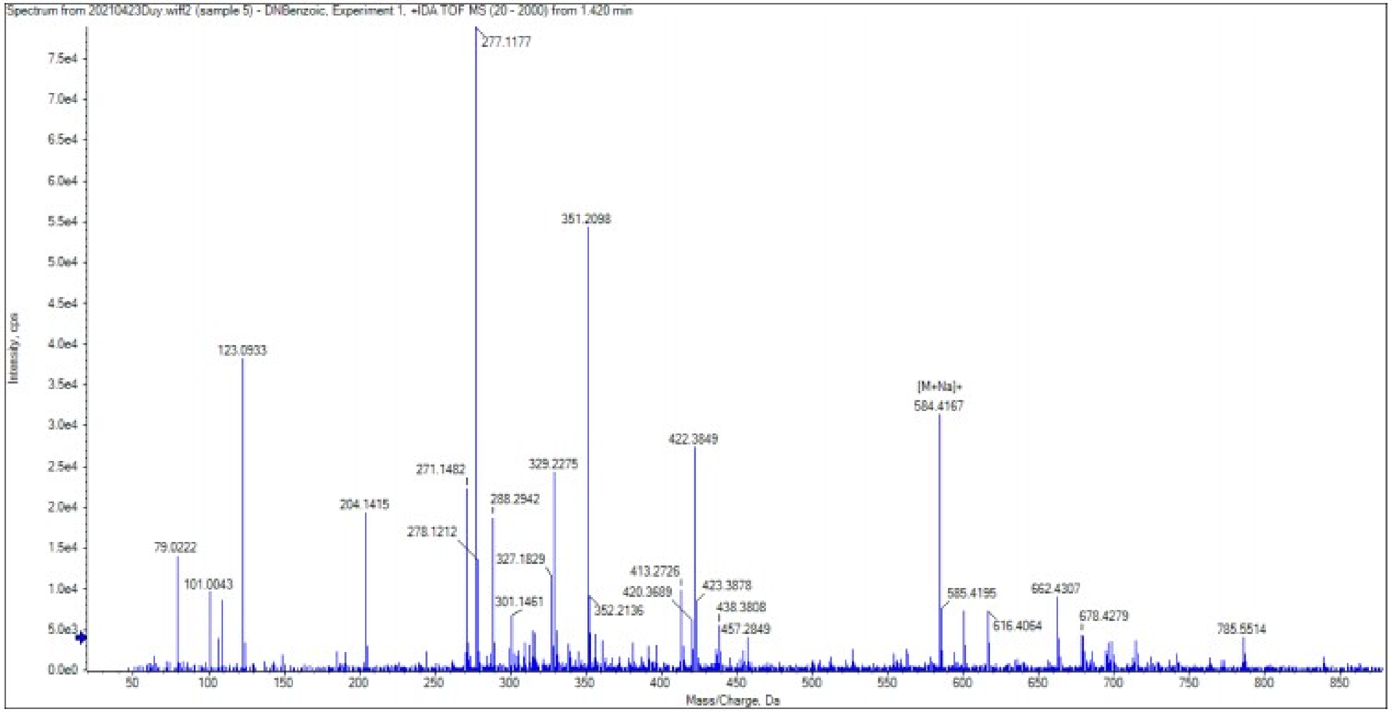


| Hit | Formula | m/z | RDB | ppm | MS Rank | MSMS ppm | MSMS Rank | Found |
|---|---|---|---|---|---|---|---|---|
| 1 | C37H55NO3 | 584.4142 | 11.0 | 3.0 | 1 | | | NA/NA |

*Device Model:* SCIEX X500 QTOF

(+)-HR-ESI-MS spectrum of compound **3c**

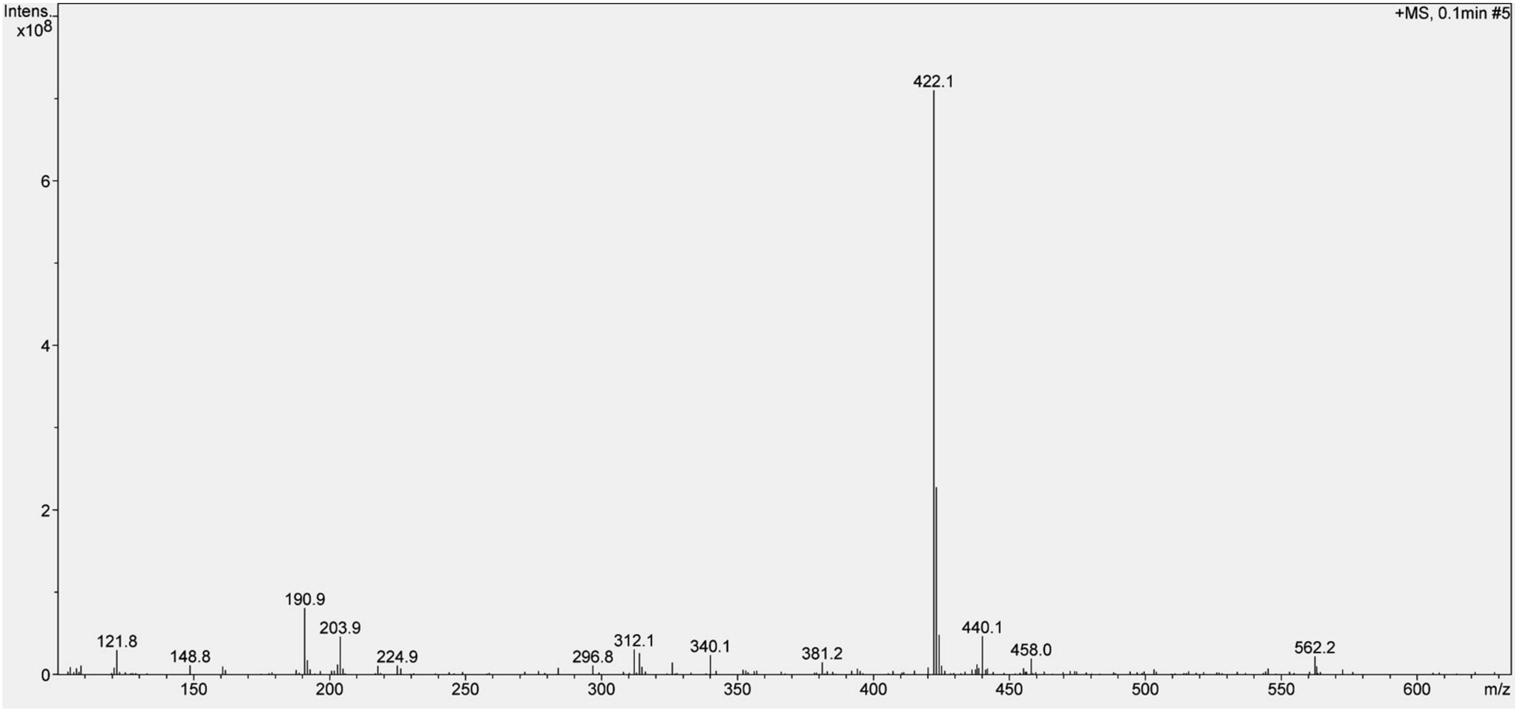


(+)-ESI-MS spectrum of compound **3c**

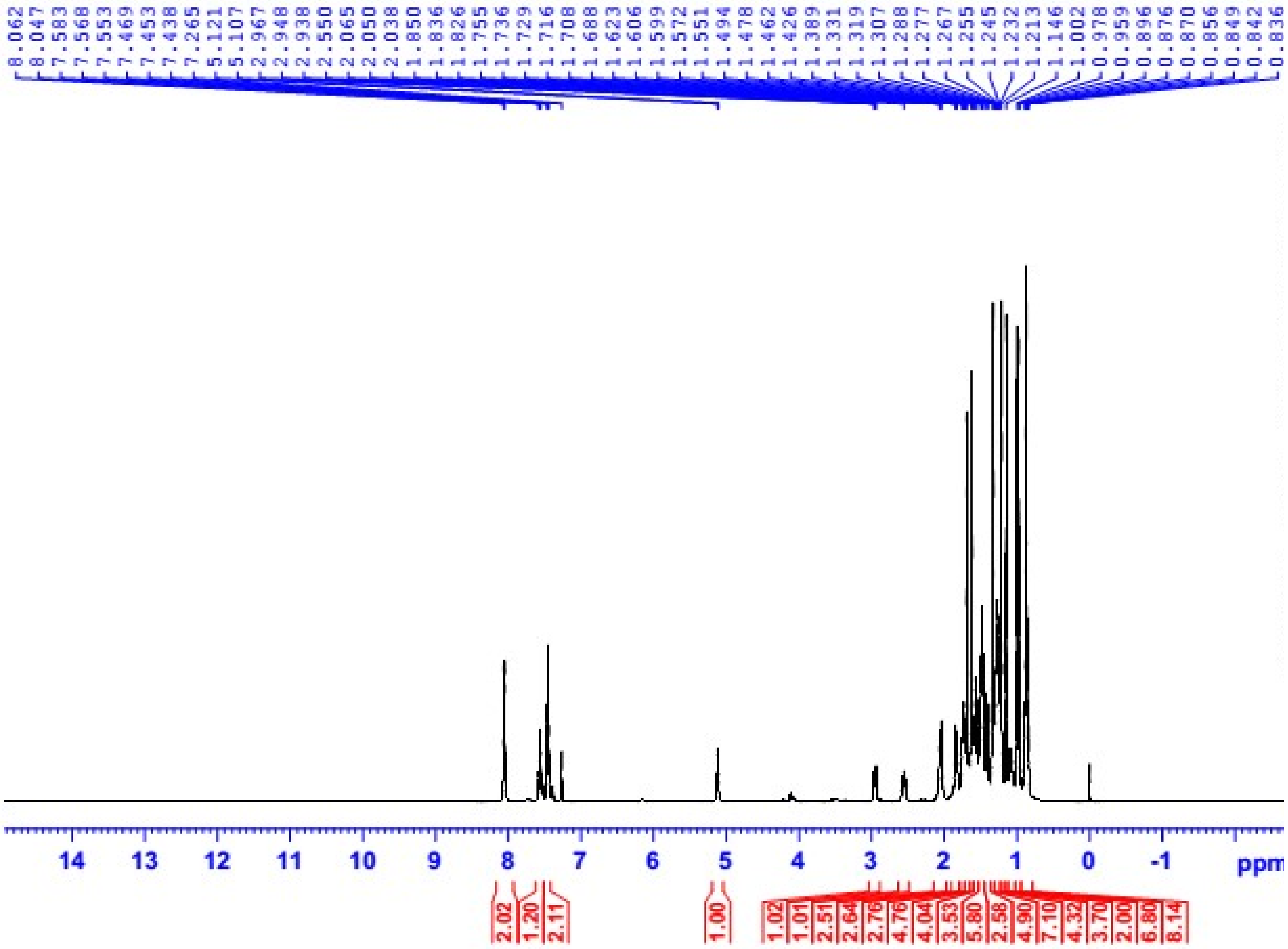


$^{1}$H-NMR spectrum of compound **3c**

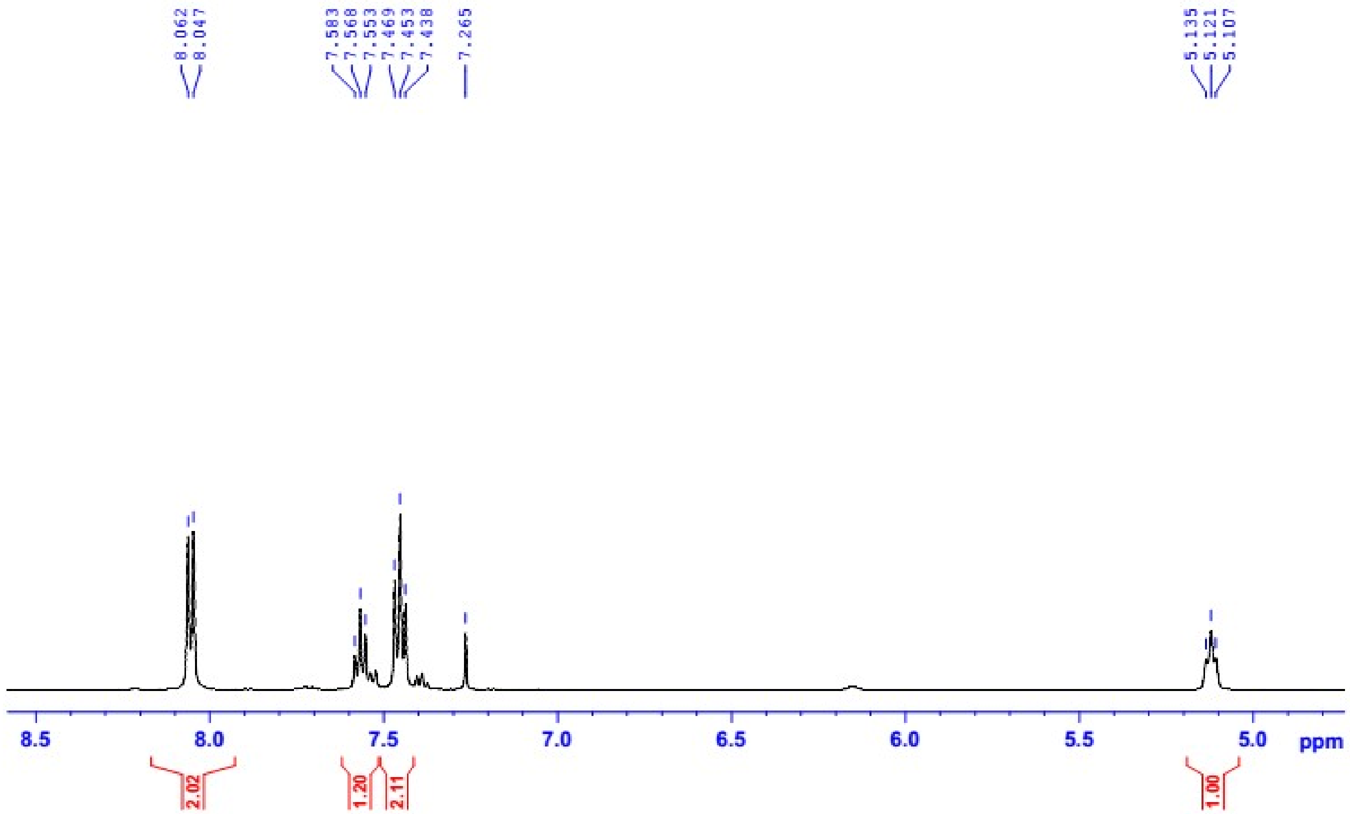


$^{1}$H-NMR spectrum of compound **3c** (extension)

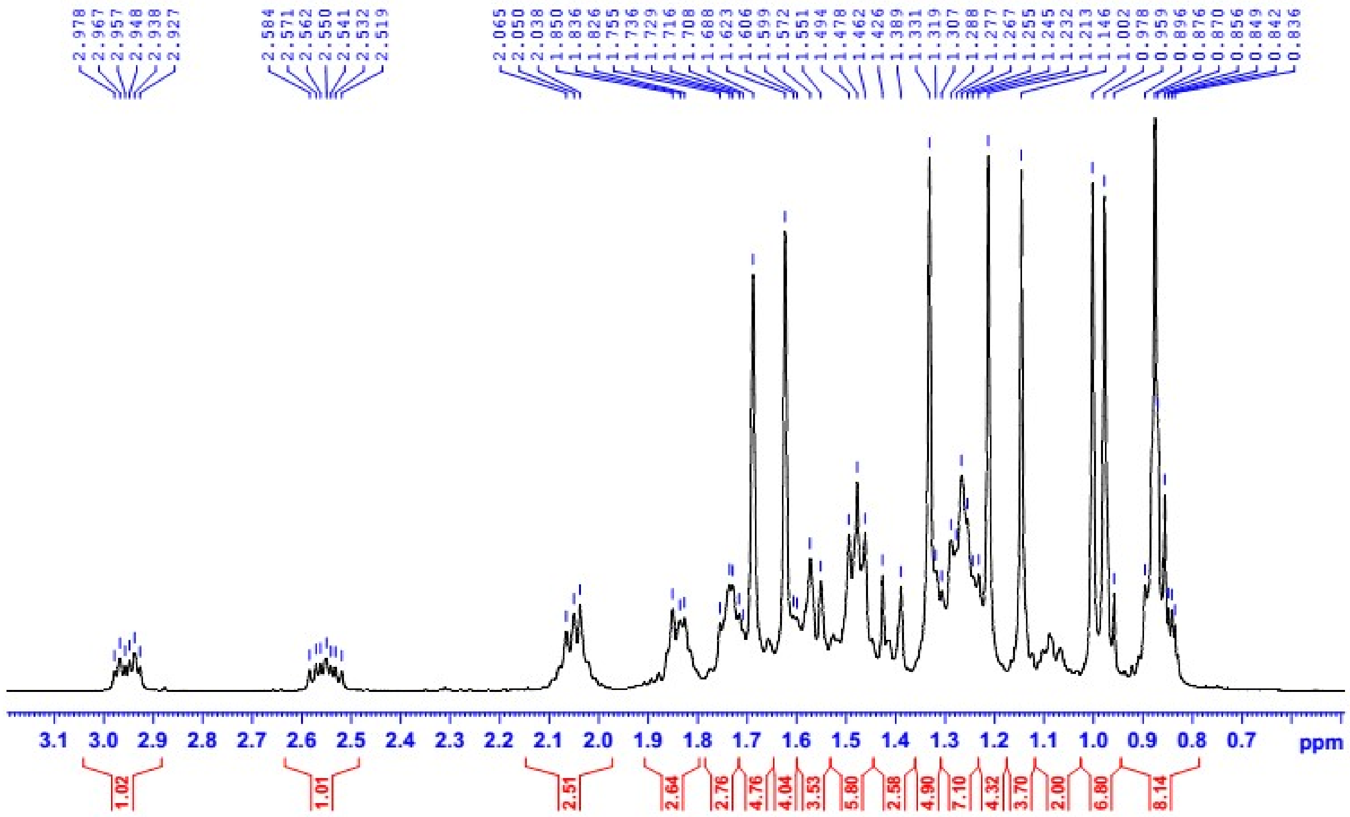

$^{1}$H-NMR spectrum of compound **3c** (extension)

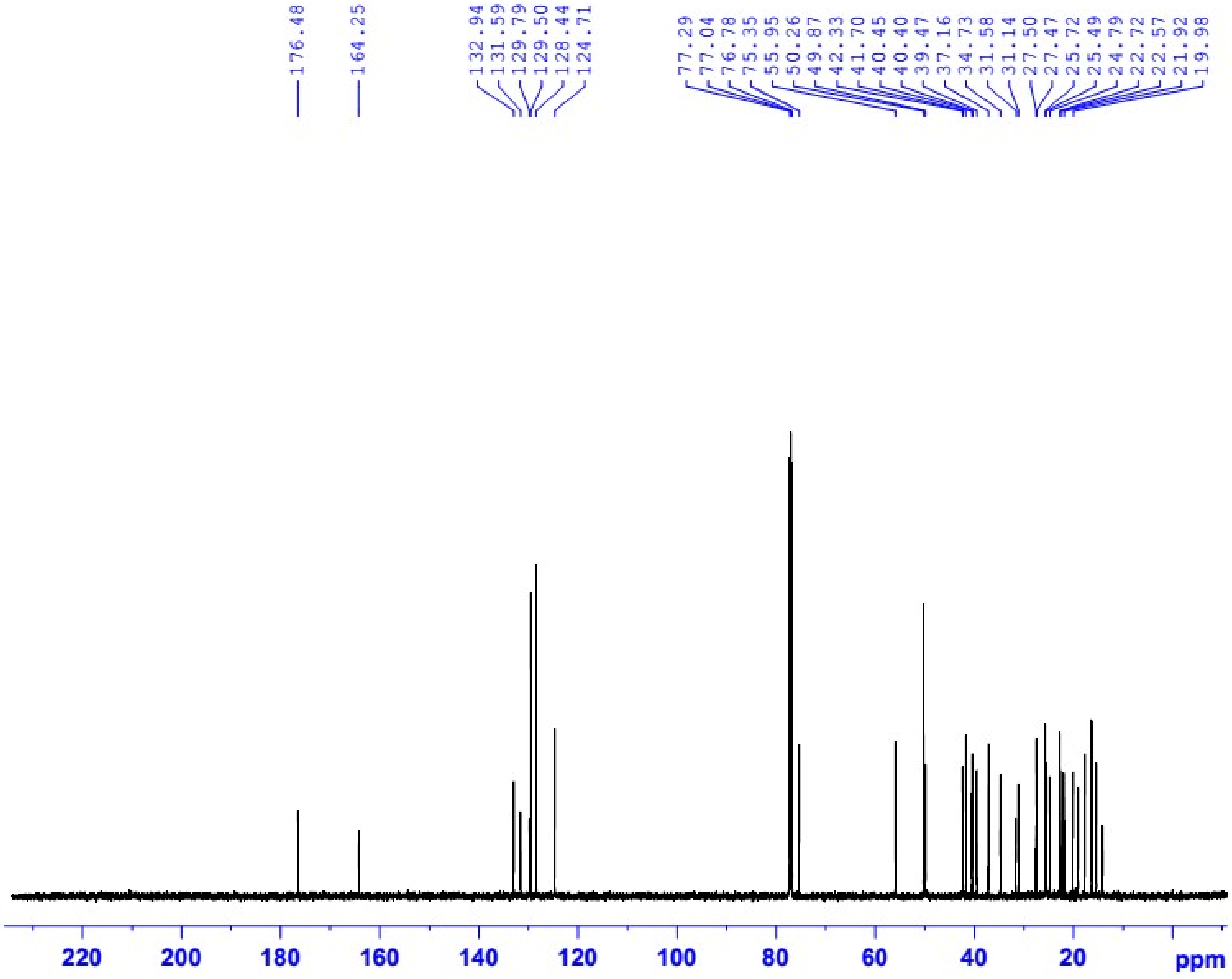


$^{13}C$-NMR spectrum of compound **3c**

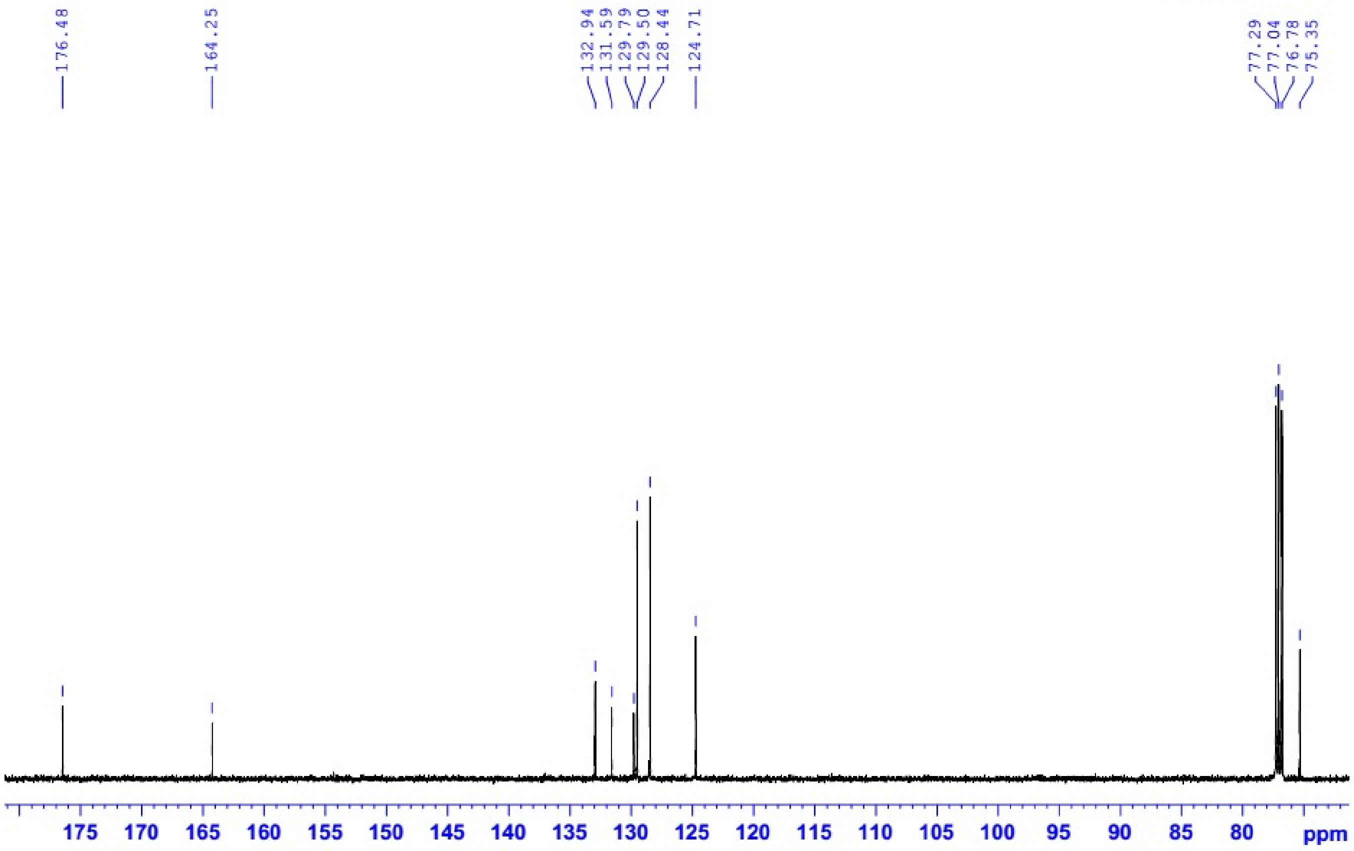


$^{13}$C-NMR spectrum of compound **3c** (extension)

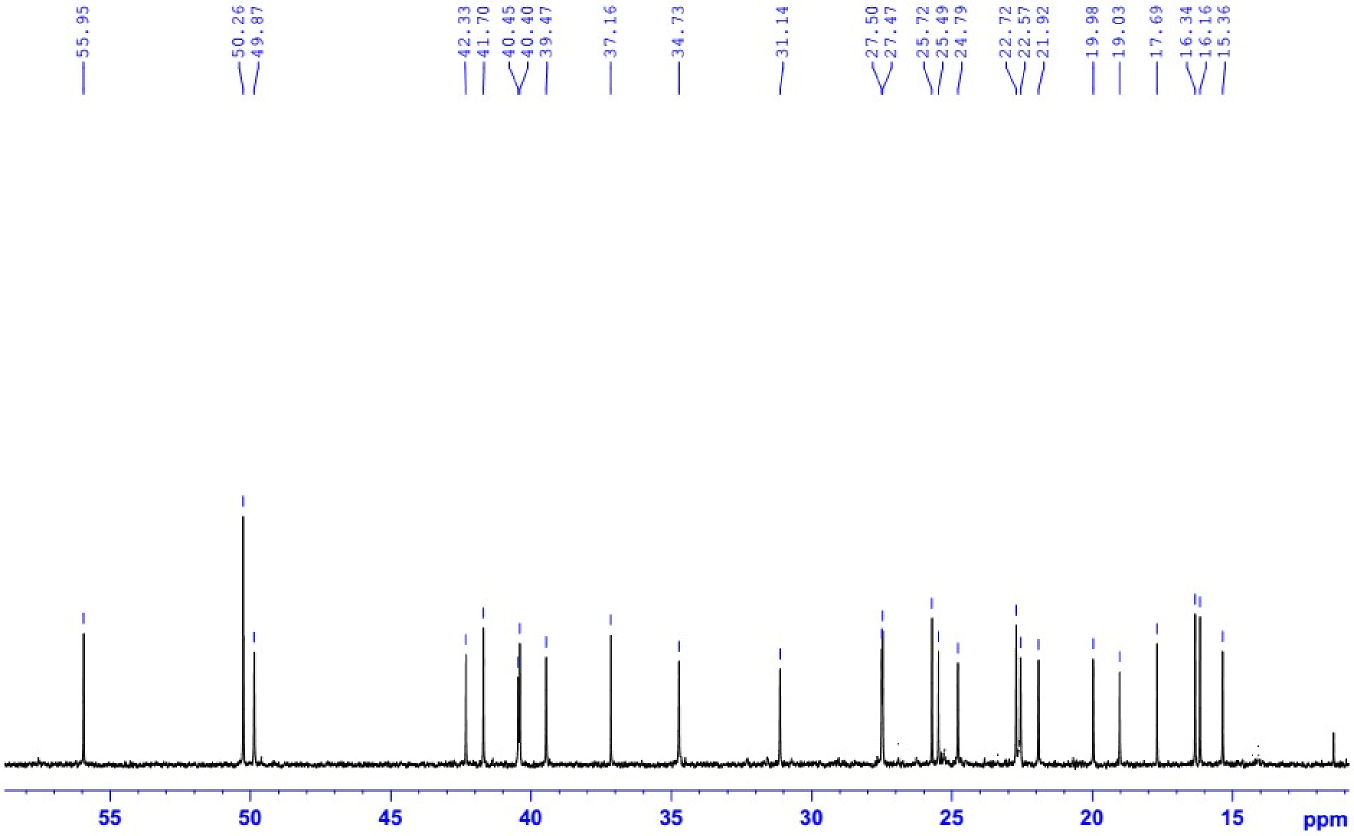


$^{13}$C-NMR spectrum of compound **3c** (extension)

## 1.6. Compound 3d

***Sample name: DNII***
***Operator: Le Anh VHH***
***Method: +IDA TOF MS/MS***
***Date: 2021.04.23***

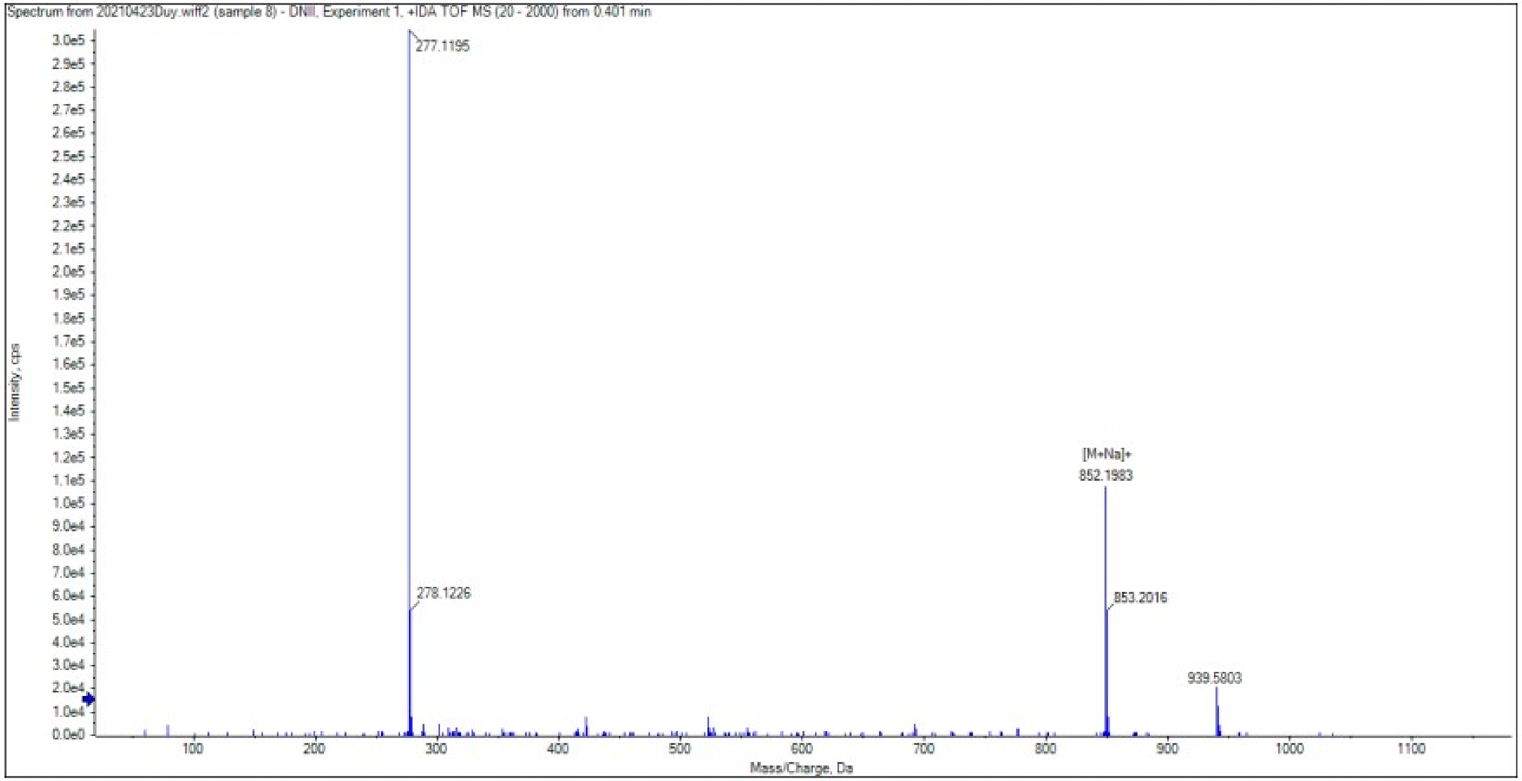


| Hit | Formula | m/z | RDB | ppm | MS Rank | MSMS ppm | MSMS Rank | Found |
|---|---|---|---|---|---|---|---|---|
| 1 | C37H53I2NO4 | 852.19617 | 11.0 | 2.6 | 1 | | | NA/NA |

(+)-HR-ESI-MS spectrum of compound **3d**

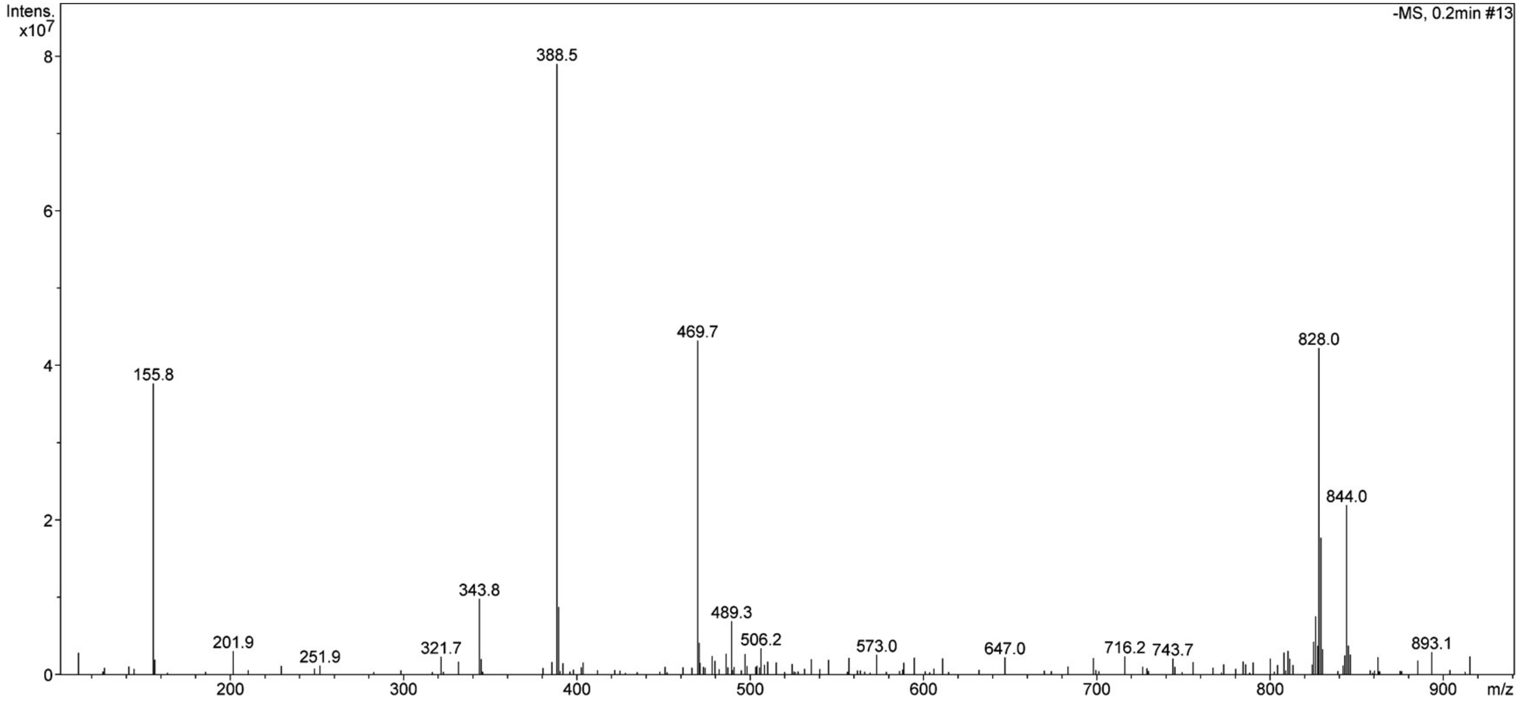


(−)-ESI–MS spectrum of compound **3d**

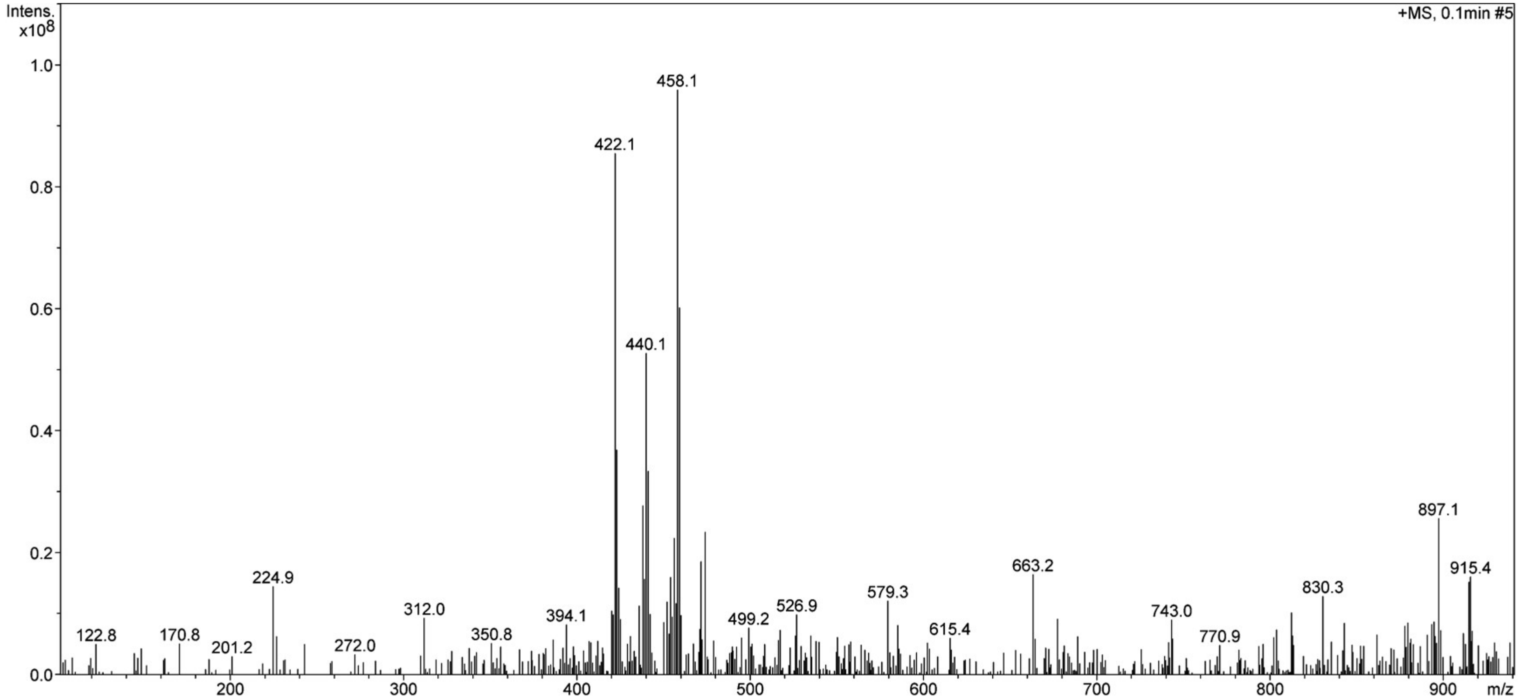


(+)-ESI–MS spectrum of compound **3d**

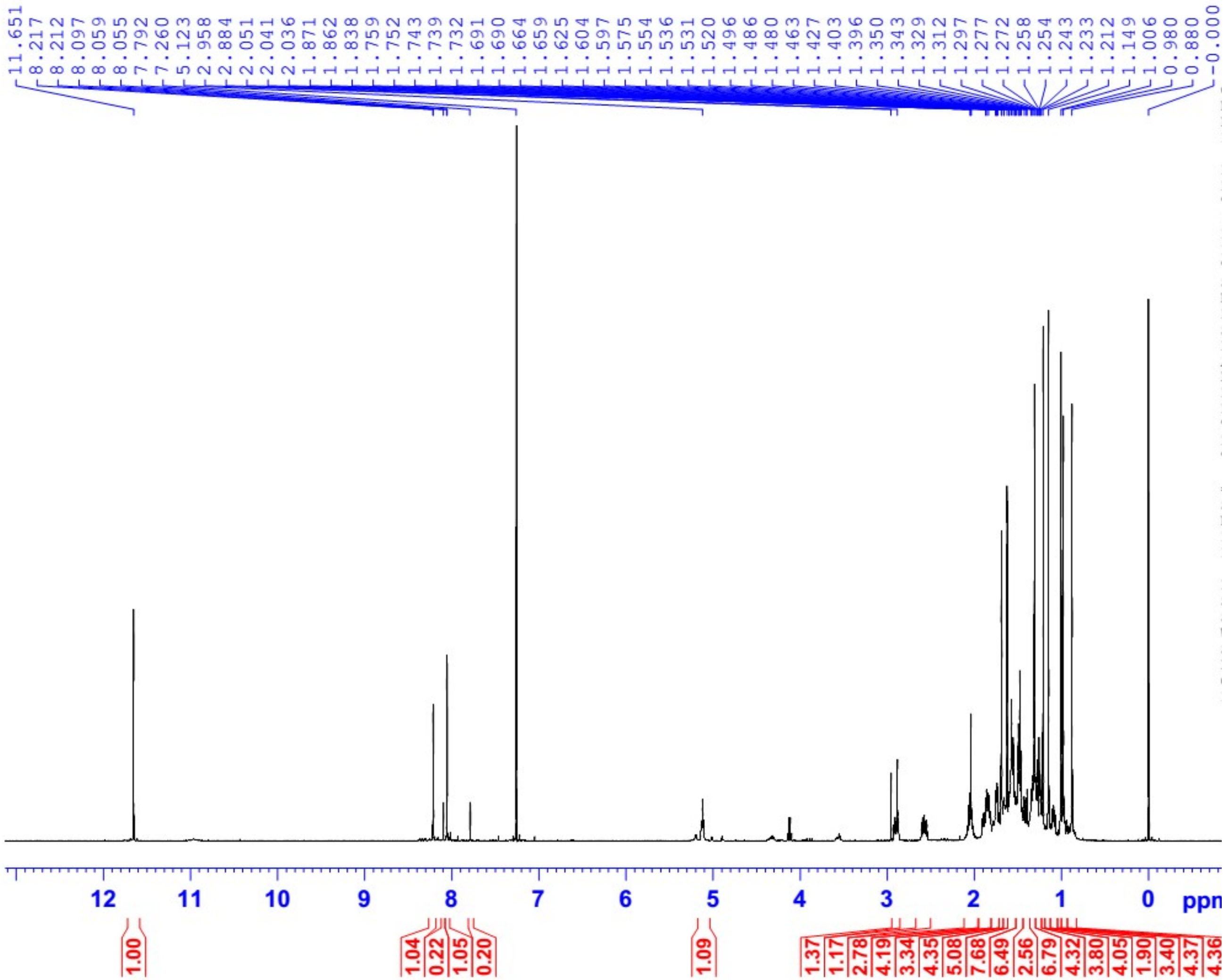

$^{1}$H-NMR spectrum of compound **3d**

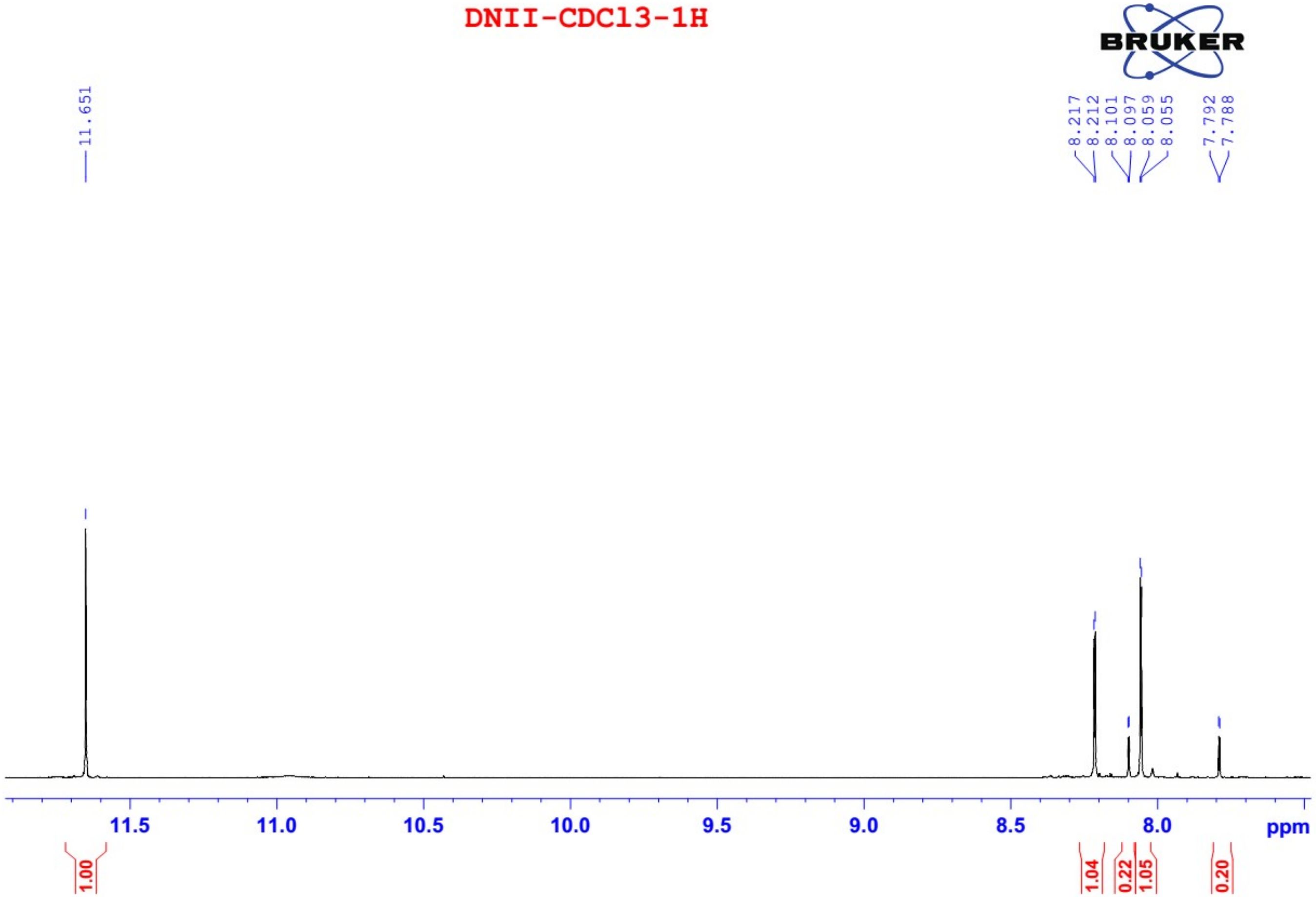


$^{1}$H-NMR spectrum of compound **3d** (extension)

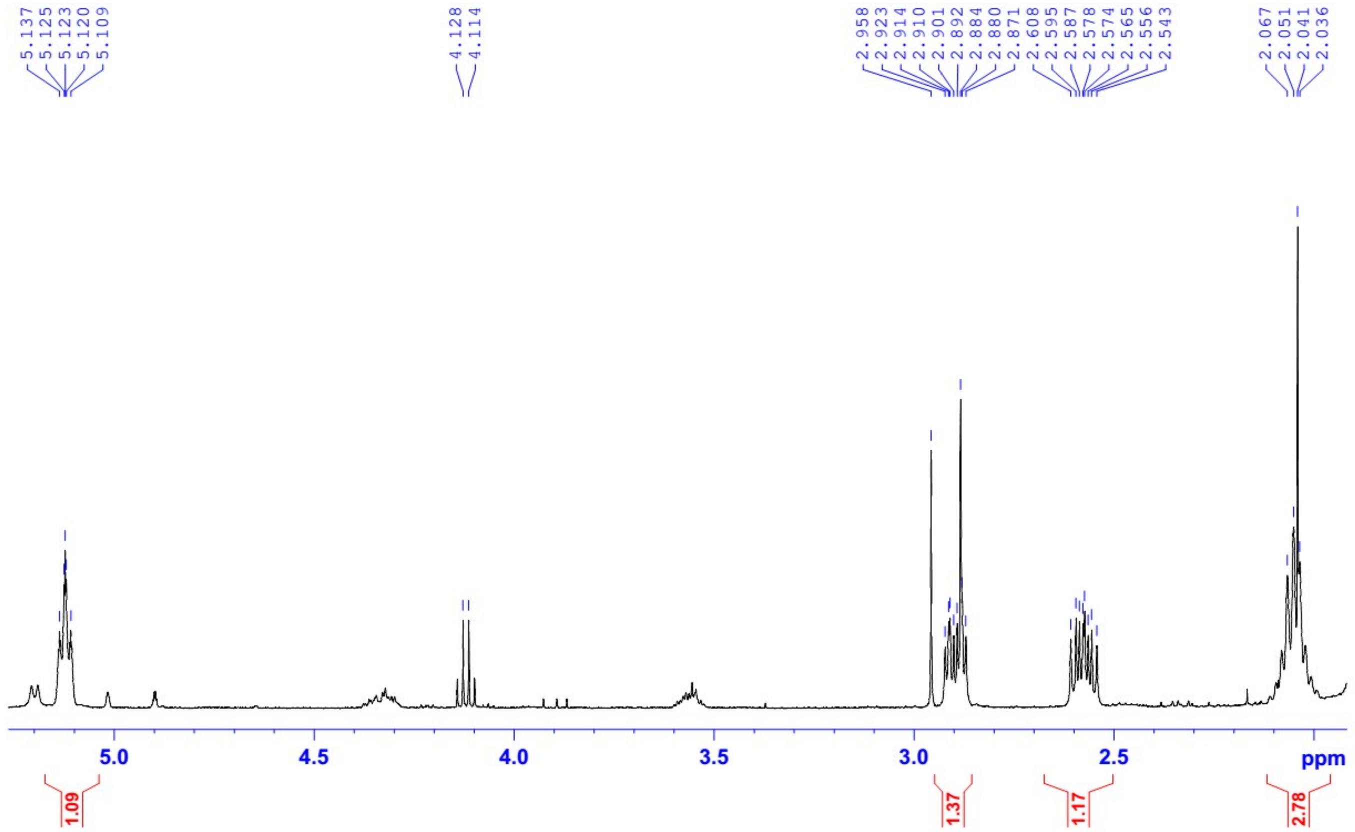


$^{1}$H-NMR spectrum of compound **3d** (extension)

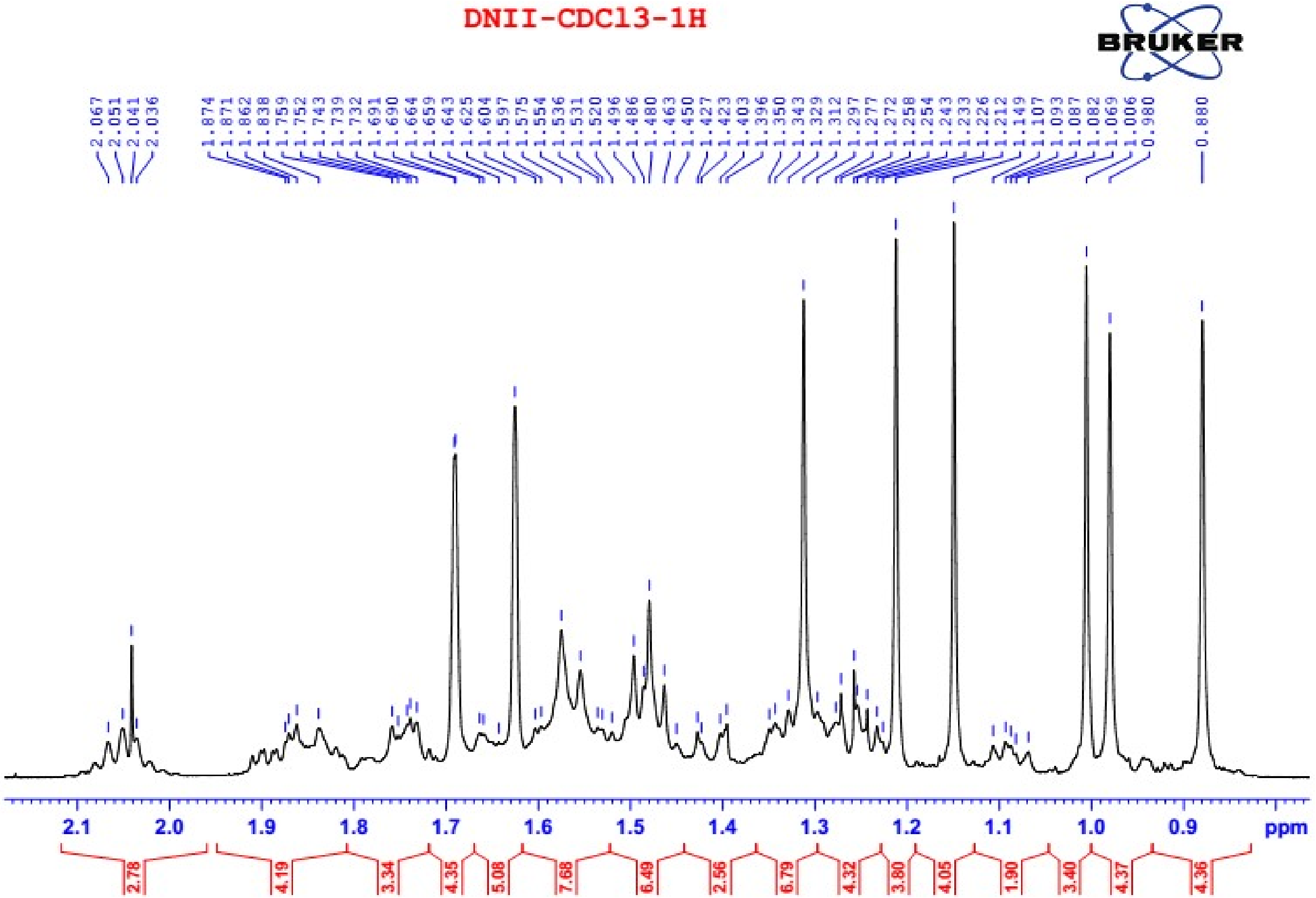

$^{1}$H-NMR spectrum of compound **3d** (extension)

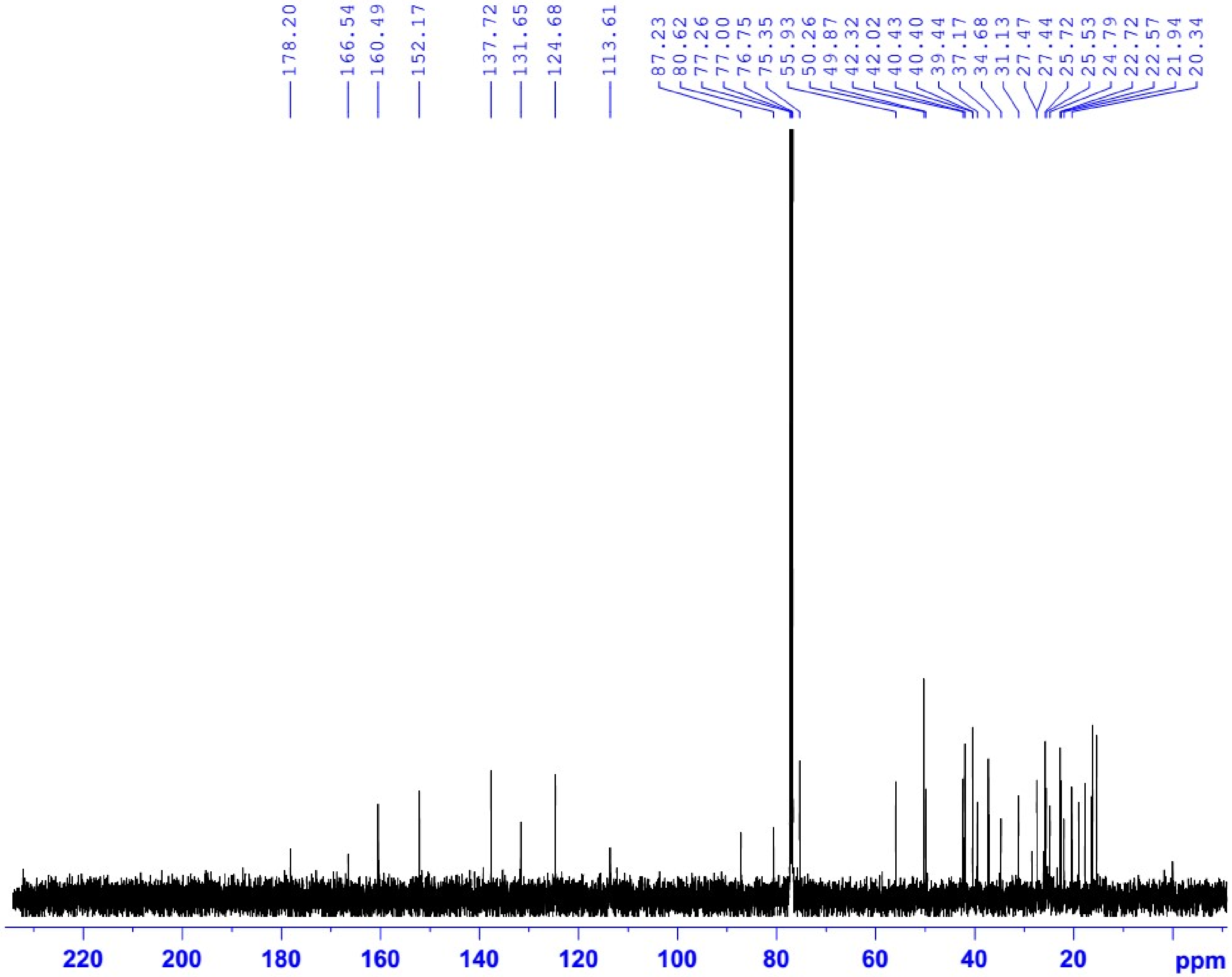


$^{13}$C-NMR spectrum of compound **3d**

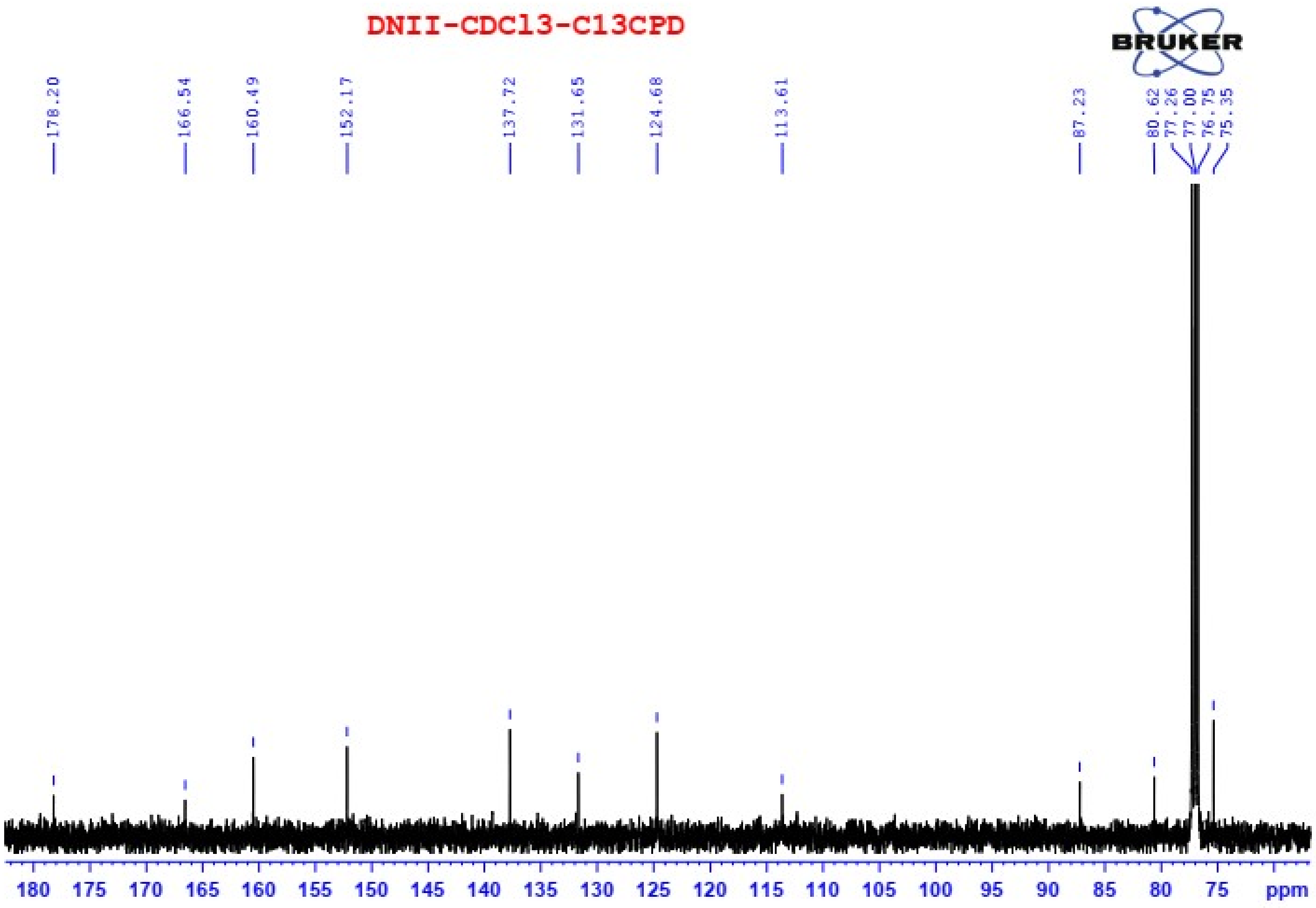


$^{13}$C-NMR spectrum of compound **3d** (extension)

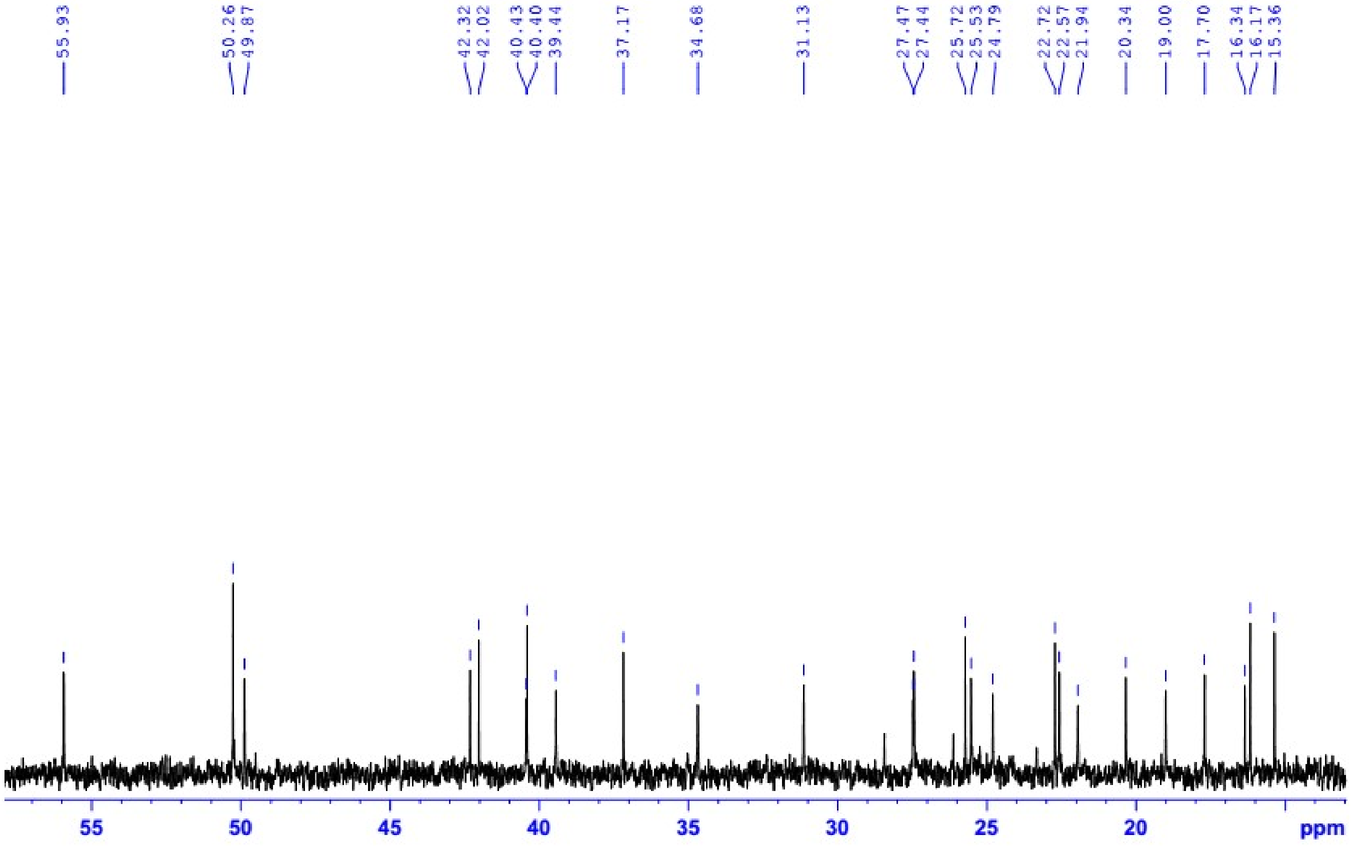


$^{13}$C-NMR spectrum of compound **3d** (extension)

### 1.7. Compound 3e

**Sample name: DNNicotinic**
**Operator: Le Anh VHH**
**Method: +IDA TOF MS/MS**
**Date: 2021.04.23**

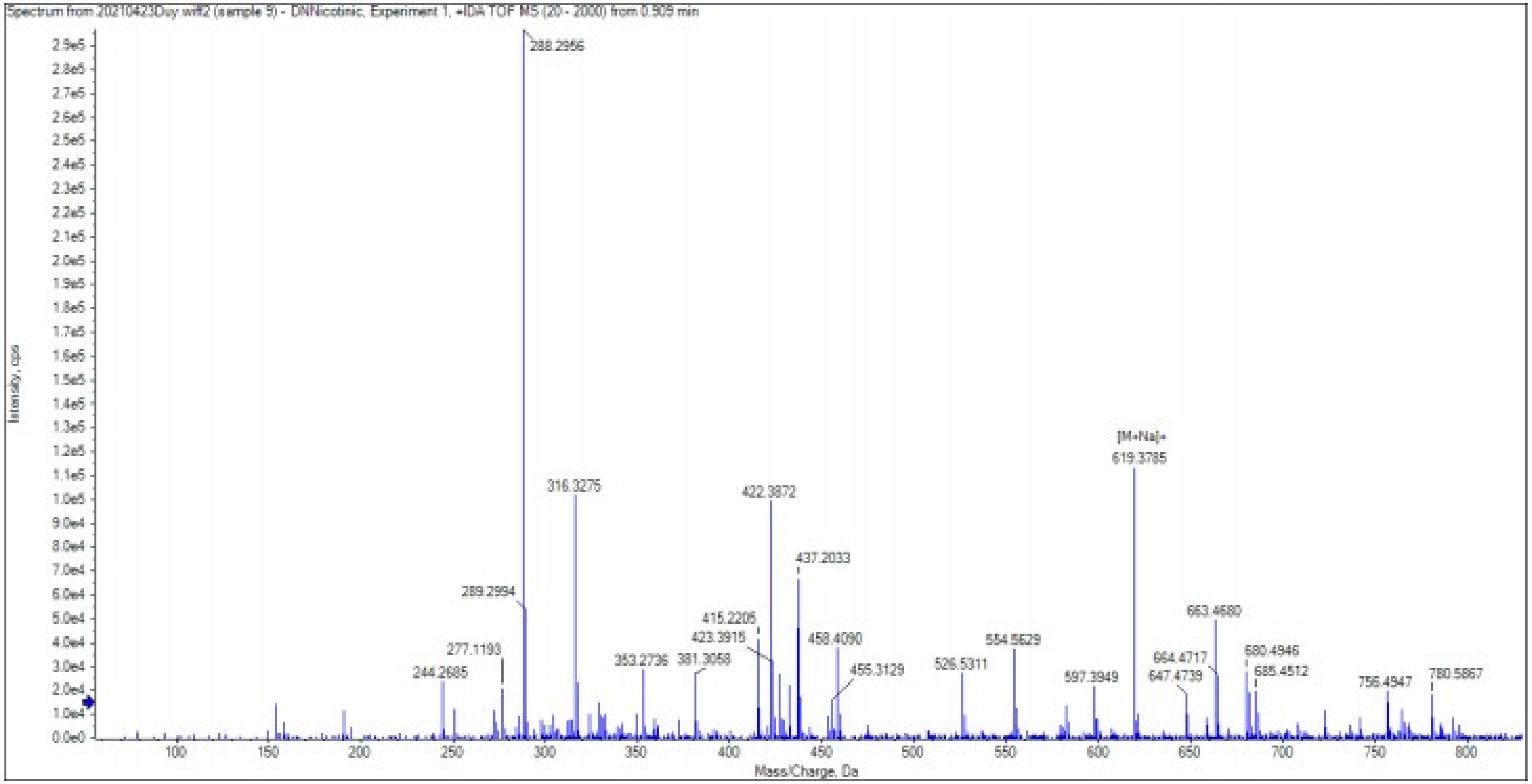


| Hit | Formula | m/z | RDB | ppm | MS Rank | MSMS ppm | MSMS Rank | Found |
|---|---|---|---|---|---|---|---|---|
| 1 | C36H53ClN2O3 | 619.3759 | 11.0 | 3.9 | 1 | | | NA/NA |

(+)-HR-ESI-MS spectrum of compound **3e**

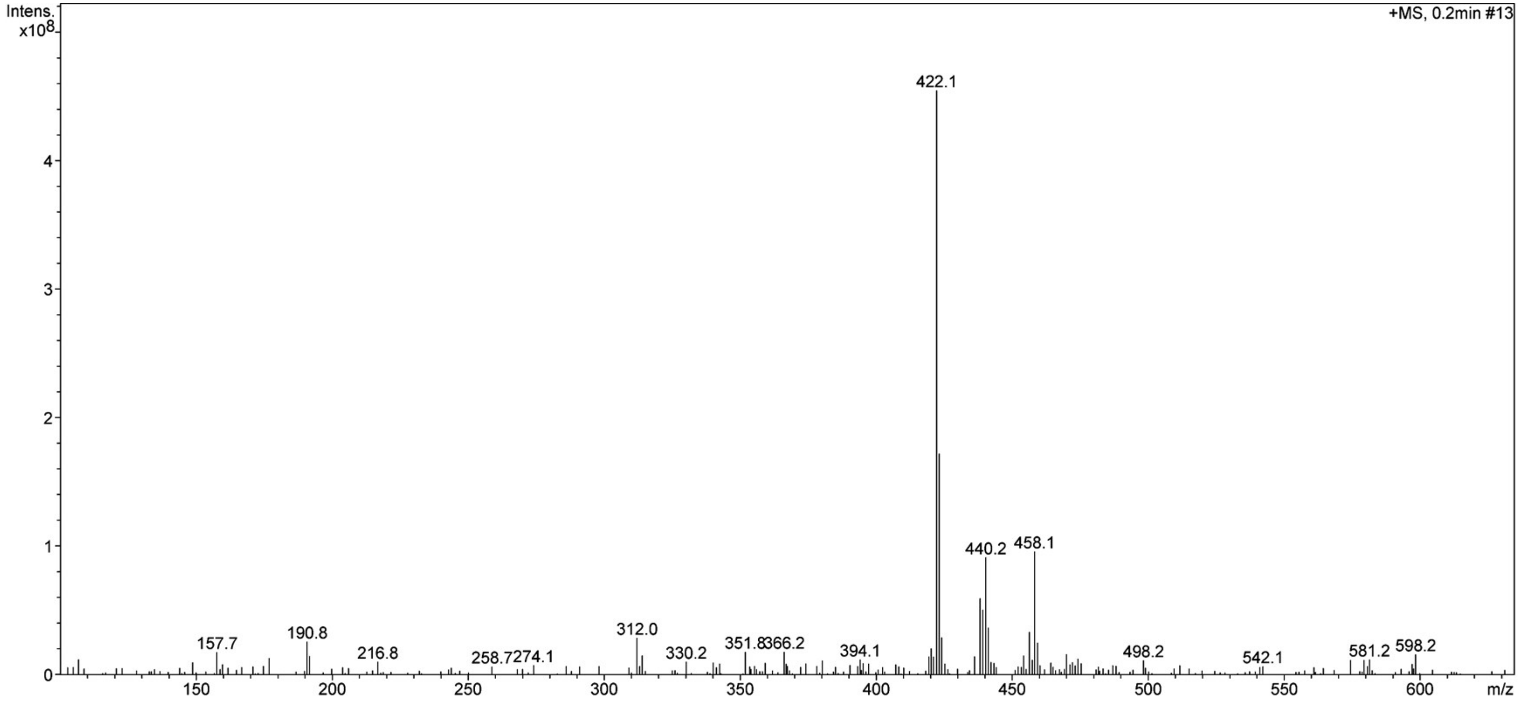


(+)-ESI–MS spectrum of compound **3e**

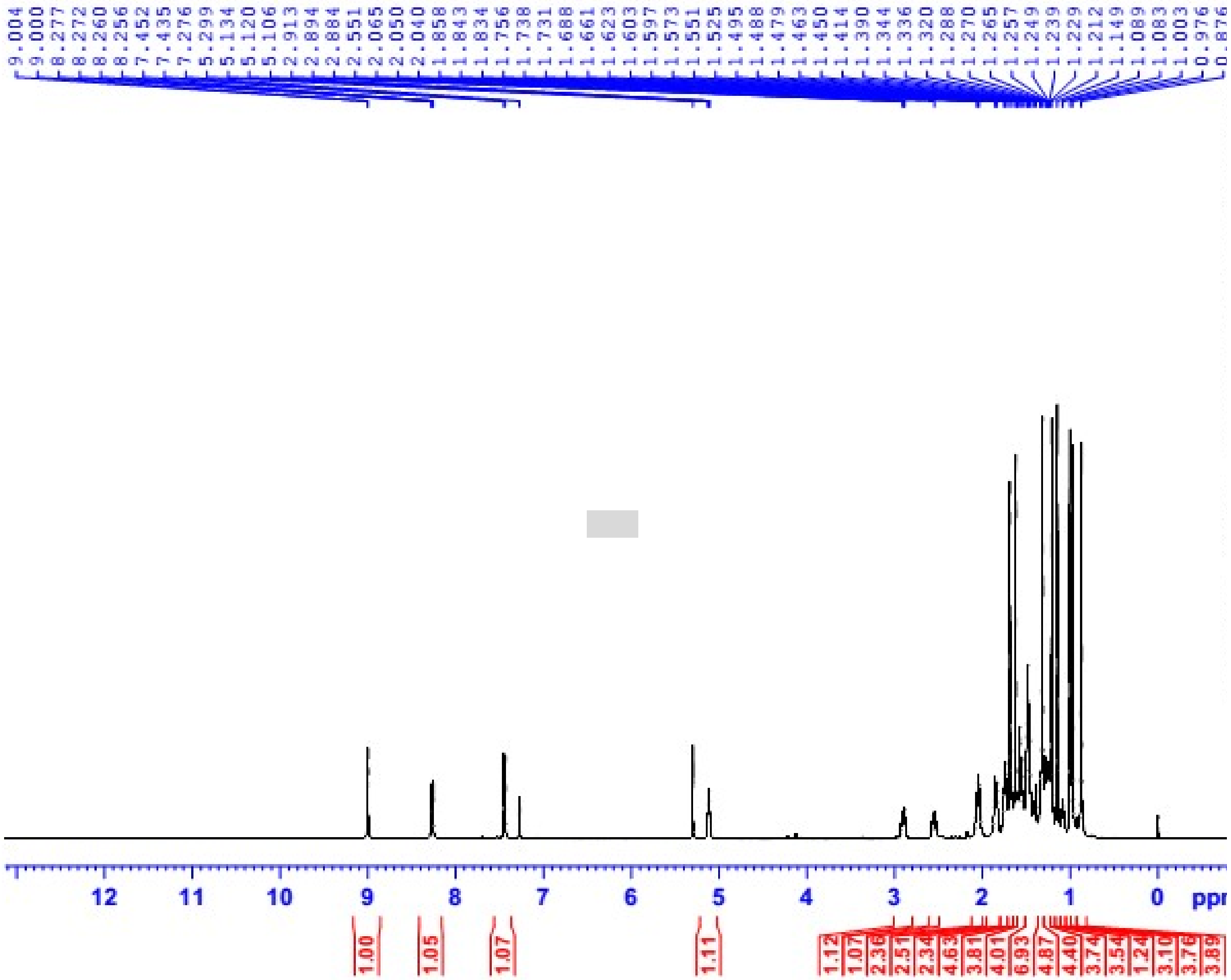

$^{1}$H-NMR spectrum of compound **3e**

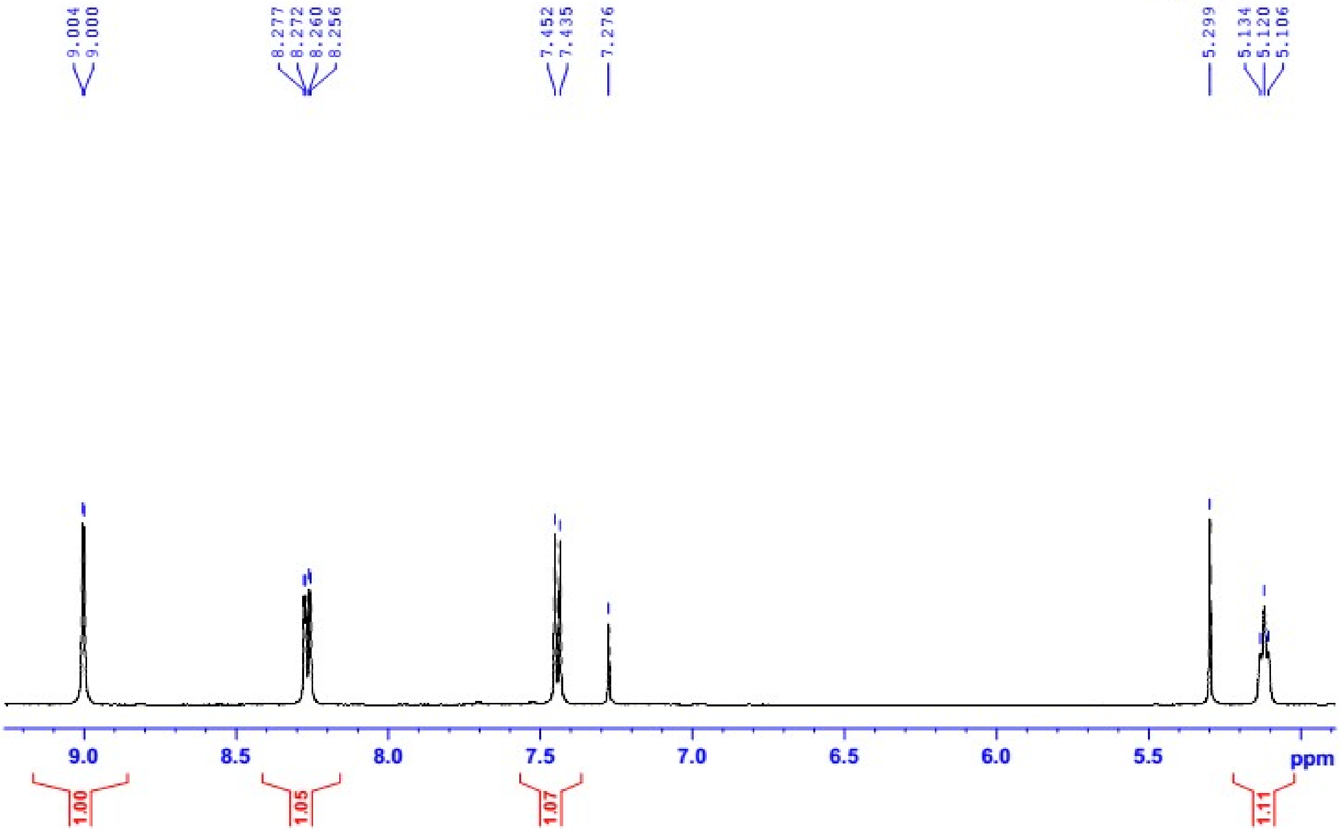


$^{1}$H-NMR spectrum of compound **3e** (extension)

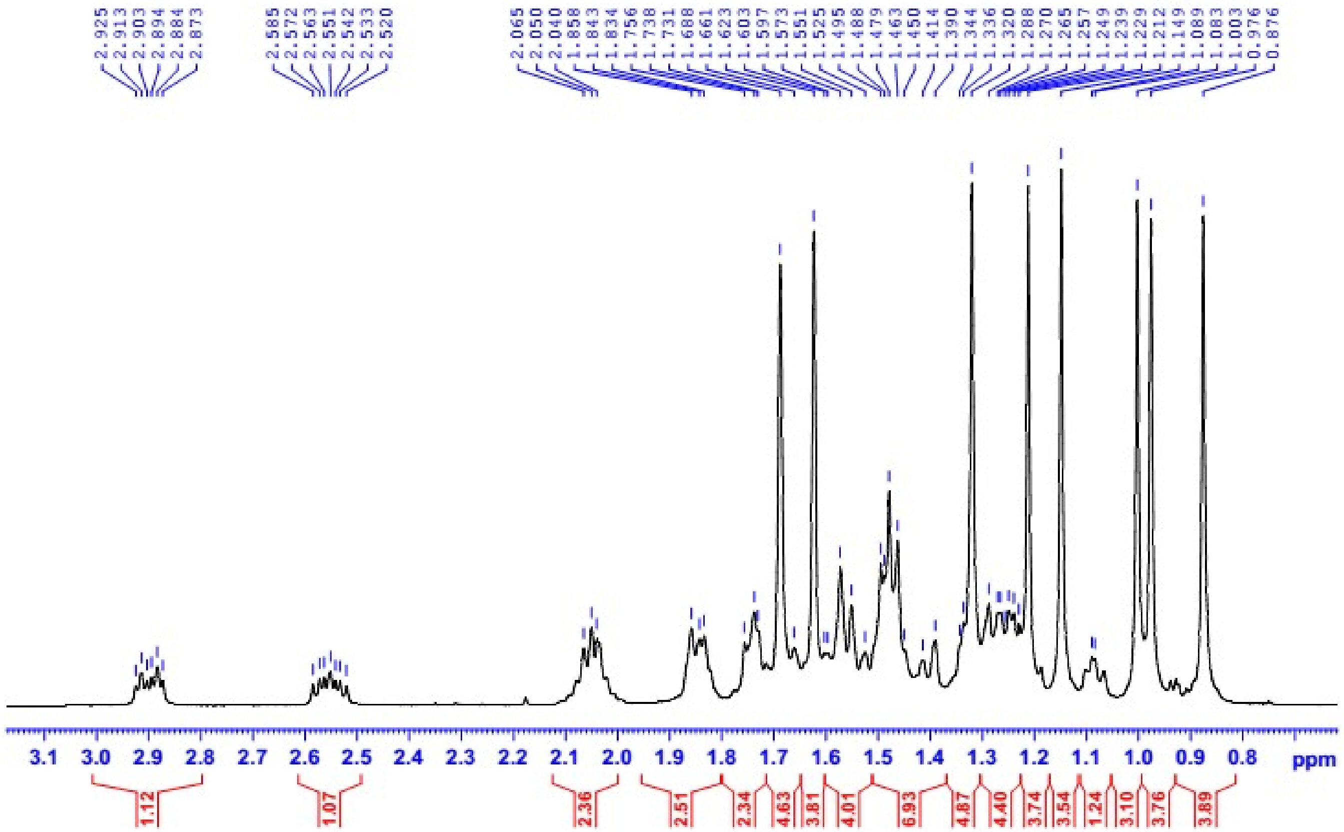

$^{1}$H-NMR spectrum of compound **3e** (extension)

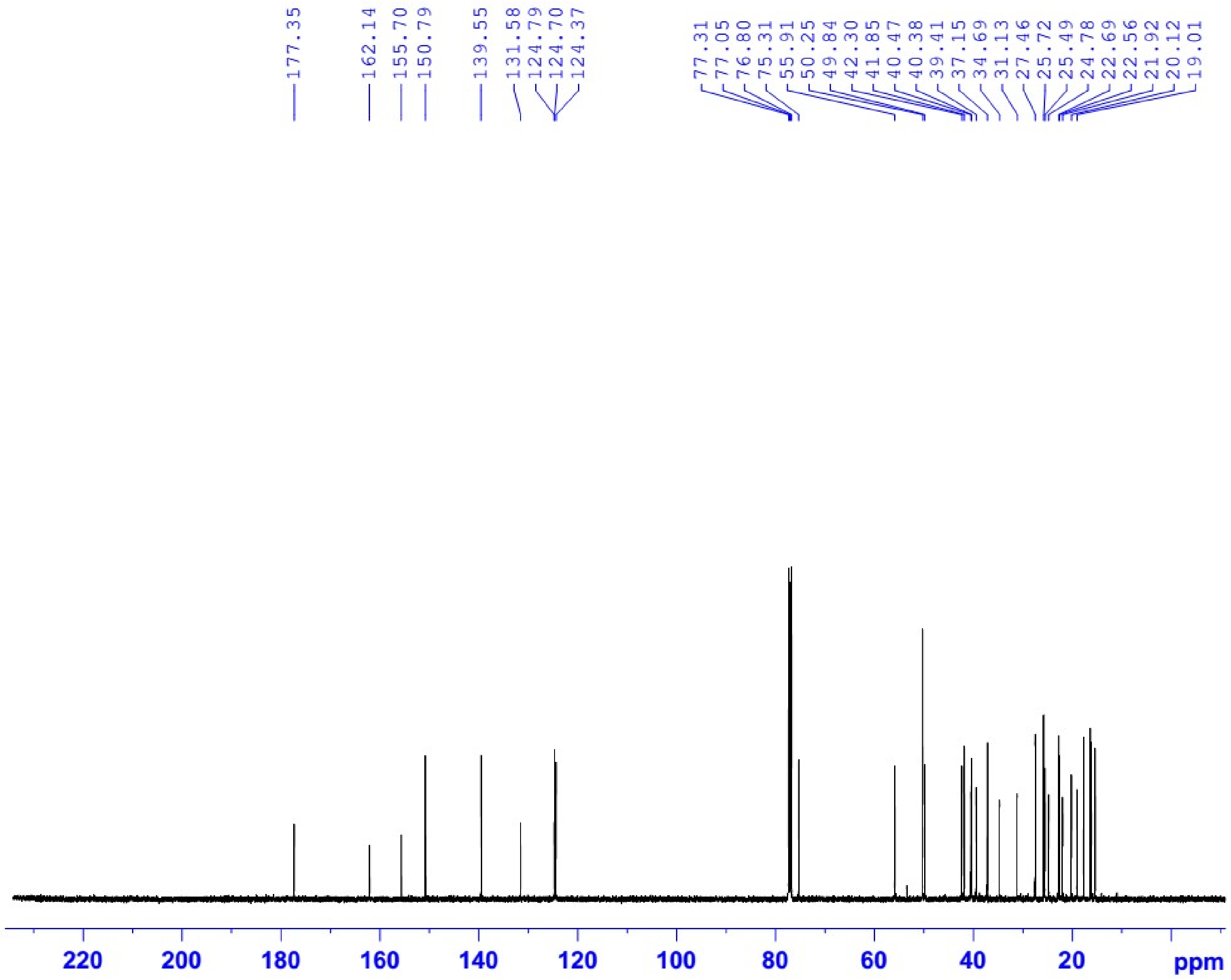


$^{13}$C-NMR spectrum of compound **3e**

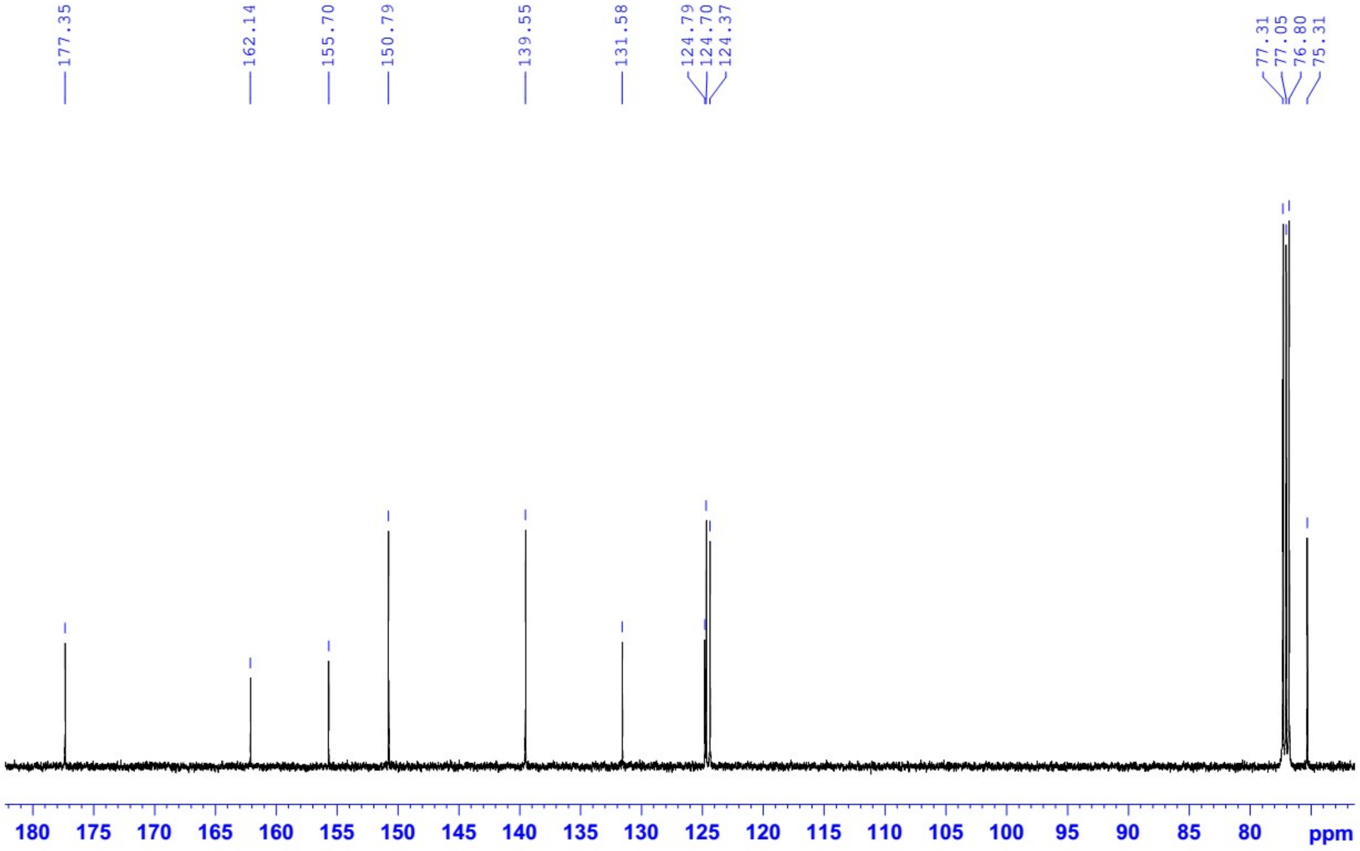


$^{13}$C-NMR spectrum of compound **3e** (extension)

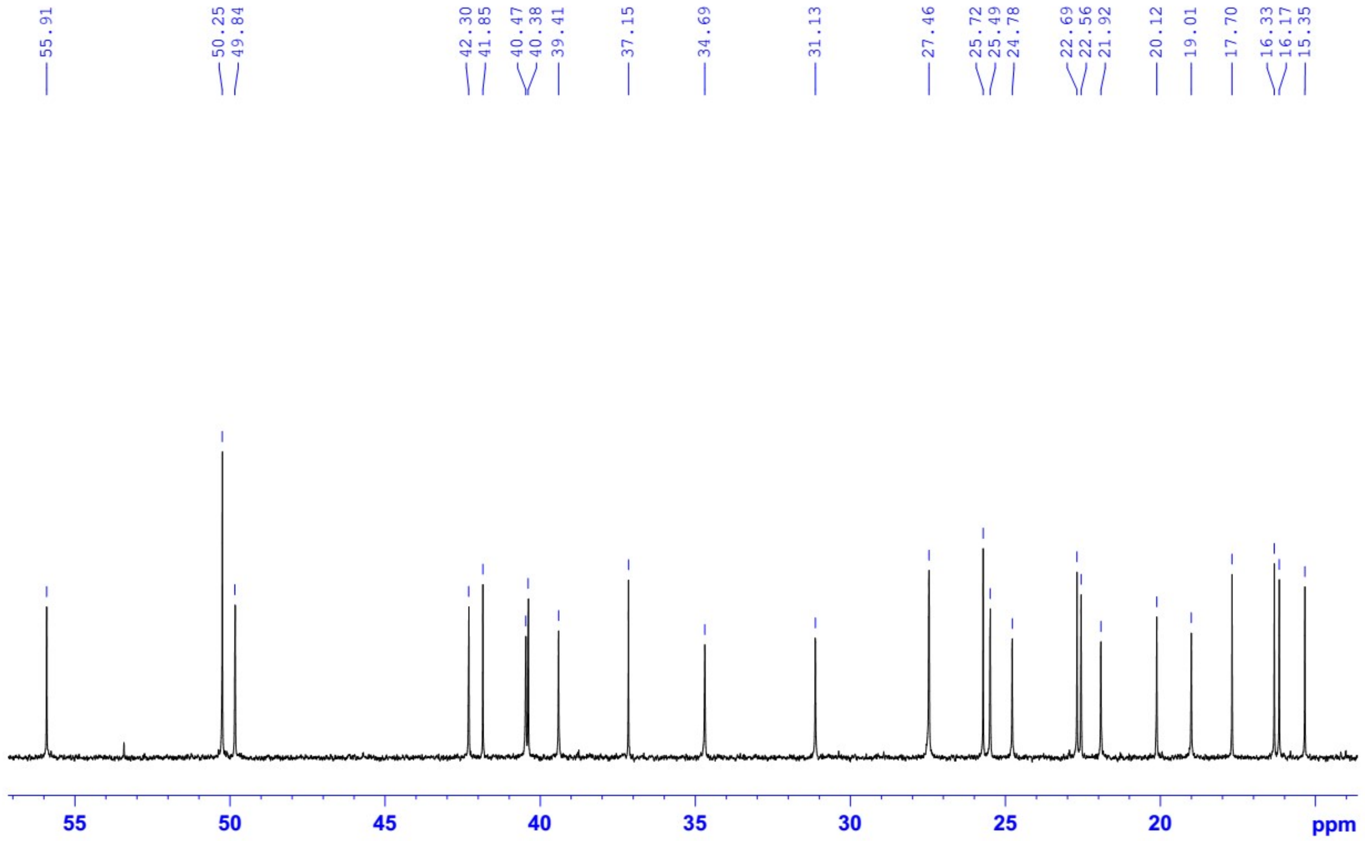


$^{13}$C-NMR spectrum of compound **3e** (extension)

## 1.8. Compound 3f

**Sample name: DN4Cla**
**Operator: Le Anh VHH**
**Method: -IDA TOF MS/MS**
**Date: 2021.04.23**

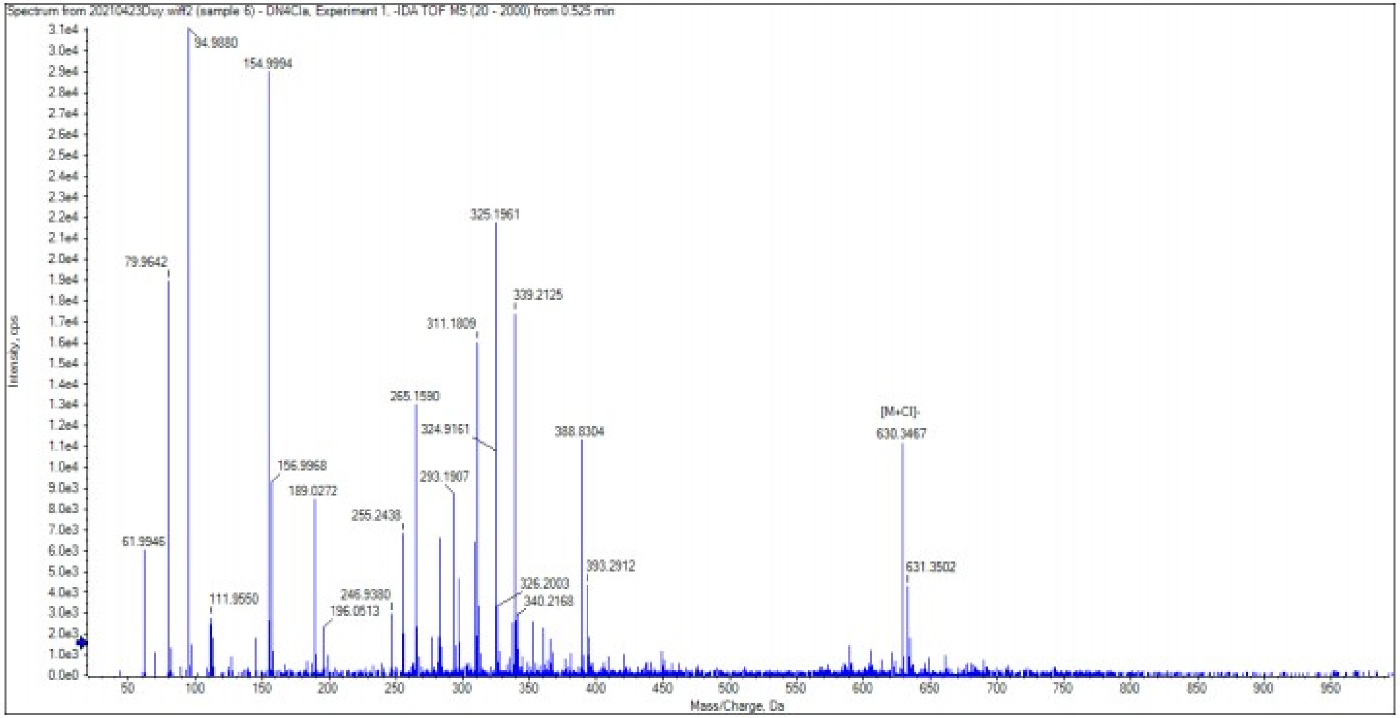


| Hit | Formula | m/z | RDB | ppm | MS Rank | MSMS ppm | MSMS Rank | Found |
|---|---|---|---|---|---|---|---|---|
| 1 | C37H54ClNO3 | 630.34862 | 10.0 | -1.2 | 1 | | | NA/NA |

(-)-HR-ESI-MS spectrum of compound **3f**

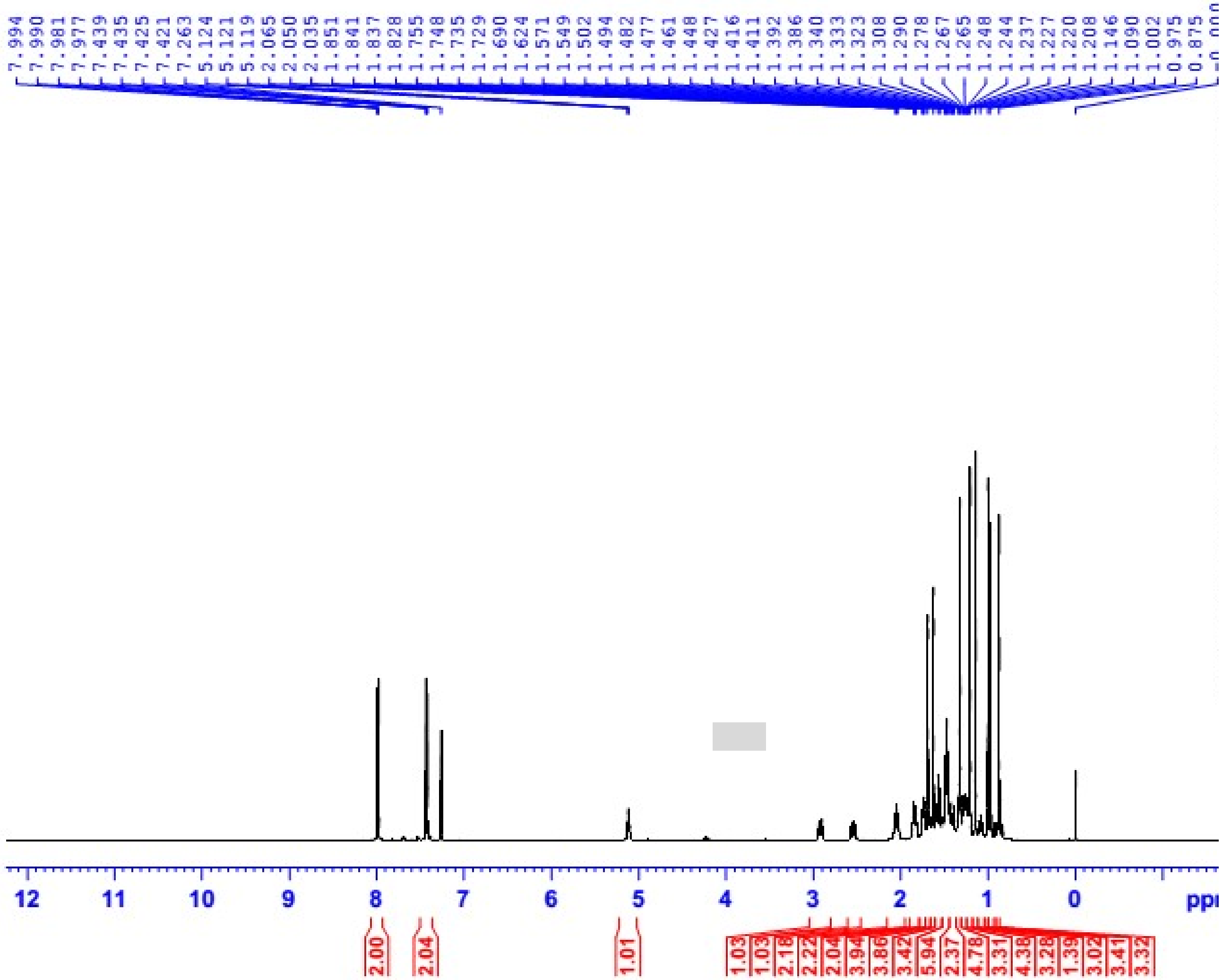

$^{1}$H-NMR spectrum of compound **3f**

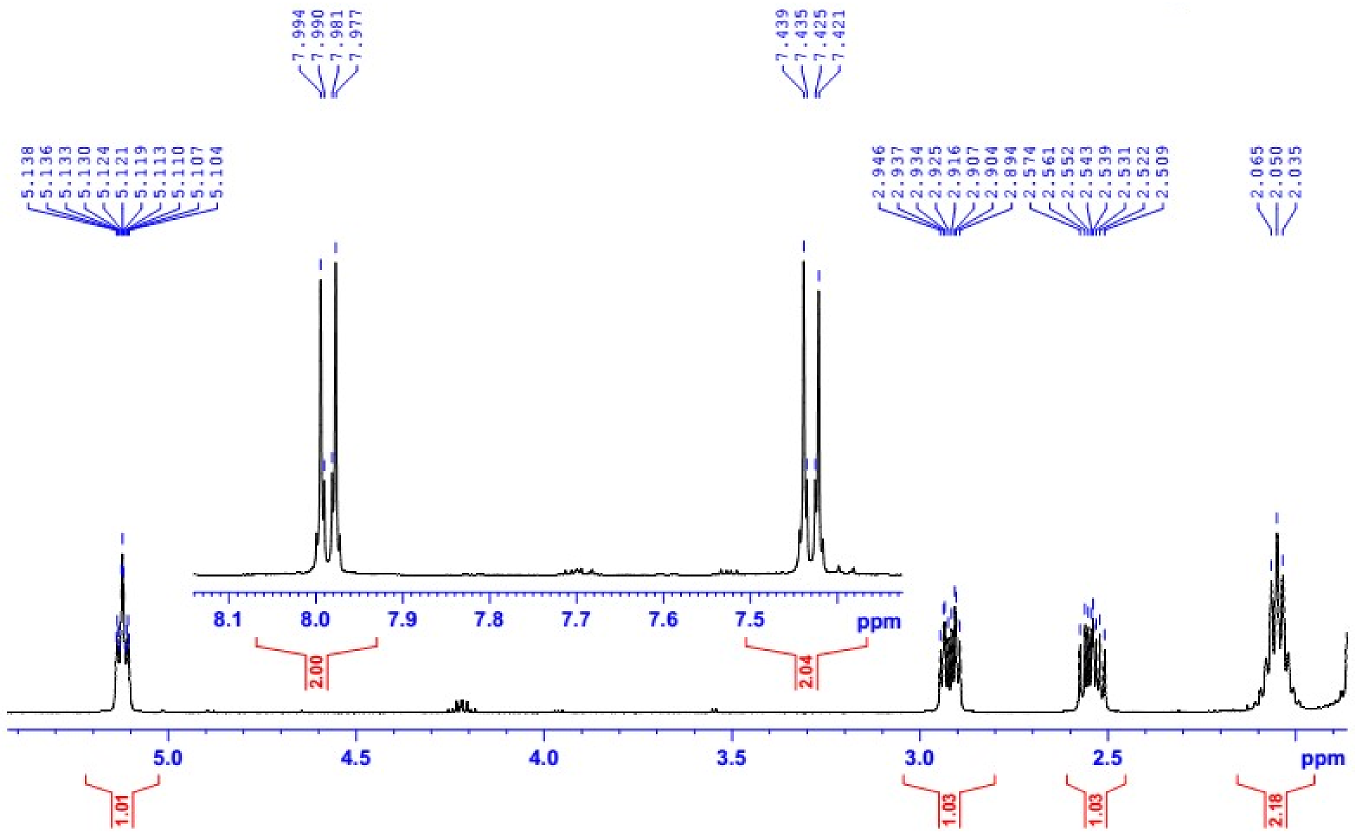

$^{1}$H-NMR spectrum of compound **3f** (extension)

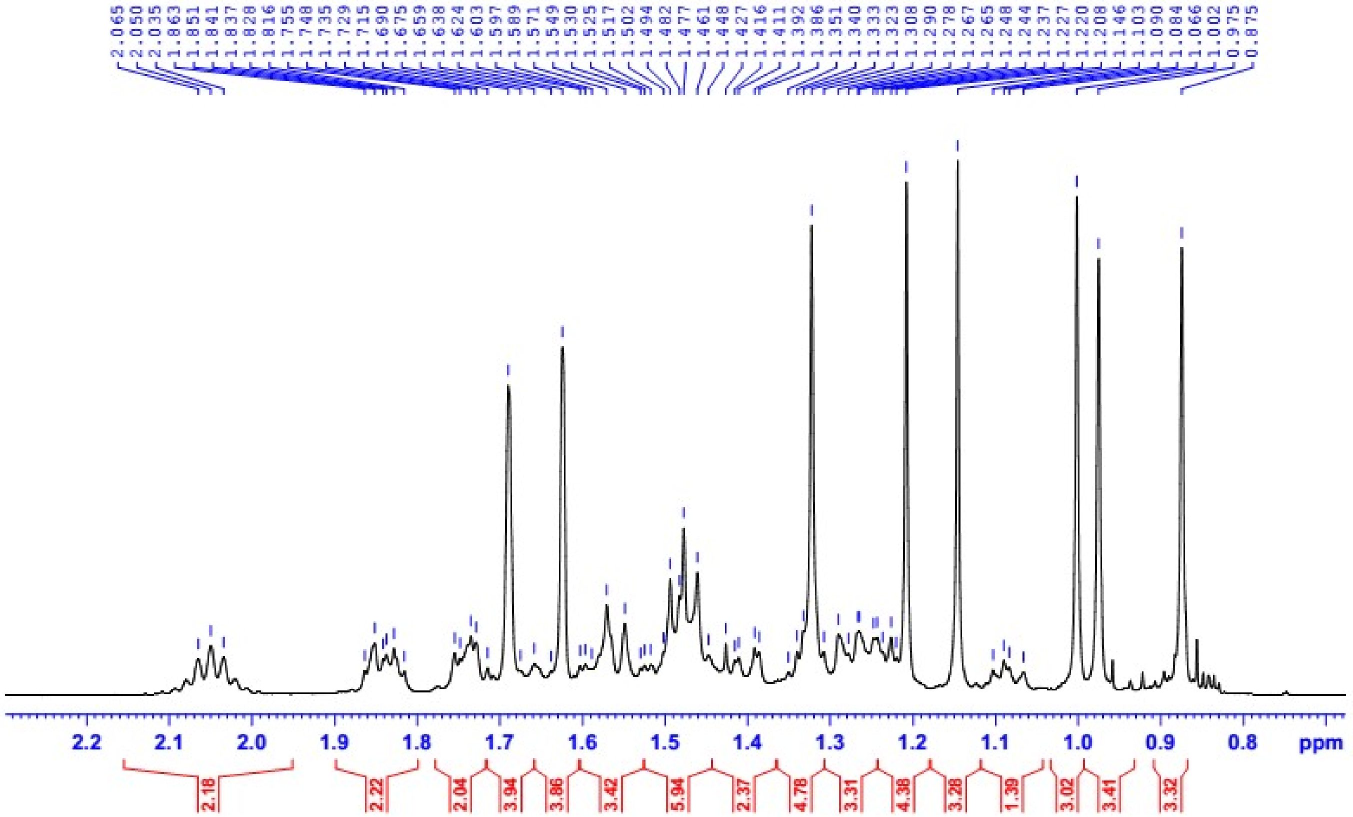

$^{1}$H-NMR spectrum of compound **3f** (extension)

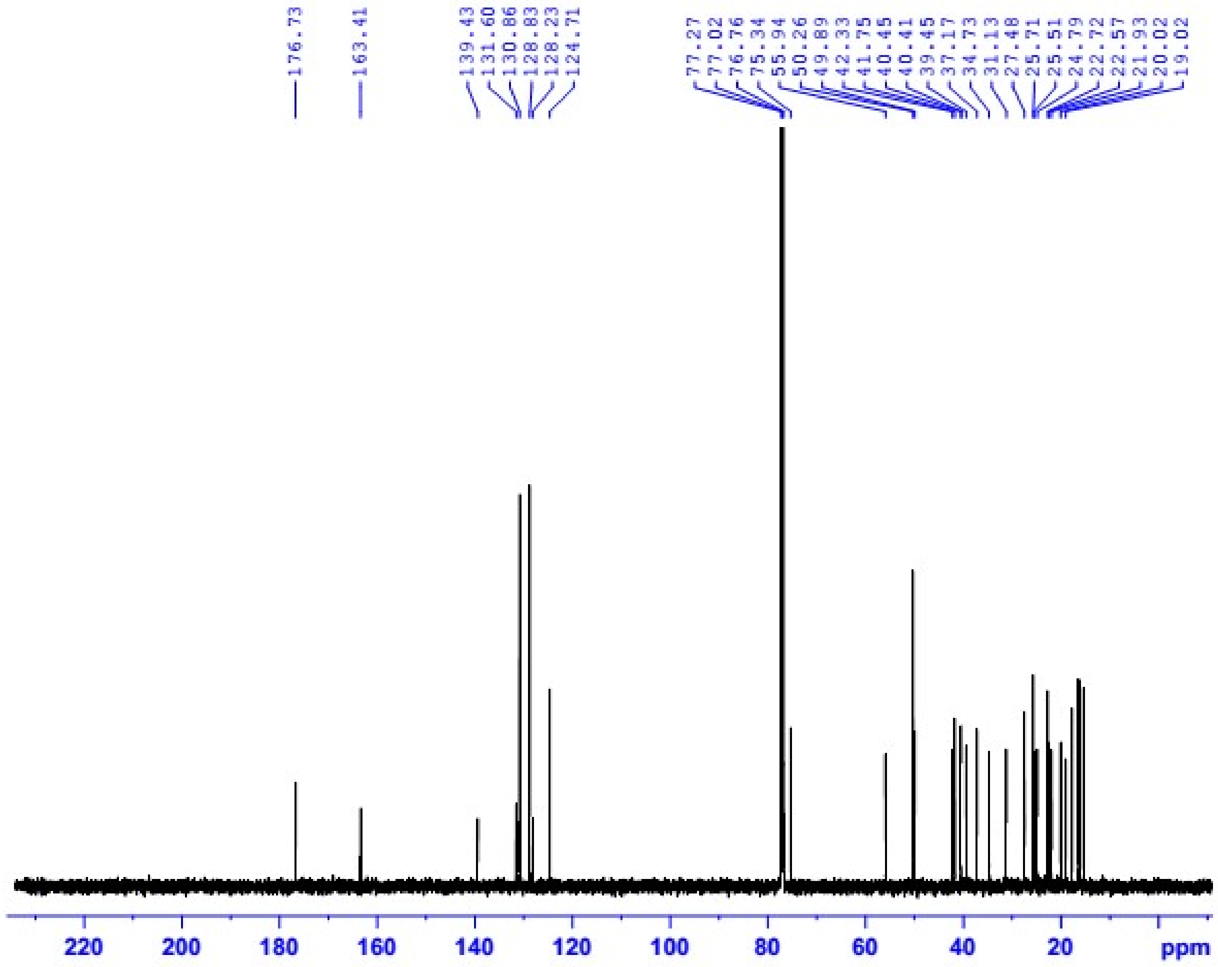


$^{13}$C-NMR spectrum of compound **3f**

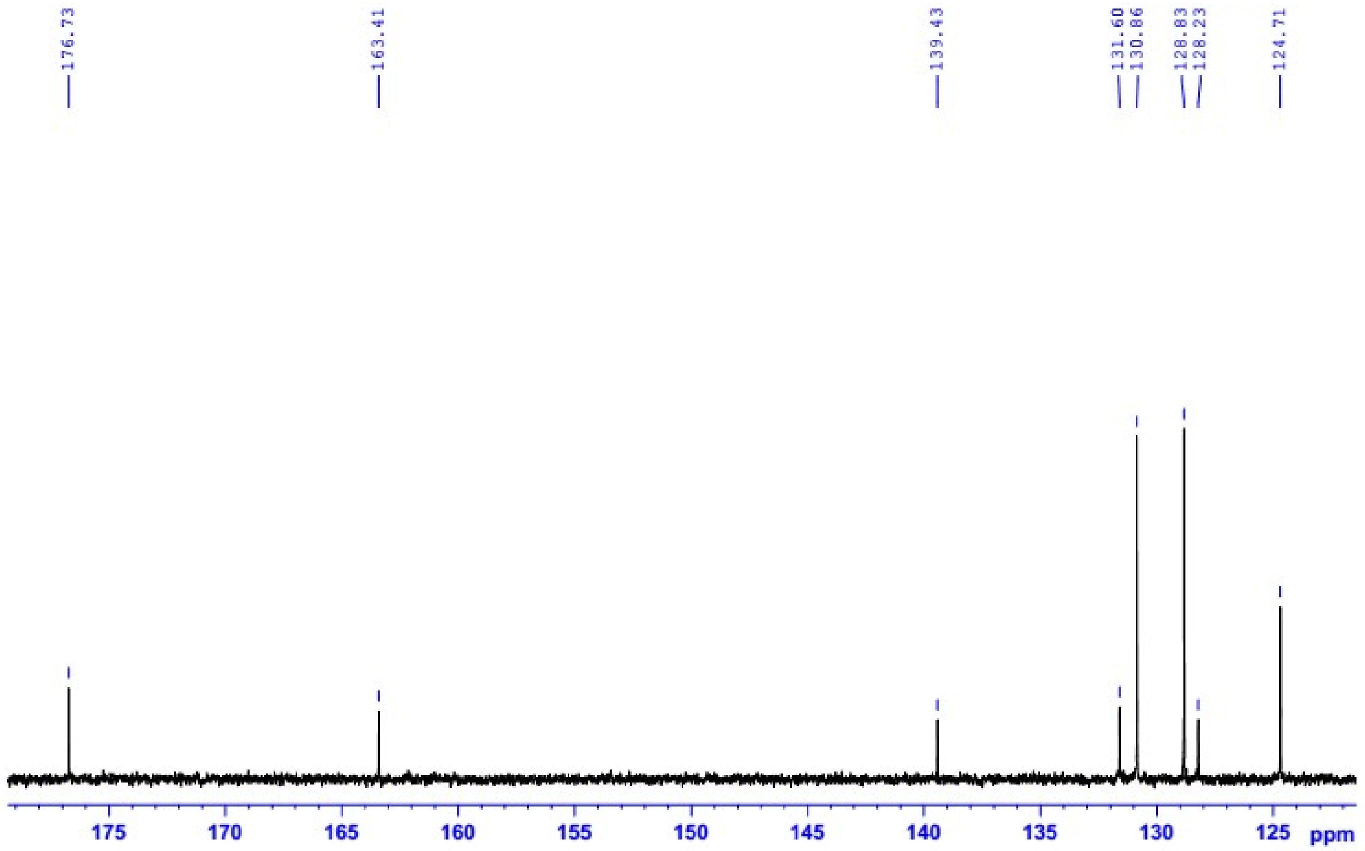


$^{13}$C-NMR spectrum of compound **3f** (extension)

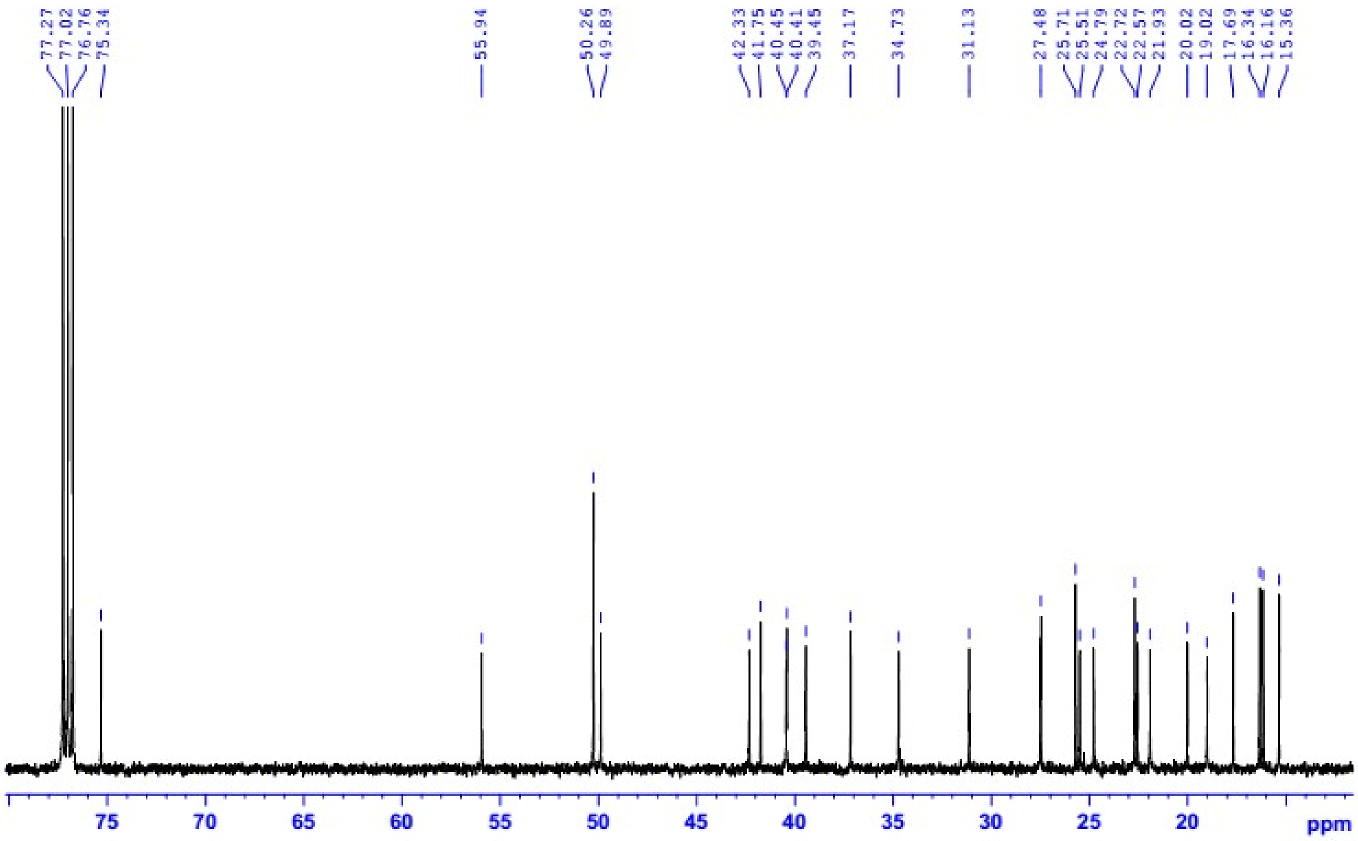


$^{13}$C-NMR spectrum of compound **3f** (extension)

### 1.9. Compound 3g

***Sample name: DN4F***
***Operator: Le Anh VHH***
***Method: +IDA TOF MS/MS***
***Date: 2021.04.23***

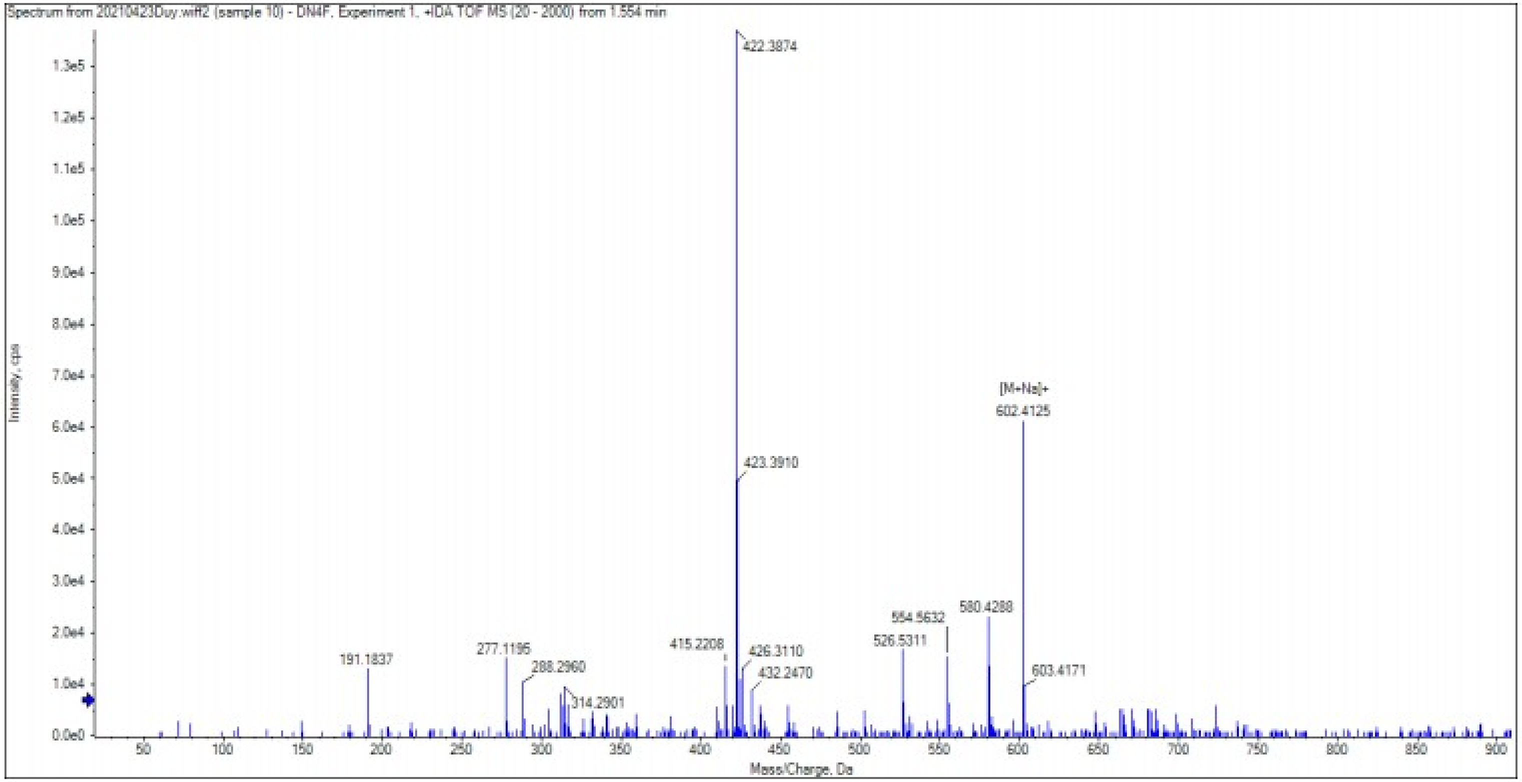


| Hit | Formula | m/z | RDB | ppm | MS Rank | MSMS ppm | MSMS Rank | Found |
|---|---|---|---|---|---|---|---|---|
| 1 | C37H54FNO3 | 602.40979 | 11.0 | 4.0 | 1 | | | NA/NA |

(+)-HR-ESI-MS spectrum of compound **3g**

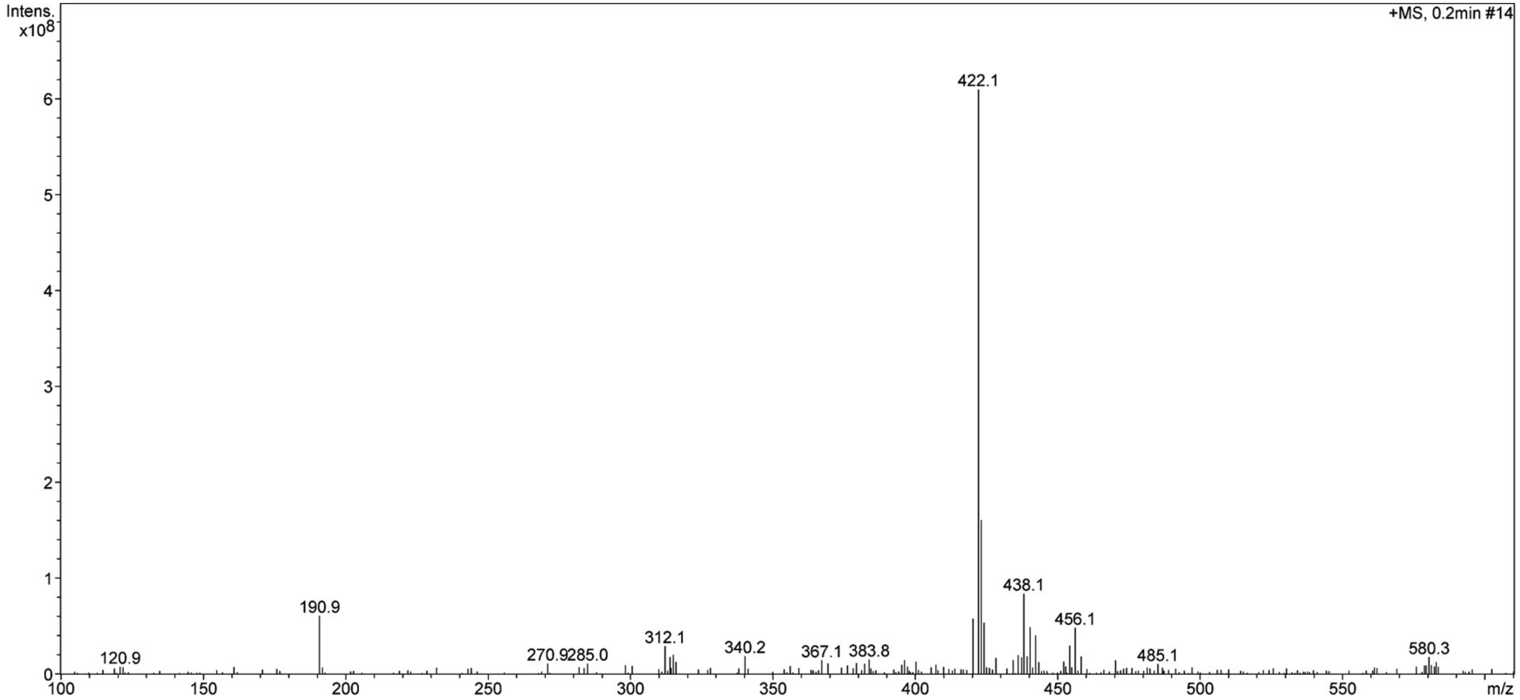

(+)-ESI–MS spectrum of compound **3g**

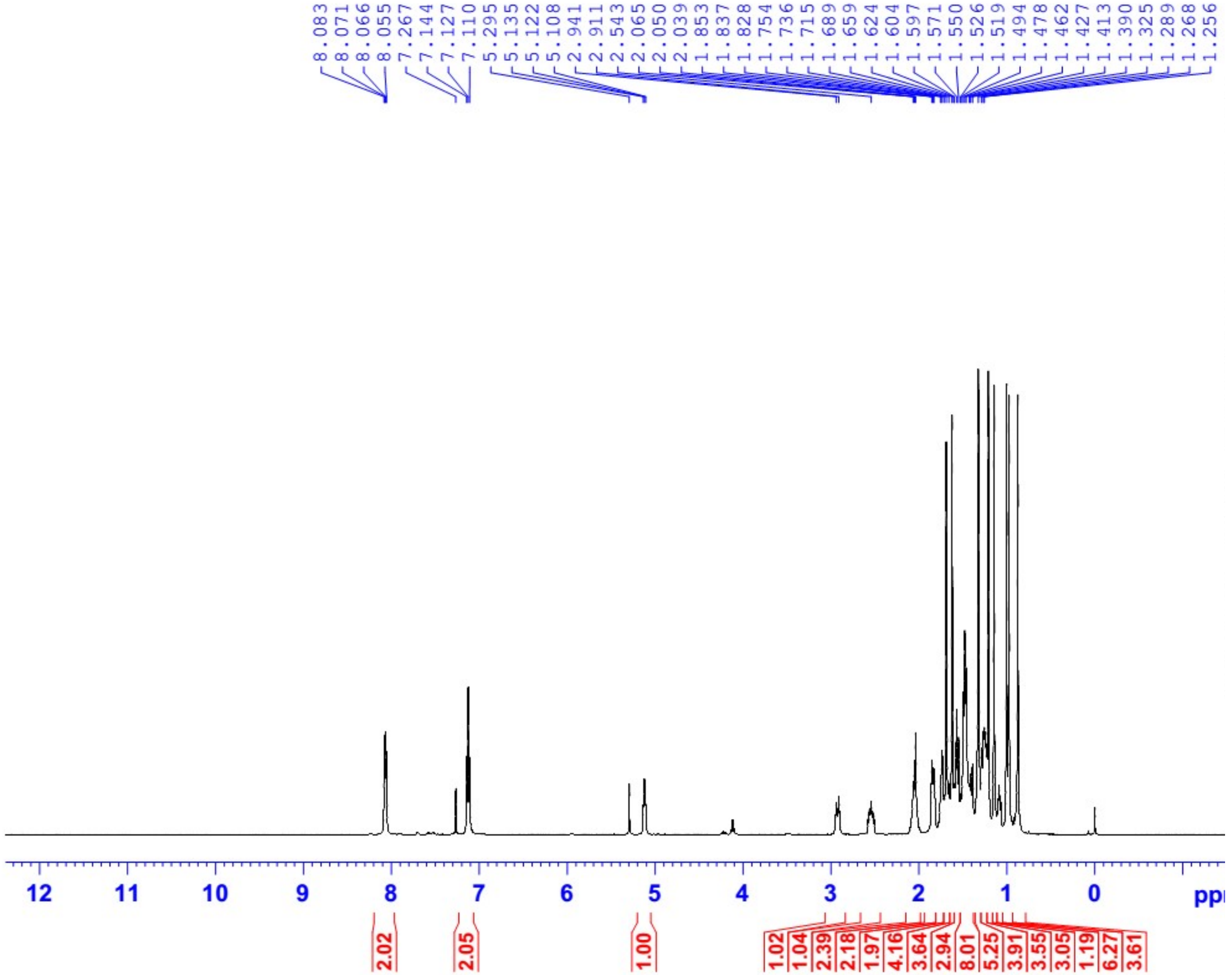


$^{1}$H-NMR spectrum of compound **3g**

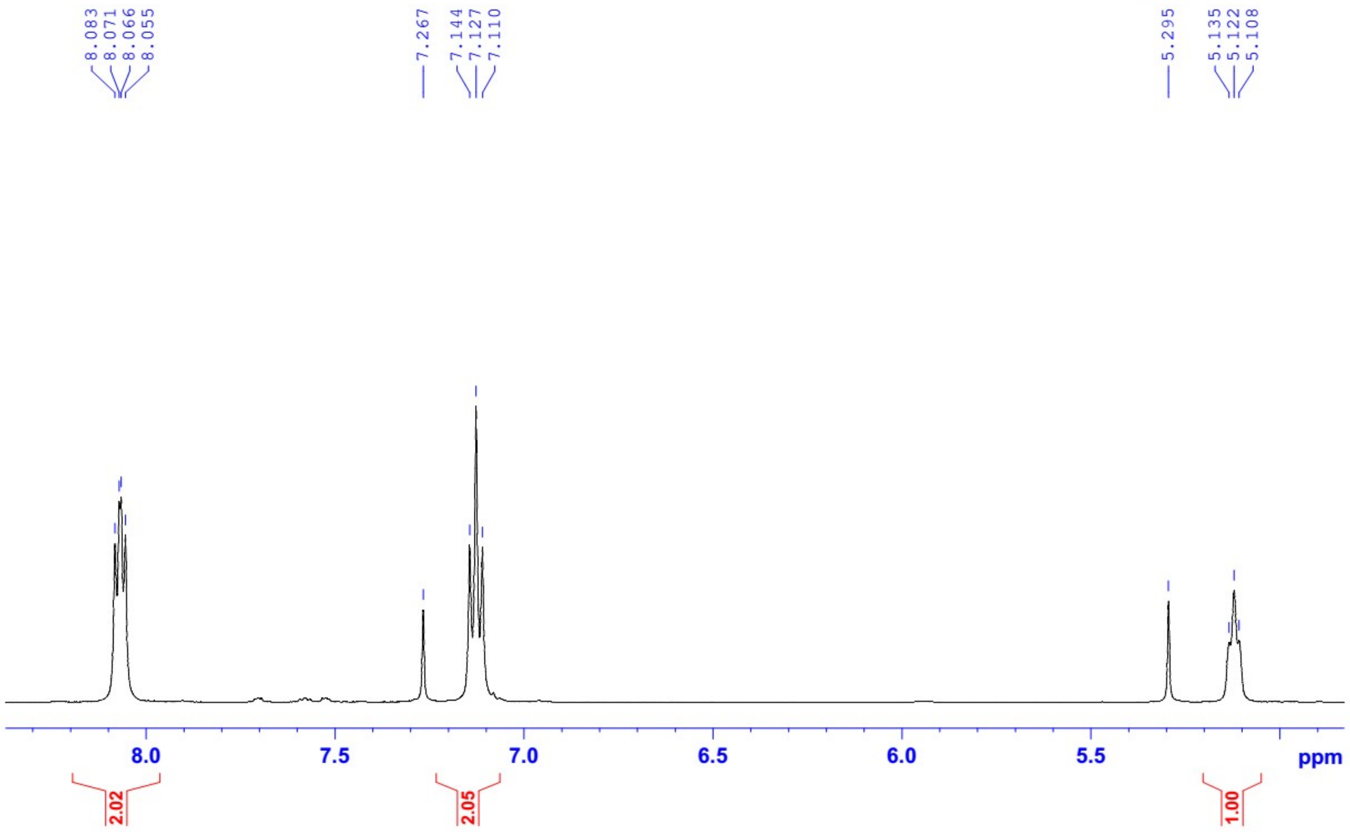


$^{1}$H-NMR spectrum of compound **3g** (extension)

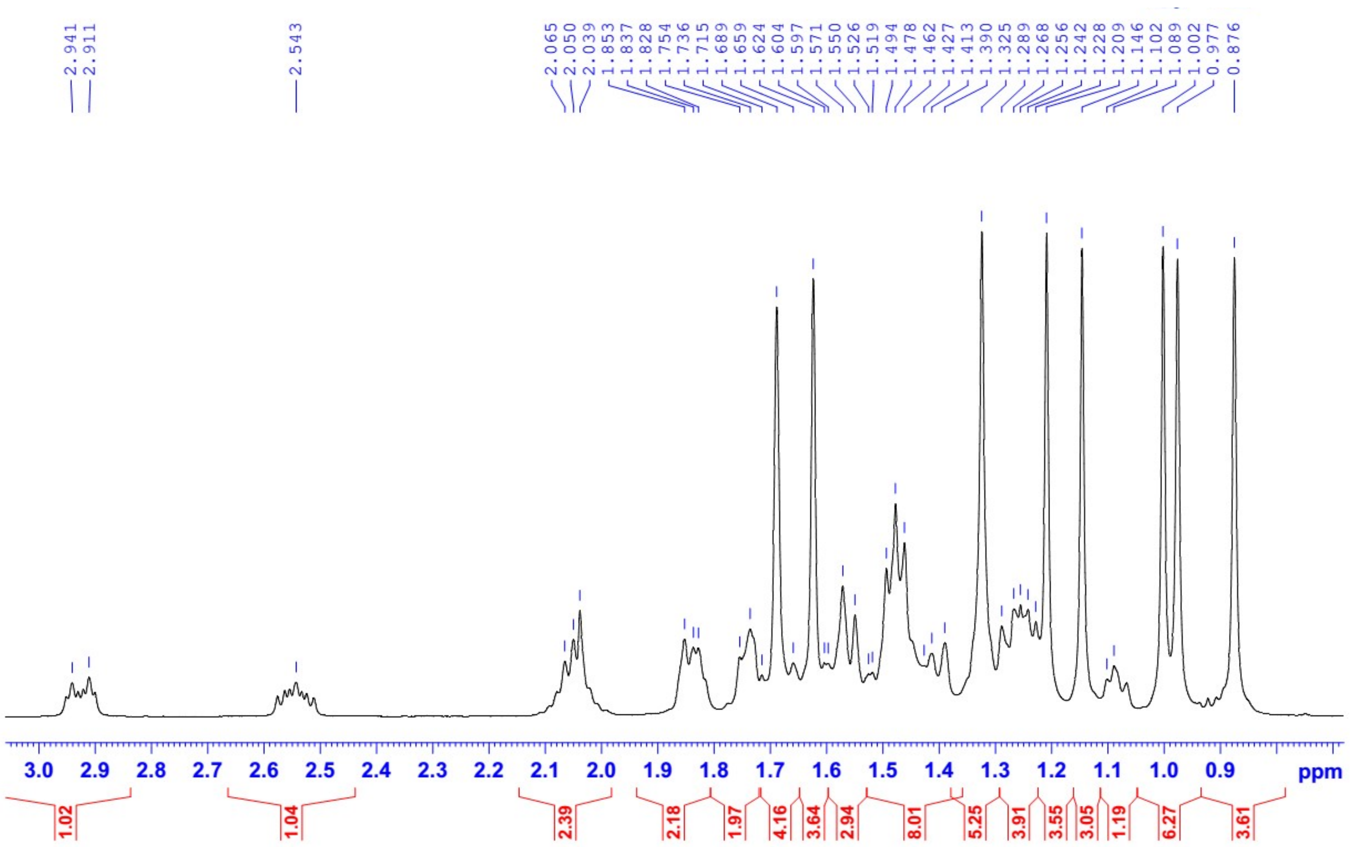

$^{1}$H-NMR spectrum of compound **3g** (extension)

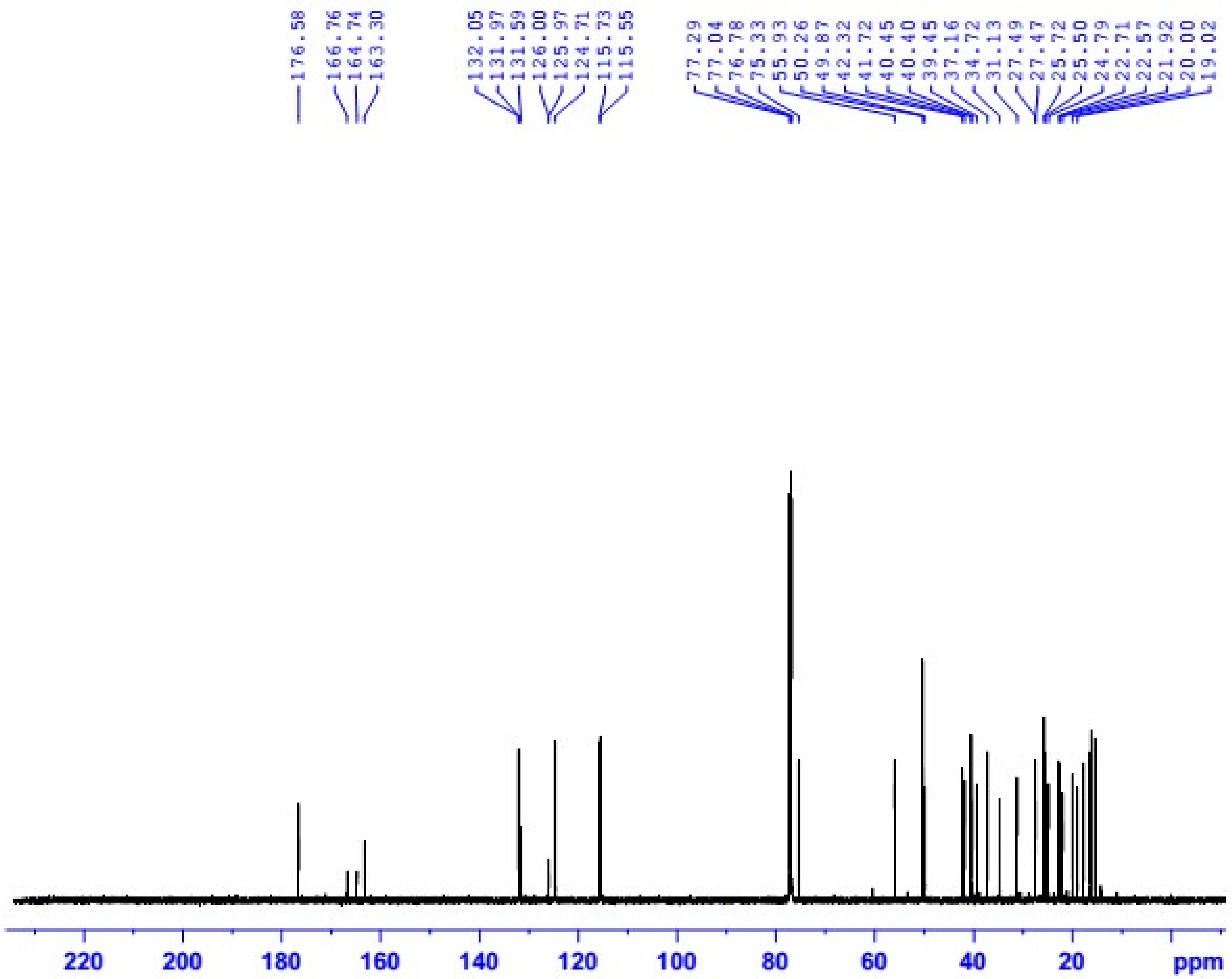


$^{13}$C-NMR spectrum of compound **3g**

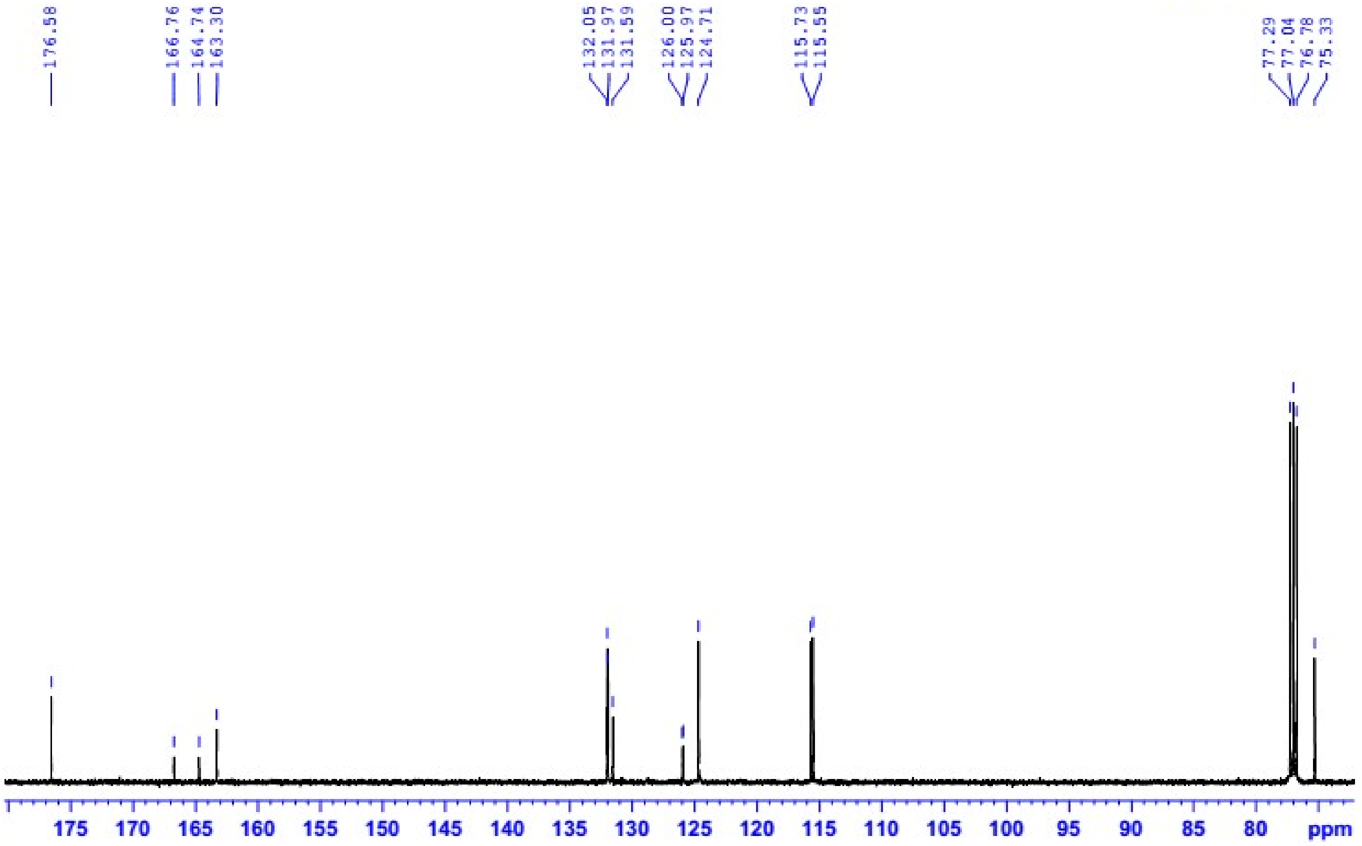


$^{13}$C-NMR spectrum of compound **3g** (extension)

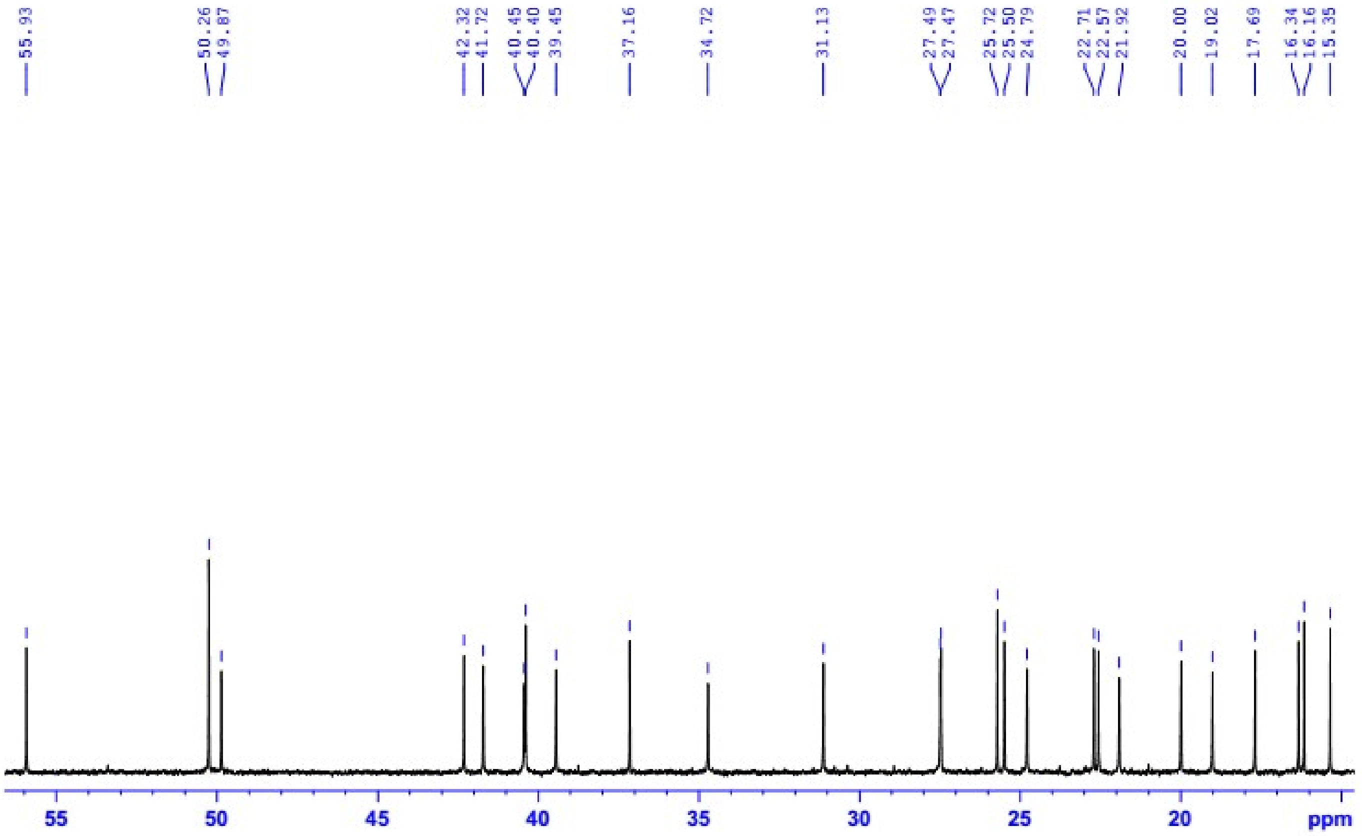


$^{13}$C-NMR spectrum of compound **3g** (extension)

## 1.10. Compound 3h

**Sample name: DNOIa**
**Operator: Le Anh VHH**
**Method: +IDA TOF MS/MS**
**Date: 2021.04.23**

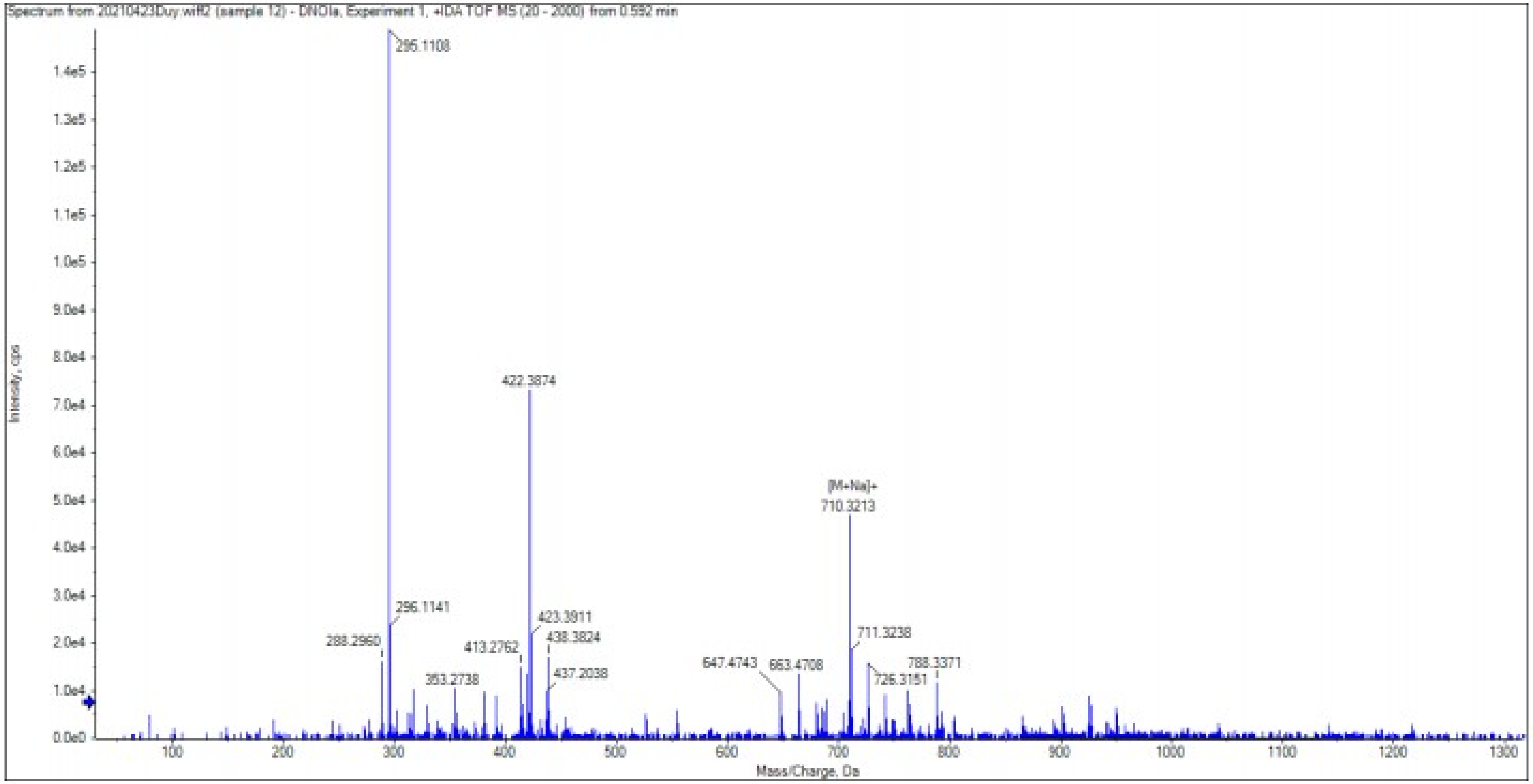


| Hit | Formula | m/z | RDB | ppm | MS Rank | MSMS ppm | MSMS Rank | Found |
|---|---|---|---|---|---|---|---|---|
| 1 | C37H54INO3 | 710.31706 | 11.0 | 4.3 | 1 | | | NA/NA |

(+)-HR-ESI-MS spectrum of compound **3h**

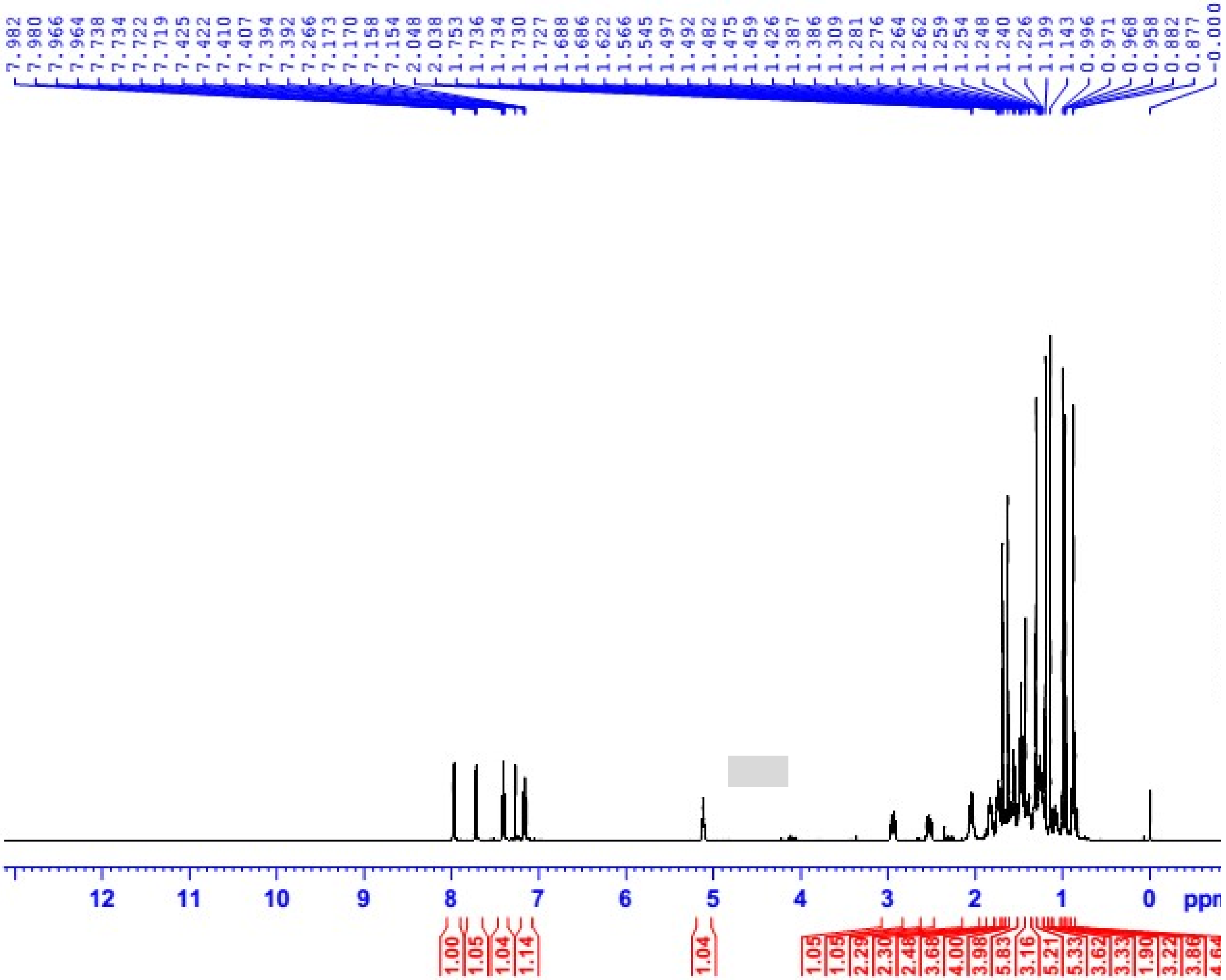

$^{1}$H-NMR spectrum of compound **3h**

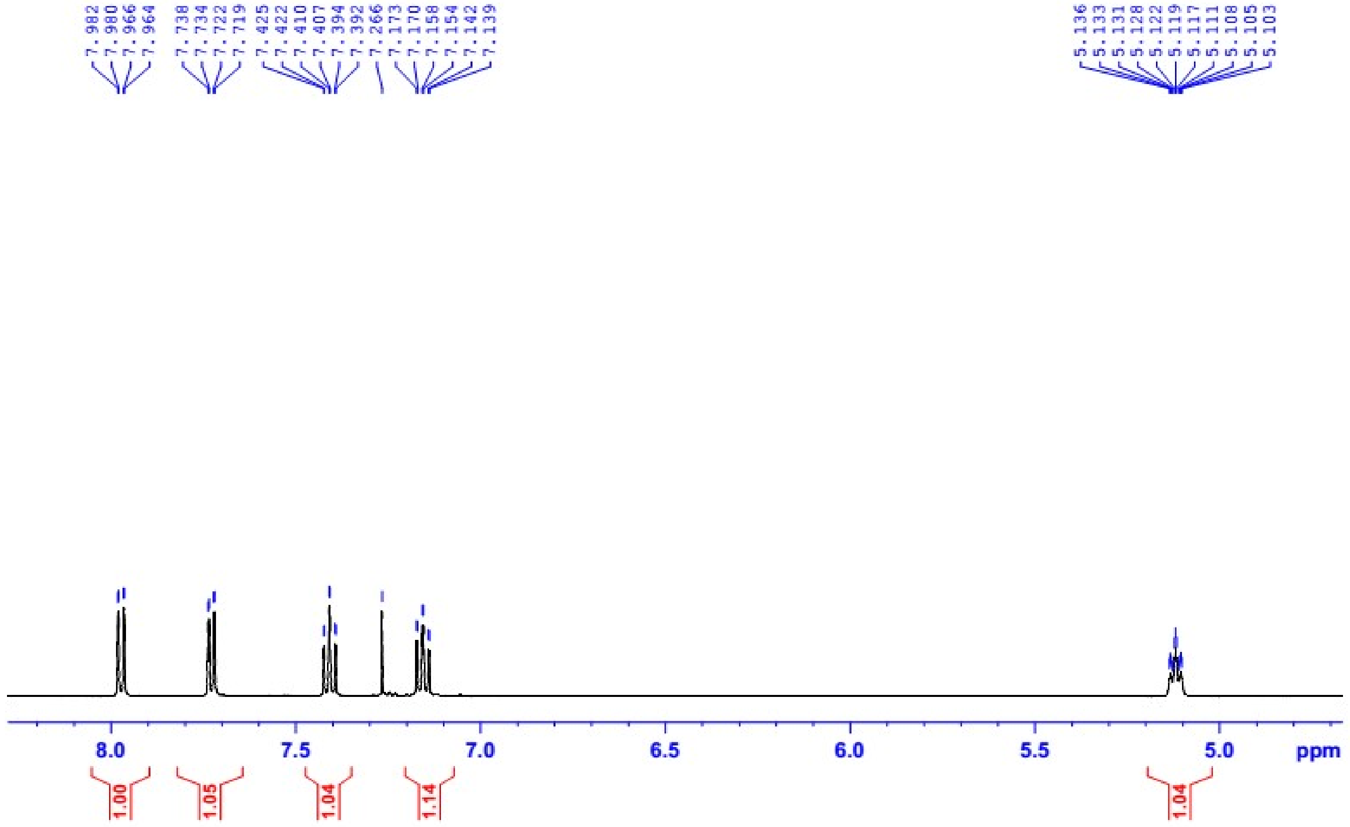


$^{1}$H-NMR spectrum of compound **3h** (extension)

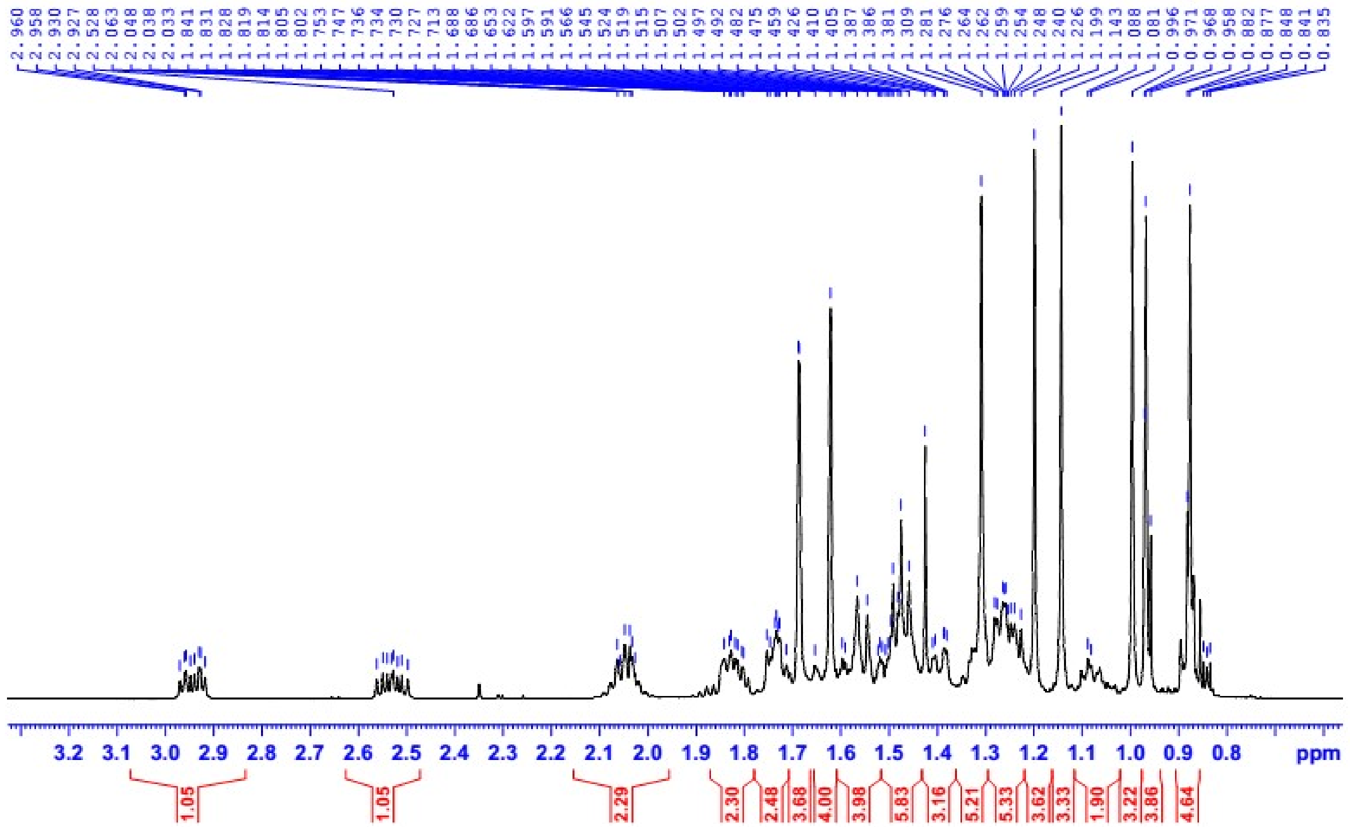

$^{1}$H-NMR spectrum of compound **3h** (extension)

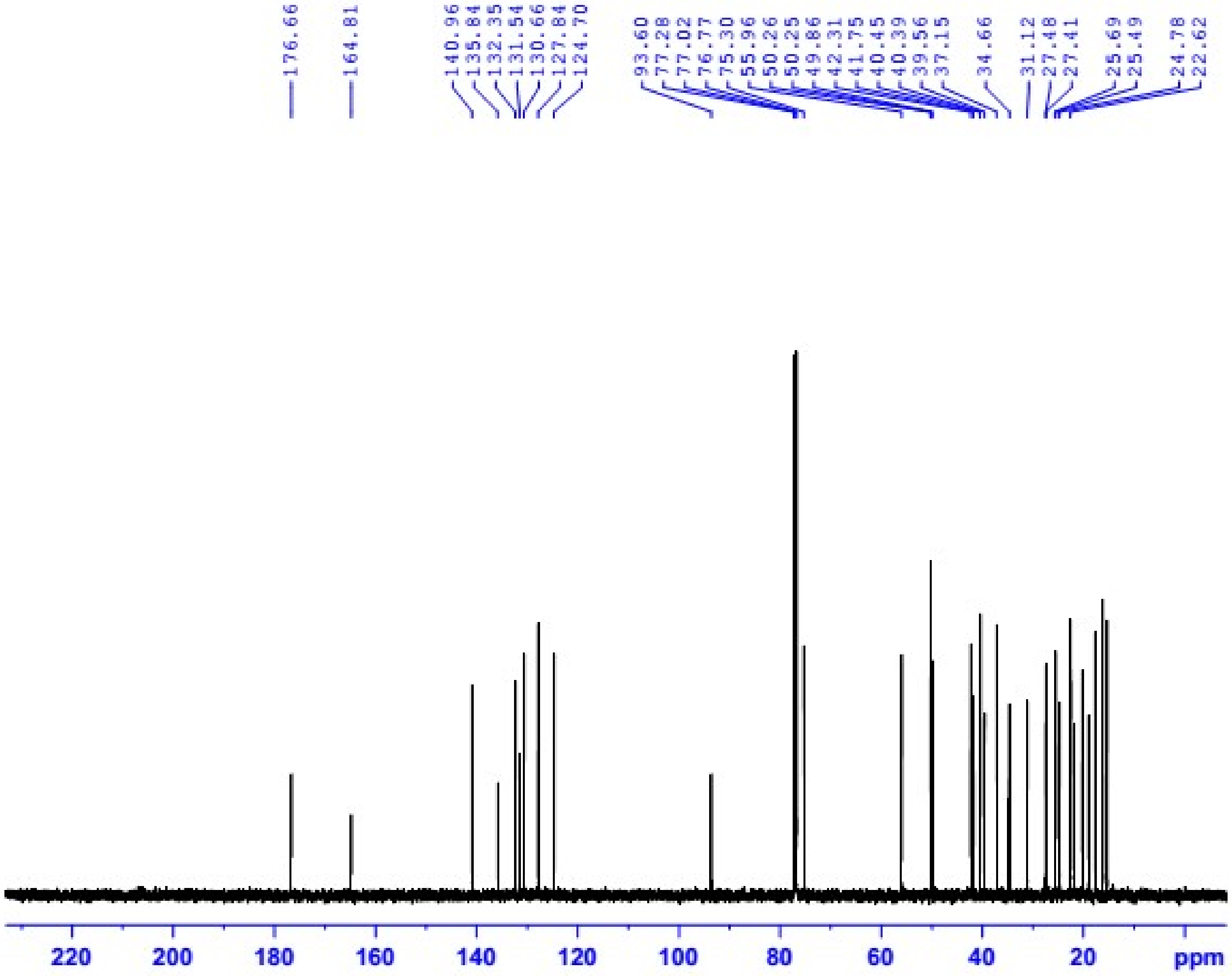


$^{13}C$-NMR spectrum of compound **3h**

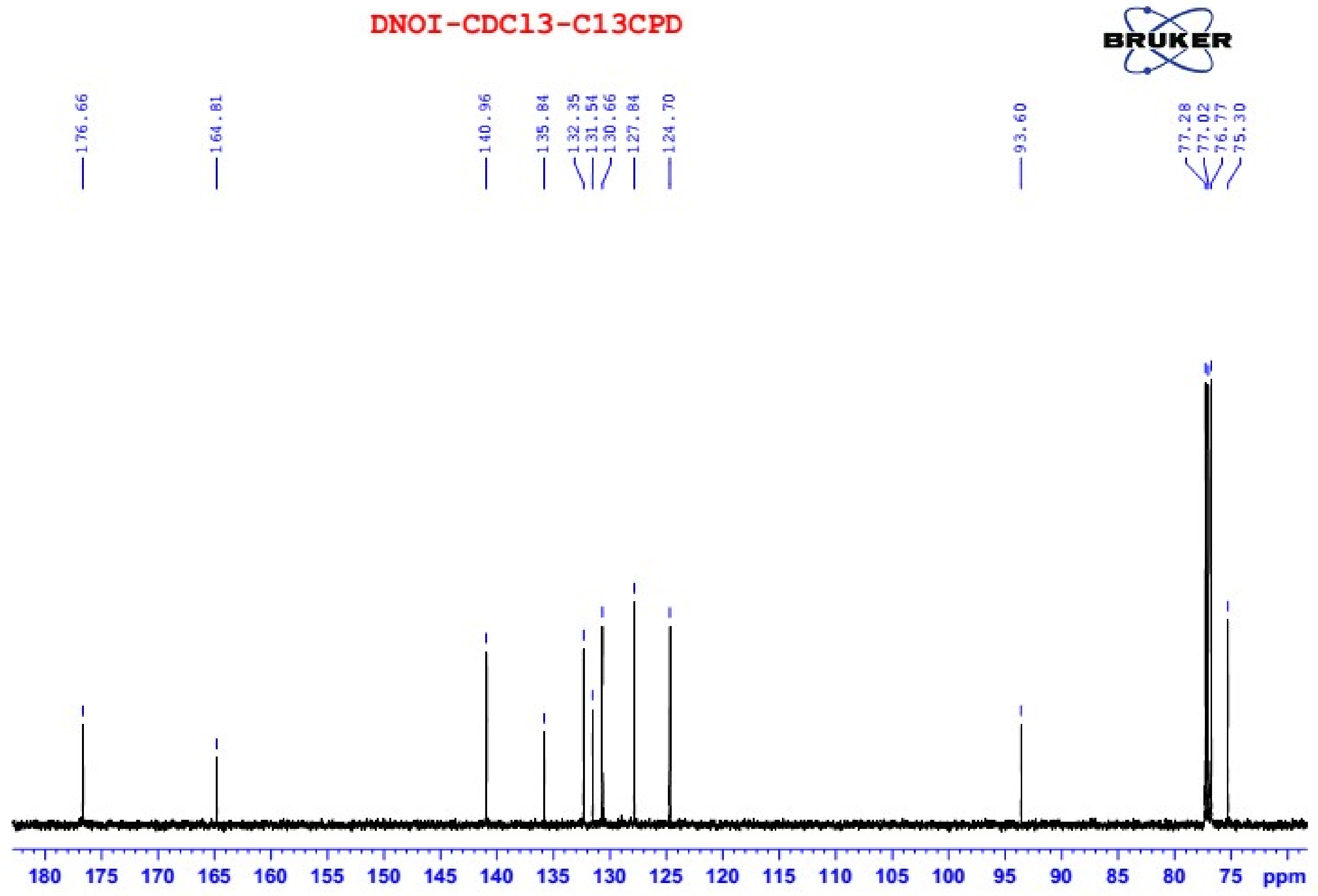


$^{13}$C-NMR spectrum of compound **3h** (extension)

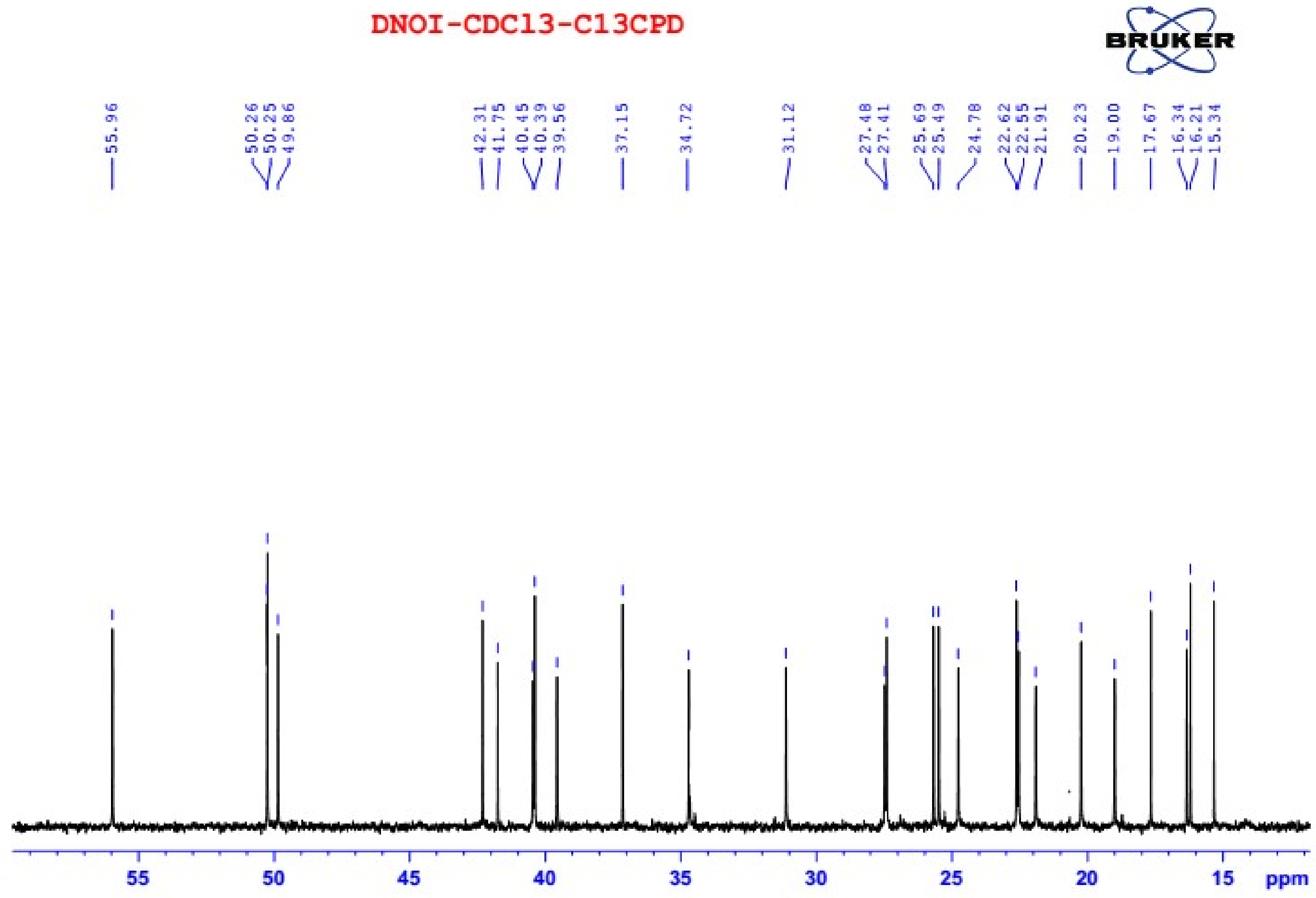


$^{13}$C-NMR spectrum of compound **3h** (extension)

### 1.11. Compound 3i

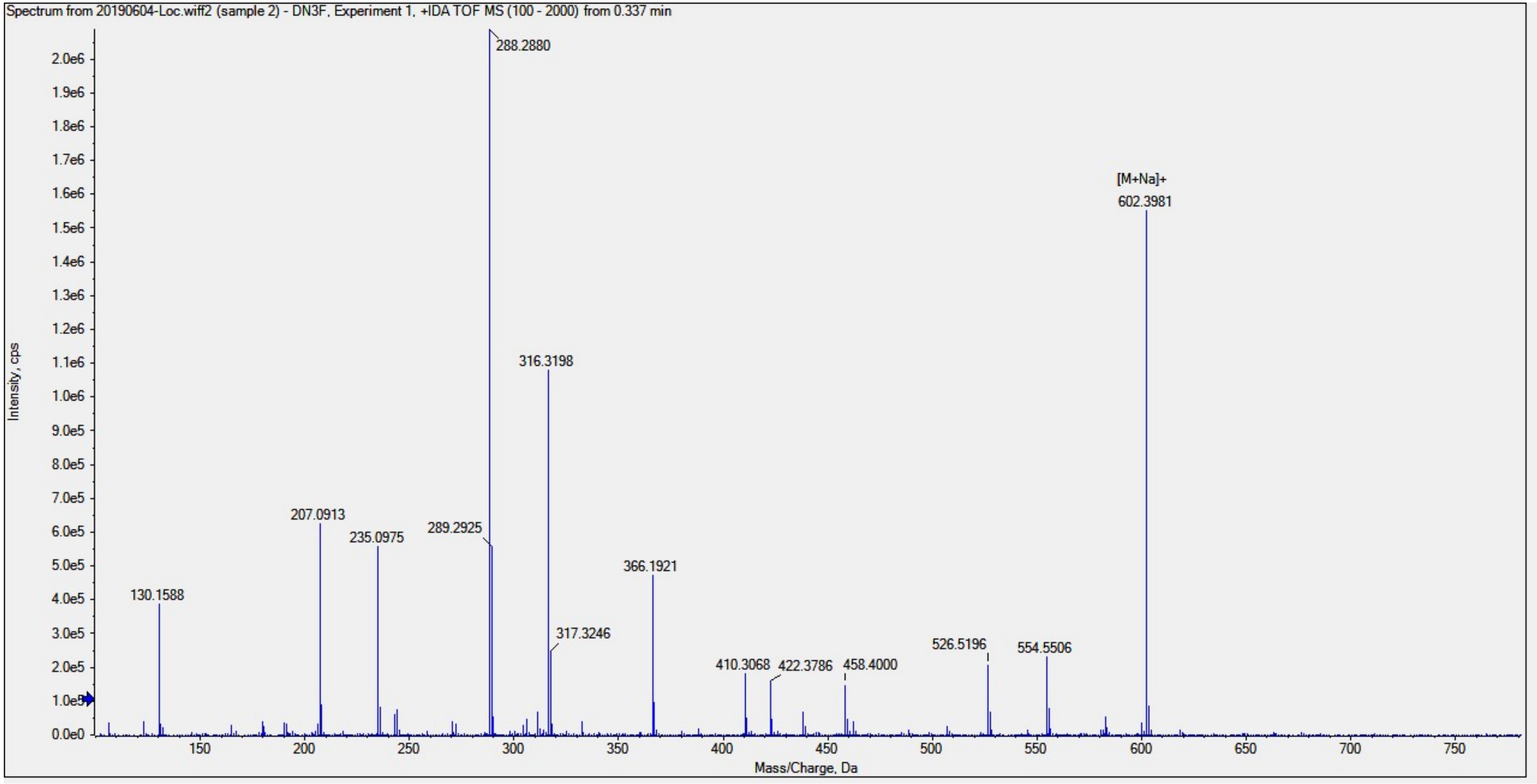


(+)-HR-ESI-MS spectrum of compound **3i**

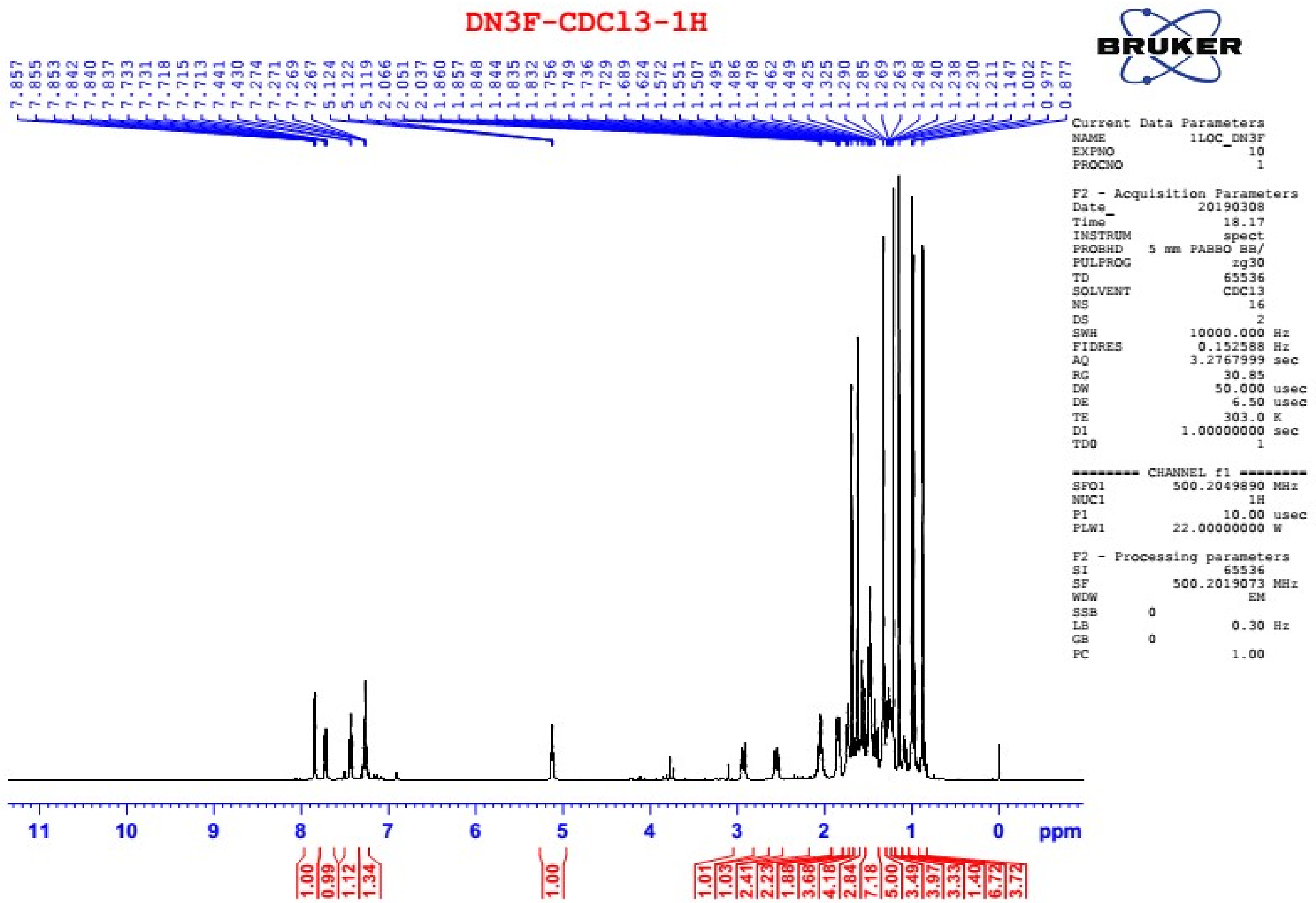


$^1$H-NMR spectrum of compound **3i**

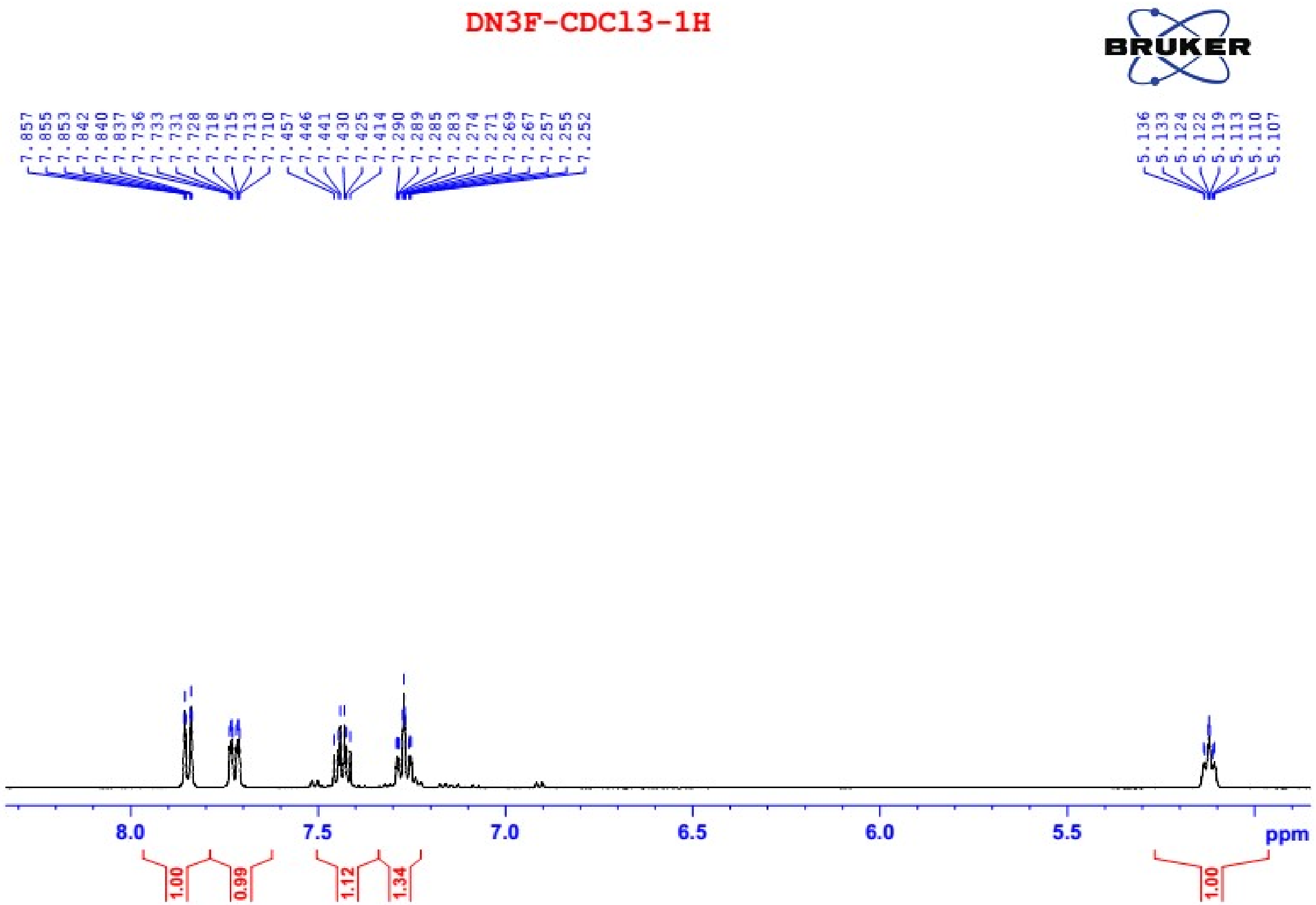


$^{1}$H-NMR spectrum of compound **3i** (extension)

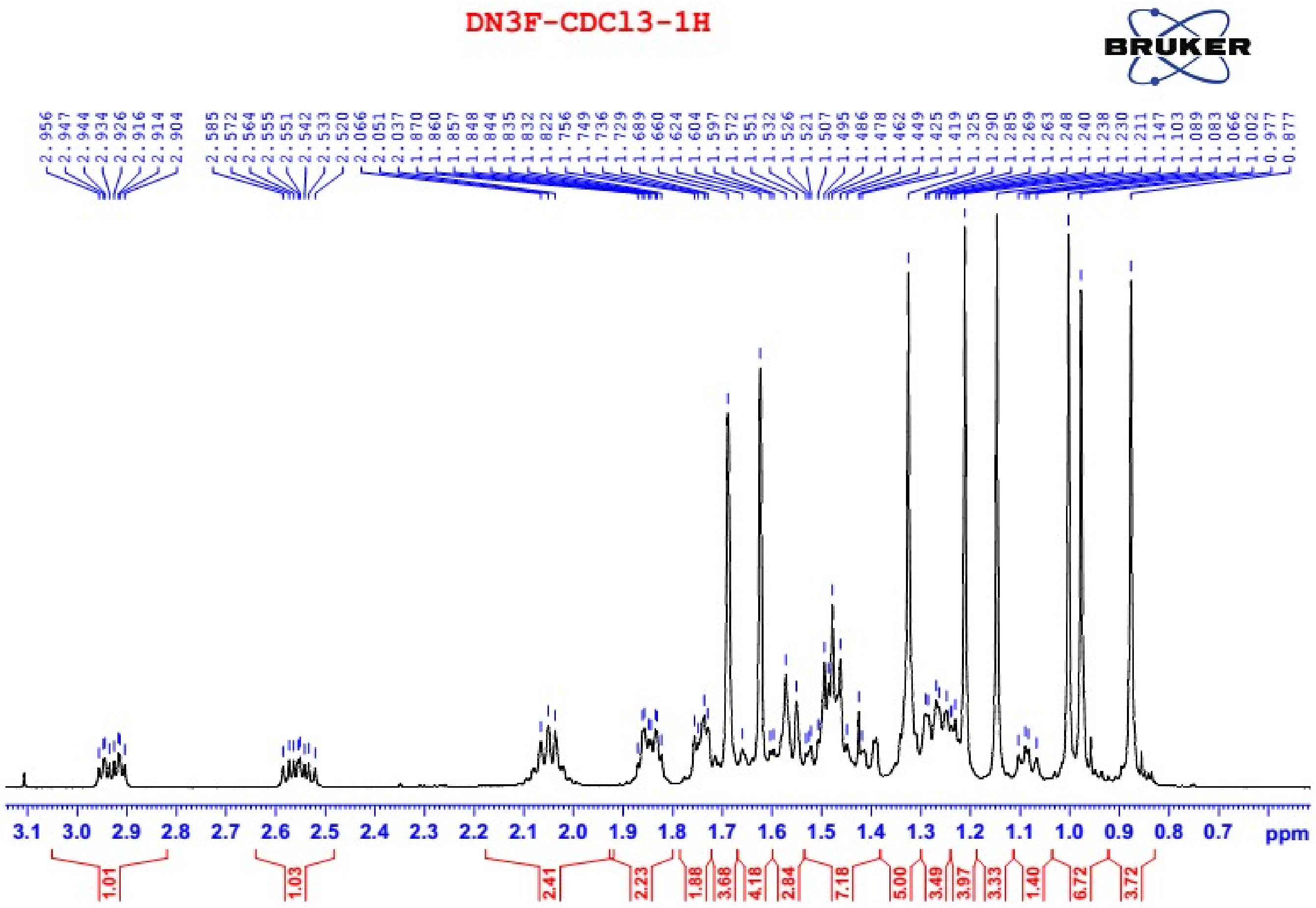


$^{1}$H-NMR spectrum of compound **3i** (extension)

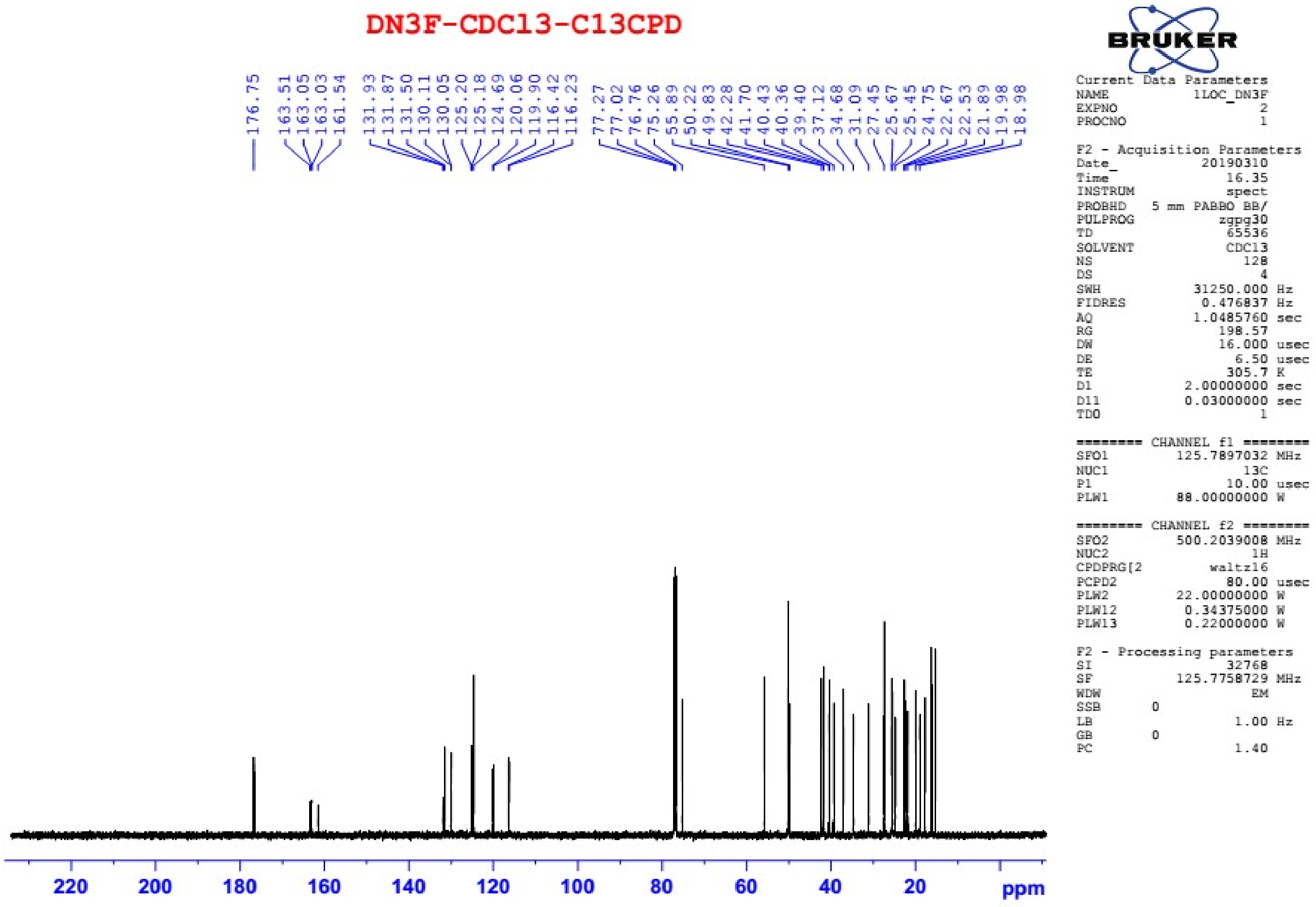


$^{13}$C-NMR spectrum of compound **3i**

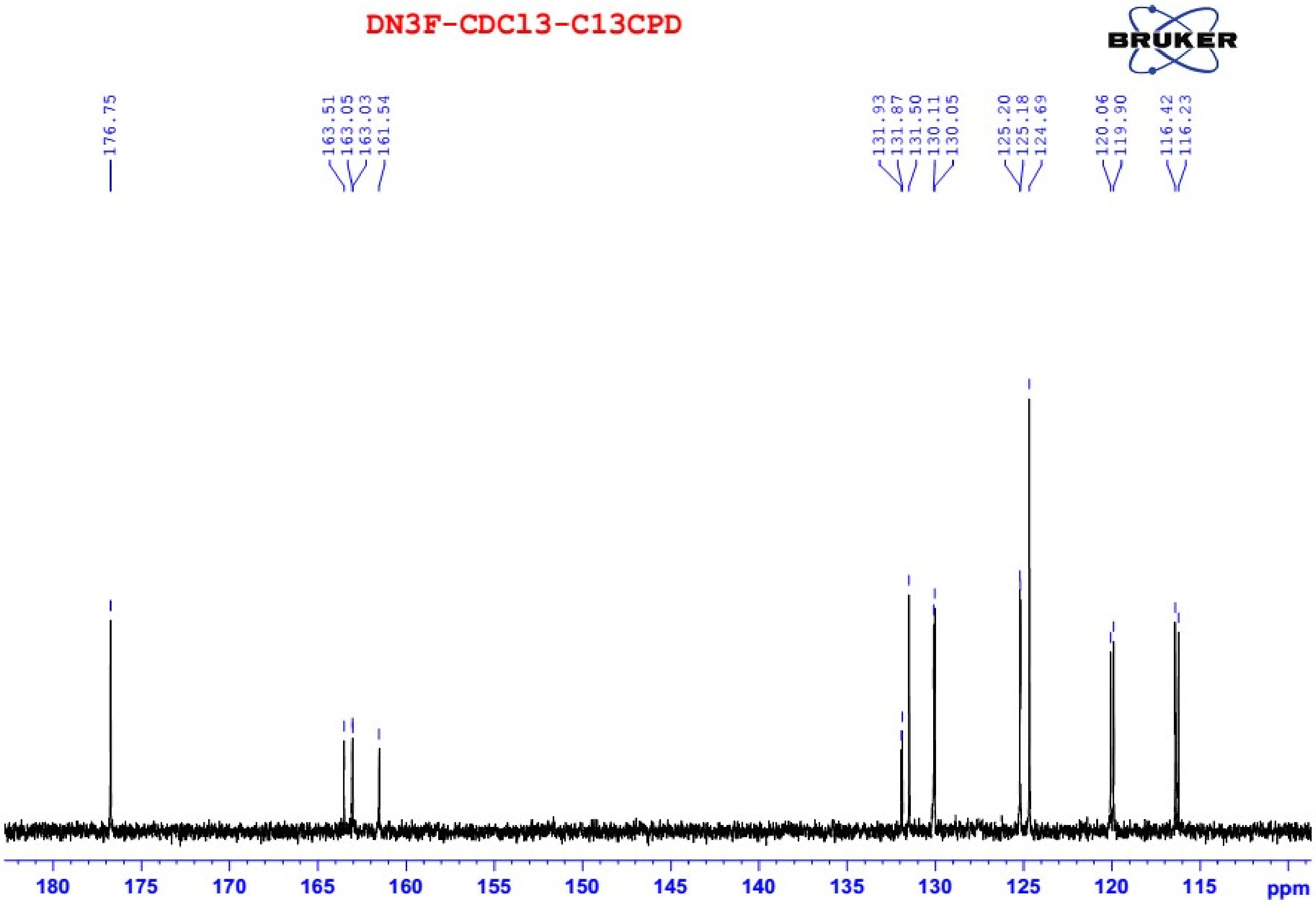


$^{13}$C-NMR spectrum of compound **3i** (extension)

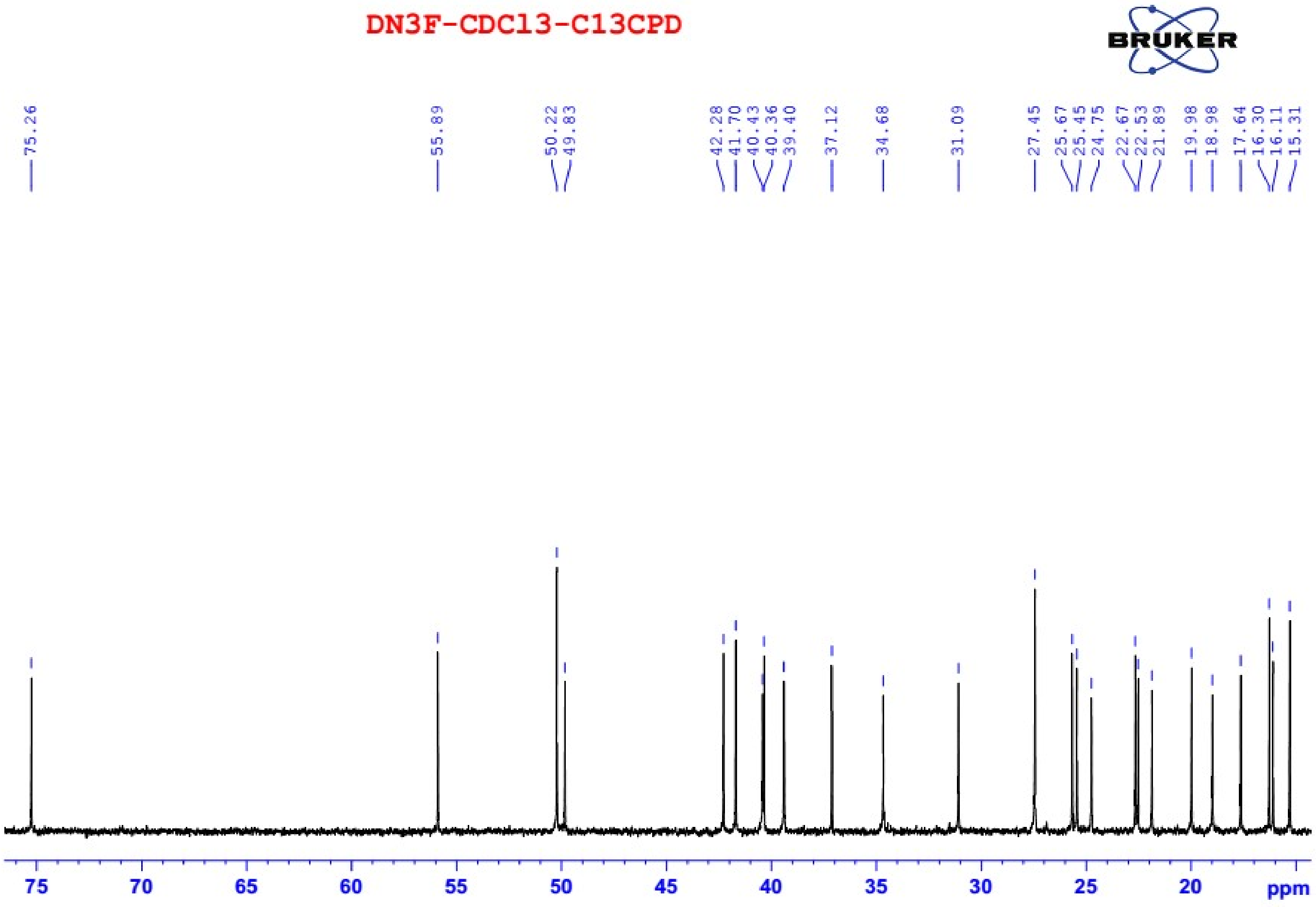


$^{13}$C-NMR spectrum of compound **3i** (extension)

## 1.12. Compound 3k

**Sample name: DNAca**
**Operator: Le Anh VHH**
**Method: +IDA TOF MS/MS**
**Date: 2021.04.23**

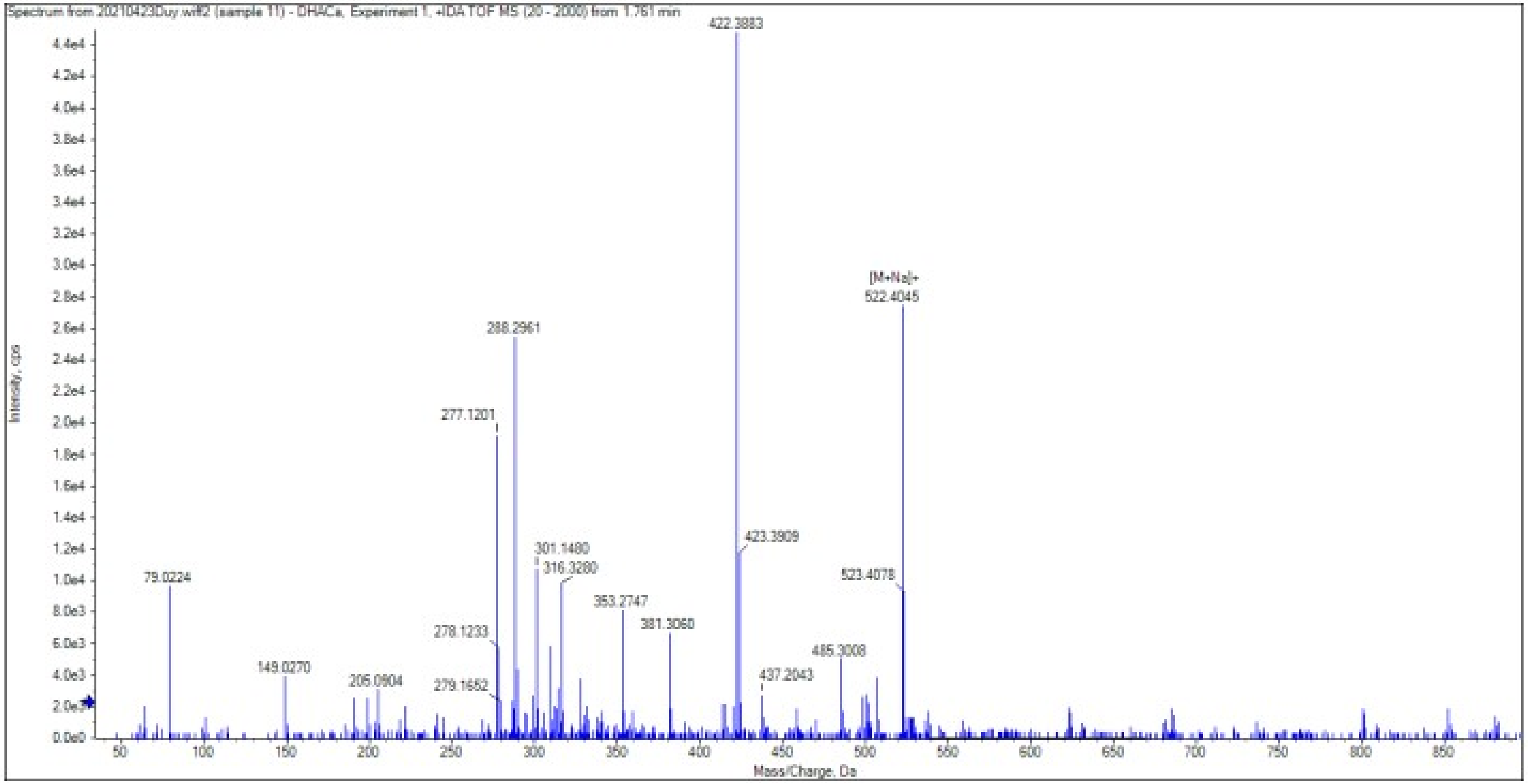


| Hit | Formula | m/z | RDB | ppm | MS Rank | MSMS ppm | MSMS Rank | Found |
|---|---|---|---|---|---|---|---|---|
| 1 | C32H53NO3 | 522.39977 | 7.0 | 4.4 | 1 | | | NA/NA |

(+)-HR-ESI-MS spectrum of compound **3k**

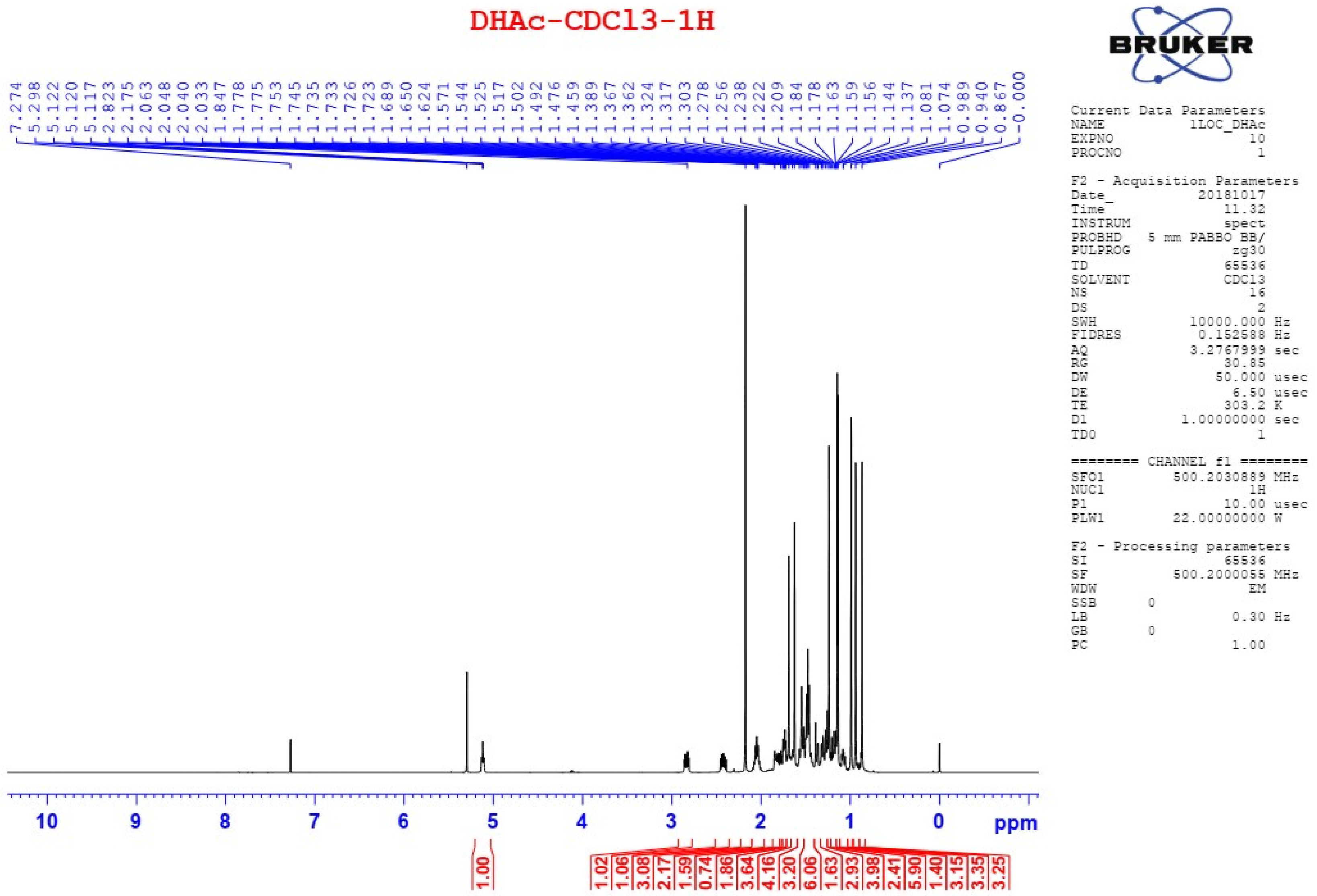

$^{1}$H-NMR spectrum of compound **3k**

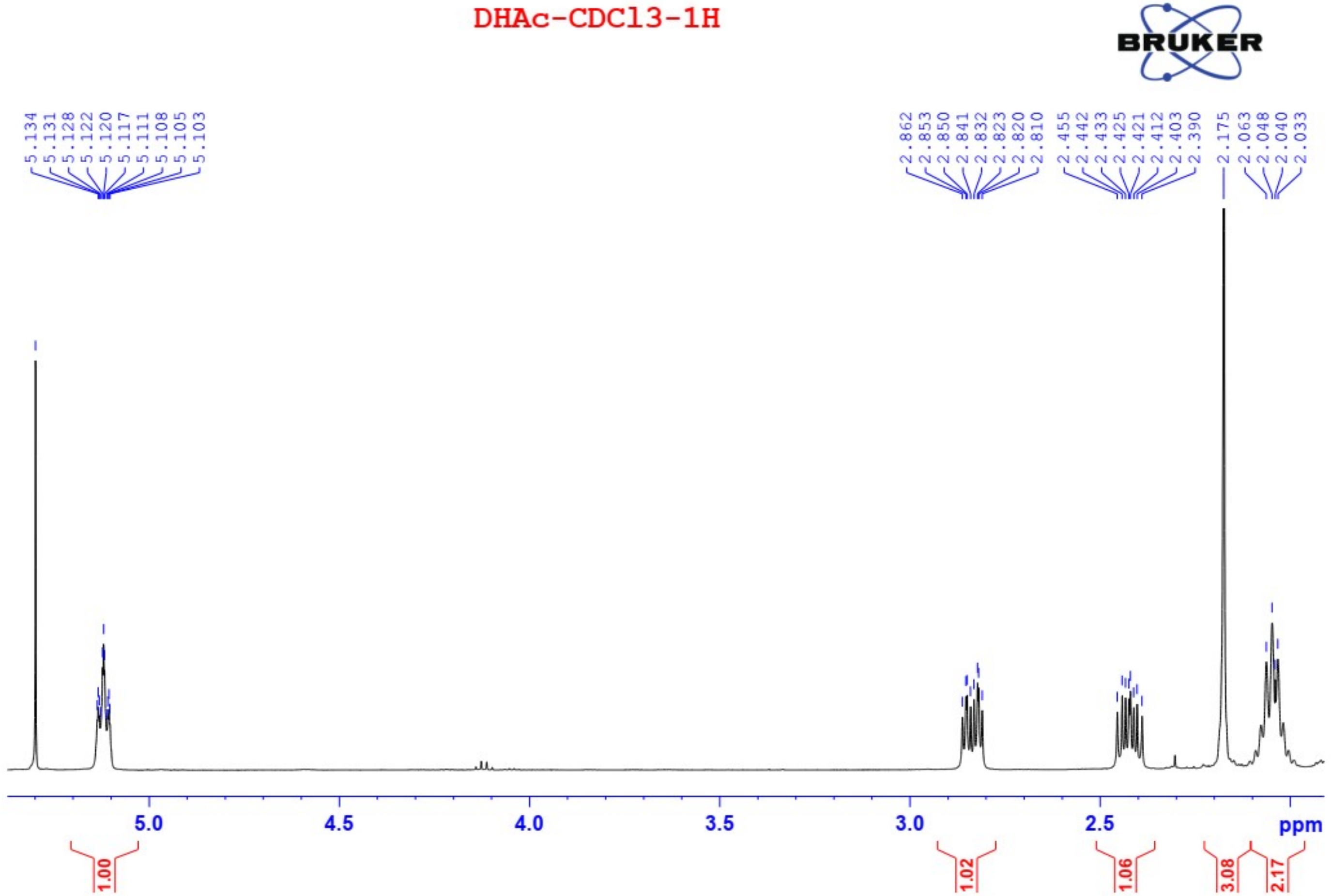


$^{1}$H-NMR spectrum of compound **3k** (extension)

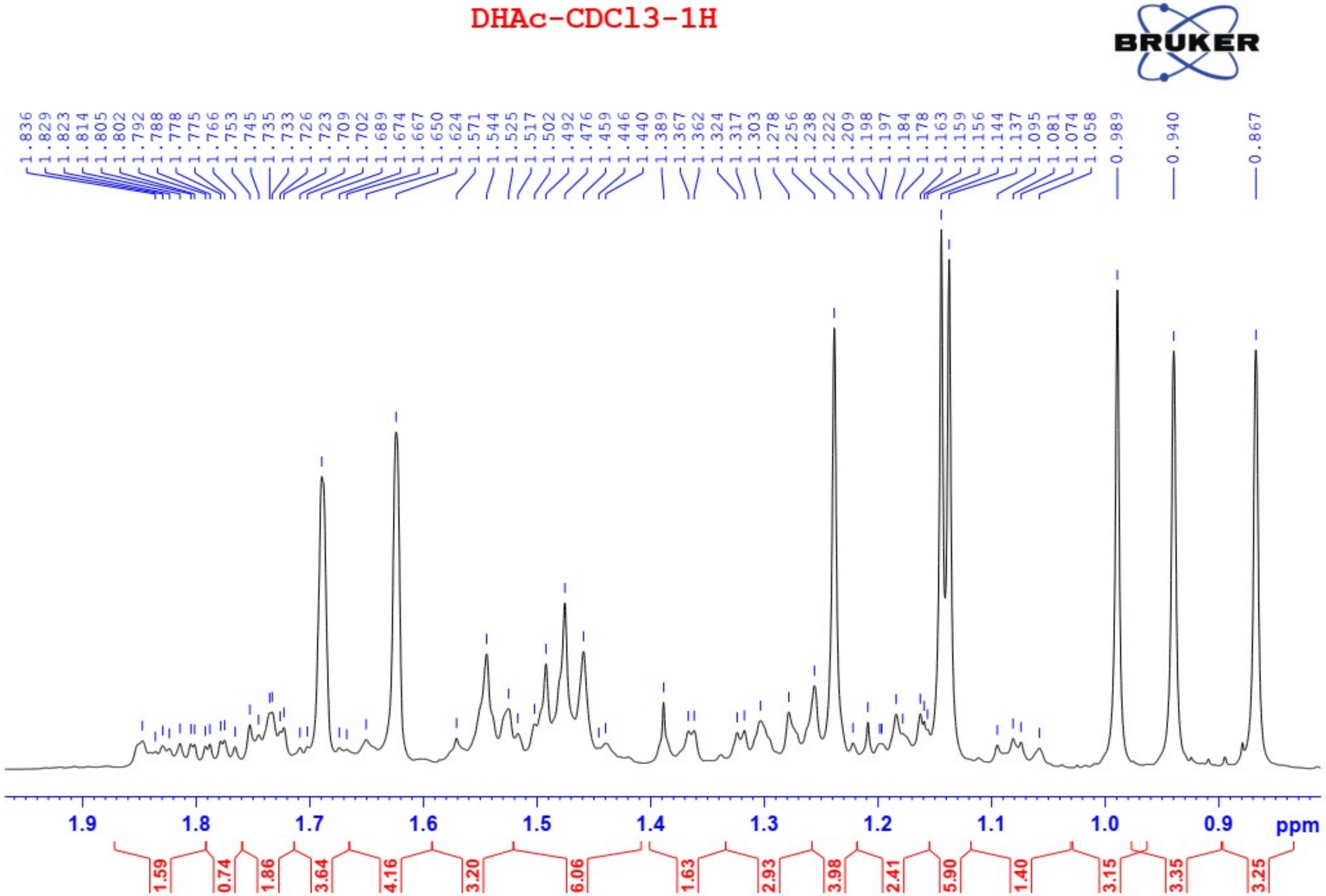


$^{1}$H-NMR spectrum of compound **3k** (extension)

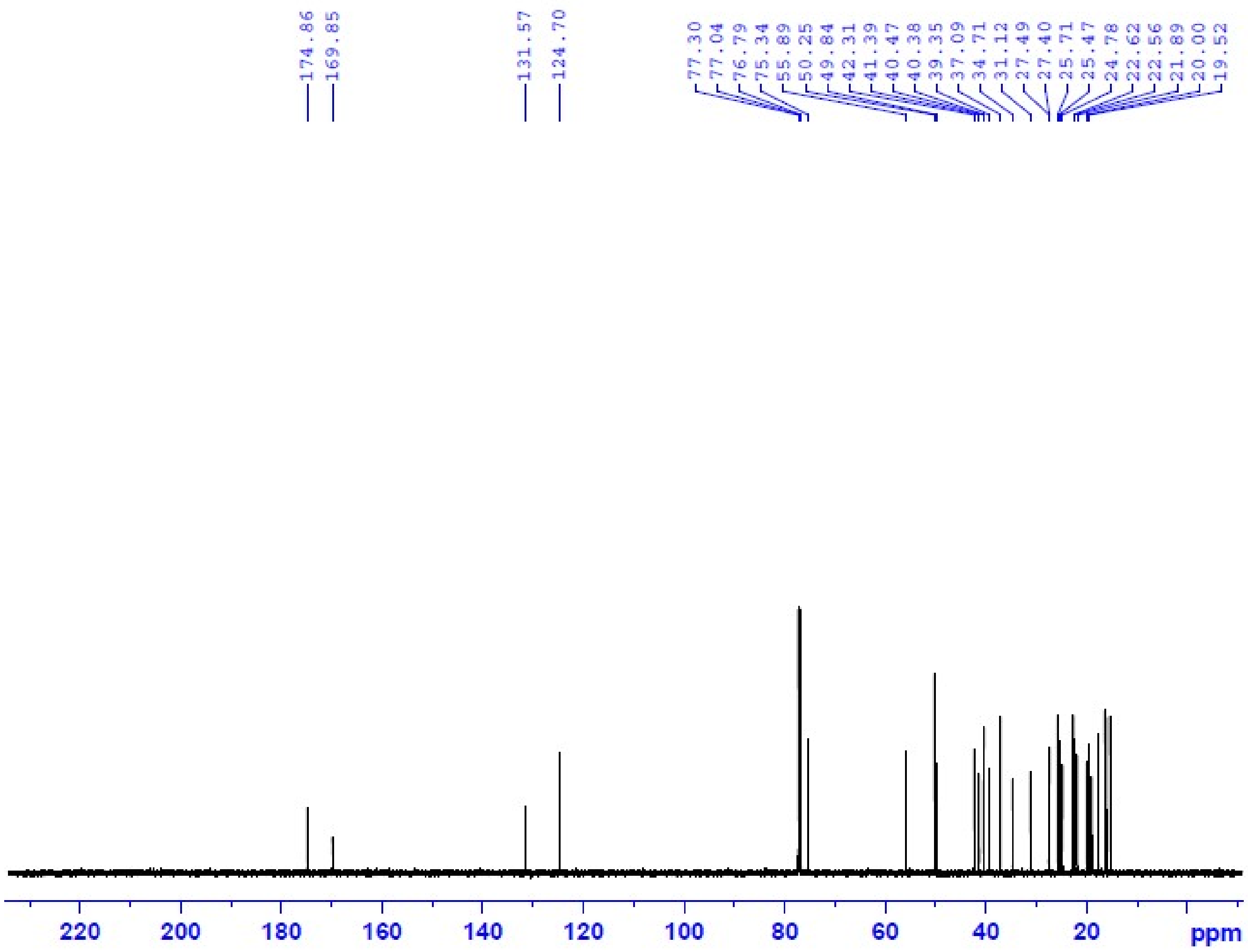


$^{13}C$-NMR spectrum of compound **3k**

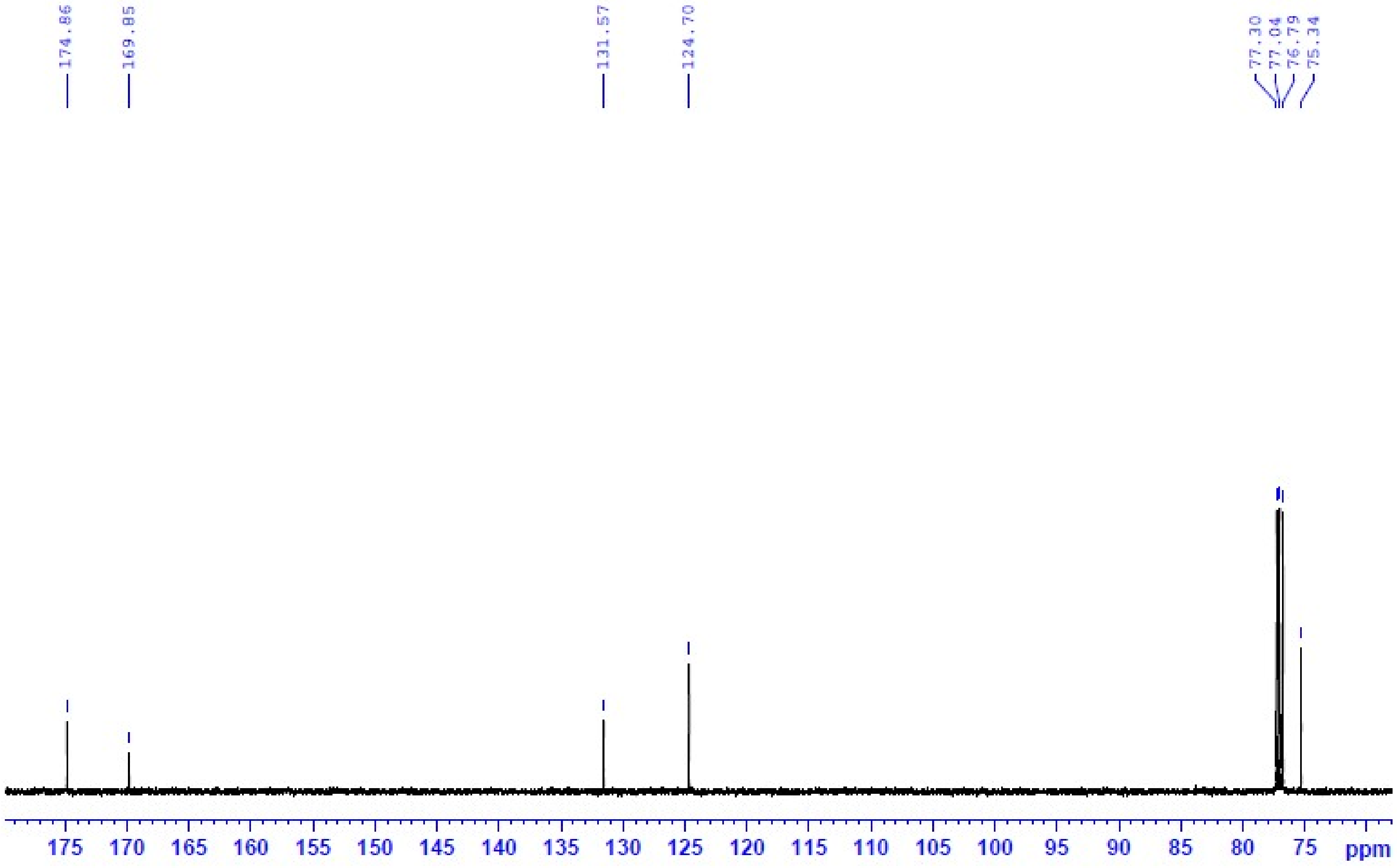

$^{13}$C-NMR spectrum of compound **3k** (extension)

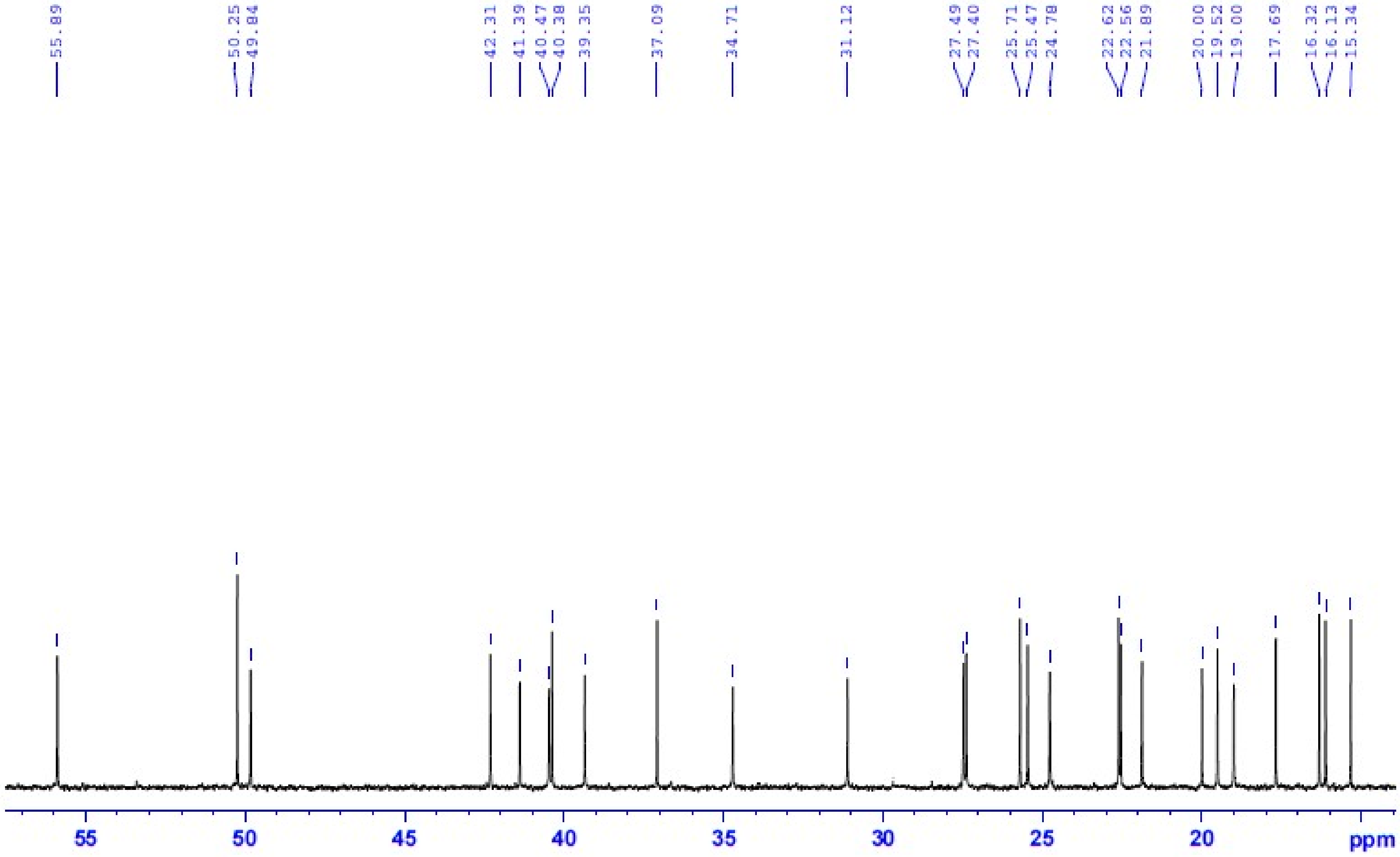

55.89
50.25
49.84
42.31
41.39
40.47
40.38
39.35
37.09
34.71
31.12
27.49
27.40
25.71
25.47
24.78
22.62
22.56
21.89
20.00
19.52
19.00
17.69
16.32
16.13
15.34
55
50
45
40
35
30
25
20
ppm

$^{13}$C-NMR spectrum of compound **3k** (extension)

## 1.13. Compound 3l

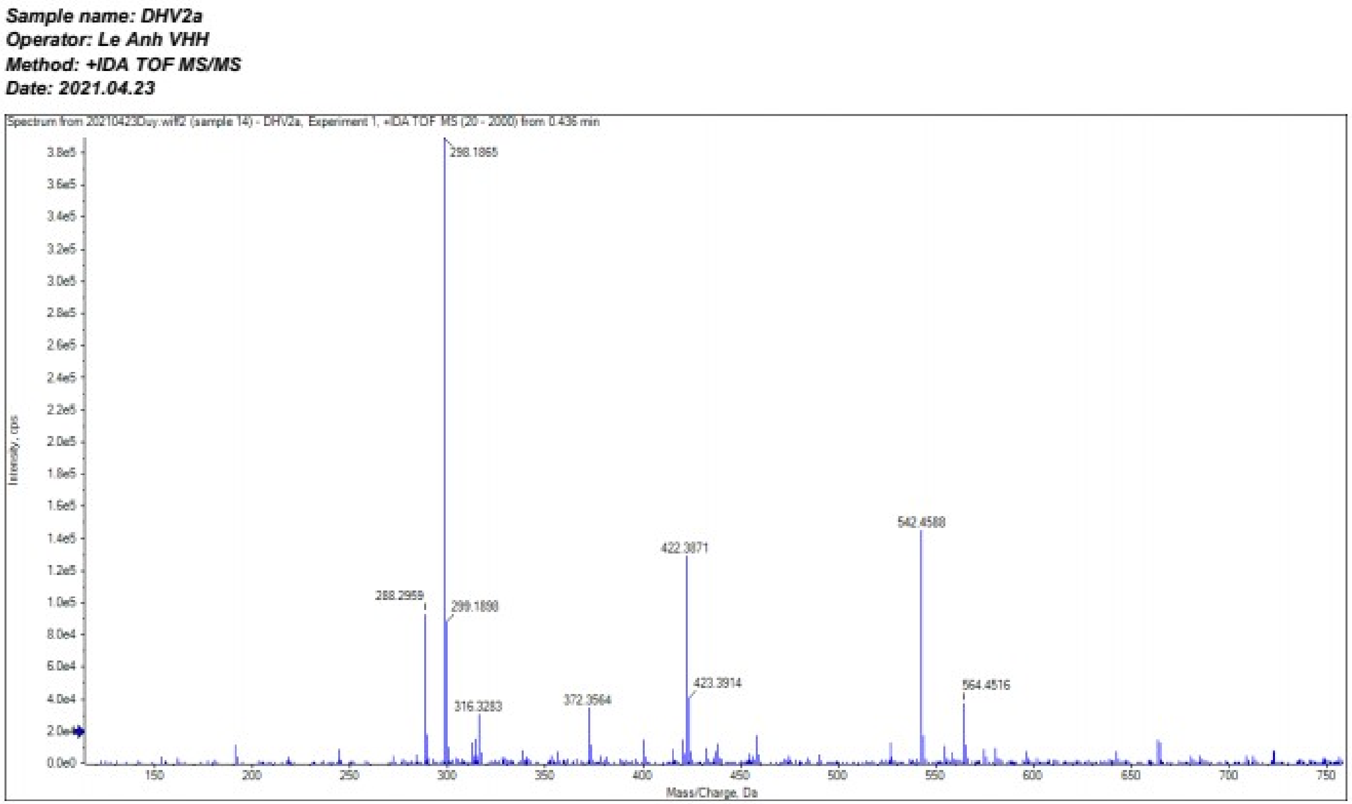


| Hit | Formula | m/z | RDB | ppm | MS Rank | MSMS ppm | MSMS Rank | Found |
|---|---|---|---|---|---|---|---|---|
| 1 | C35H59NO3 | 542.45677 | 7.0 | 3.7 | 1 | | | NA/NA |

(+)-HR-ESI-MS spectrum of compound **3l**

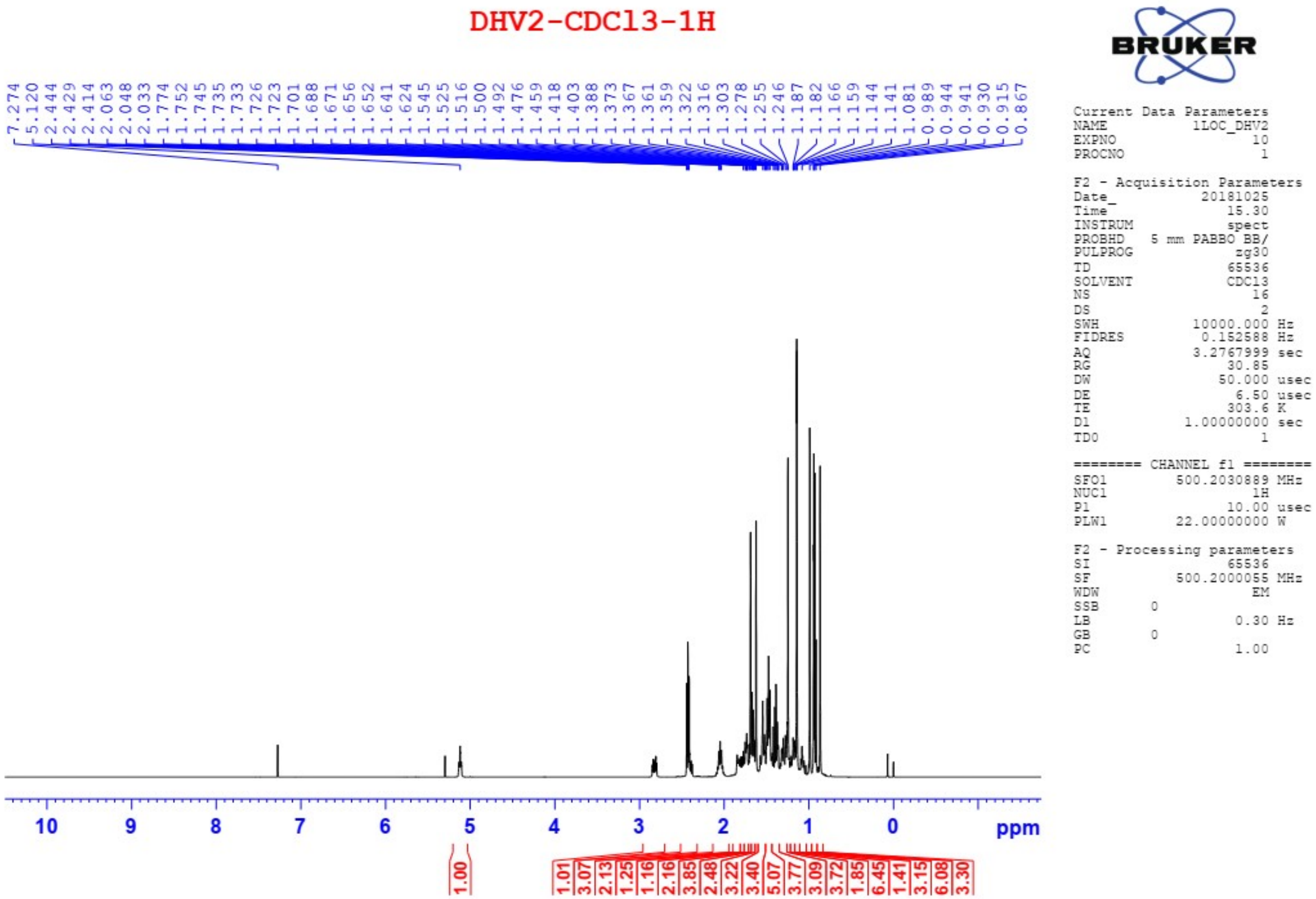


$^{1}$H-NMR spectrum of compound **3l**

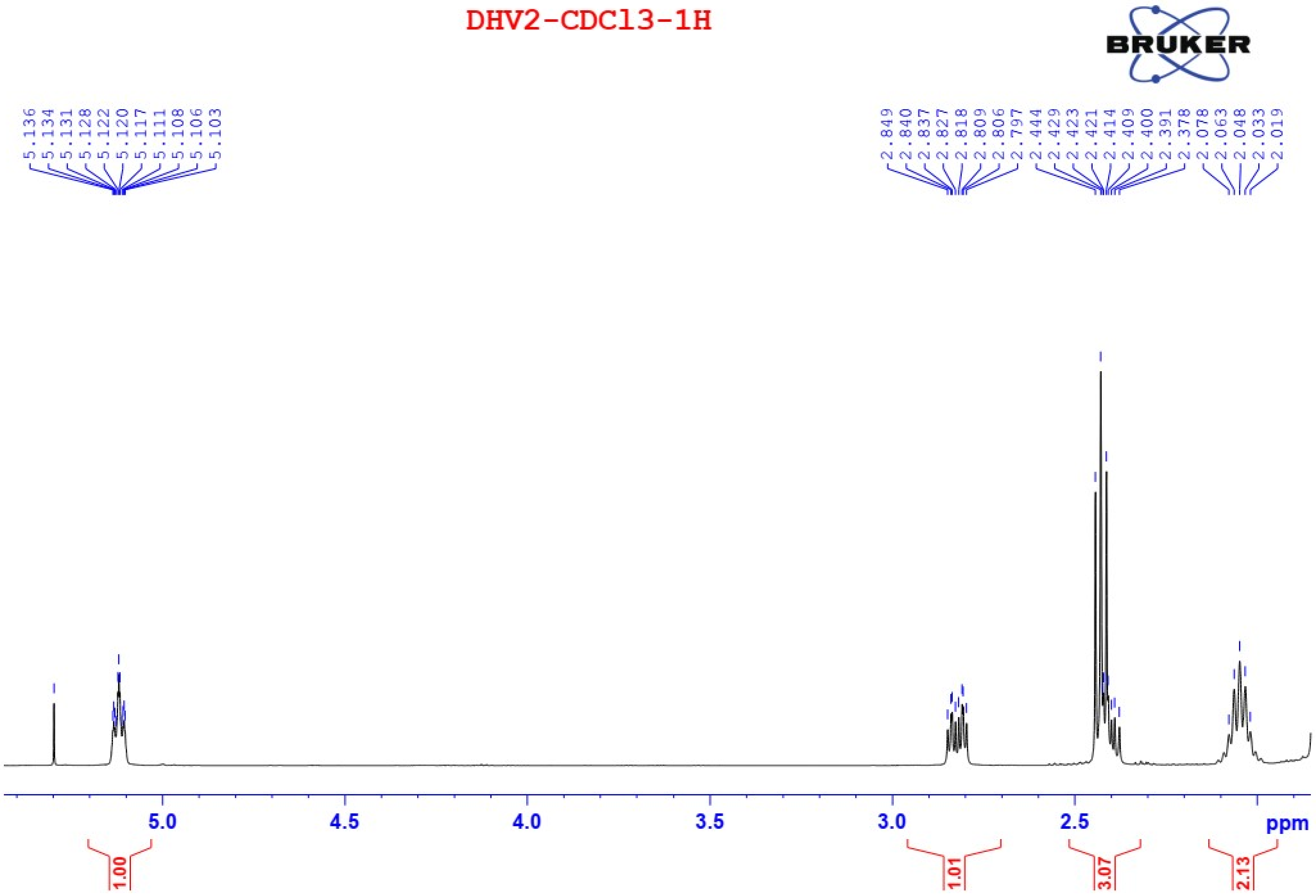


$^{1}$H-NMR spectrum of compound **3l** (extension)

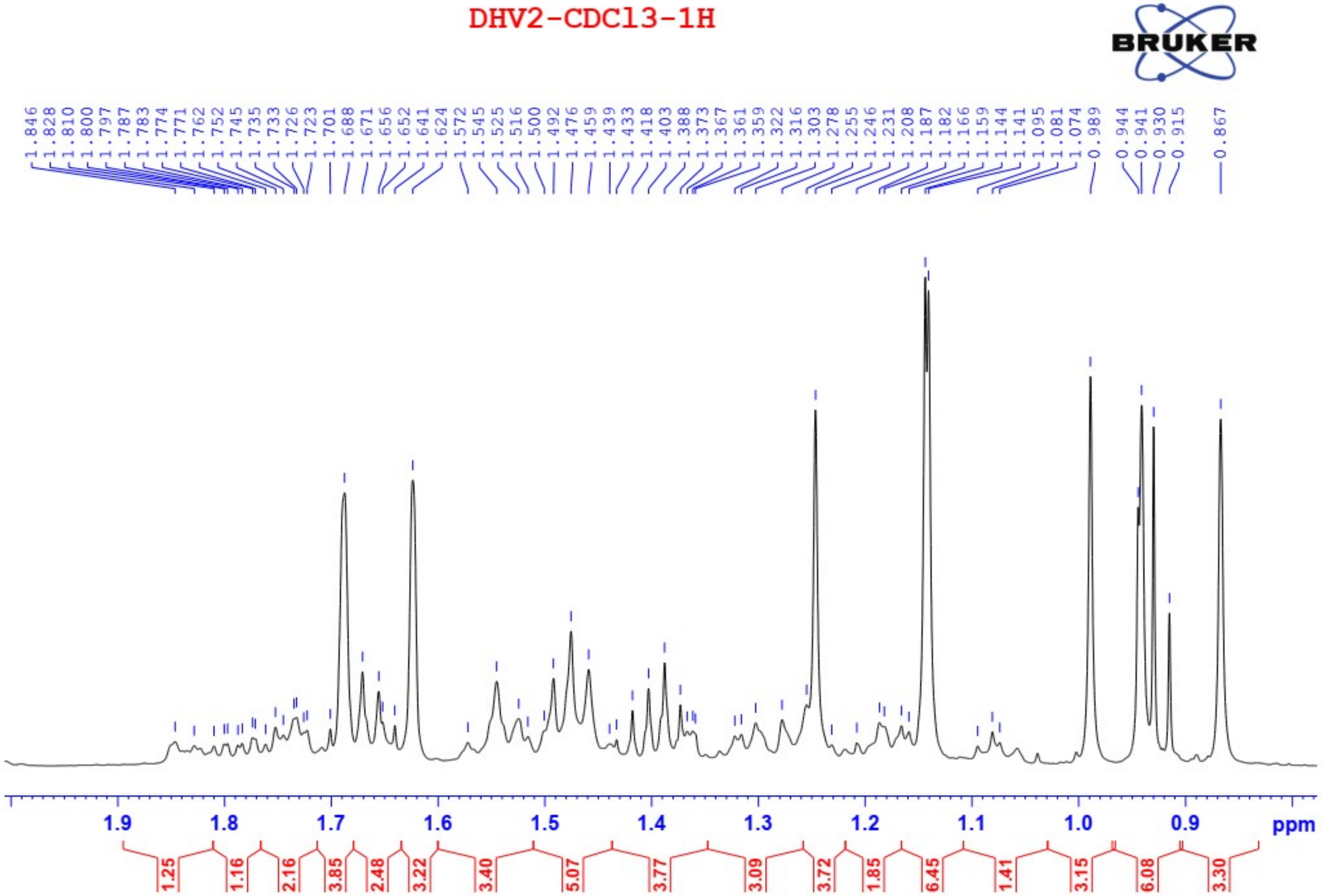

$^{1}$H-NMR spectrum of compound **3l** (extension)

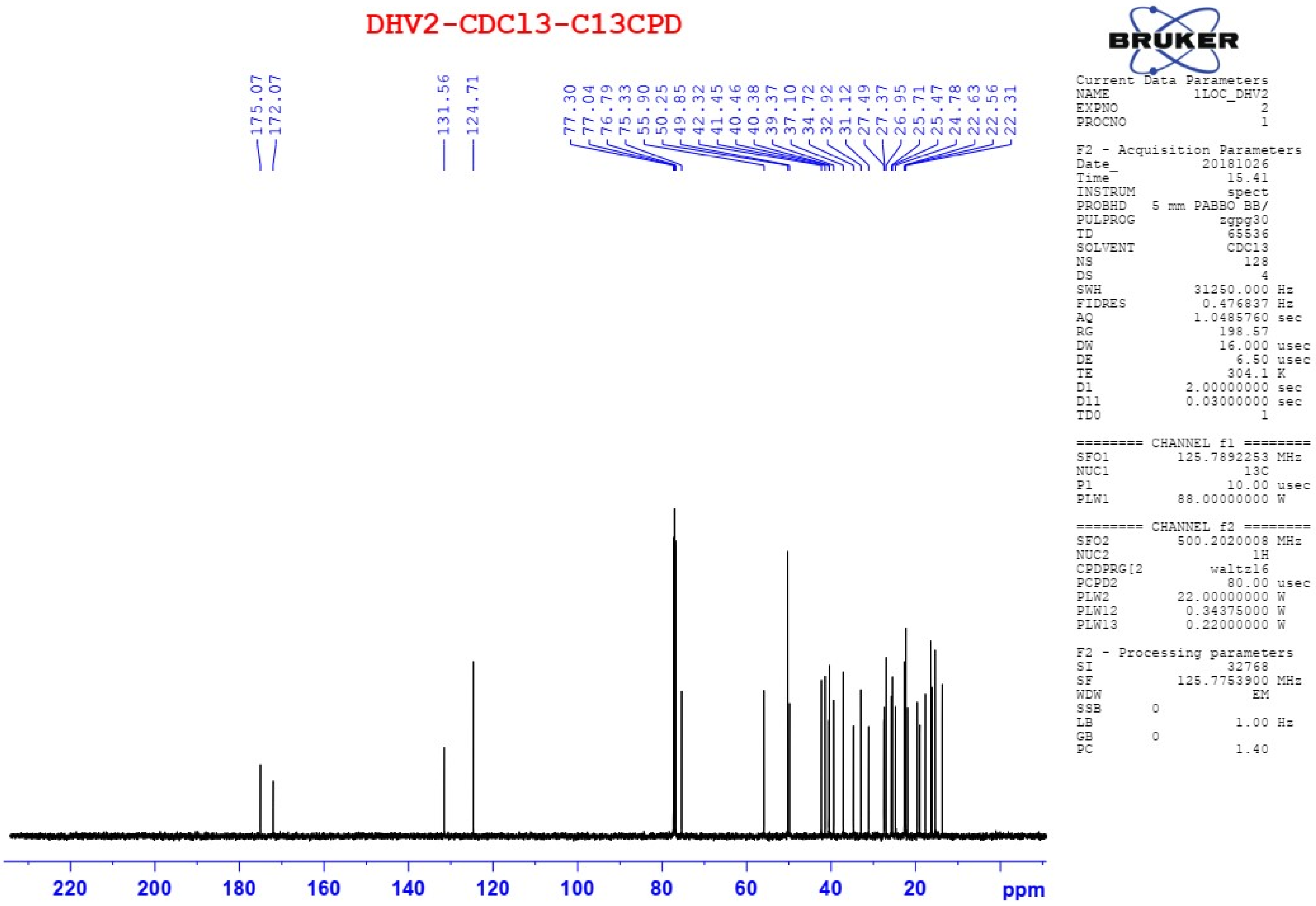


$^{13}$C-NMR spectrum of compound **3l**

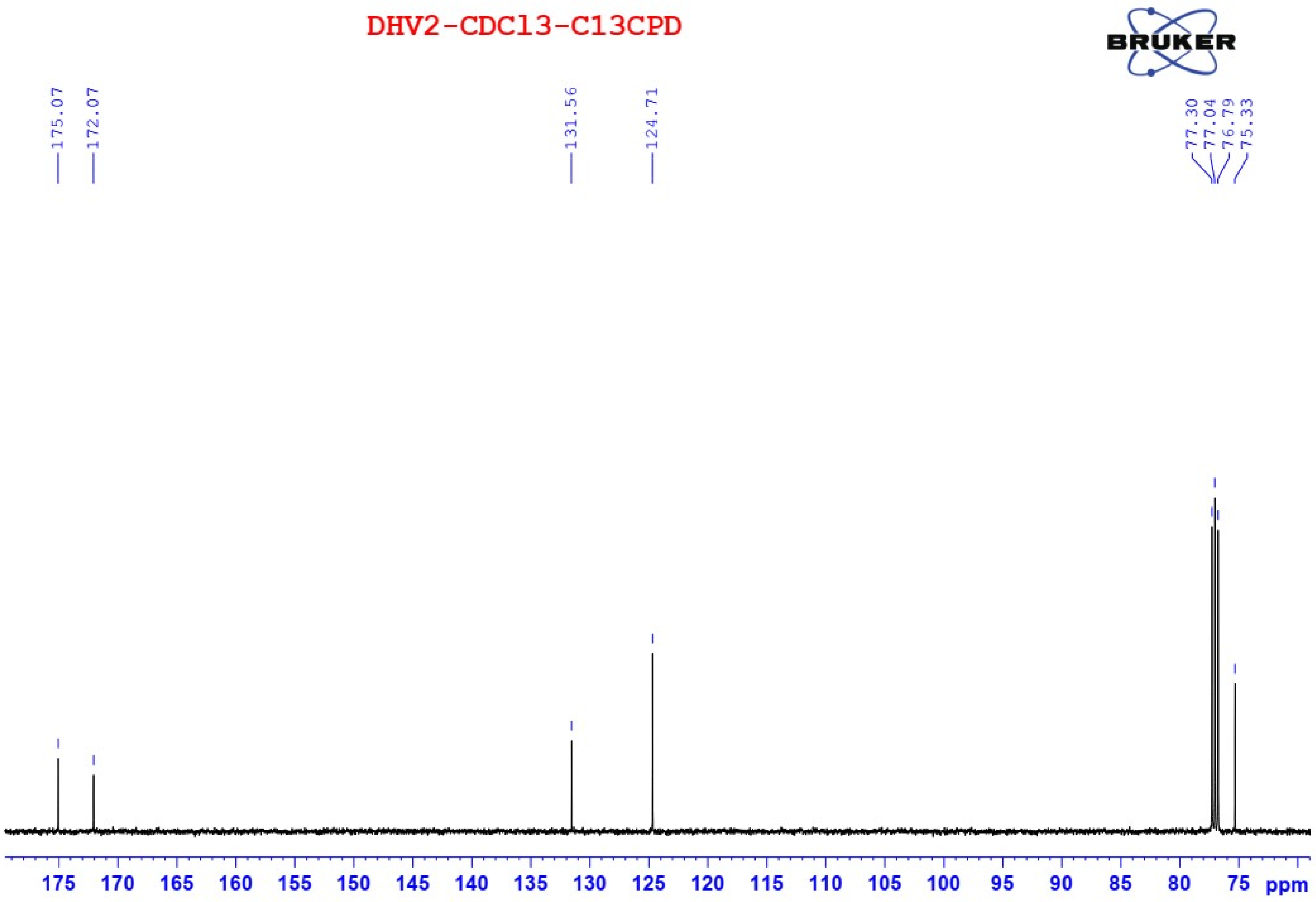


$^{13}C$-NMR spectrum of compound **3l** (extension)

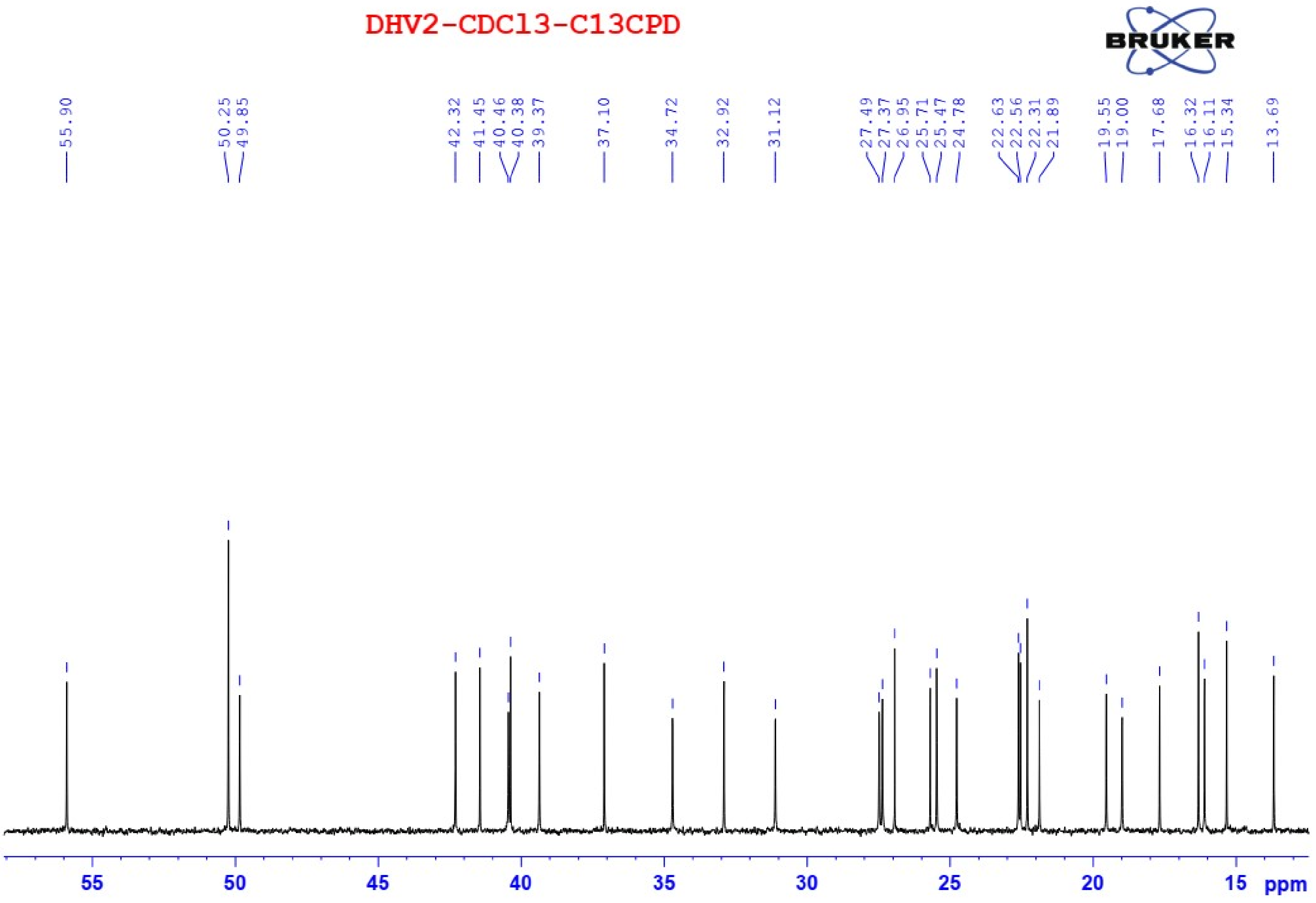


$^{13}$C-NMR spectrum of compound **3l** (extension)

## 1.14. Compound 3m

***Sample name: DNOIZb***
***Operator: Le Anh VHH***
***Method: +IDA TOF MS/MS***
***Date: 2021.04.23***

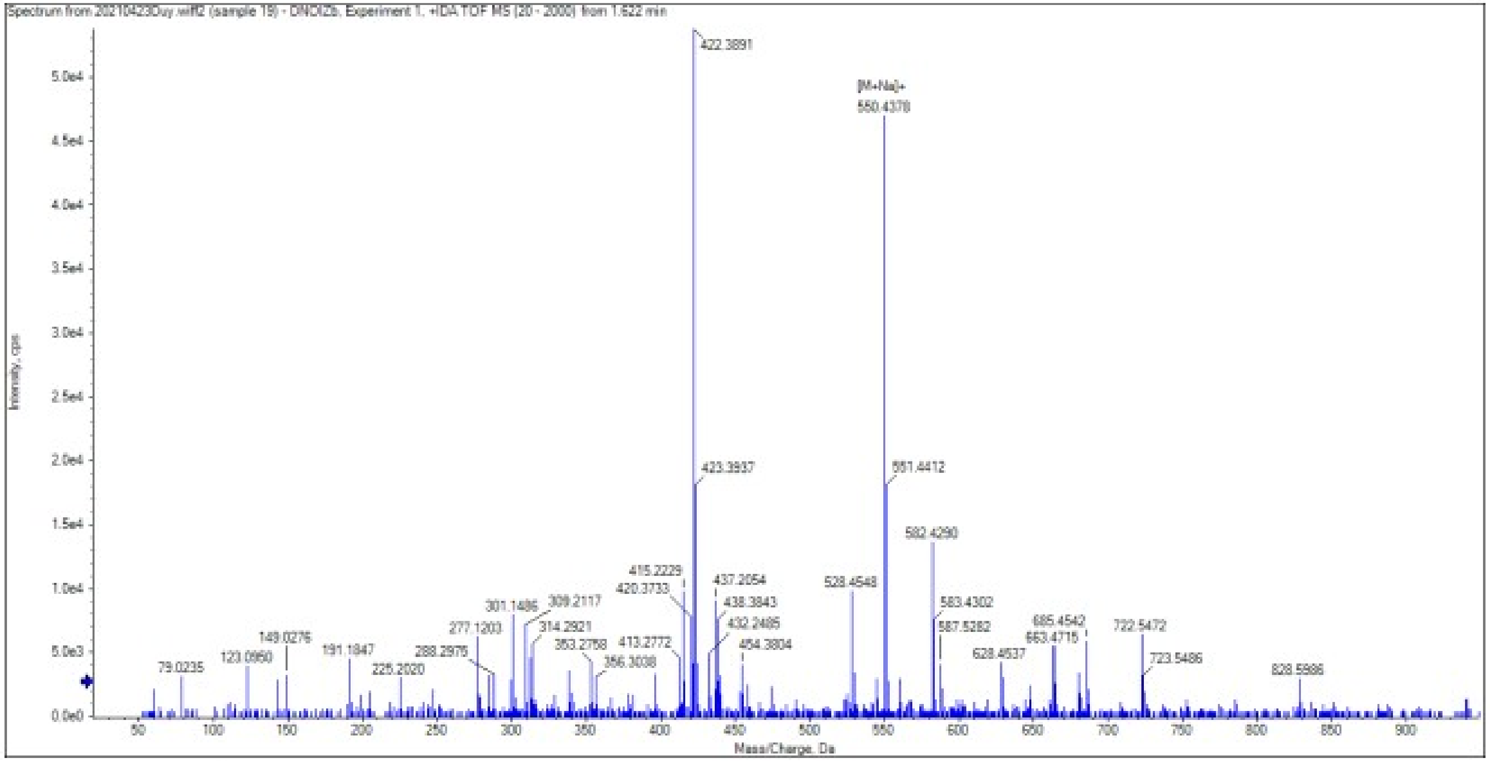


| Hit | Formula | m/z | RDB | ppm | MS Rank | MSMS ppm | MSMS Rank | Found |
|---|---|---|---|---|---|---|---|---|
| 1 | C34H57NO3 | 550.43307 | 7.0 | 4.8 | 1 | | | NA/NA |

(+)-HR-ESI-MS spectrum of compound **3m**

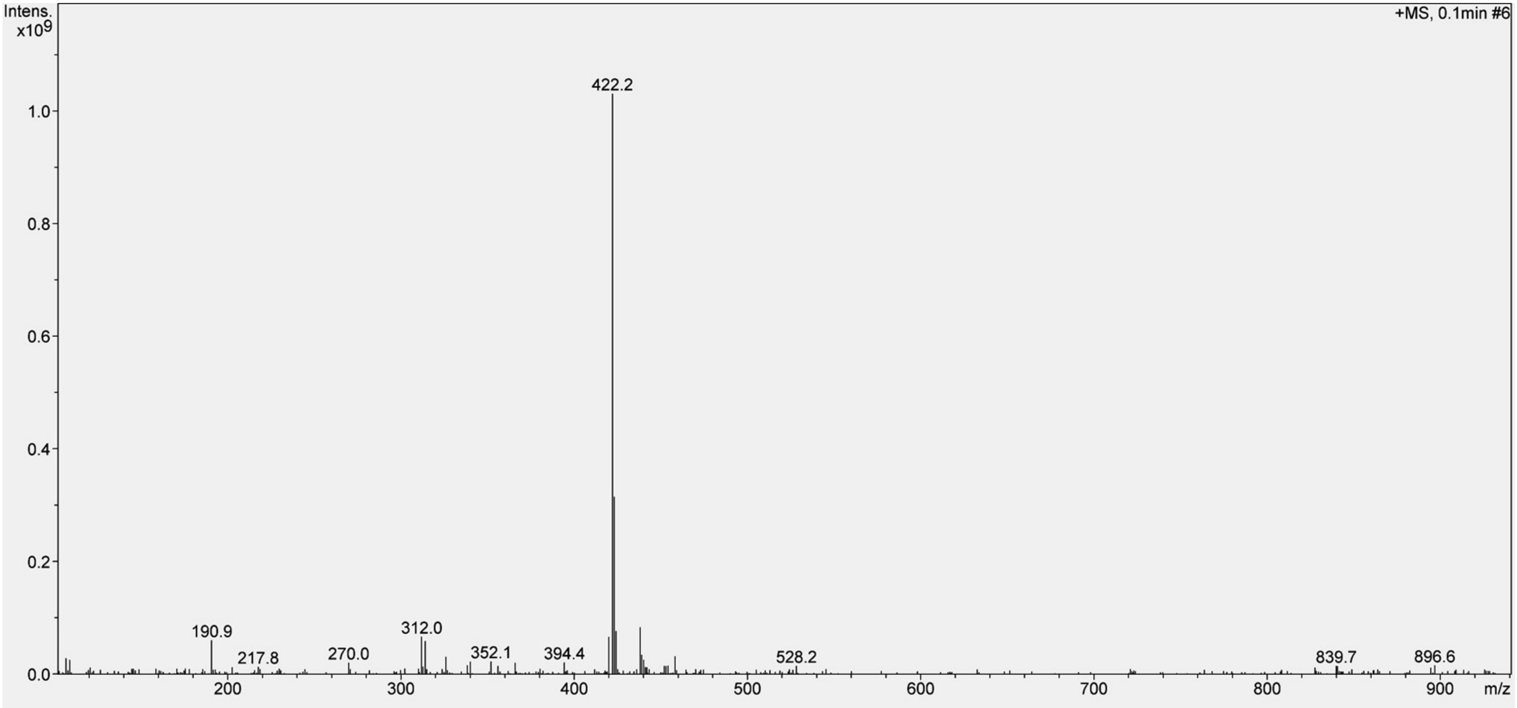


(+)-ESI–MS spectrum of compound **3m**

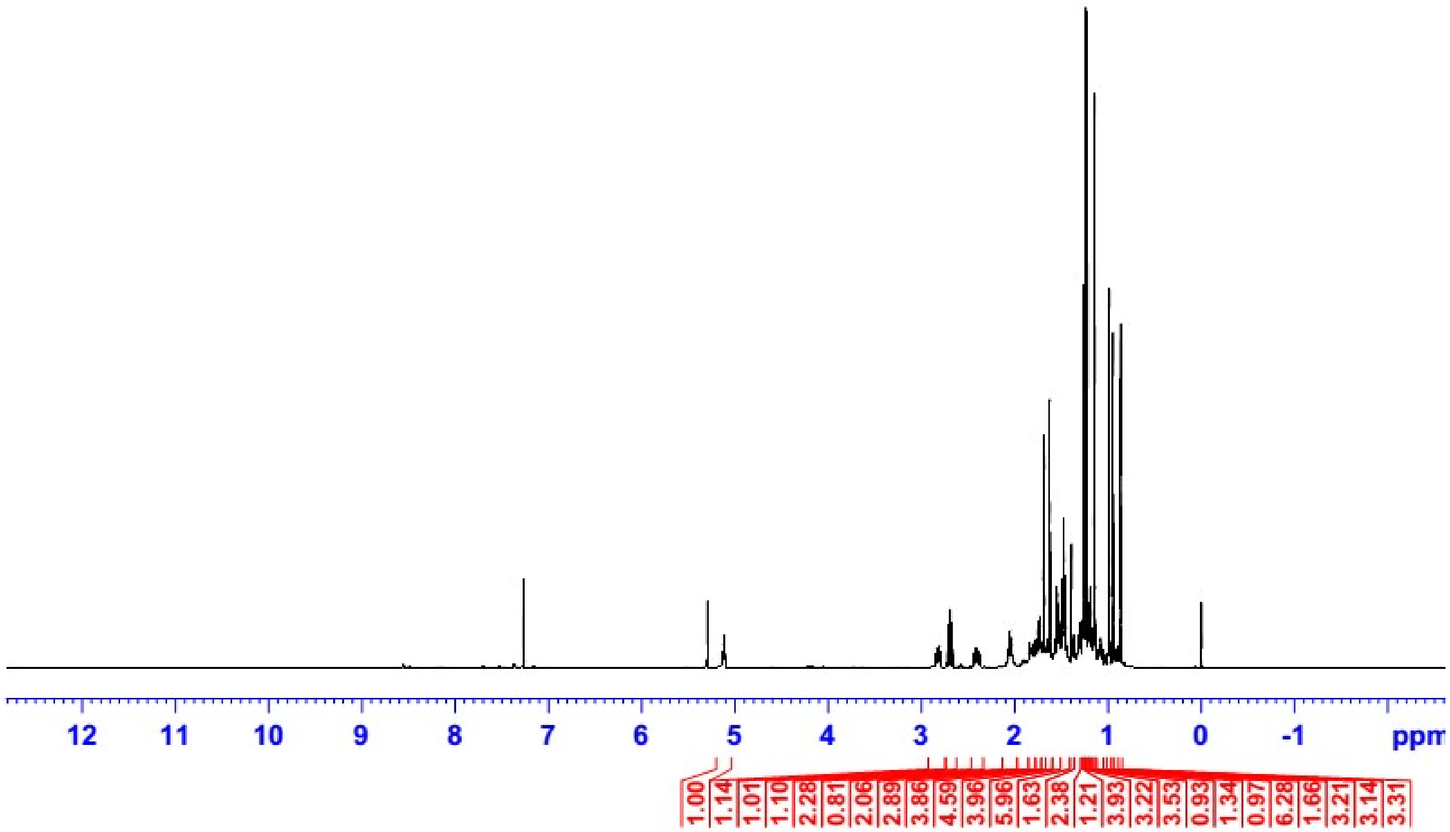


$^{1}$H NMR spectrum of compound **3m**

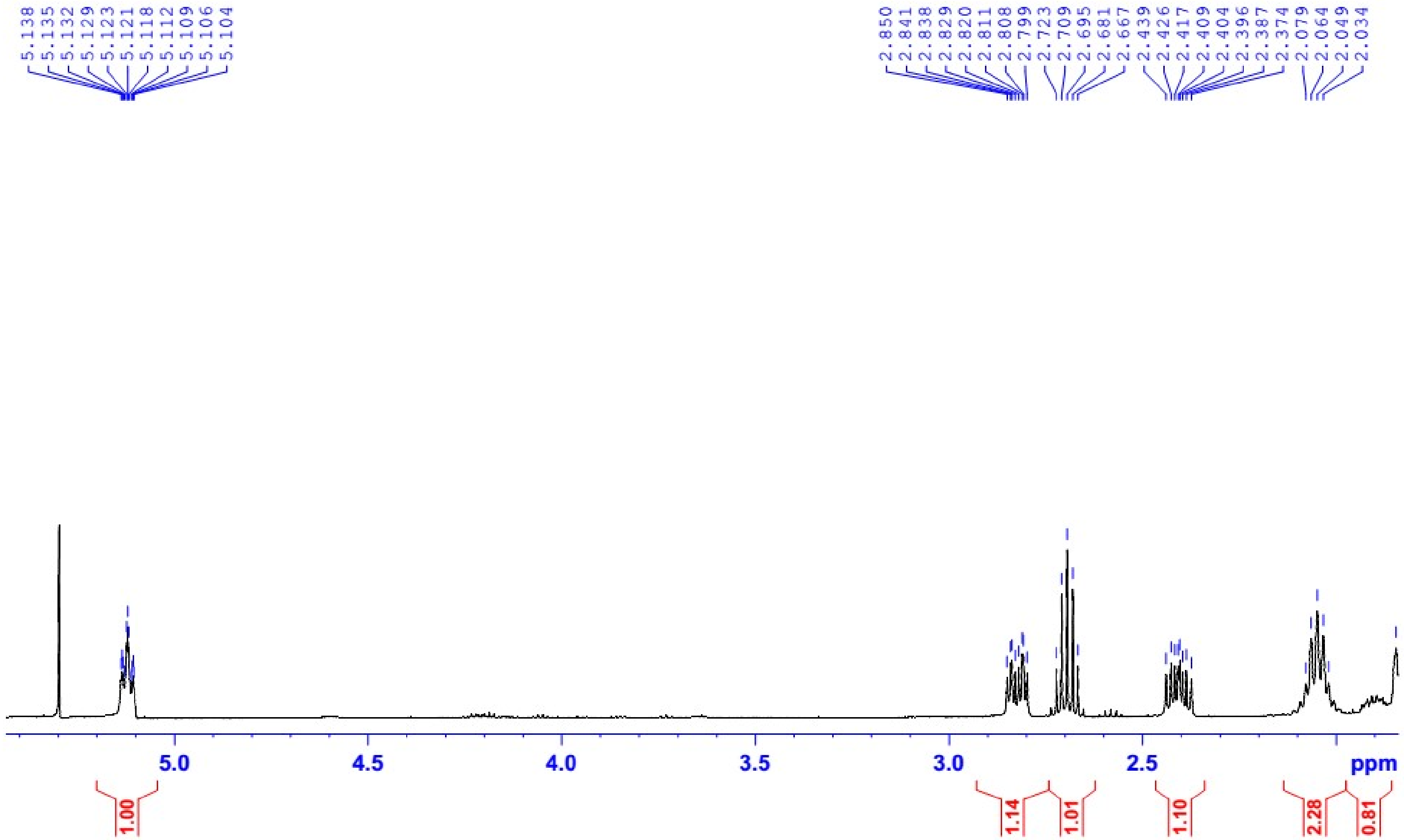


$^{1}$H NMR spectrum of compound **3m** (extension)

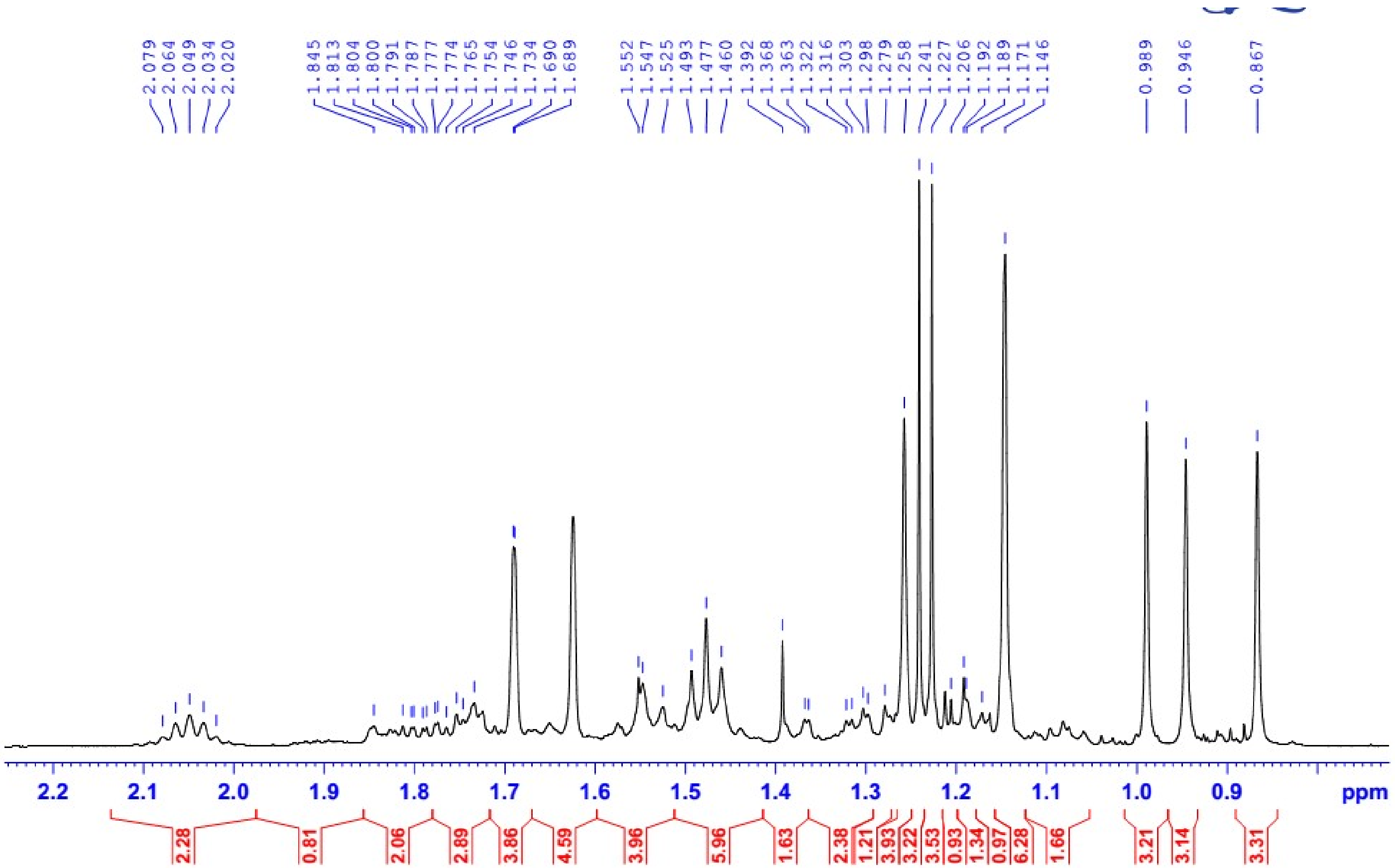

$^{1}$H NMR spectrum of compound **3m** (extension)

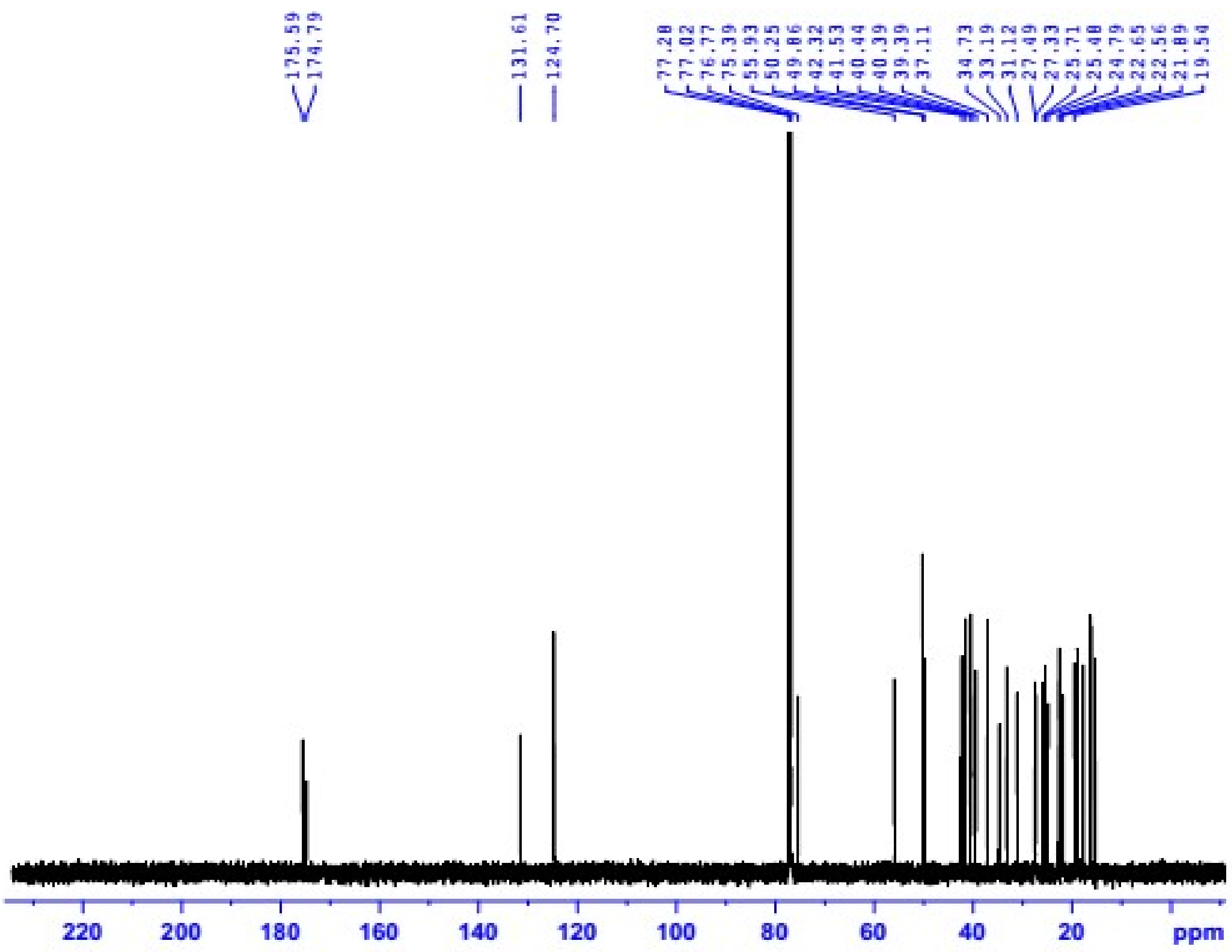


$^{13}C$-NMR spectrum of compound **3m**

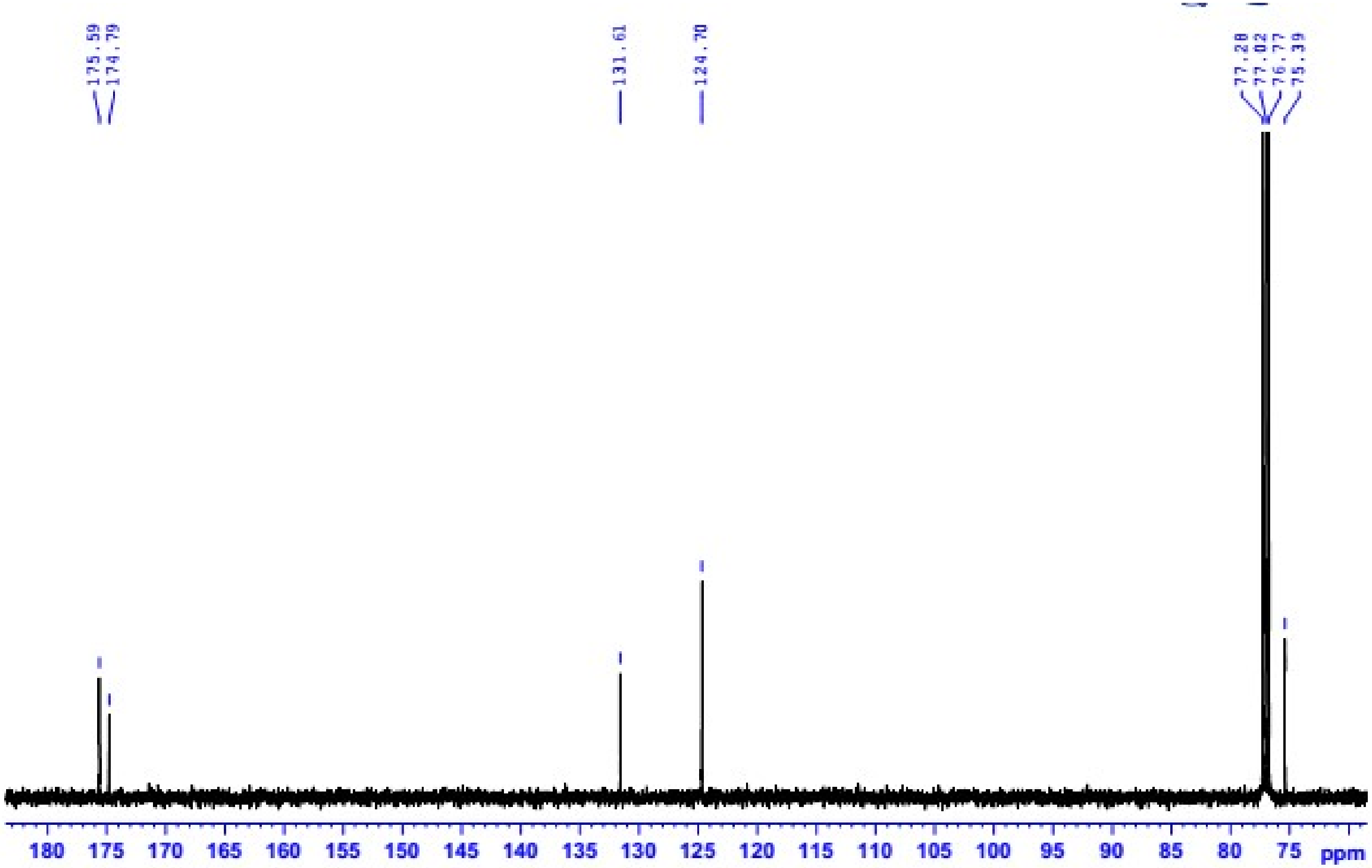


$^{13}C$-NMR spectrum of compound **3m** (extension)

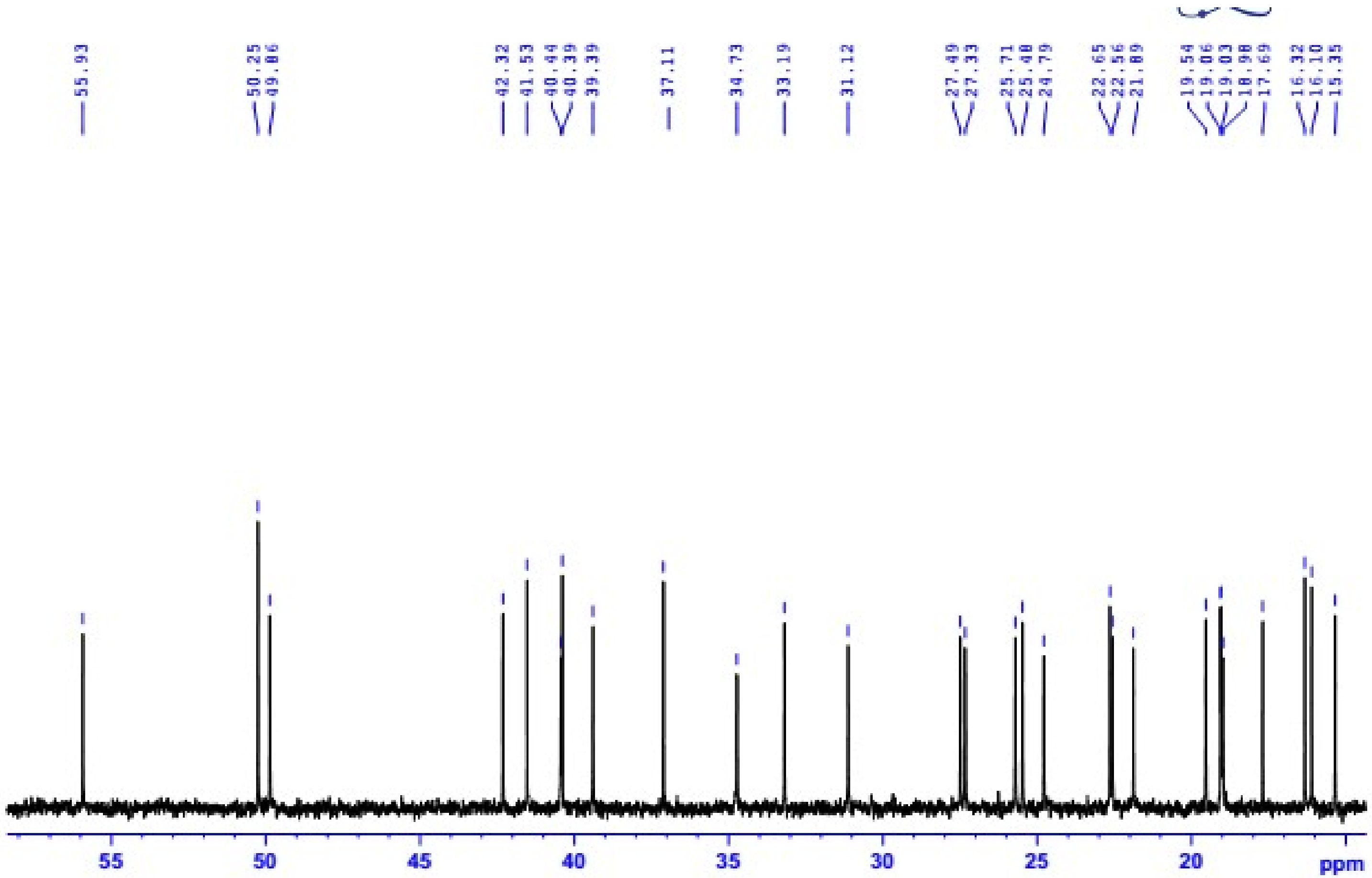


$^{13}$C-NMR spectrum of compound **3m** (extention)

## 1.15. Compound 4

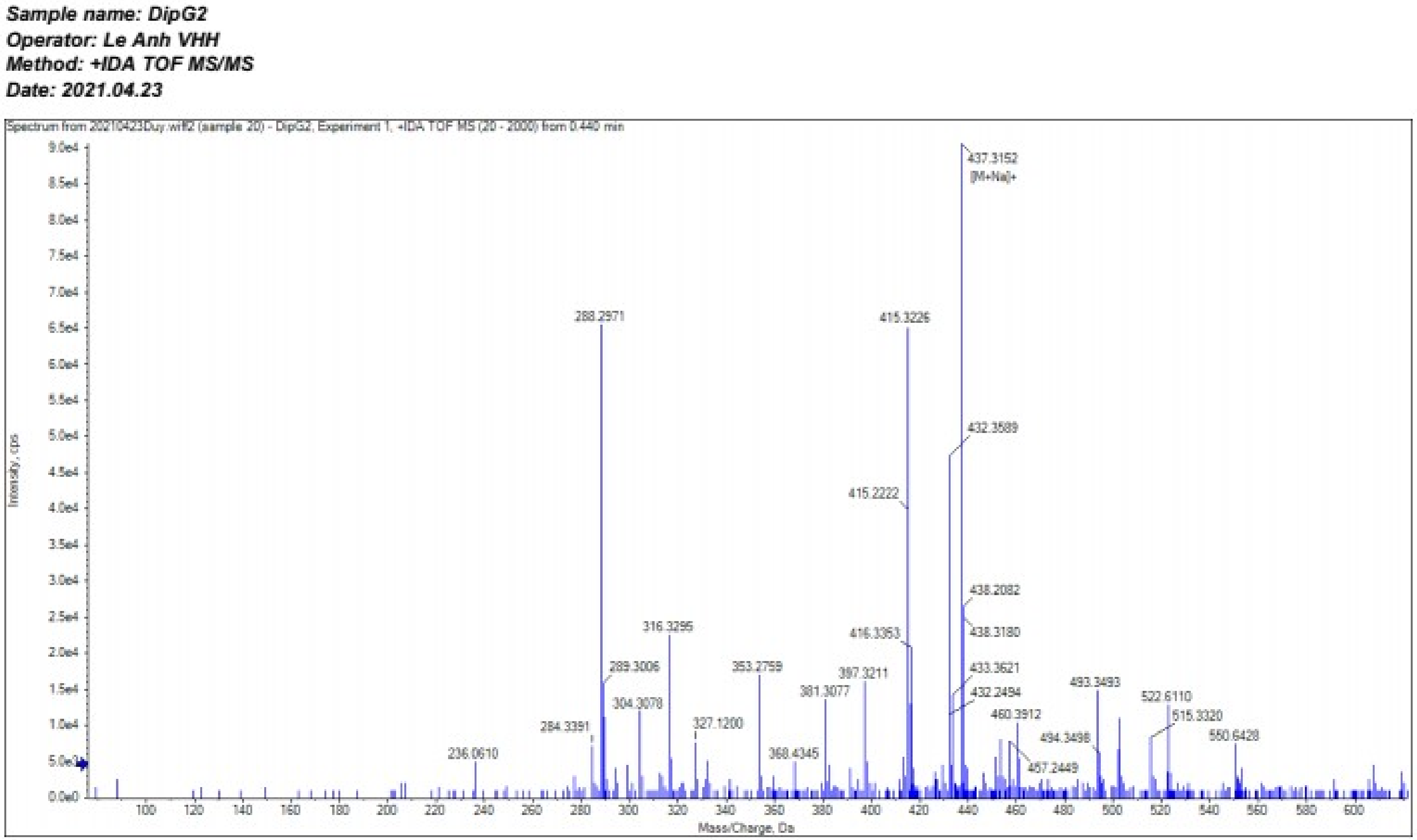


| Hit | Formula | m/z | RDB | ppm | MS Rank | MSMS ppm | MSMS Rank | Found |
|---|---|---|---|---|---|---|---|---|
| 1 | C27H42O3 | 415.32067 | 7.0 | 2.3 | 1 | | | NA/NA |

(+)-HR-ESI-MS spectrum of compound **4**

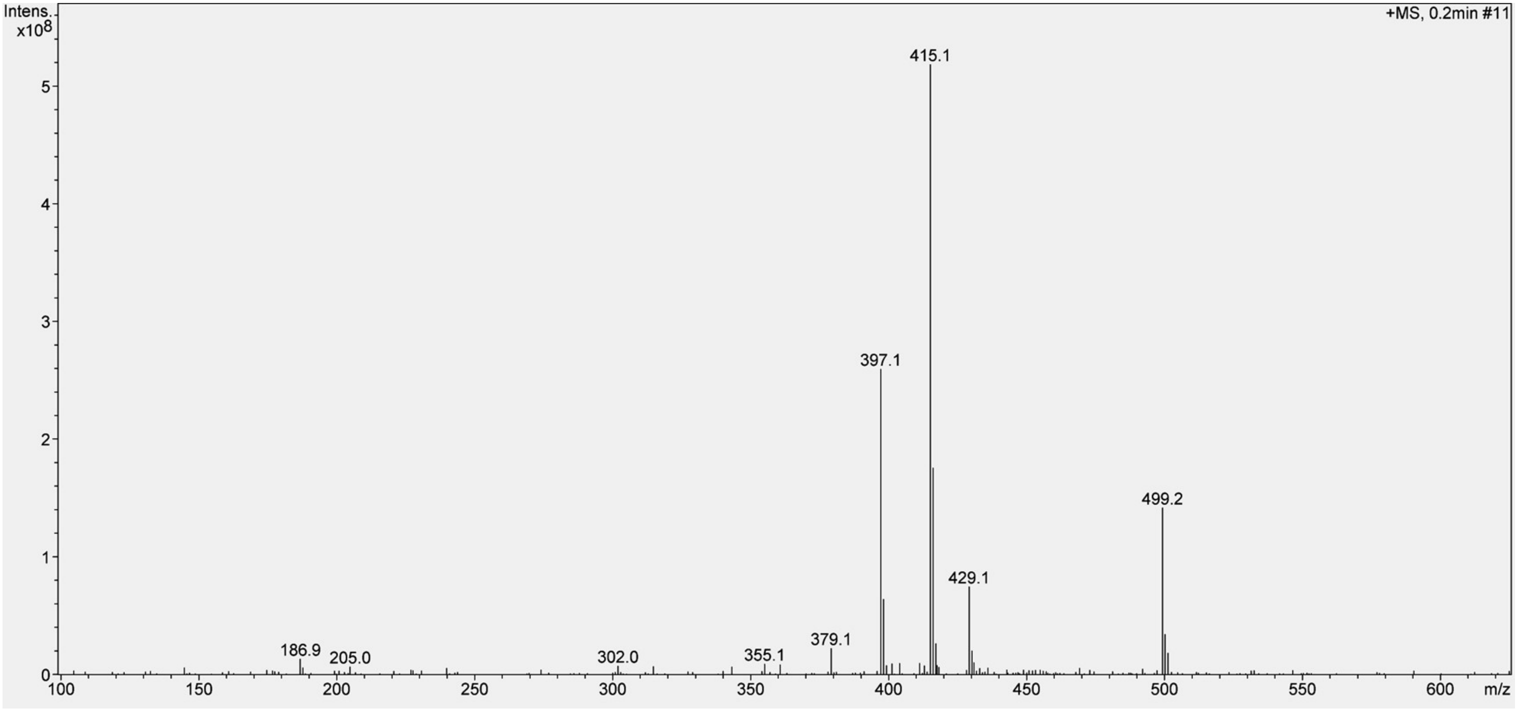


(+)-ESI-MS spectrum of compound **4**

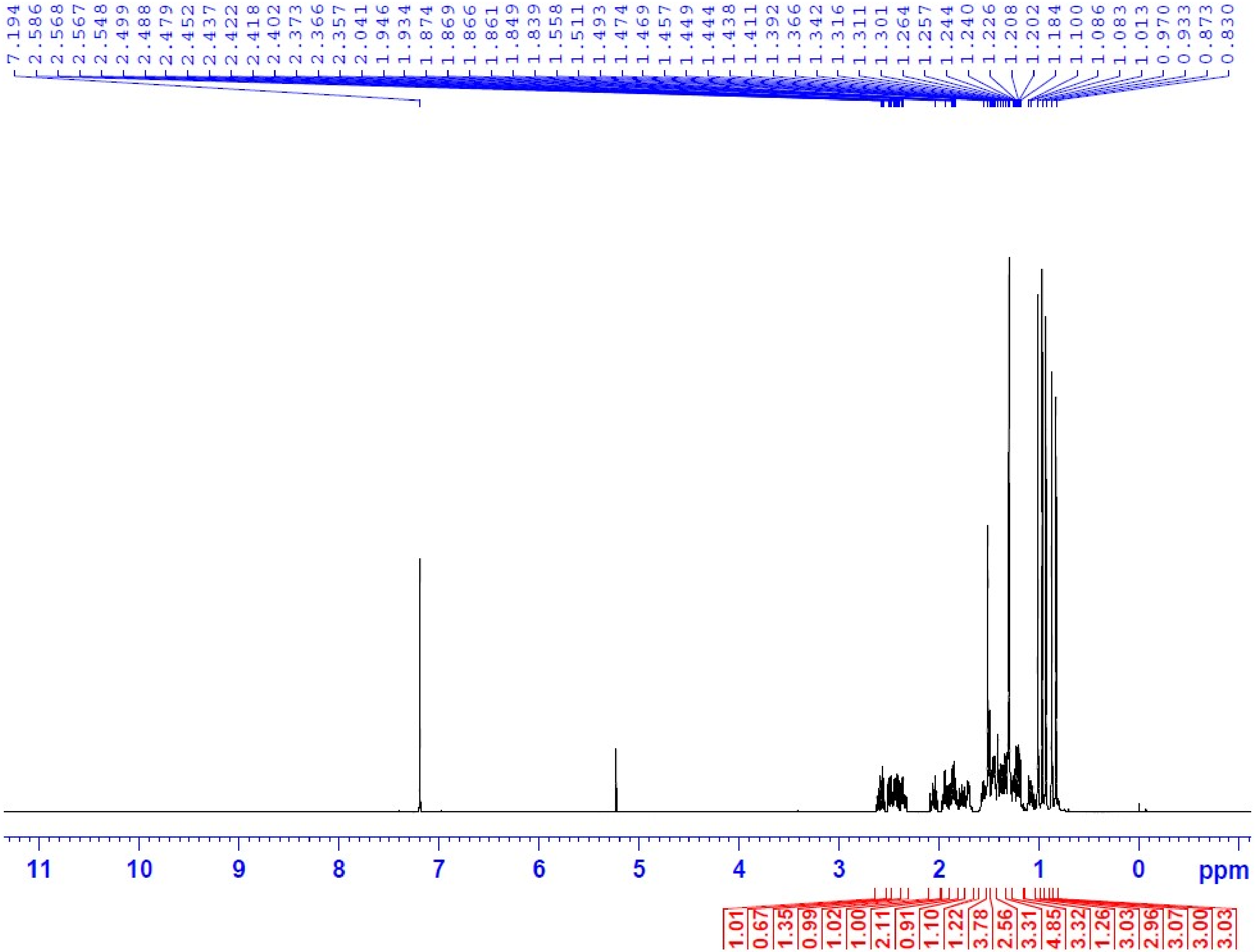

$^{1}$H-NMR spectrum of compound **4**

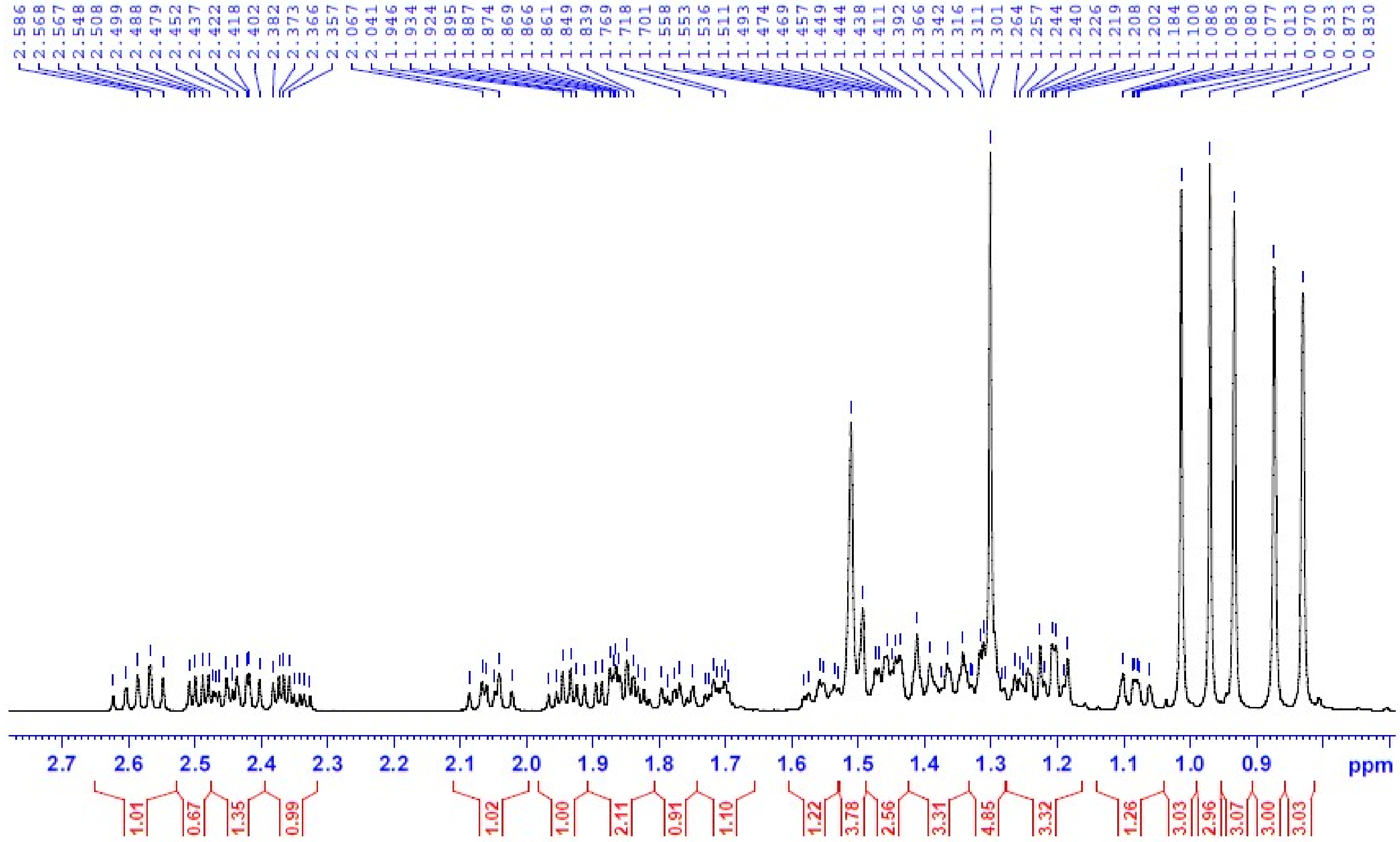

$^{1}$H-NMR spectrum of compound **4** (extension)

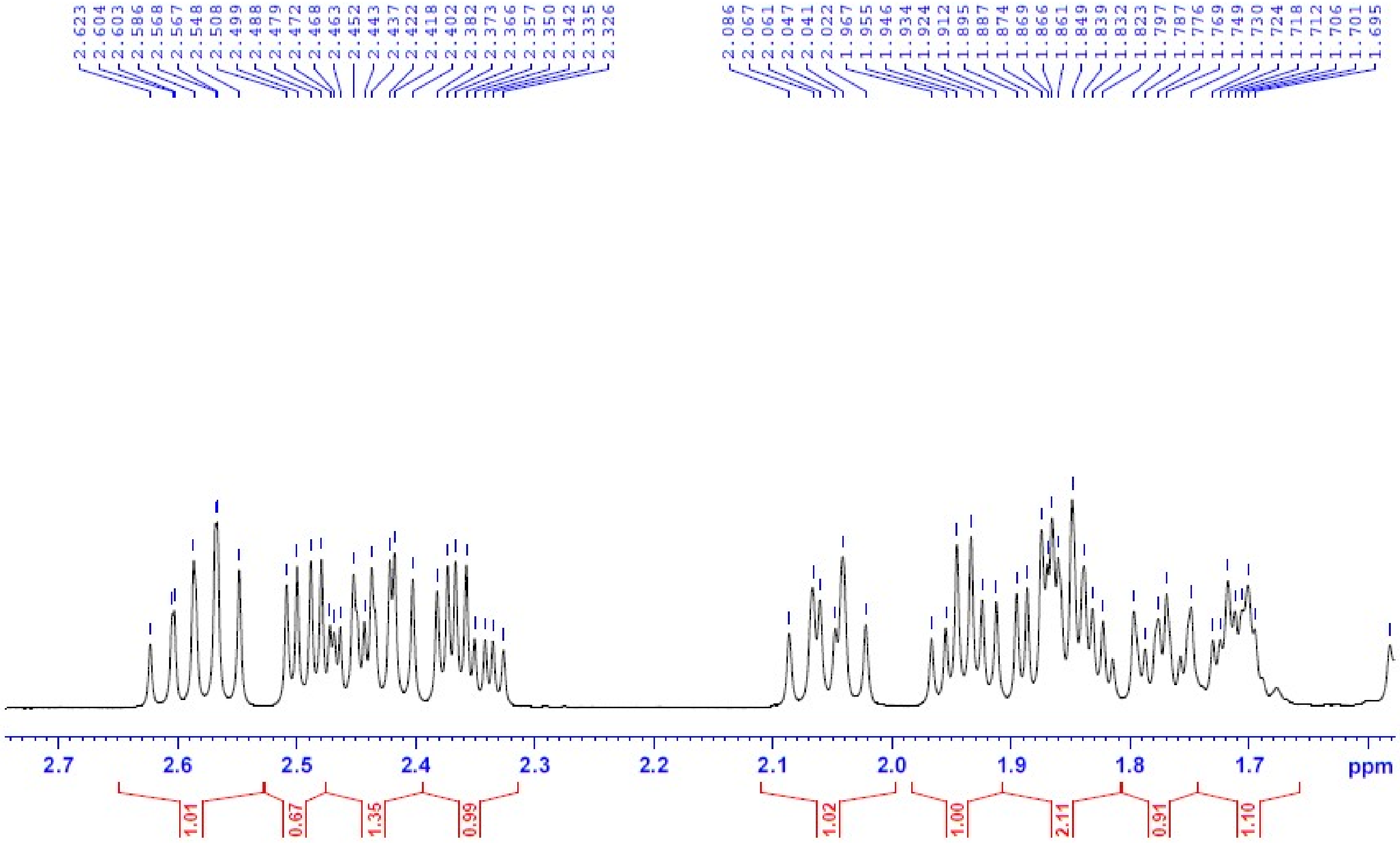
2.623 2.604 2.603 2.586 2.568 2.567 2.548 2.508 2.499 2.488 2.479 2.472 2.468 2.463 2.452 2.443 2.437 2.422 2.418 2.402 2.382 2.373 2.366 2.357 2.350 2.342 2.335 2.326
2.086 2.067 2.061 2.047 2.041 2.022 1.967 1.955 1.946 1.934 1.924 1.912 1.895 1.887 1.874 1.869 1.866 1.861 1.849 1.839 1.832 1.823 1.797 1.787 1.776 1.769 1.749 1.730 1.724 1.718 1.712 1.706 1.701 1.695
2.7 2.6 2.5 2.4 2.3 2.2 2.1 2.0 1.9 1.8 1.7 ppm
1.01 0.67 1.35 0.99 1.02 1.00 2.11 0.91 1.10

$^{1}$H-NMR spectrum of compound **4** (extension)

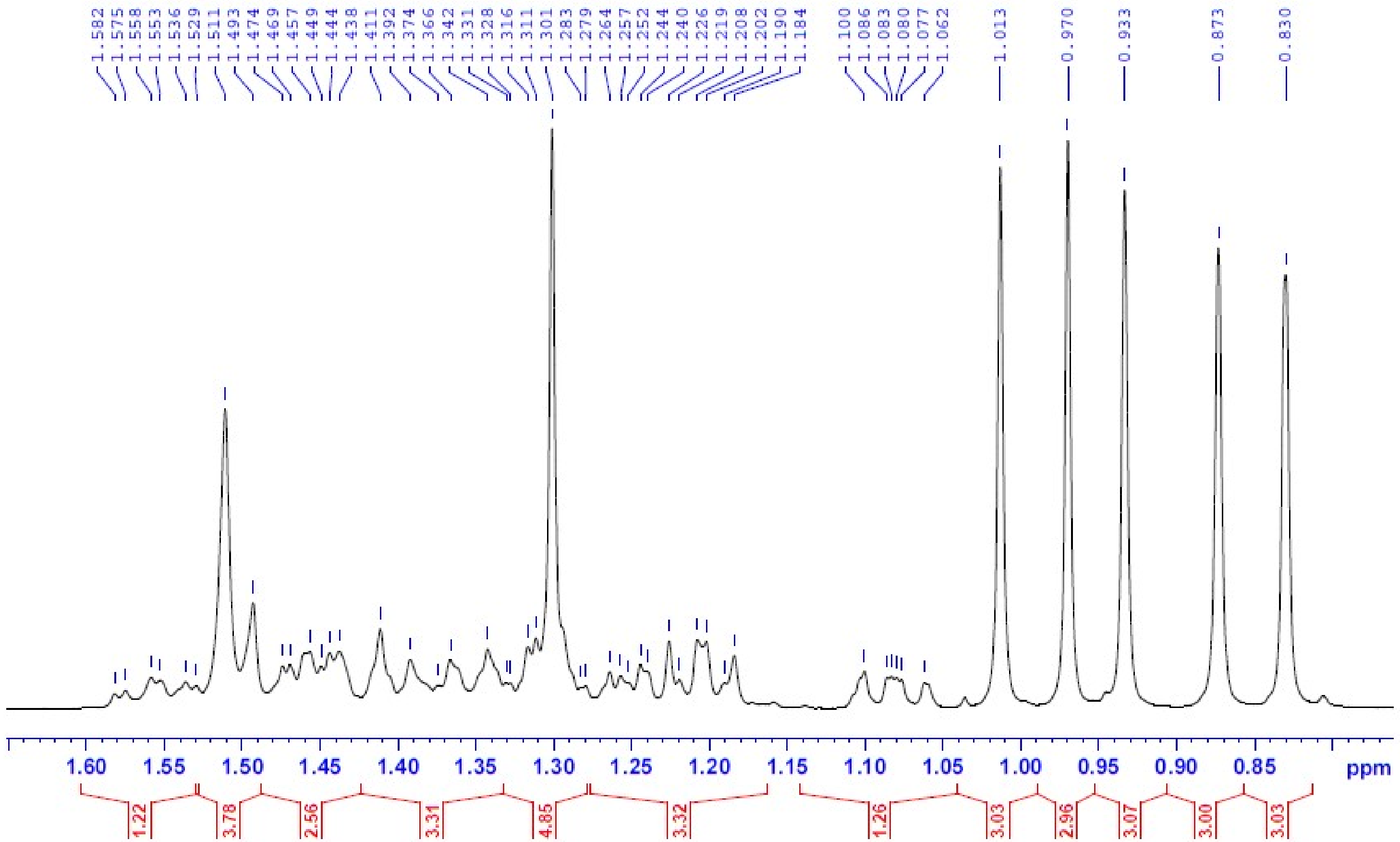
1.582
1.575
1.558
1.553
1.536
1.529
1.511
1.493
1.474
1.469
1.457
1.449
1.444
1.438
1.411
1.392
1.374
1.366
1.342
1.331
1.328
1.316
1.311
1.301
1.283
1.279
1.264
1.257
1.252
1.244
1.240
1.226
1.219
1.208
1.202
1.190
1.184
1.100
1.086
1.083
1.080
1.077
1.062
1.013
0.970
0.933
0.873
0.830
1.60
1.55
1.50
1.45
1.40
1.35
1.30
1.25
1.20
1.15
1.10
1.05
1.00
0.95
0.90
0.85
ppm
1.22
3.78
2.56
3.31
4.85
3.32
1.26
3.03
2.96
3.07
3.00
3.03

$^{1}$H-NMR spectrum of compound **4** (extension)

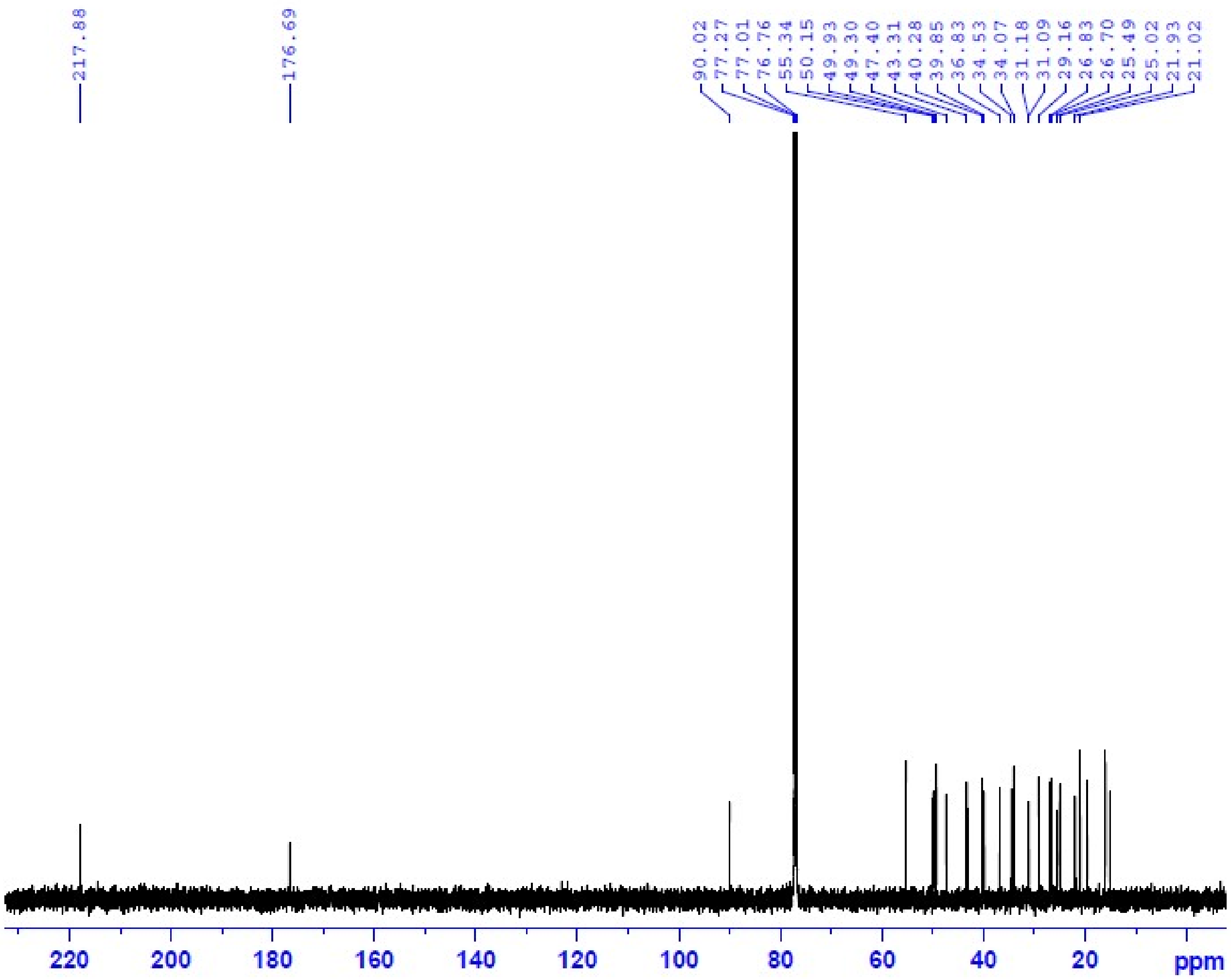


$^{13}$C-NMR spectrum of compound **4**

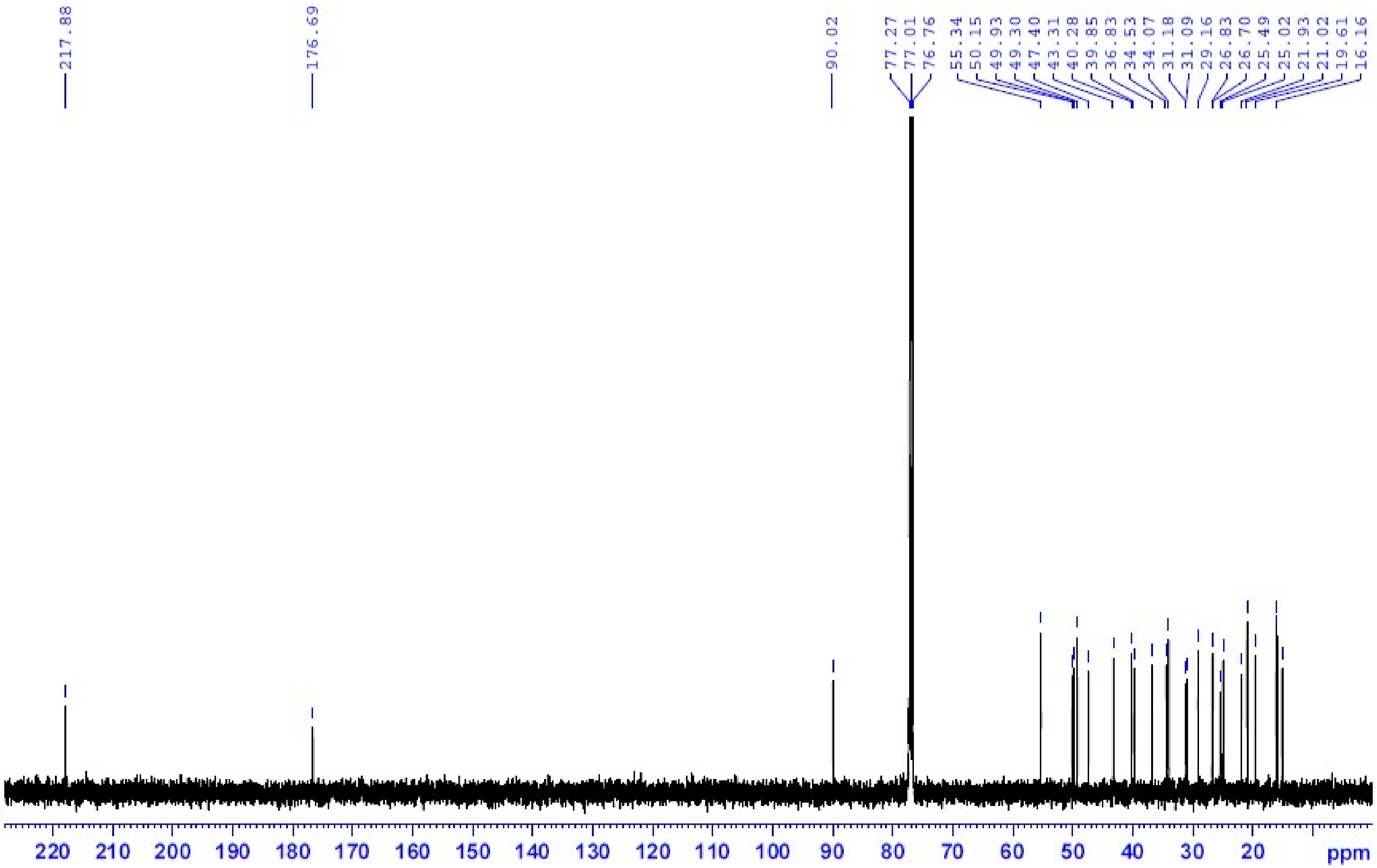
217.88
176.69
90.02
77.27
77.01
76.76
55.34
50.15
49.93
49.30
47.40
43.31
40.28
39.85
36.83
34.53
34.07
31.18
31.09
29.16
26.83
26.70
25.49
25.02
21.93
21.02
19.61
16.16
220 210 200 190 180 170 160 150 140 130 120 110 100 90 80 70 60 50 40 30 20 ppm

$^{13}$C-NMR spectrum of compound **4** (extension)

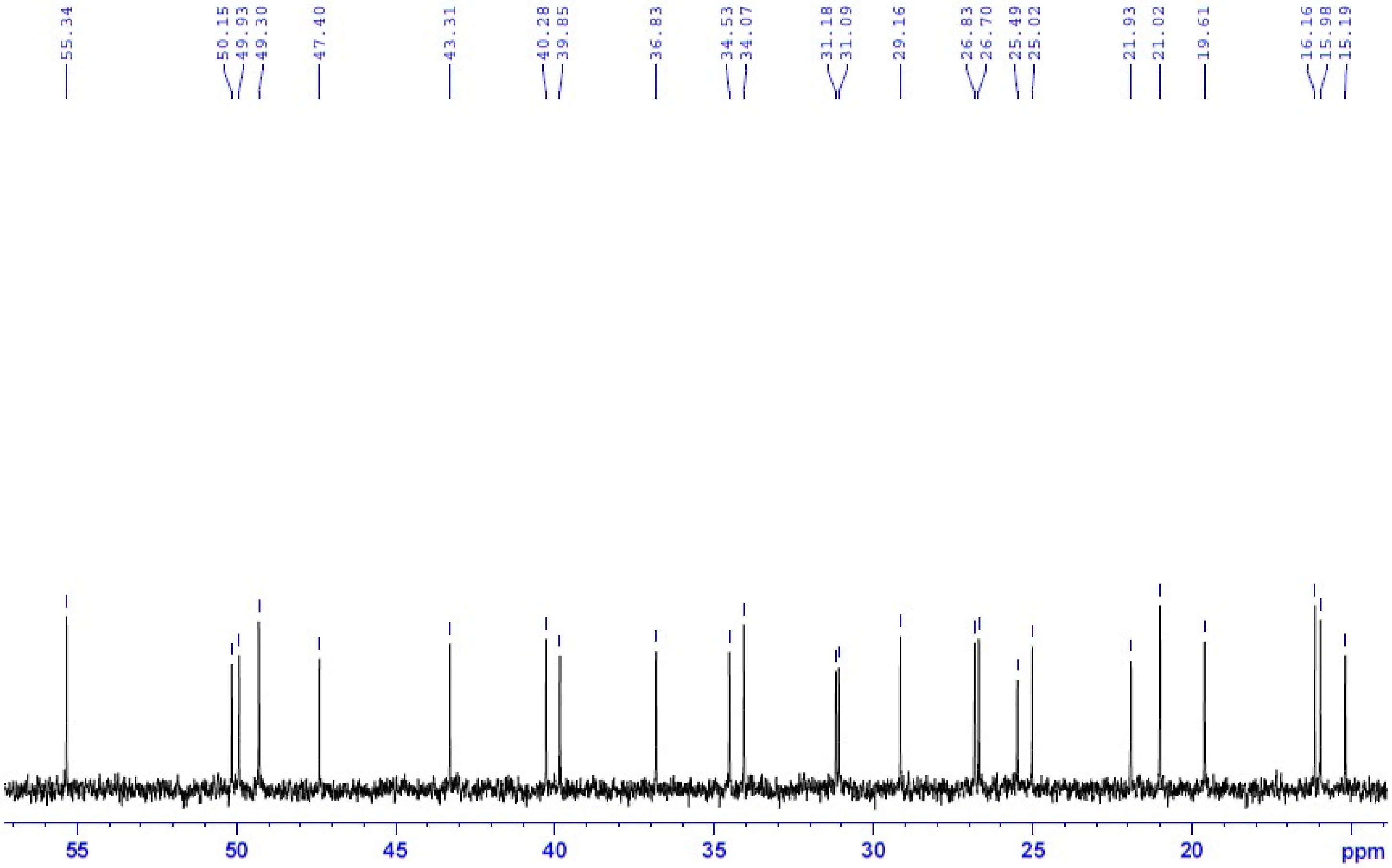
55.34
50.15
49.93
49.30
47.40
43.31
40.28
39.85
36.83
34.53
34.07
31.18
31.09
29.16
26.83
26.70
25.49
25.02
21.93
21.02
19.61
16.16
15.98
15.19
55
50
45
40
35
30
25
20
ppm

$^{13}C$-NMR spectrum of compound **4** (extension)

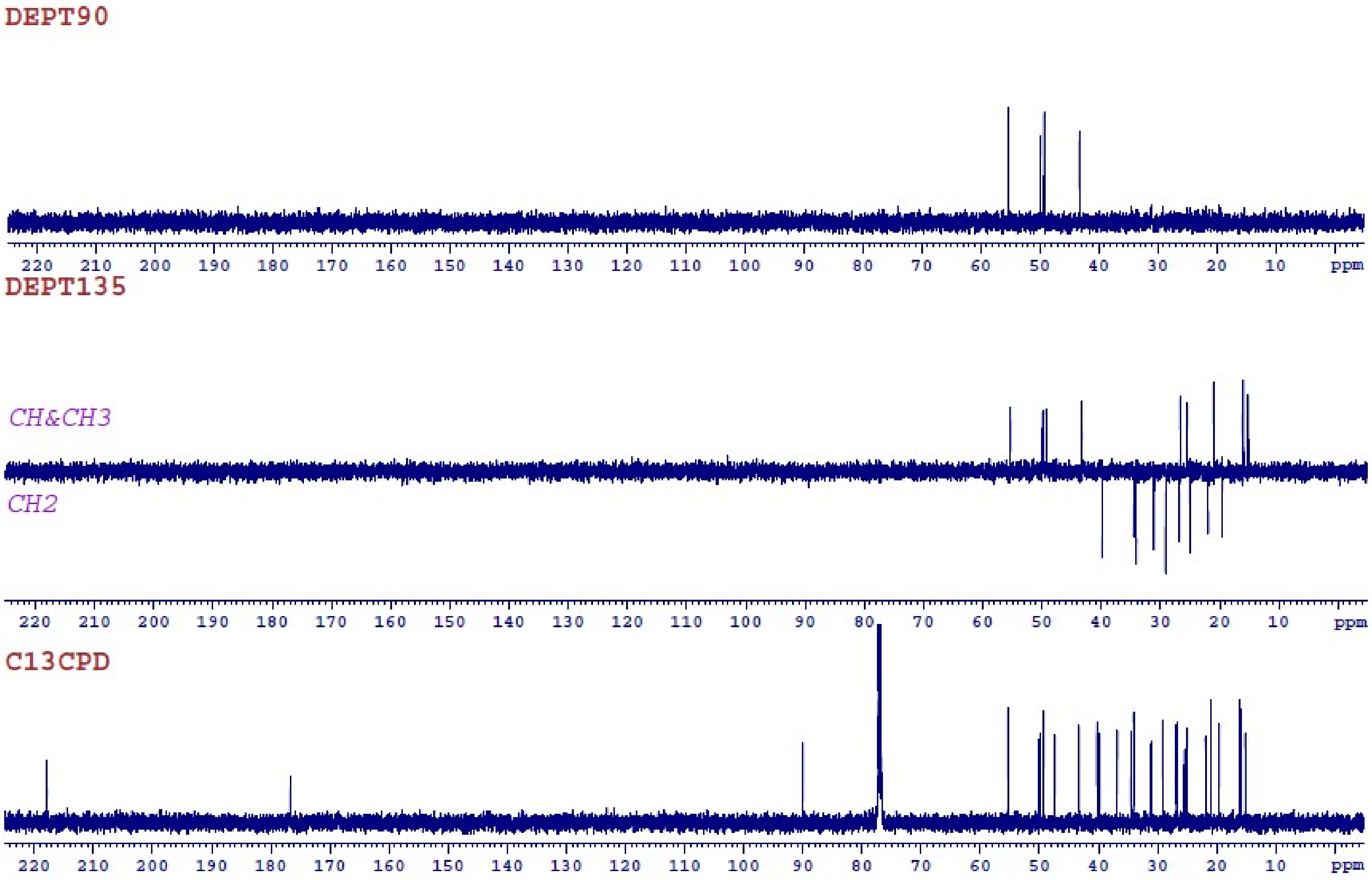


DEPT spectrum of compound **4**

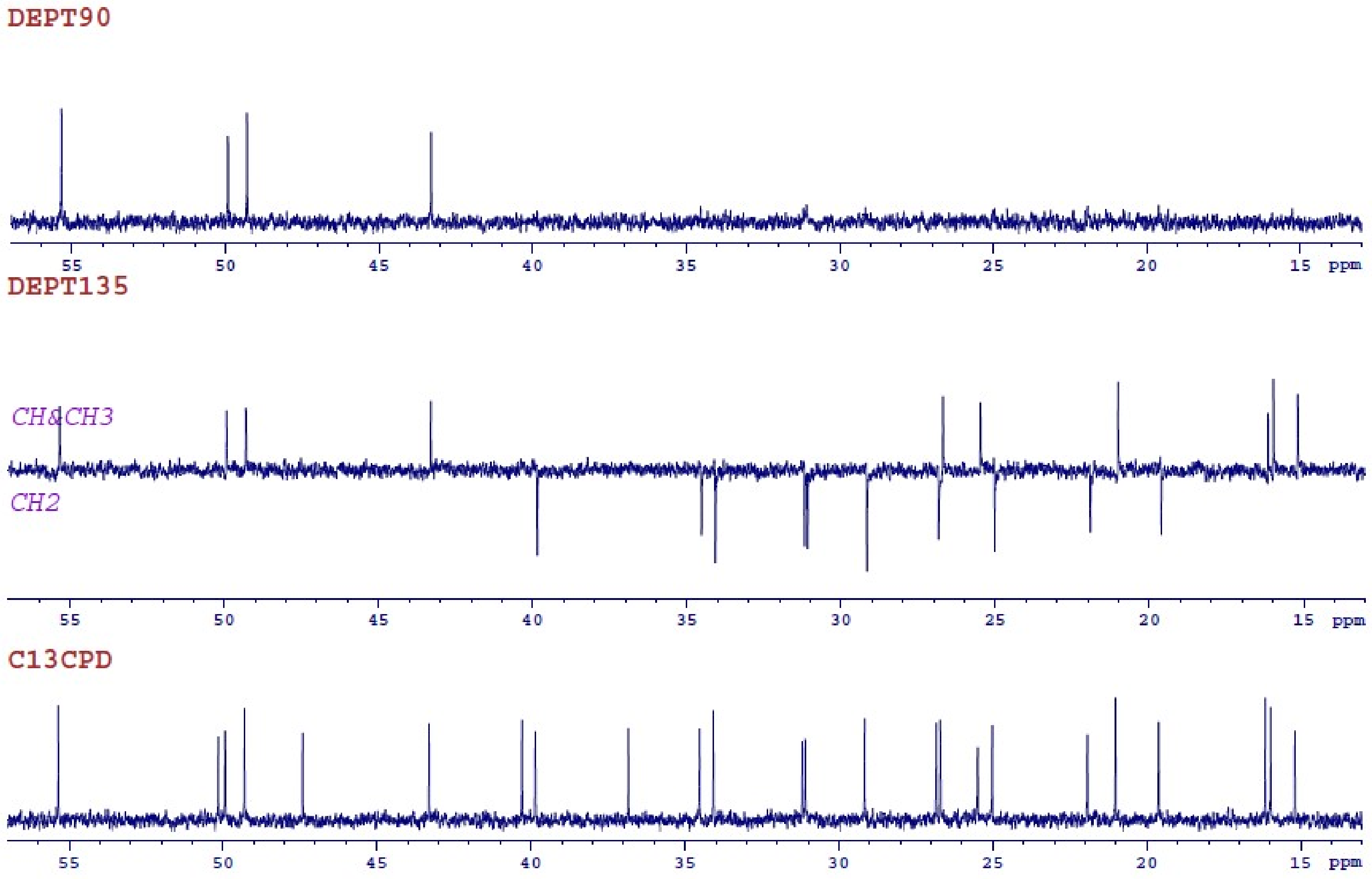


DEPT spectrum of compound **4** (extension)

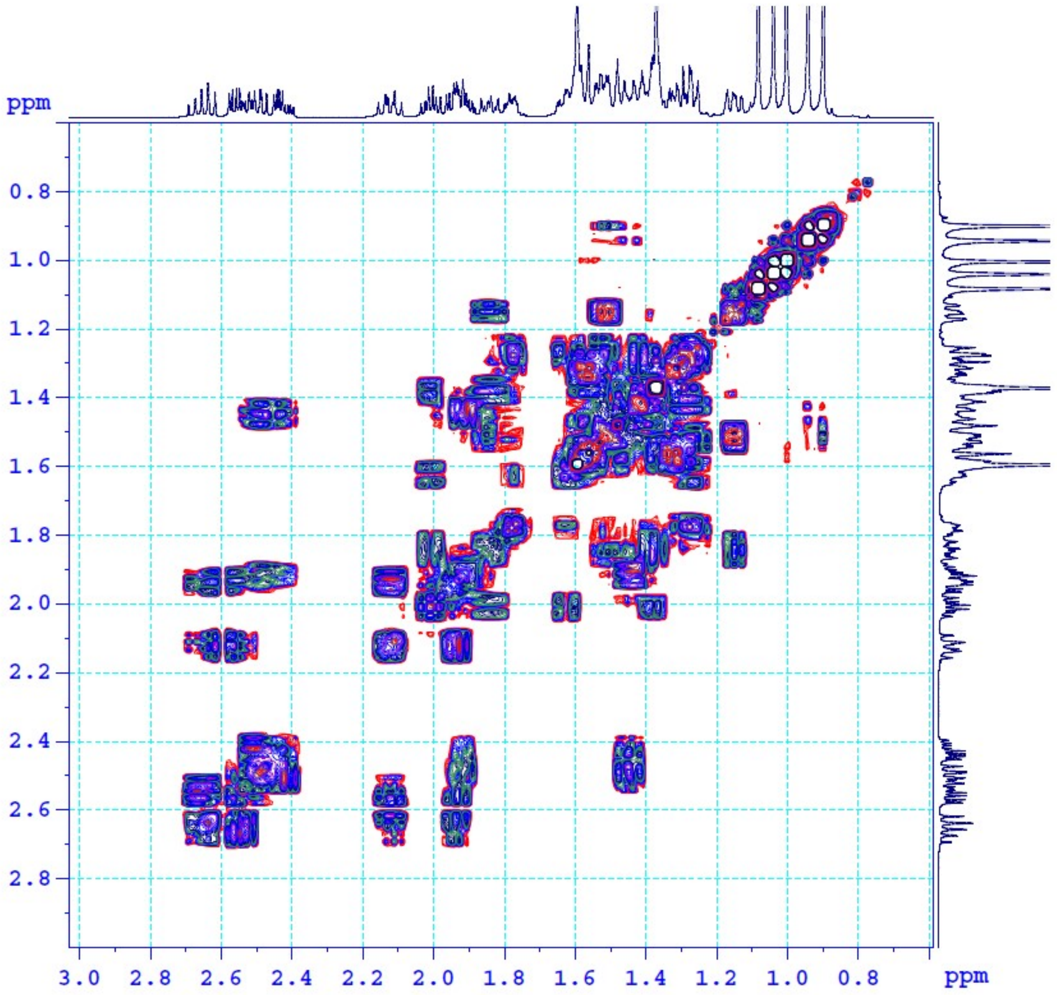


COSY spectrum of compound **4**

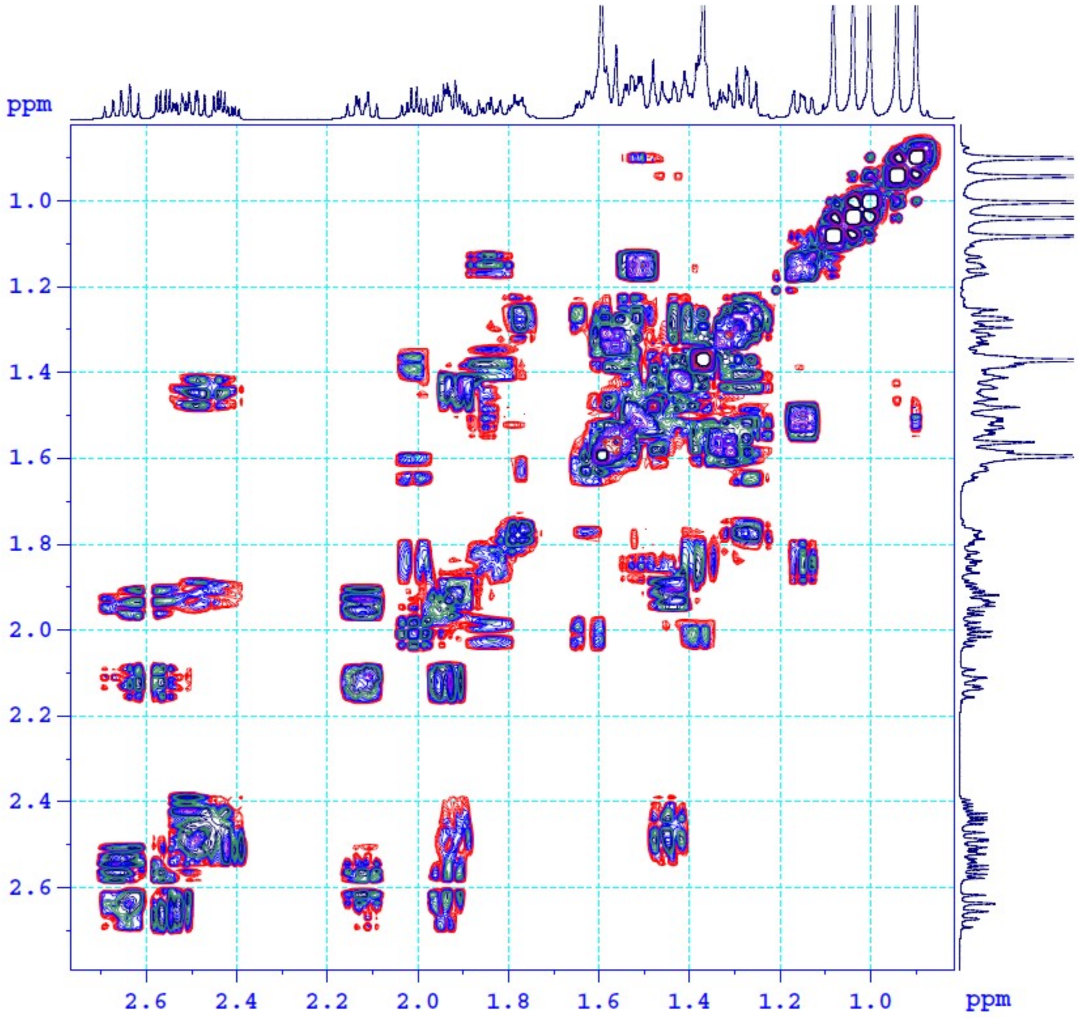


COSY spectrum of compound **4** (extension)

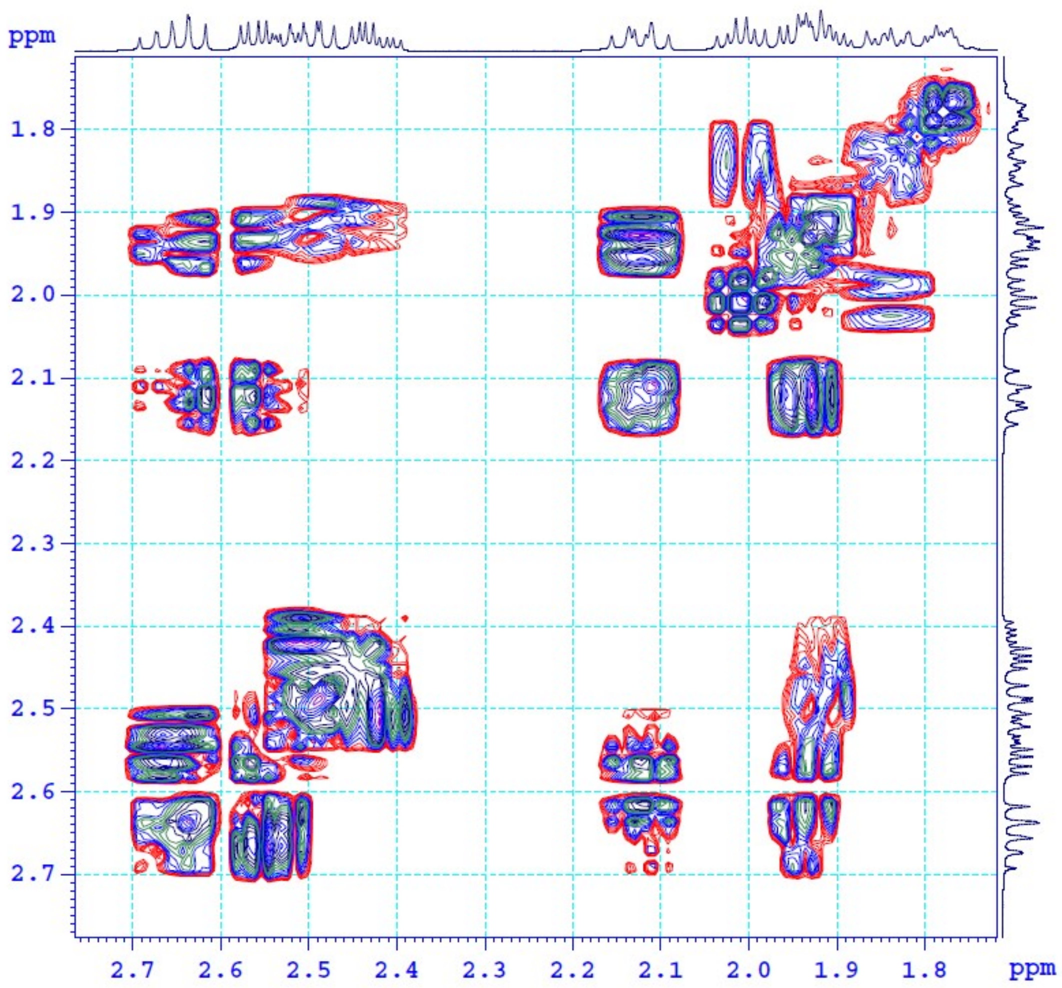


COSY spectrum of compound **4** (extension)

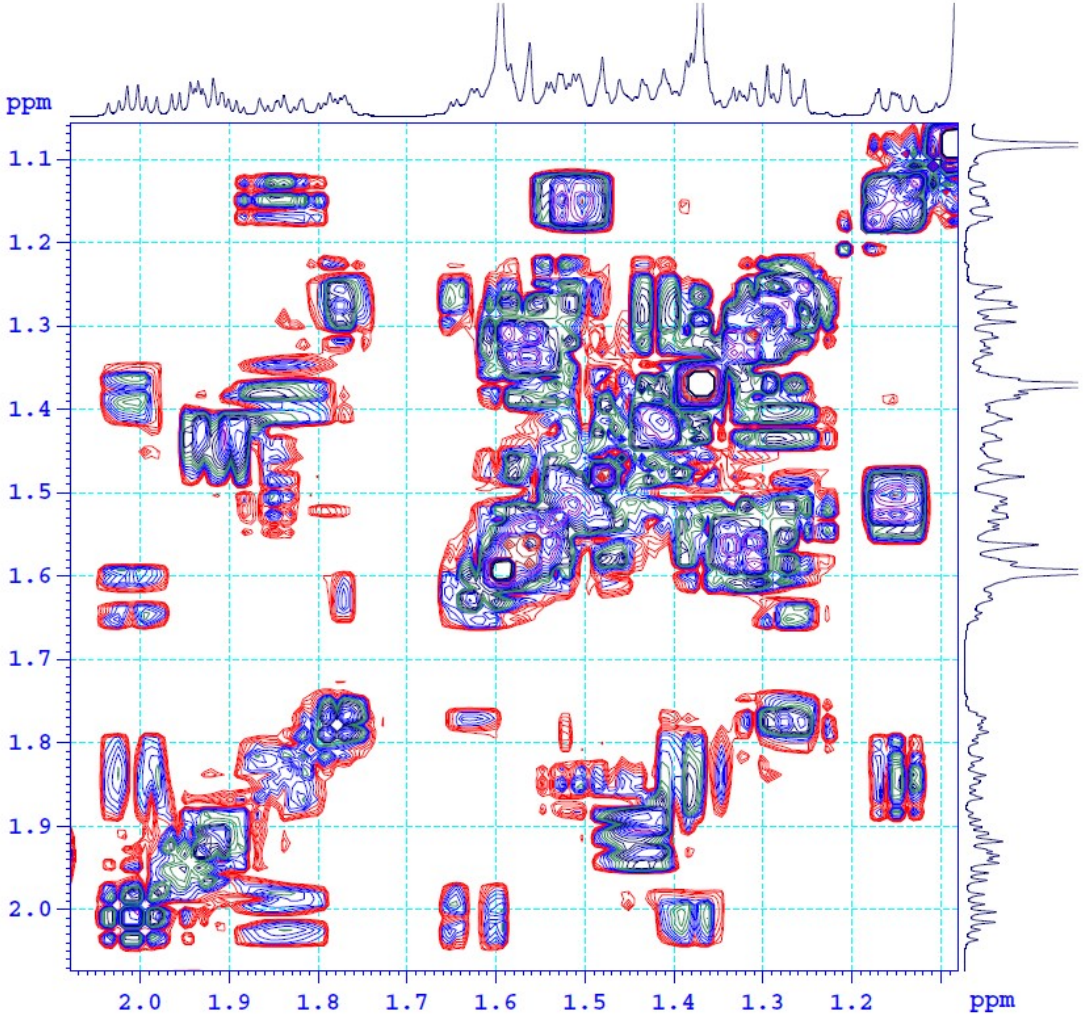


COSY spectrum of compound **4** (extension)

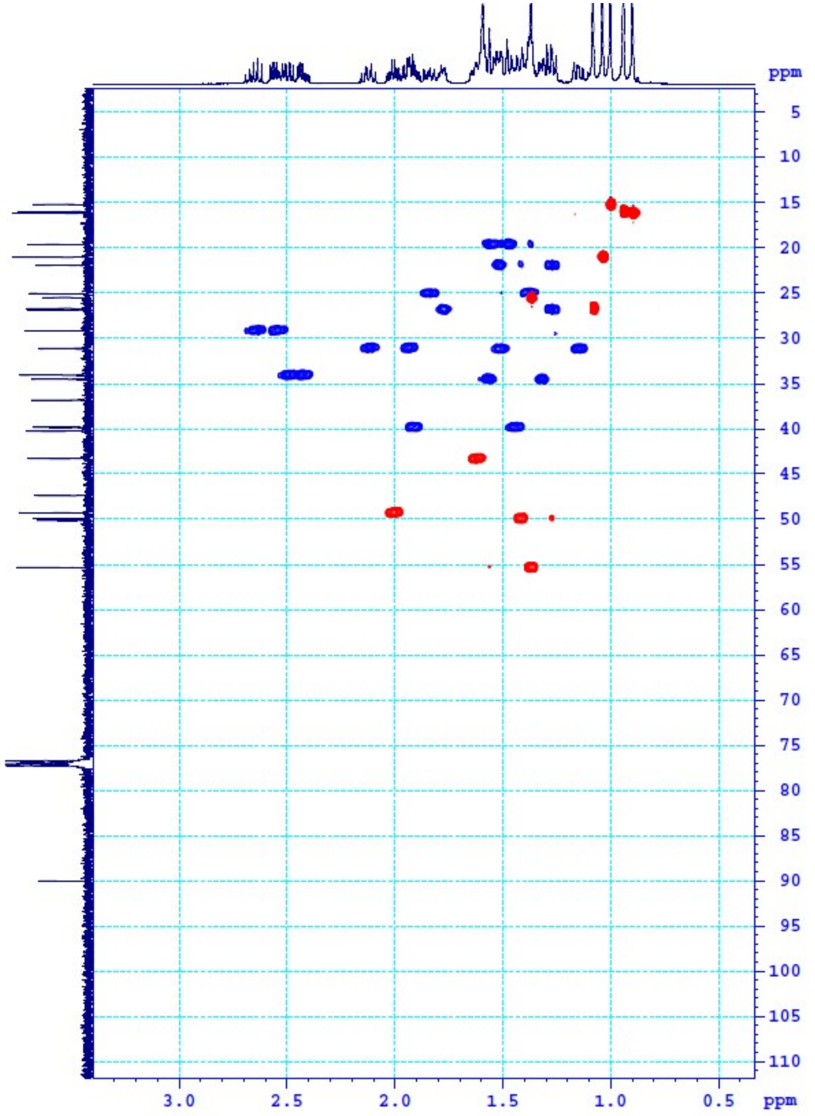


HSQC spectrum of compound **4**

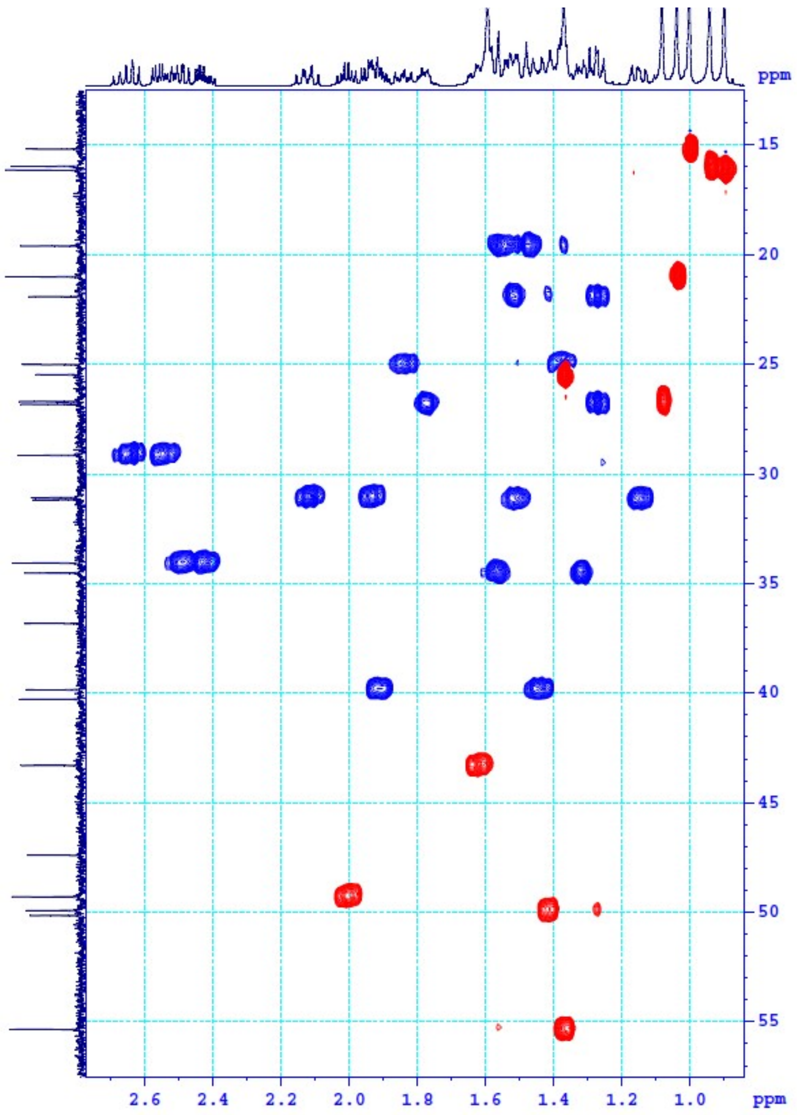


HSQC spectrum of compound **4** (extension)

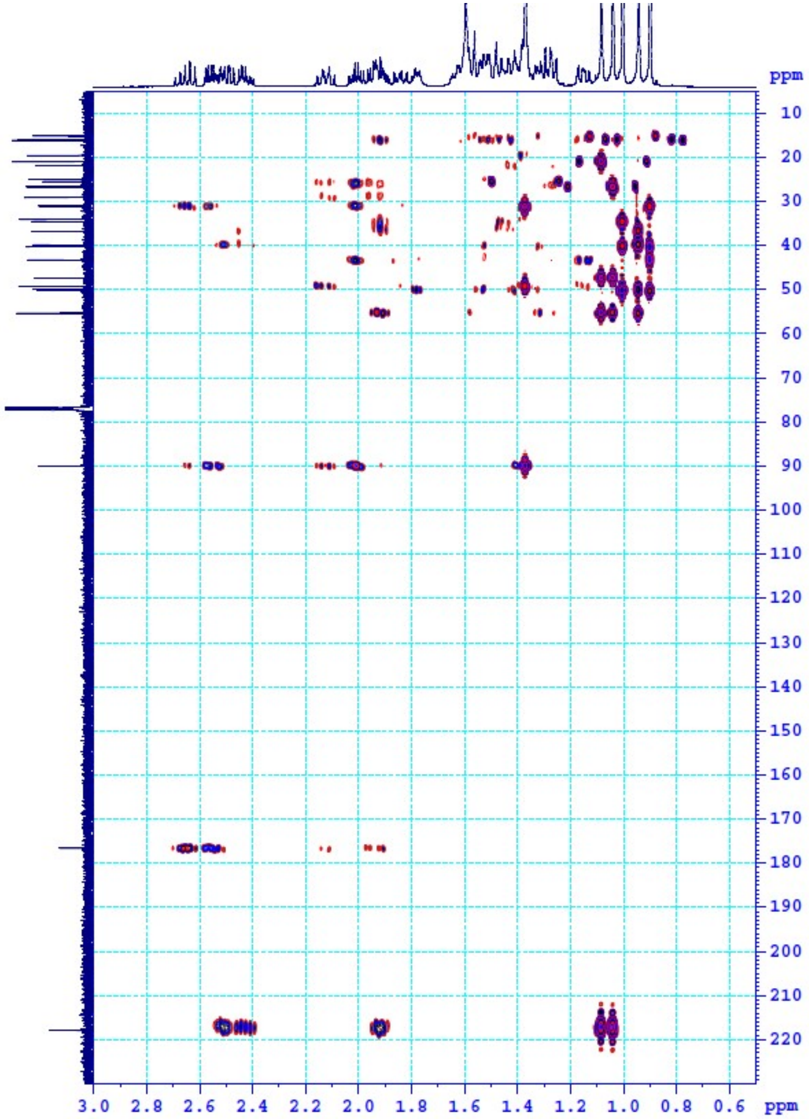


HMBC spectrum of compound 4

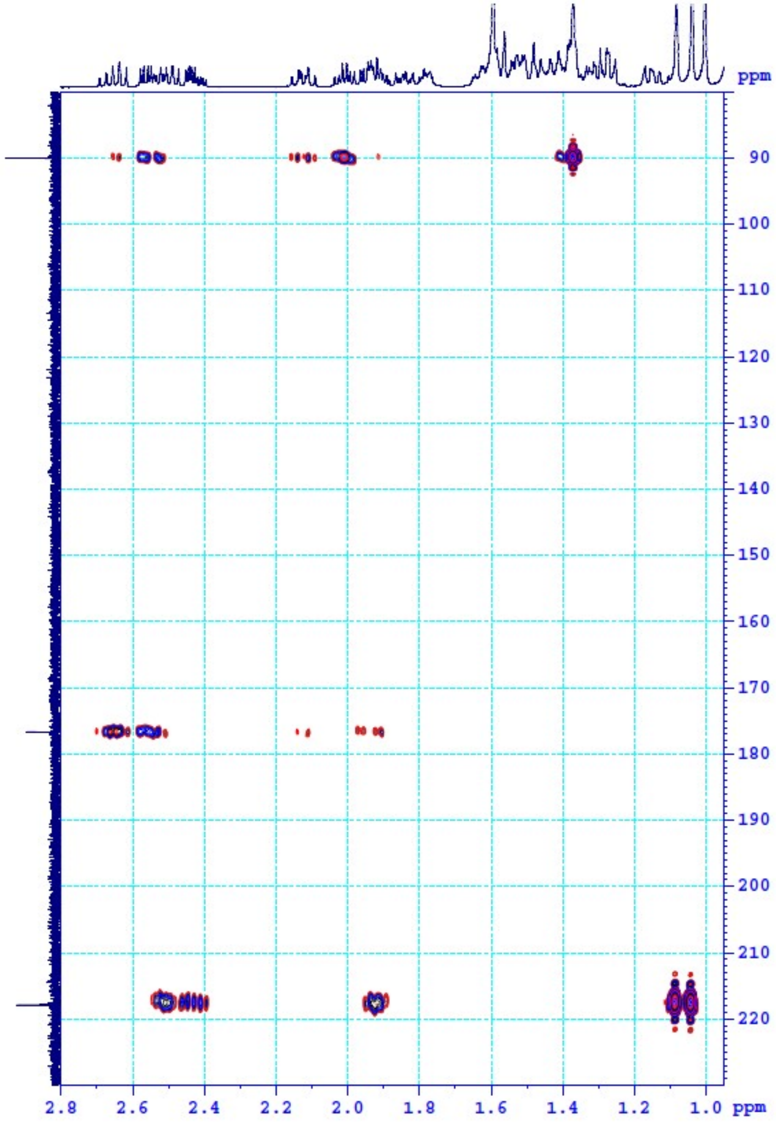


HMBC spectrum of compound **4** (extension)

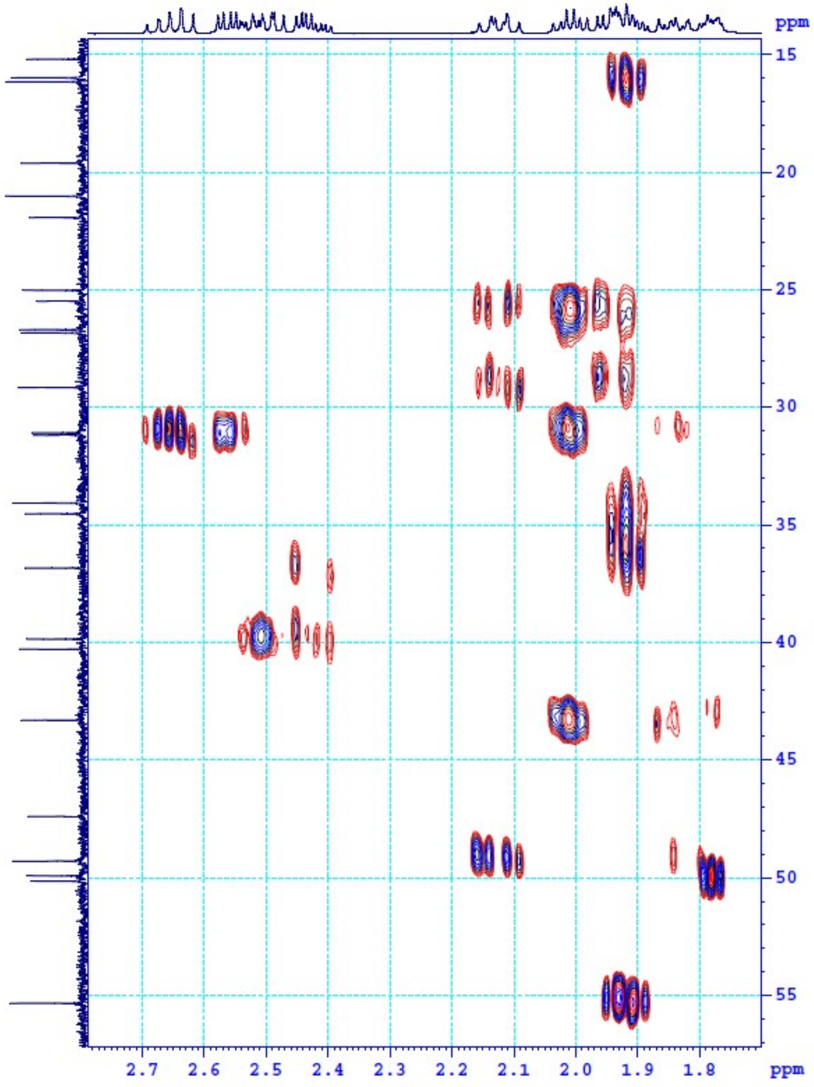


HMBC spectrum of compound **4** (extension)

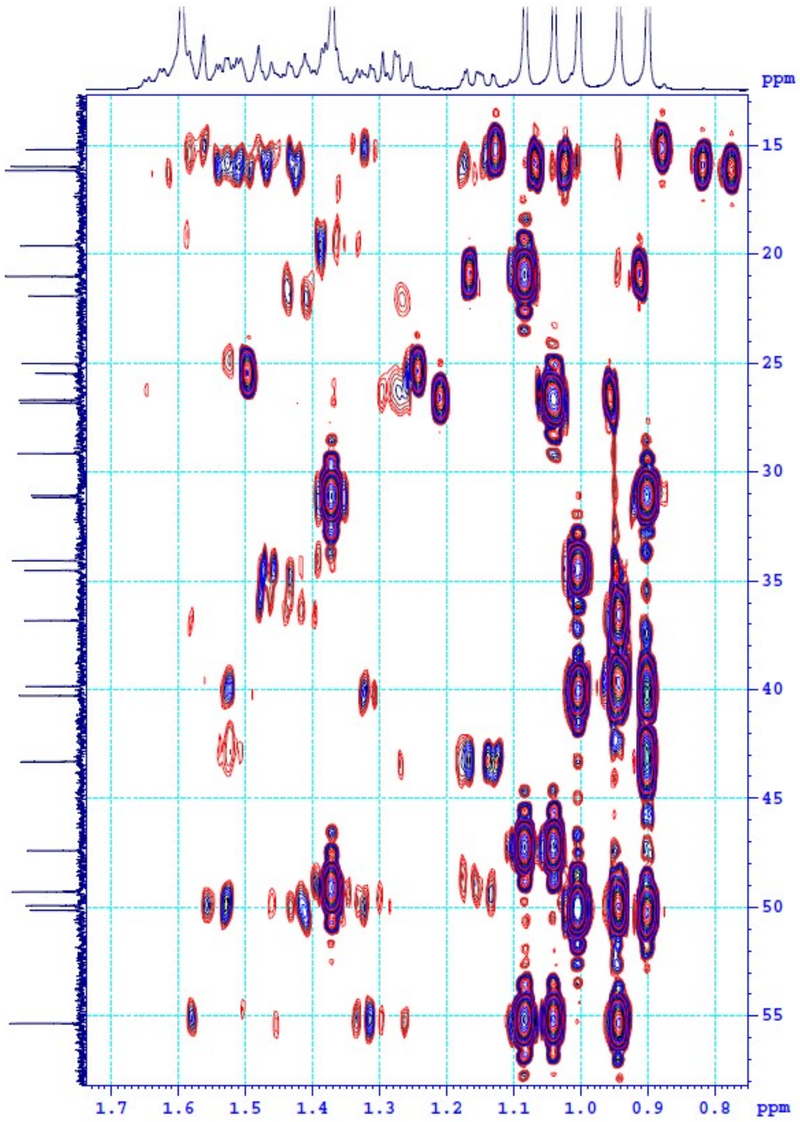


HMBC spectrum of compound **4** (extension)

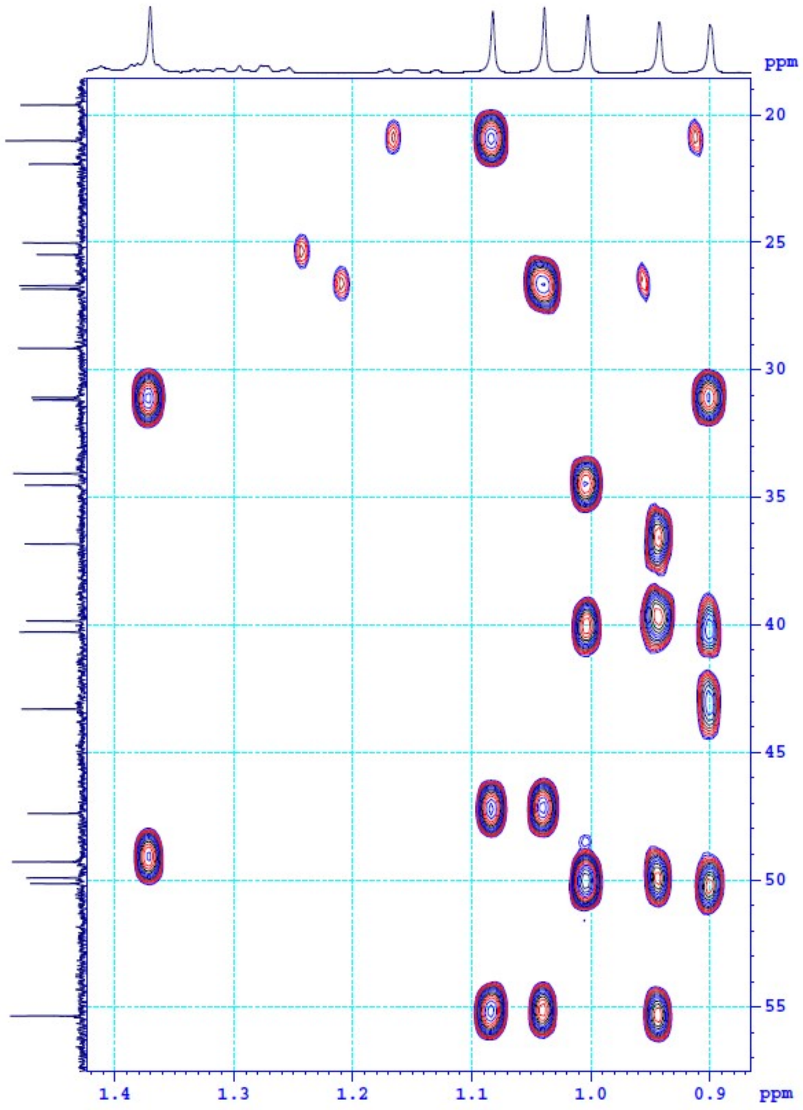


HMBC spectrum of compound **4** (extension)

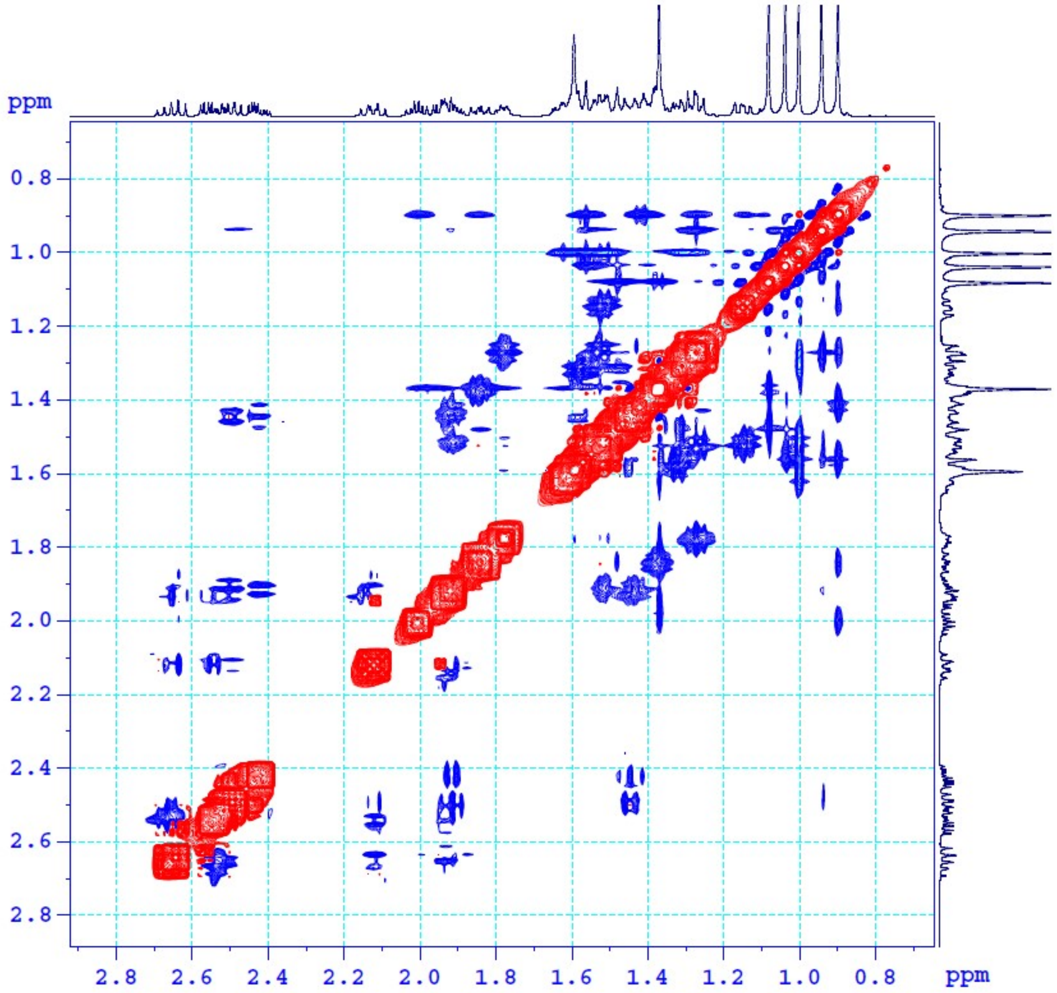


NOESY spectrum of compound **4**

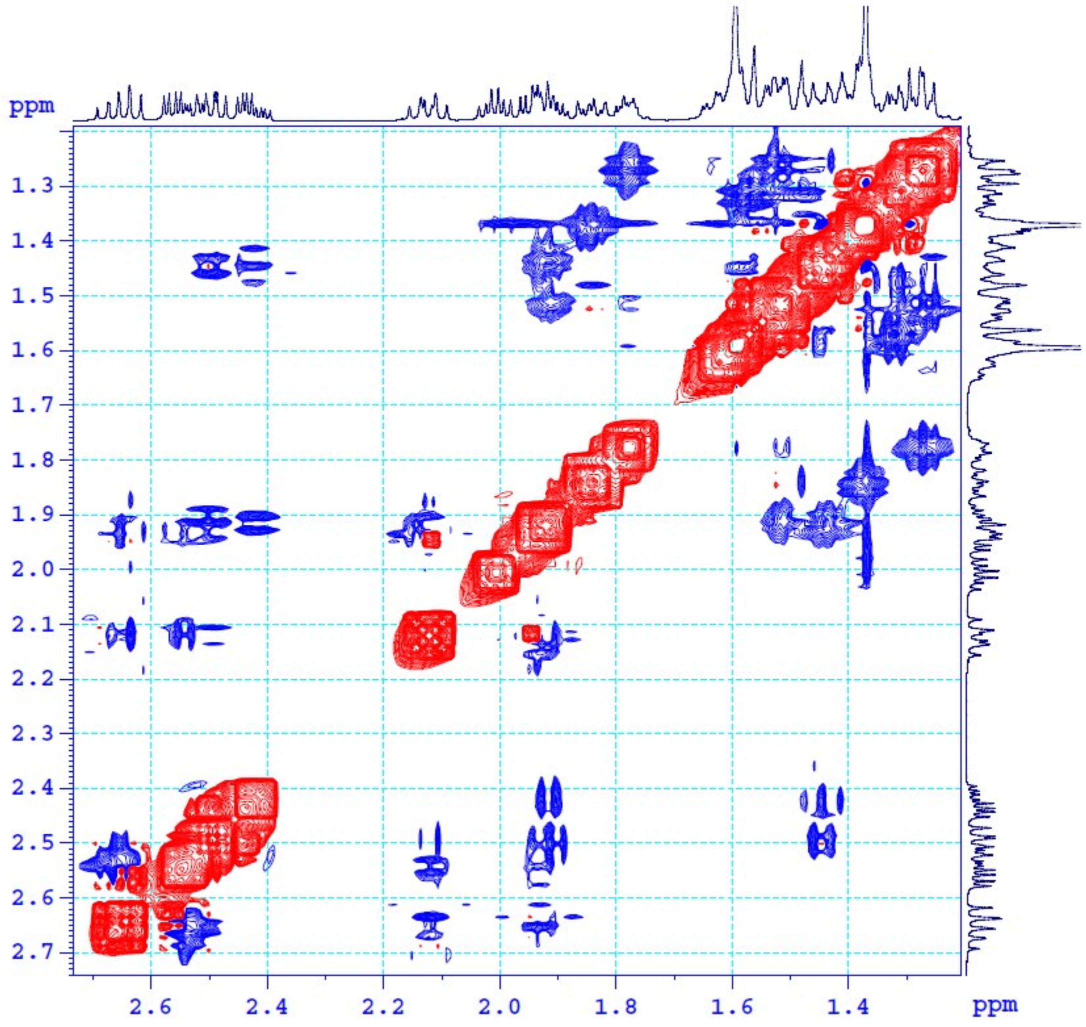


NOESY spectrum of compound **4** (extension)

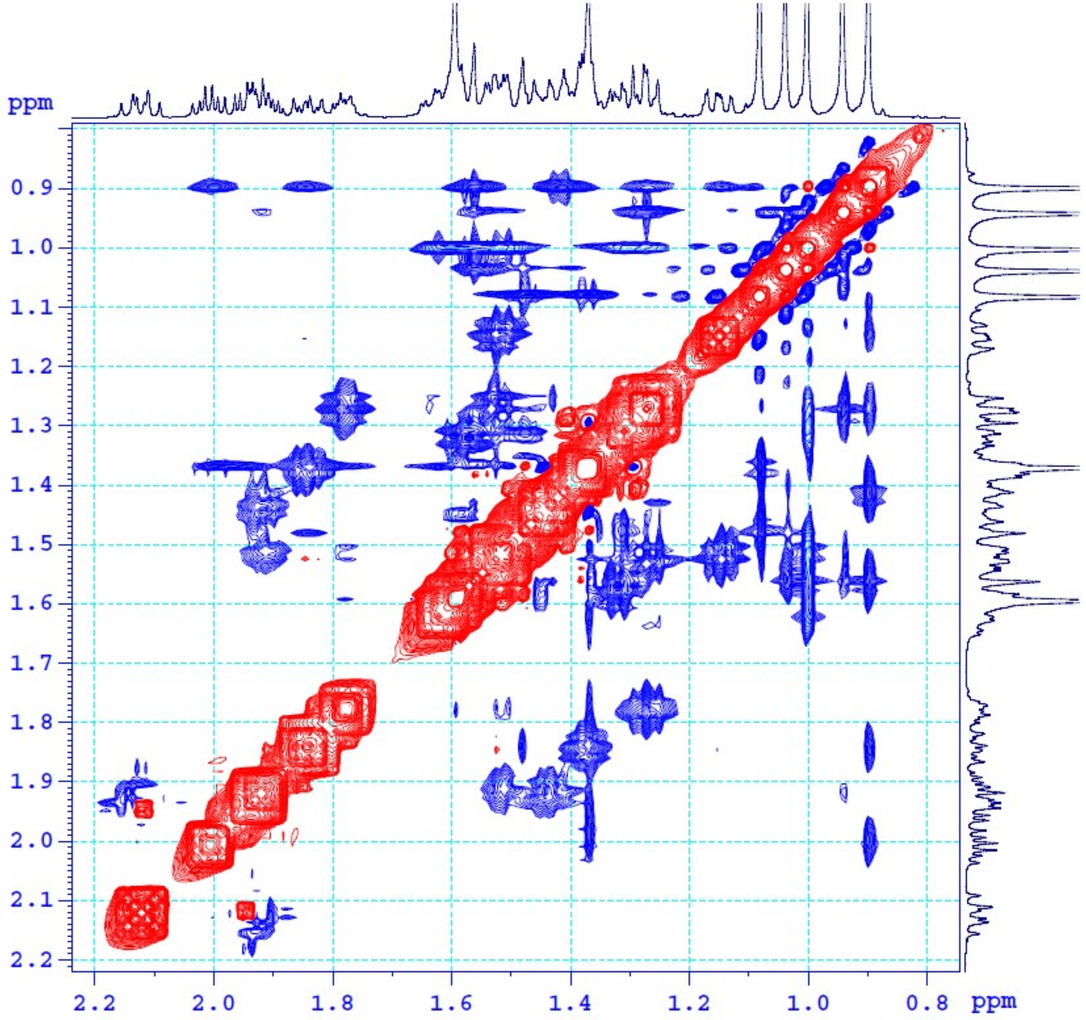


NOESY spectrum of compound **4** (extension)

## 1.16. Compound 5

Sample name: DipOH
Operator: Le Anh VHH
Method: +IDA TOF MS/MS
Date: 2021.04.23

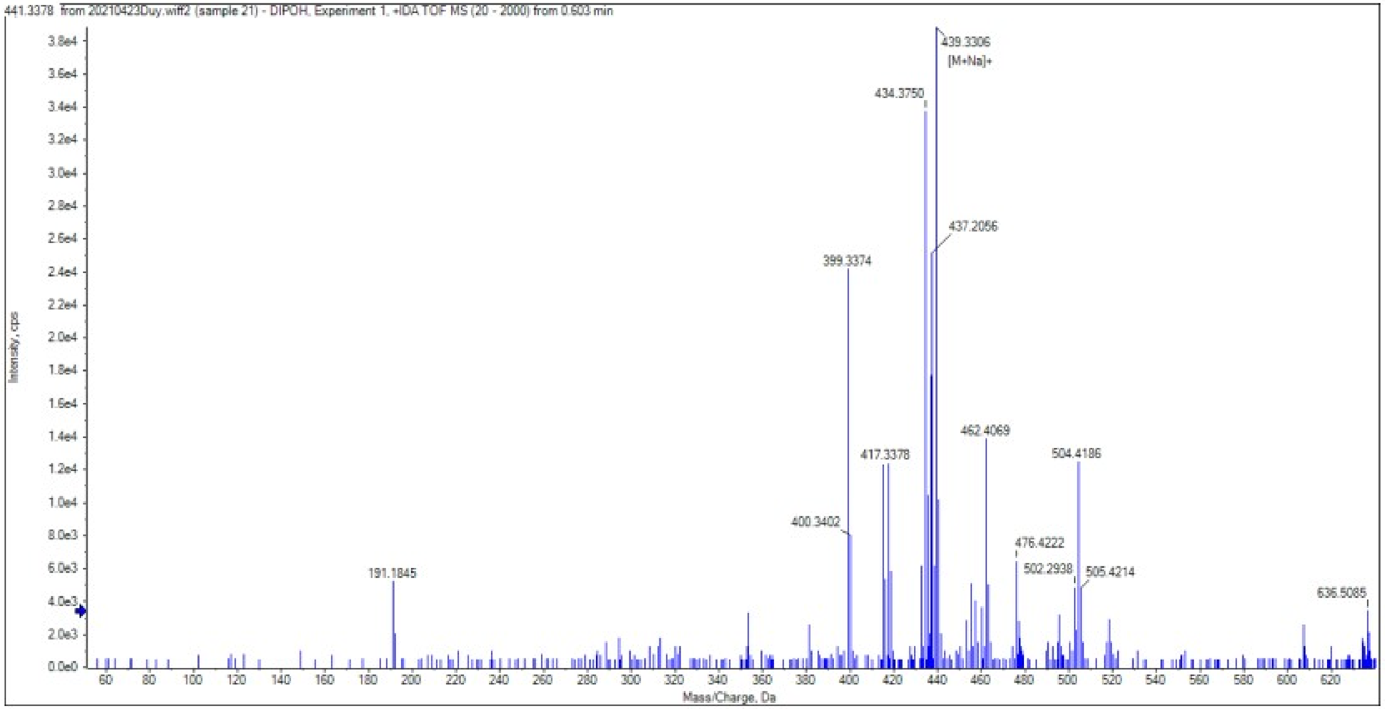


| Hit | Formula | m/z | RDB | ppm | MS Rank | MSMS ppm | MSMS Rank | Found |
|---|---|---|---|---|---|---|---|---|
| 1 | C27H44O3 | 417.33632 | 6.0 | 1.9 | 1 | | | NA/NA |

(+)-HR-ESI-MS spectrum of compound **5**

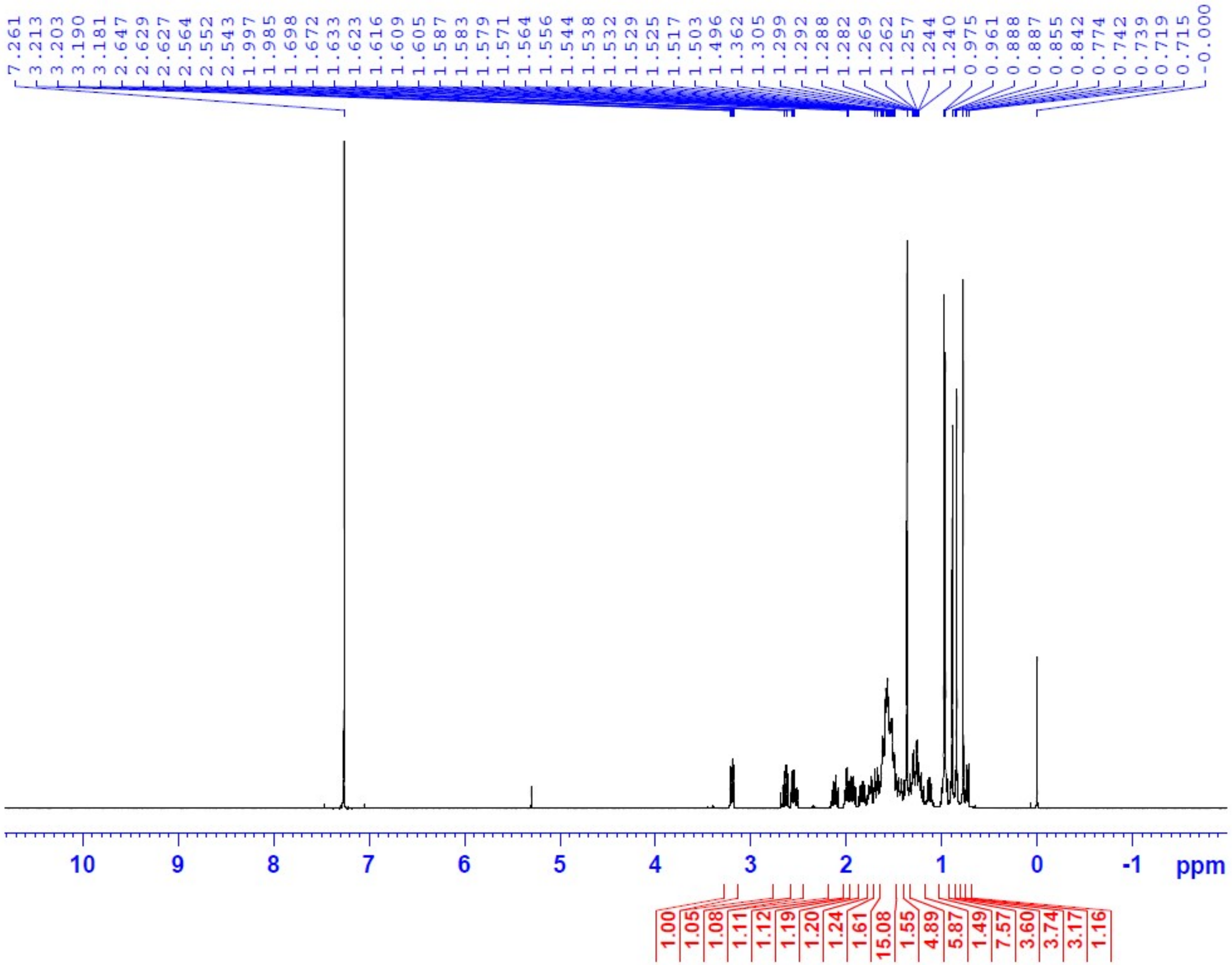

$^{1}$H-NMR spectrum of compound **5**

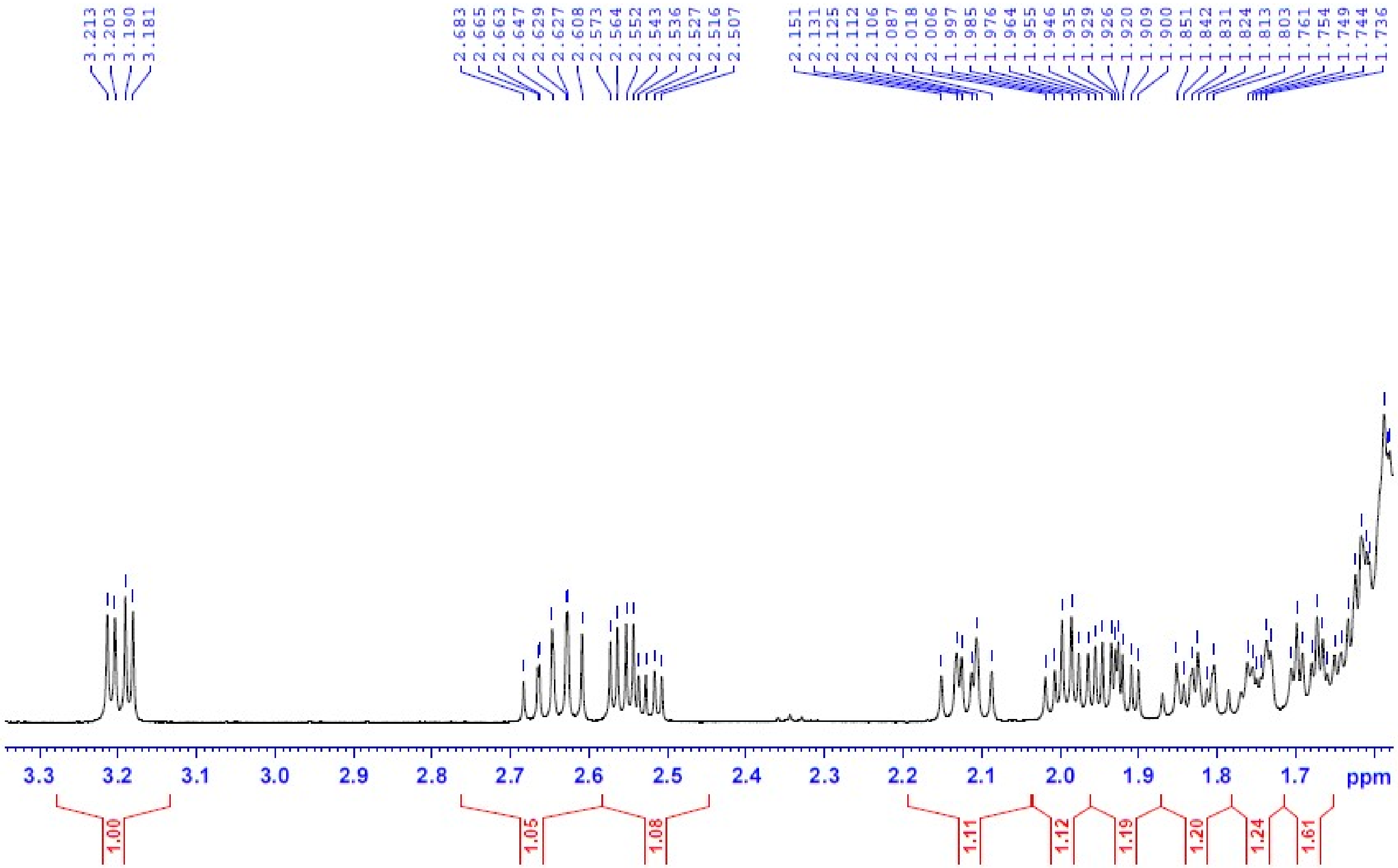
3.213
3.203
3.190
3.181
2.683
2.665
2.663
2.647
2.629
2.627
2.608
2.573
2.564
2.552
2.543
2.536
2.527
2.516
2.507
2.151
2.131
2.125
2.112
2.106
2.087
2.018
2.006
1.997
1.985
1.976
1.964
1.955
1.946
1.935
1.929
1.926
1.920
1.909
1.900
1.851
1.842
1.831
1.824
1.813
1.803
1.761
1.754
1.749
1.744
1.736
3.3
3.2
3.1
3.0
2.9
2.8
2.7
2.6
2.5
2.4
2.3
2.2
2.1
2.0
1.9
1.8
1.7
ppm
1.00
1.05
1.08
1.11
1.12
1.19
1.20
1.24
1.61

$^{1}$H-NMR spectrum of compound **5** (extension)

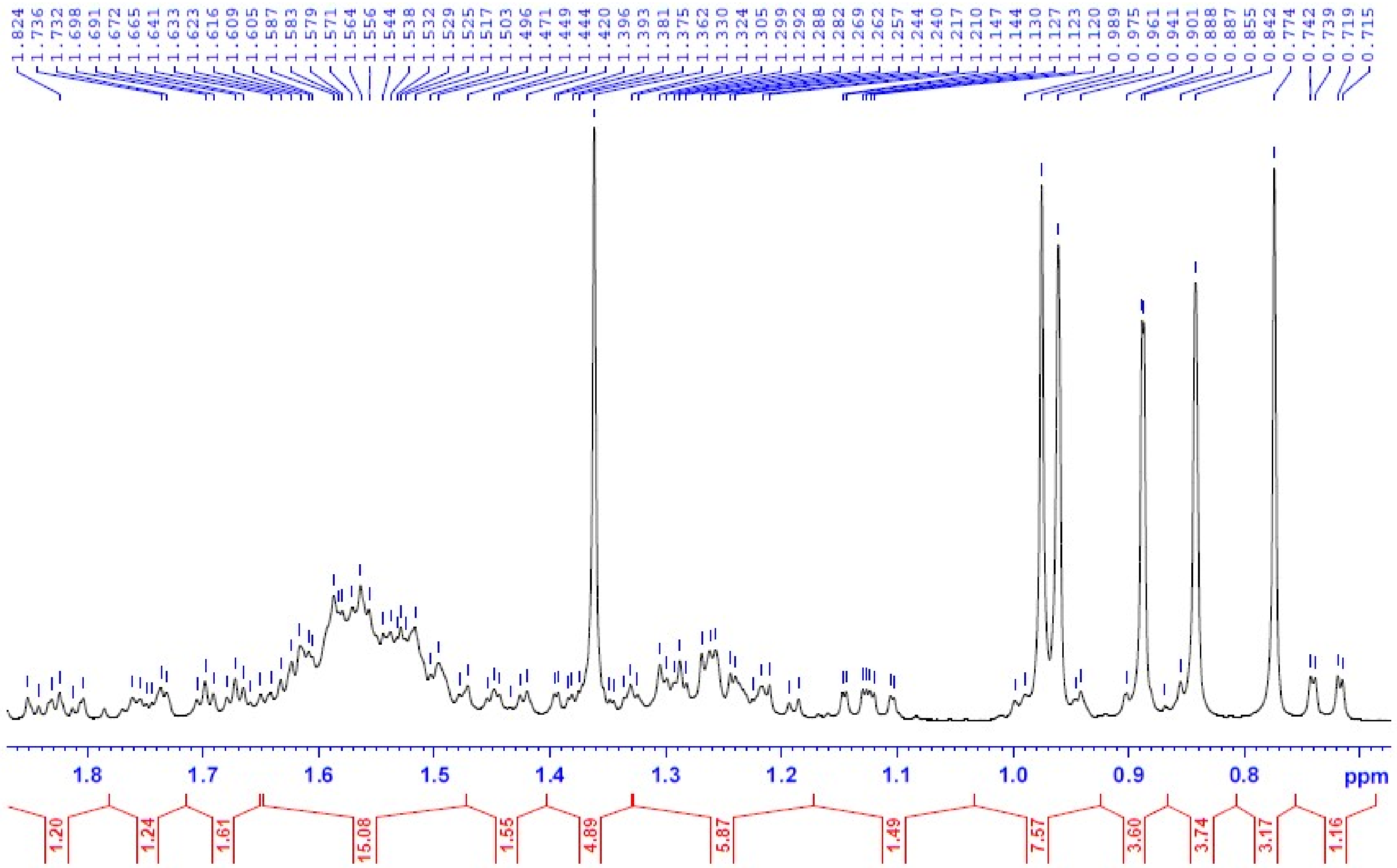
1.824
1.736
1.732
1.698
1.691
1.672
1.665
1.641
1.633
1.623
1.616
1.609
1.605
1.587
1.583
1.579
1.571
1.564
1.556
1.544
1.538
1.532
1.529
1.525
1.517
1.503
1.496
1.471
1.449
1.444
1.420
1.396
1.393
1.381
1.375
1.362
1.330
1.324
1.305
1.299
1.292
1.288
1.282
1.269
1.262
1.257
1.244
1.240
1.217
1.210
1.147
1.144
1.130
1.127
1.123
1.120
0.989
0.975
0.961
0.941
0.901
0.888
0.887
0.855
0.842
0.774
0.742
0.739
0.719
0.715
1.8
1.7
1.6
1.5
1.4
1.3
1.2
1.1
1.0
0.9
0.8
ppm
1.20
1.24
1.61
15.08
1.55
4.89
5.87
1.49
7.57
3.60
3.74
3.17
1.16

$^{1}H$-NMR spectrum of compound **5** (extension)

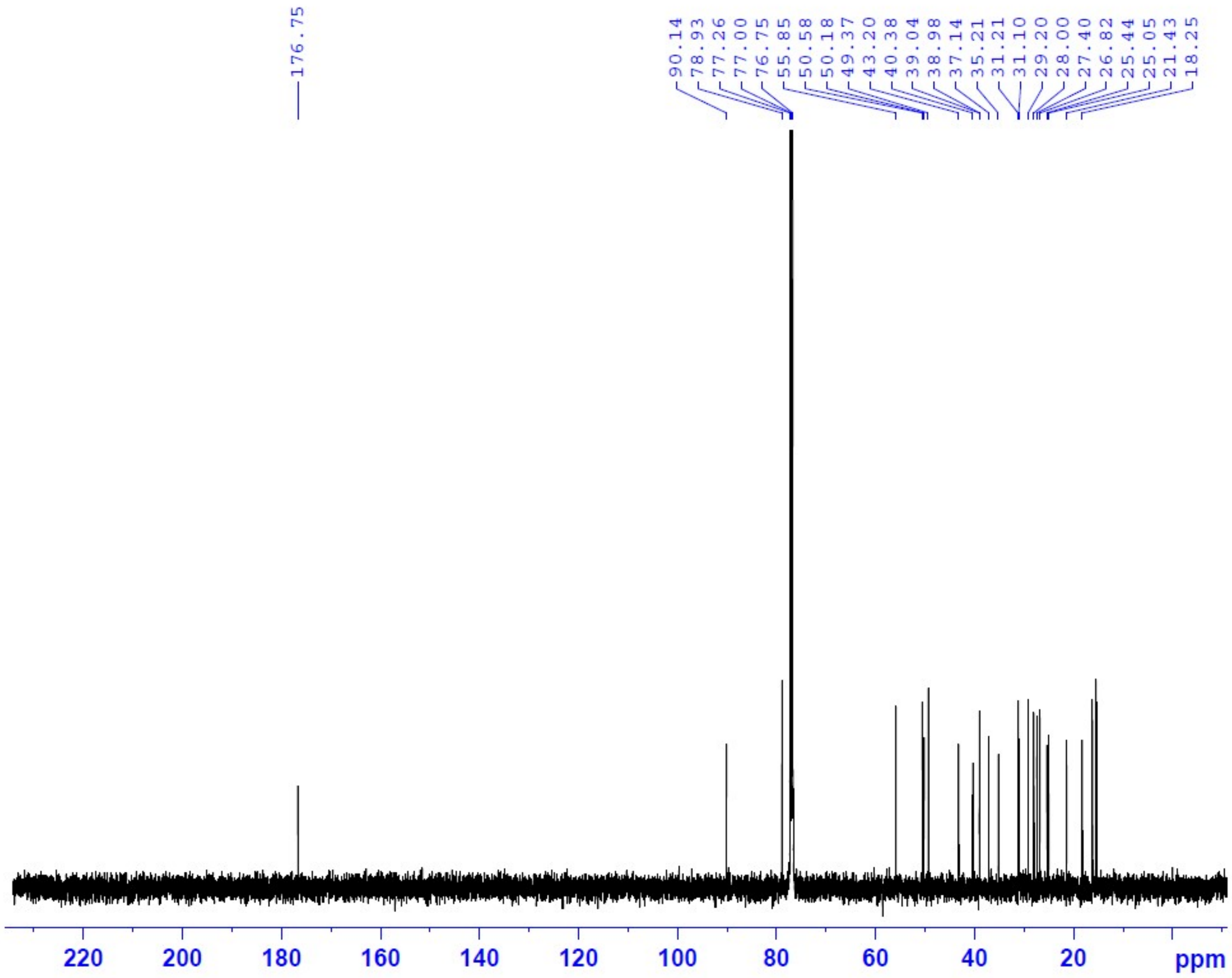


$^{13}C$-NMR spectrum of compound **5**

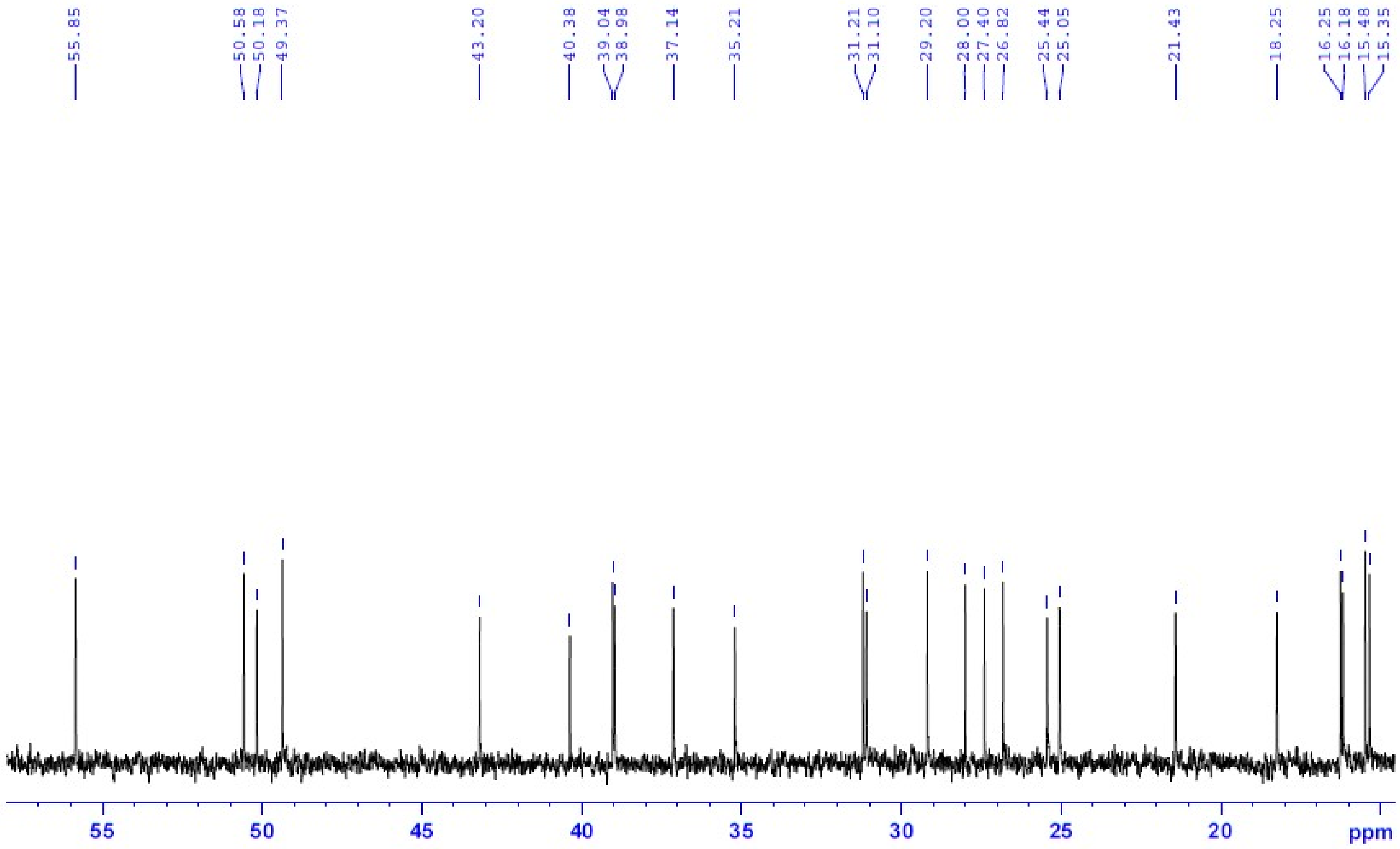
55.85
50.58
50.18
49.37
43.20
40.38
39.04
38.98
37.14
35.21
31.21
31.10
29.20
28.00
27.40
26.82
25.44
25.05
21.43
18.25
16.25
16.18
15.48
15.35
55
50
45
40
35
30
25
20
ppm

$^{13}$C-NMR spectrum of compound **5** (extension)

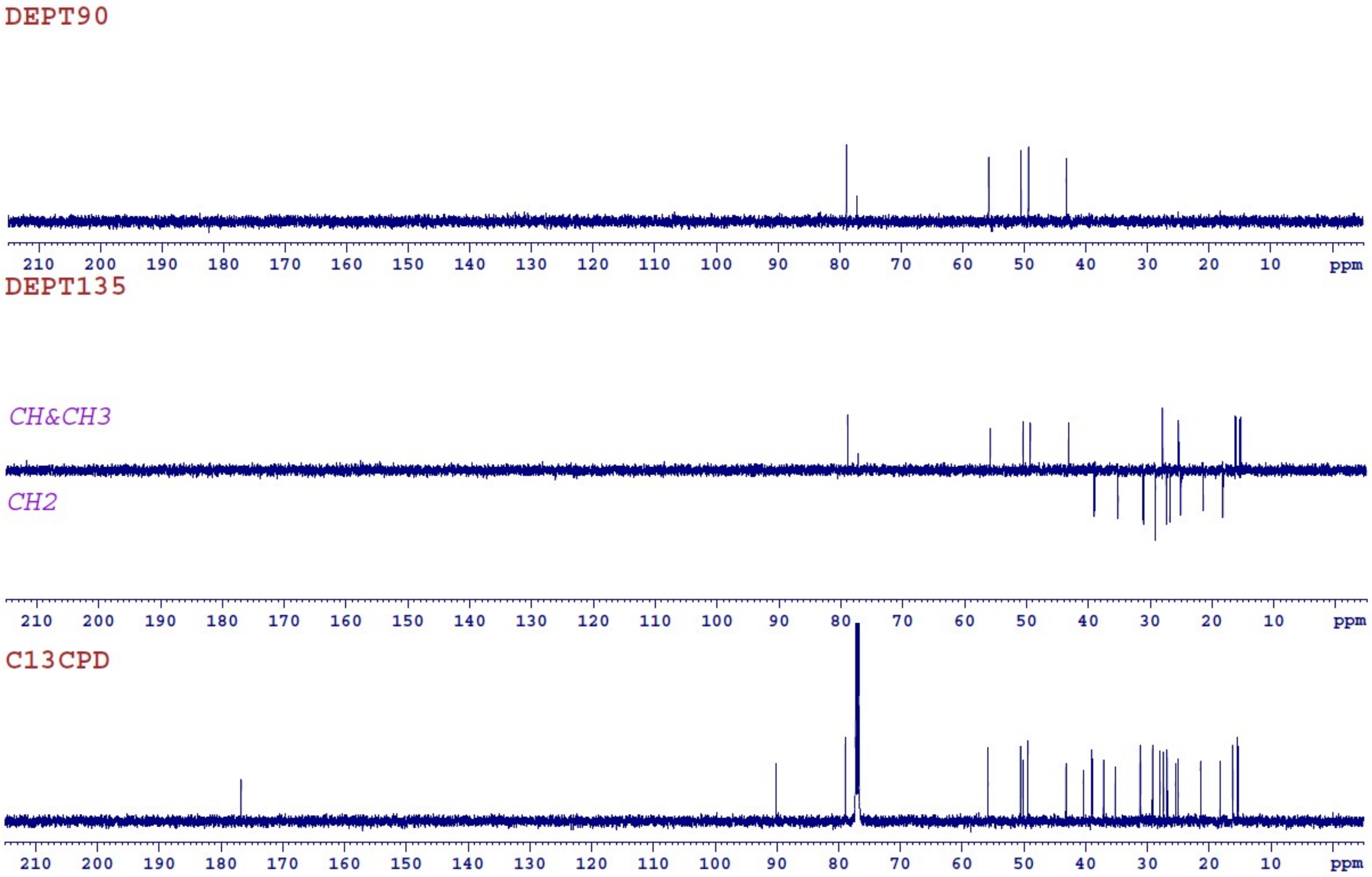


DEPT spectrum of compound **5**

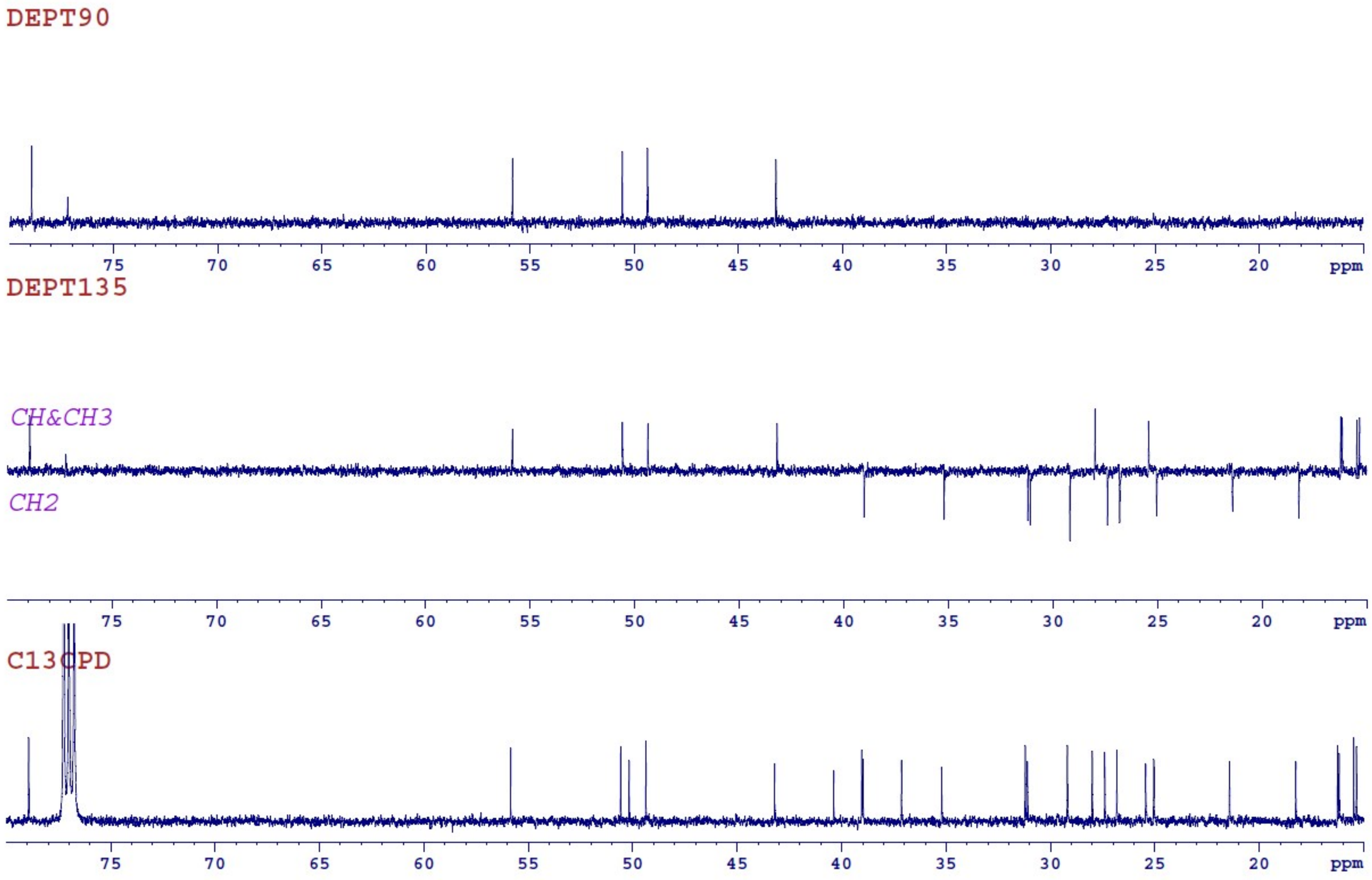


DEPT spectrum of compound **5** (extension)

### 1.17. Compound 6a

**Sample name: DIPAcE**
**Operator: Le Anh VHH**
**Method: +IDA TOF MS/MS**
**Date: 2021.04.23**

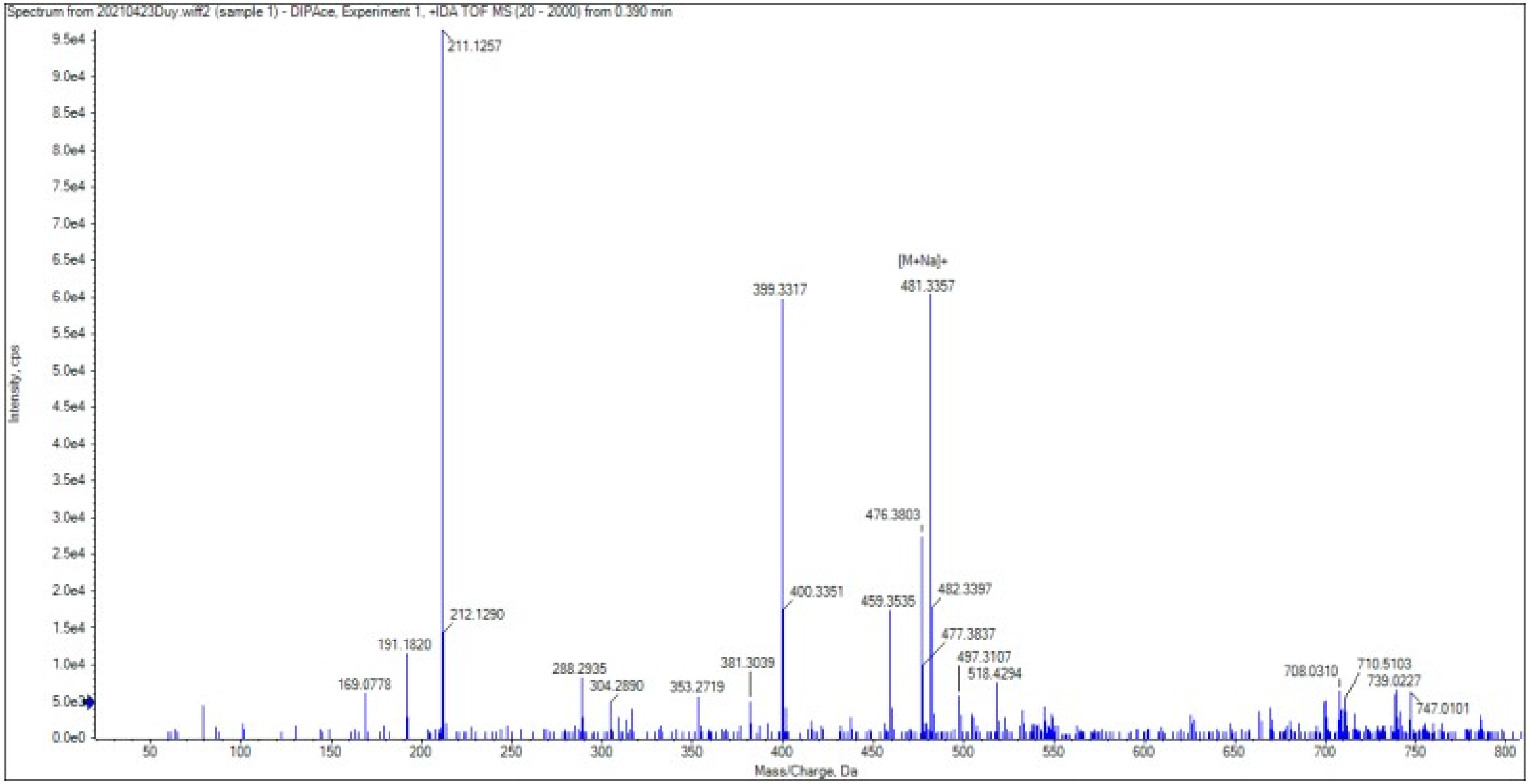


| Hit | Formula | m/z | RDB | ppm | MS Rank | MSMS ppm | MSMS Rank | Found |
|---|---|---|---|---|---|---|---|---|
| 1 | C29H46O4 | 481.32883 | 7.0 | 4.3 | 1 | | | NA/NA |

(+)-HR-ESI-MS spectrum of compound **6a**

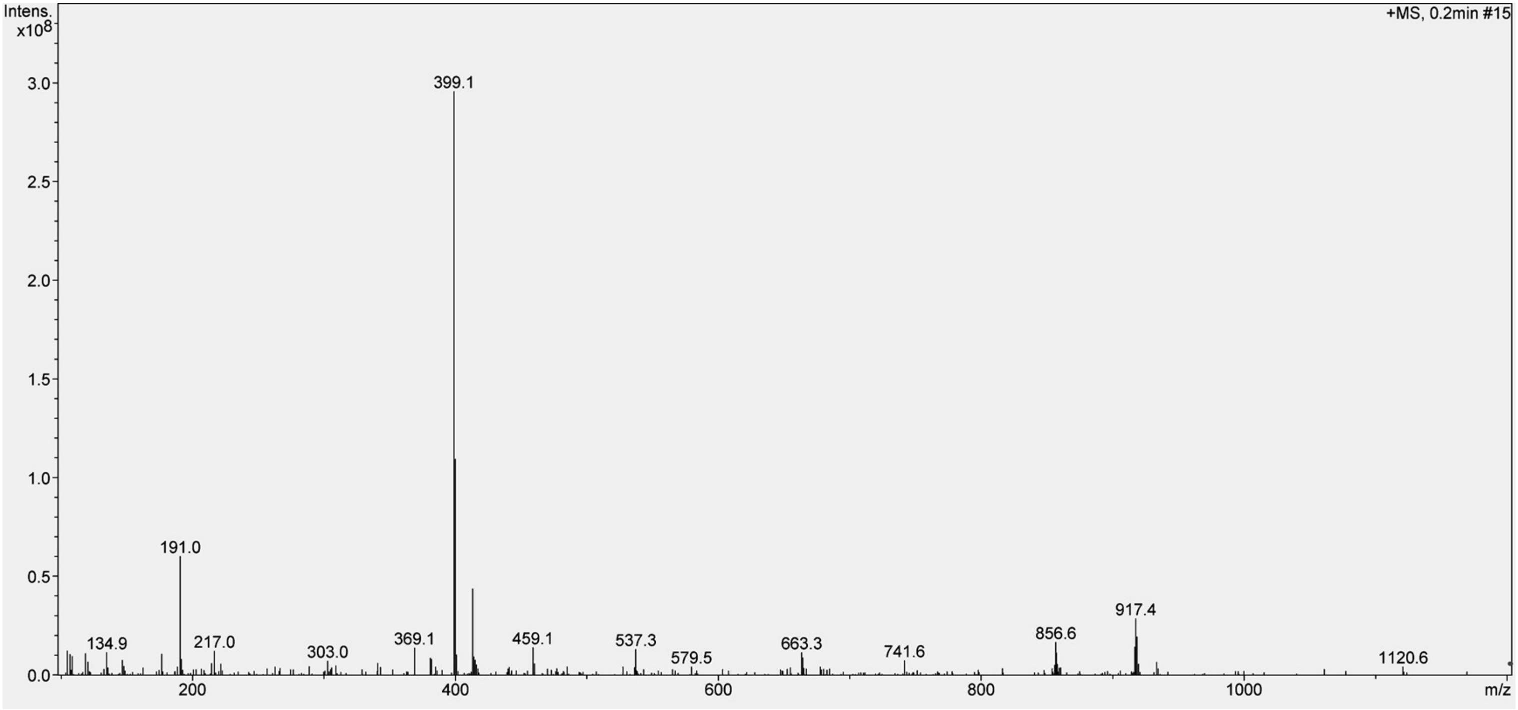


(+)-ESI–MS spectrum of compound **6a**

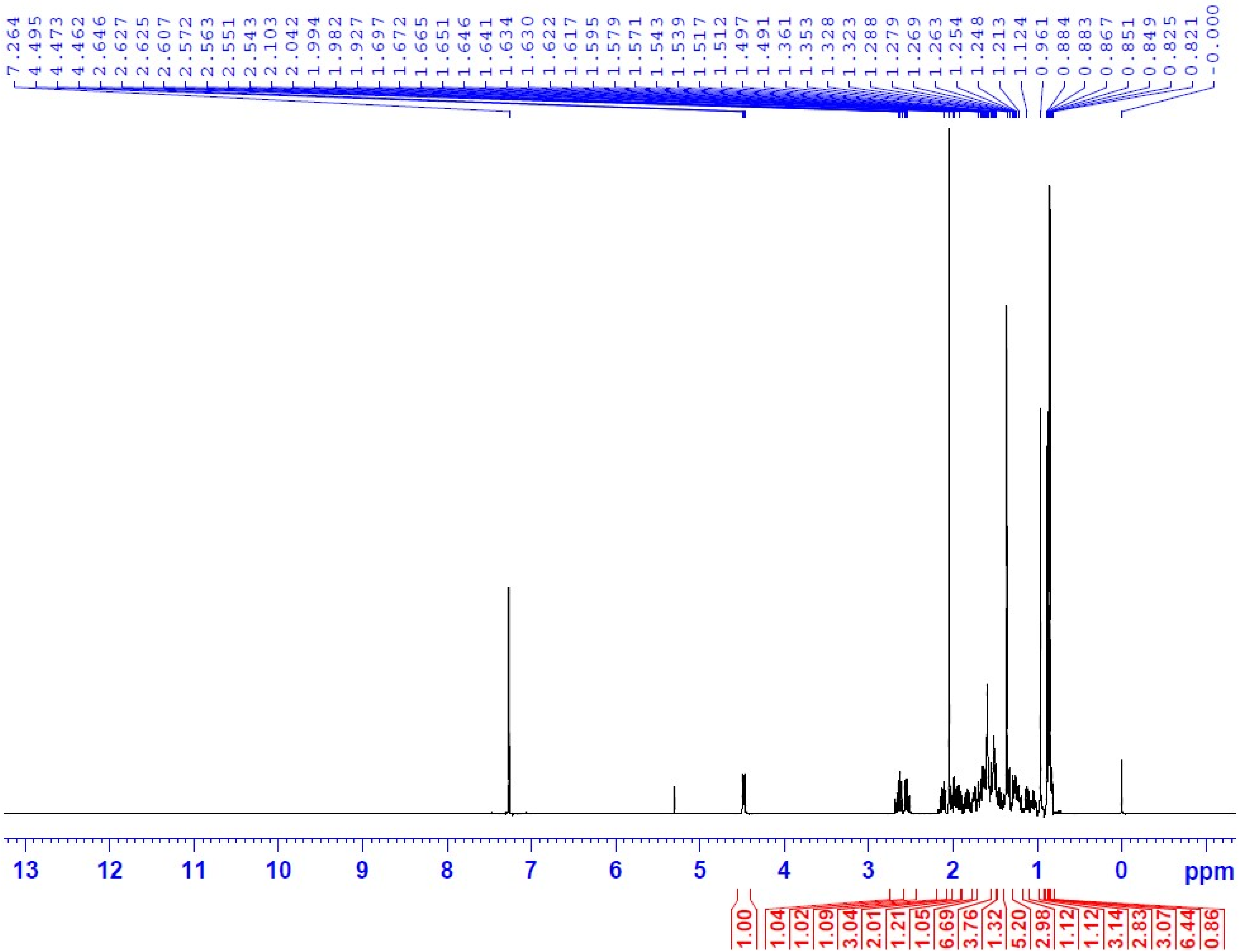

$^{1}$H-NMR spectrum of compound **6a**

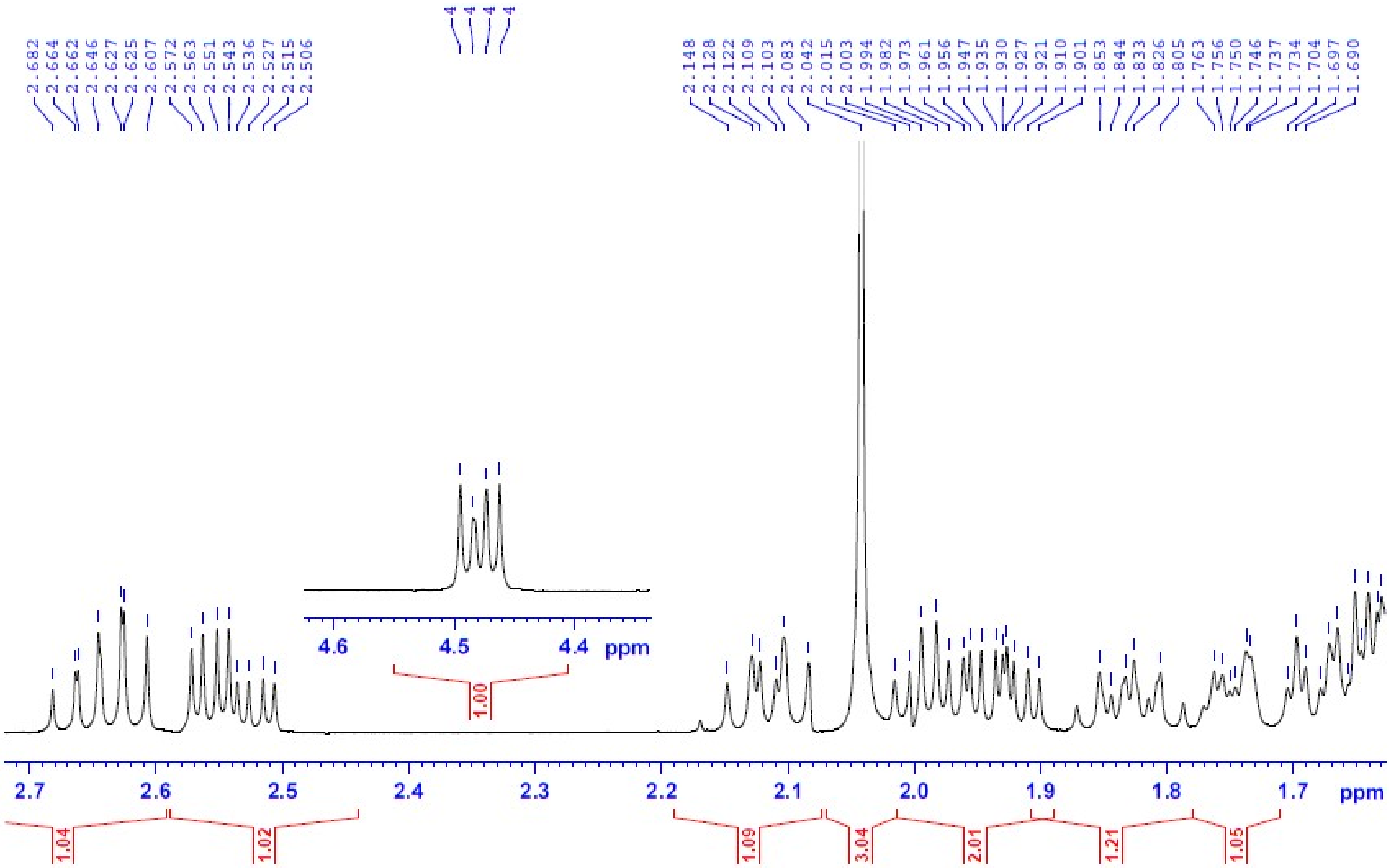

$^{1}$H-NMR spectrum of compound **6a** (extension)

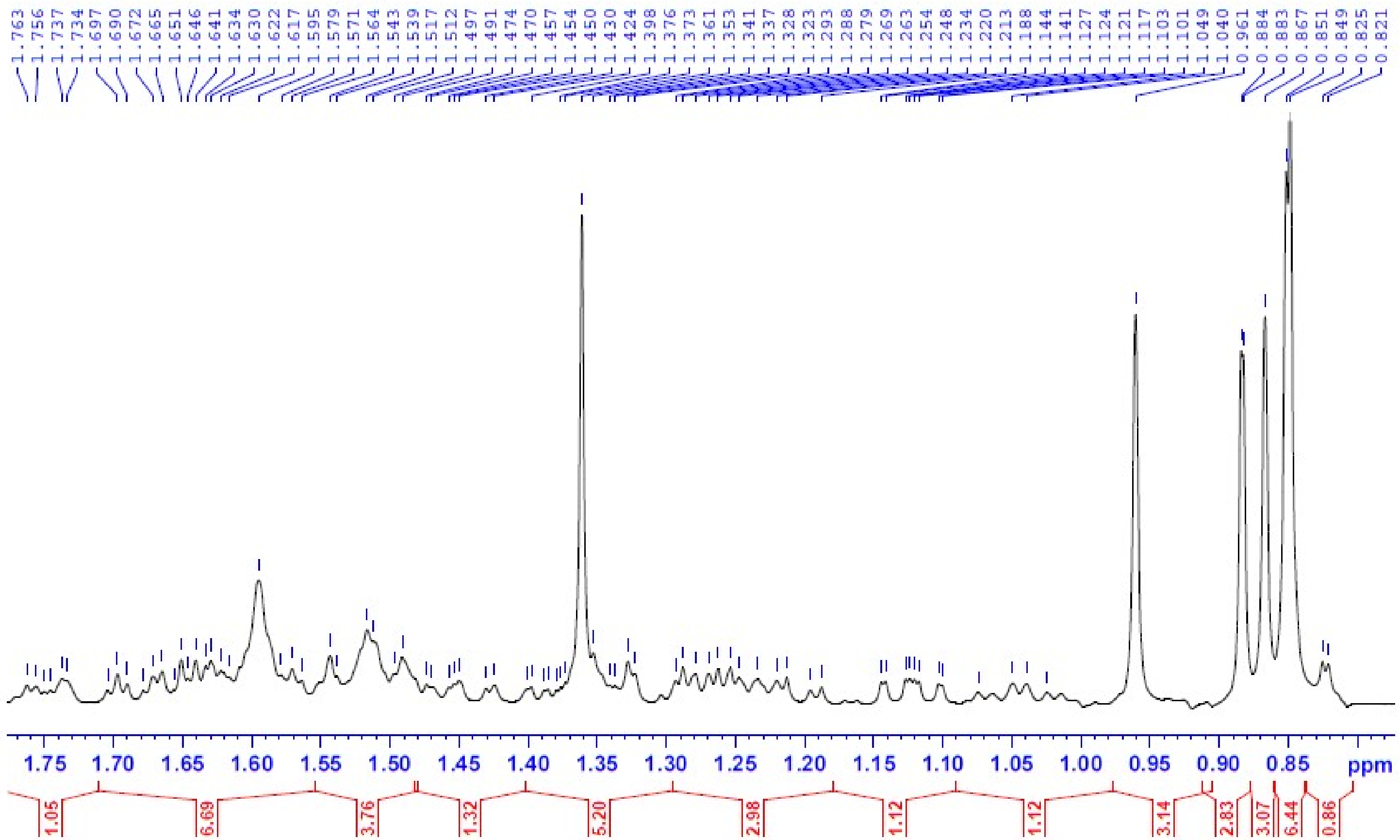

$^{1}$H-NMR spectrum of compound **6a** (extension)

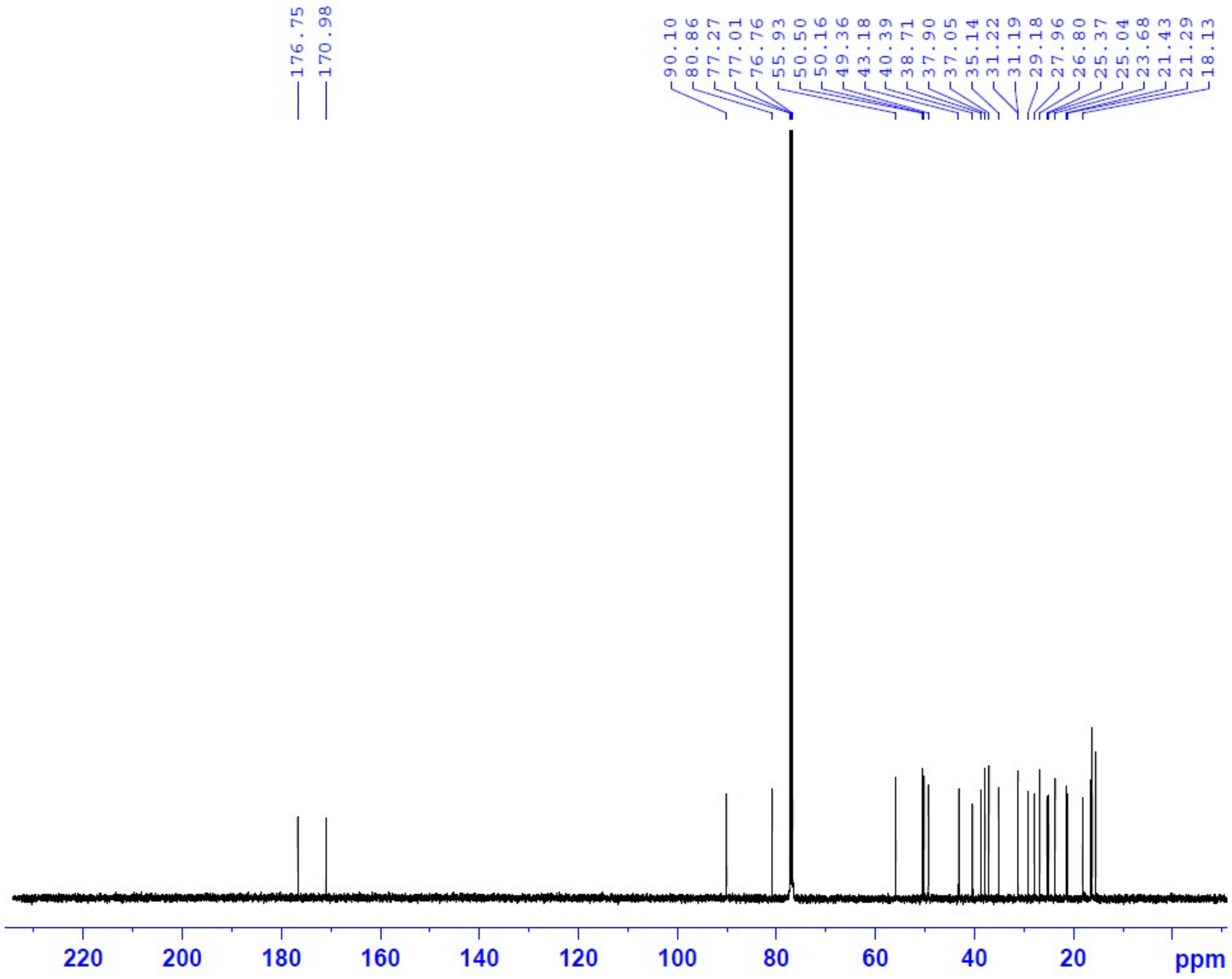


$^{13}$C-NMR spectrum of compound **6a**

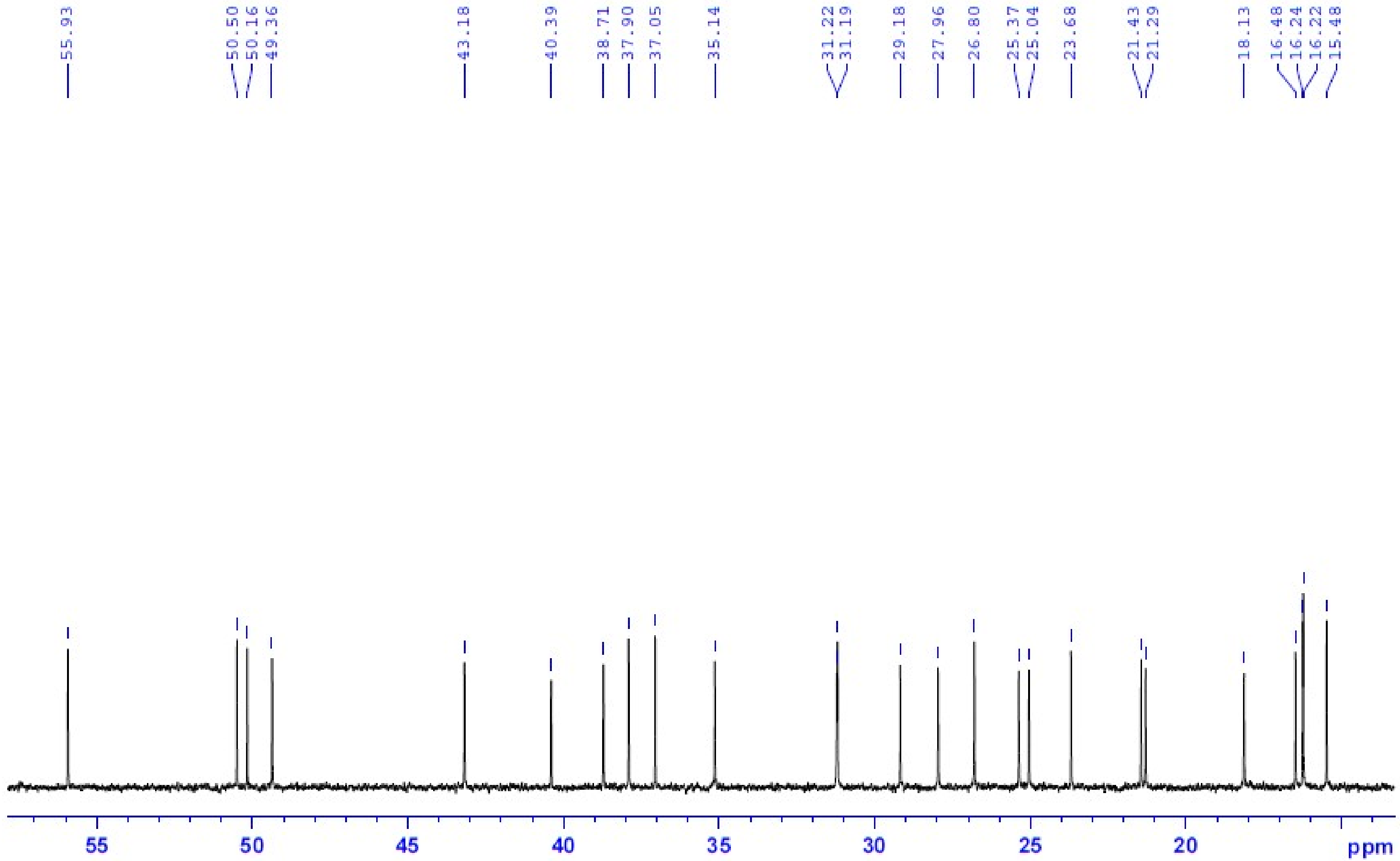
55.93
50.50
50.16
49.36
43.18
40.39
38.71
37.90
37.05
35.14
31.22
31.19
29.18
27.96
26.80
25.37
25.04
23.68
21.43
21.29
18.13
16.48
16.24
16.22
15.48
55
50
45
40
35
30
25
20
ppm

$^{13}$C-NMR spectrum of compound **6a** (extension)

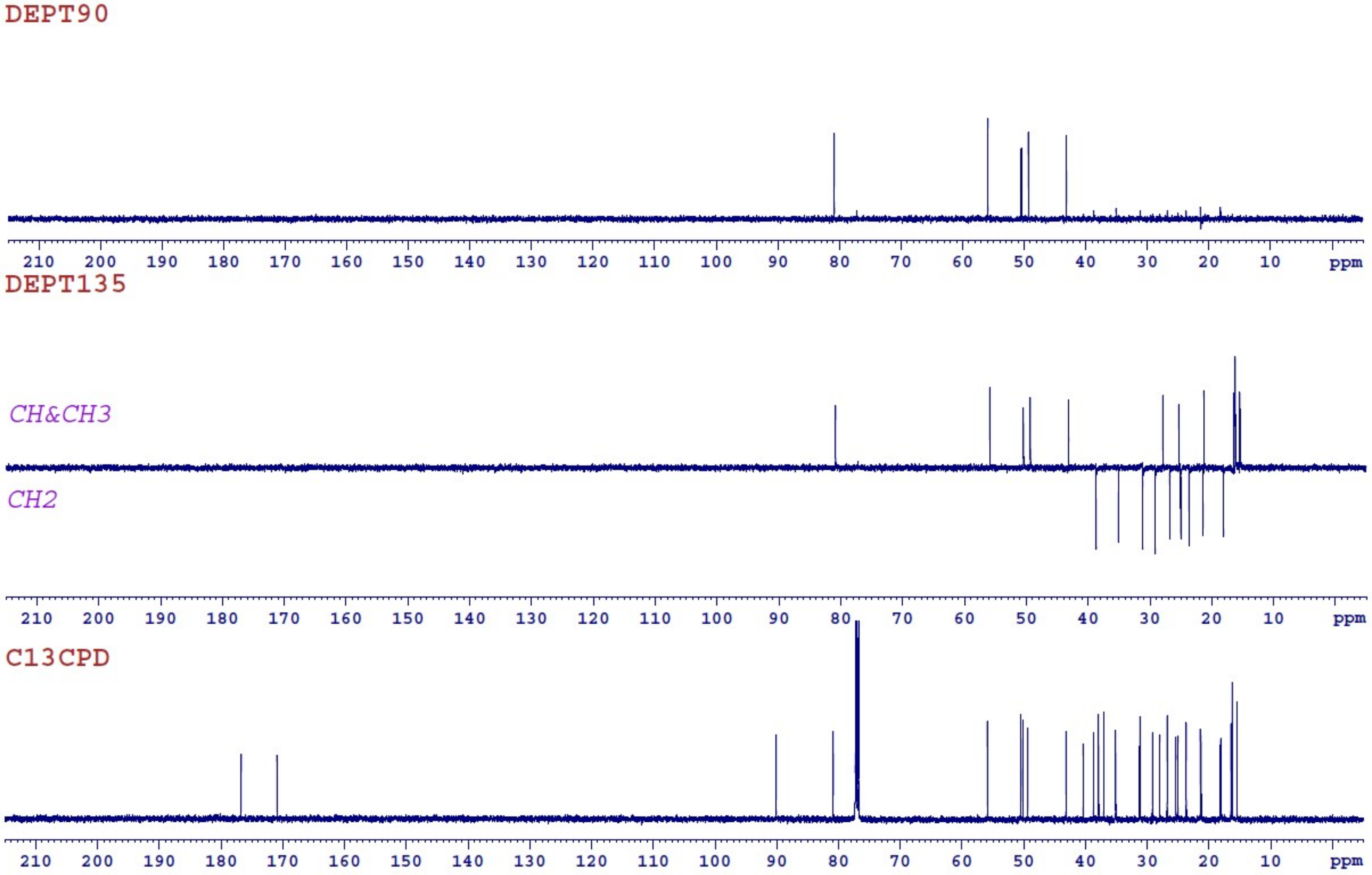


DEPT spectrum of compound **6a**

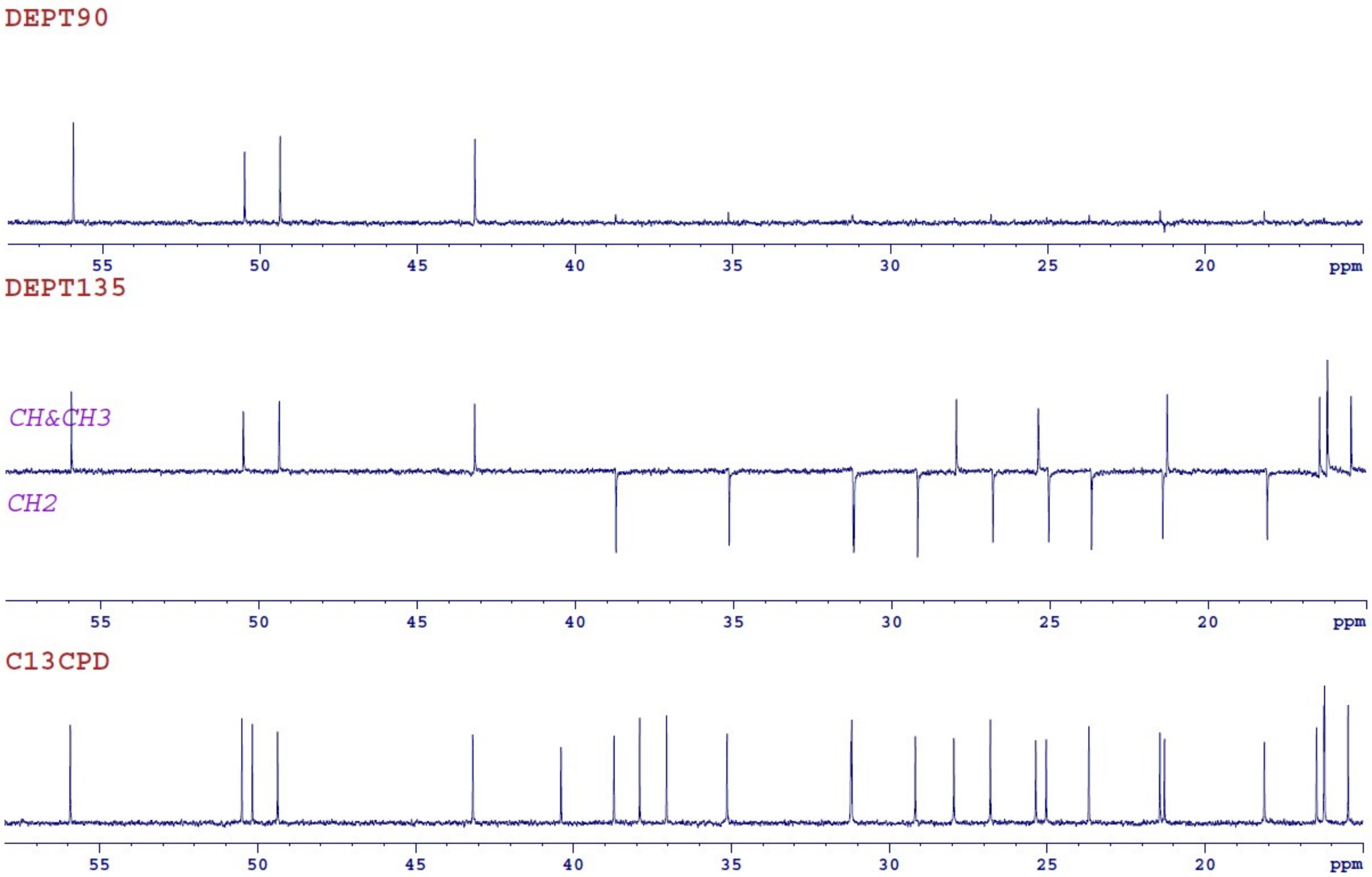


DEPT spectrum of compound **6a** (extension)

### 1.18. Compound 6b

***Sample name: DipVali***
***Operator: Le Anh VHH***
***Method: +IDA TOF MS/MS***
***Date: 2021.04.23***

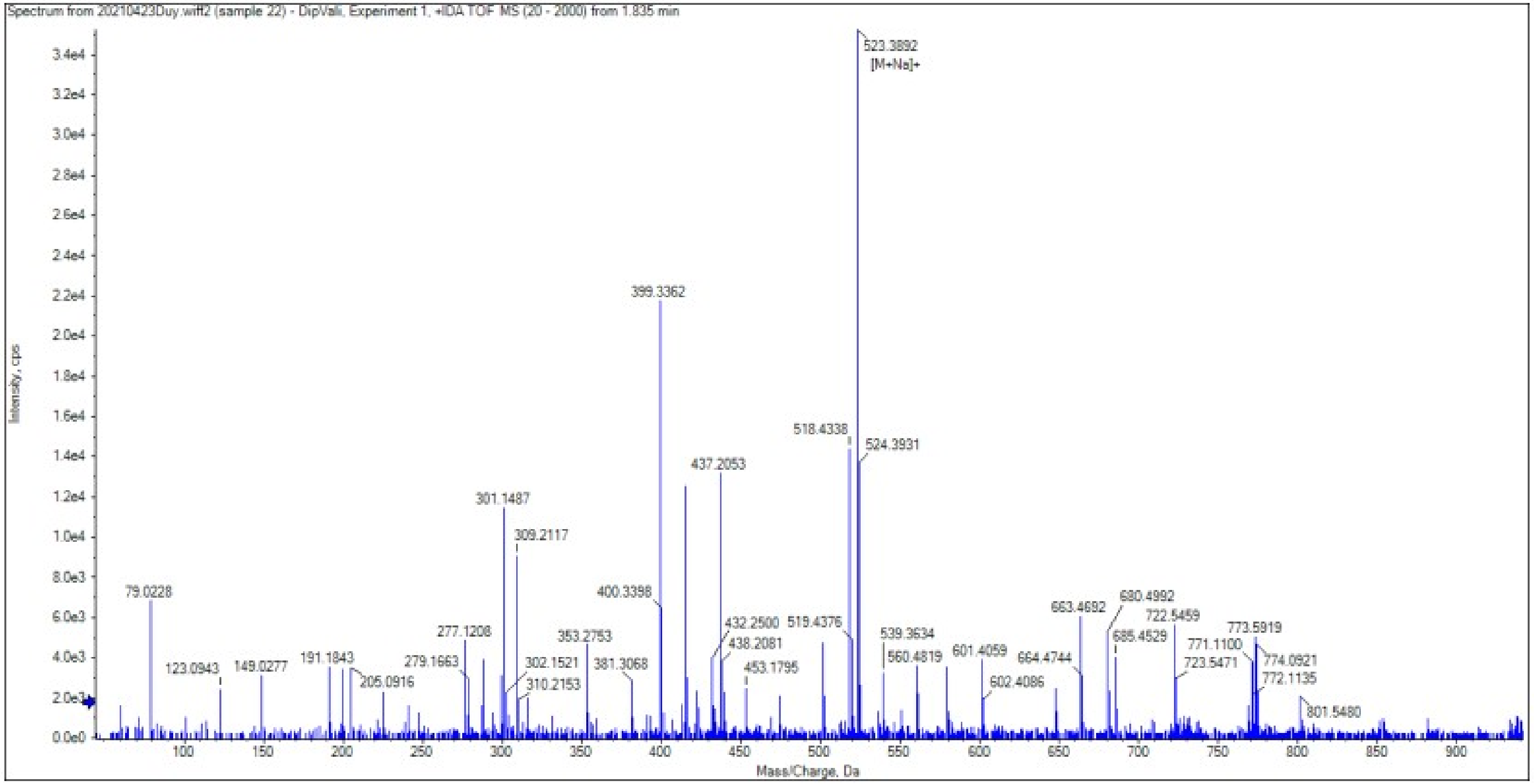


| Hit | Formula | m/z | RDB | ppm | MS Rank | MSMS ppm | MSMS Rank | Found |
|---|---|---|---|---|---|---|---|---|
| 1 | C32H52O4 | 523.3858 | 7.0 | 3.6 | 1 | | | NA/NA |

(+)-HR-ESI-MS spectrum of compound **6b**

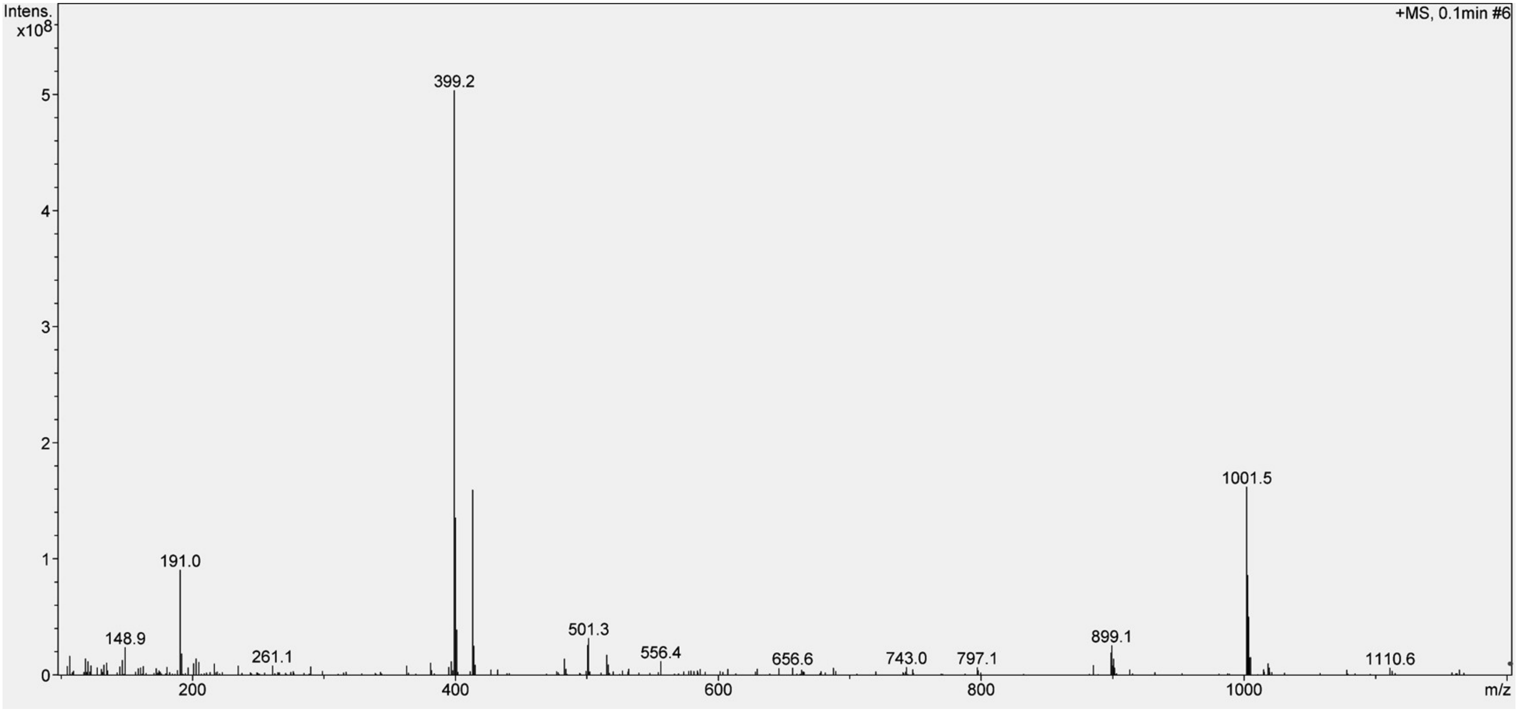


(+)-ESI–MS spectrum of compound **6b**

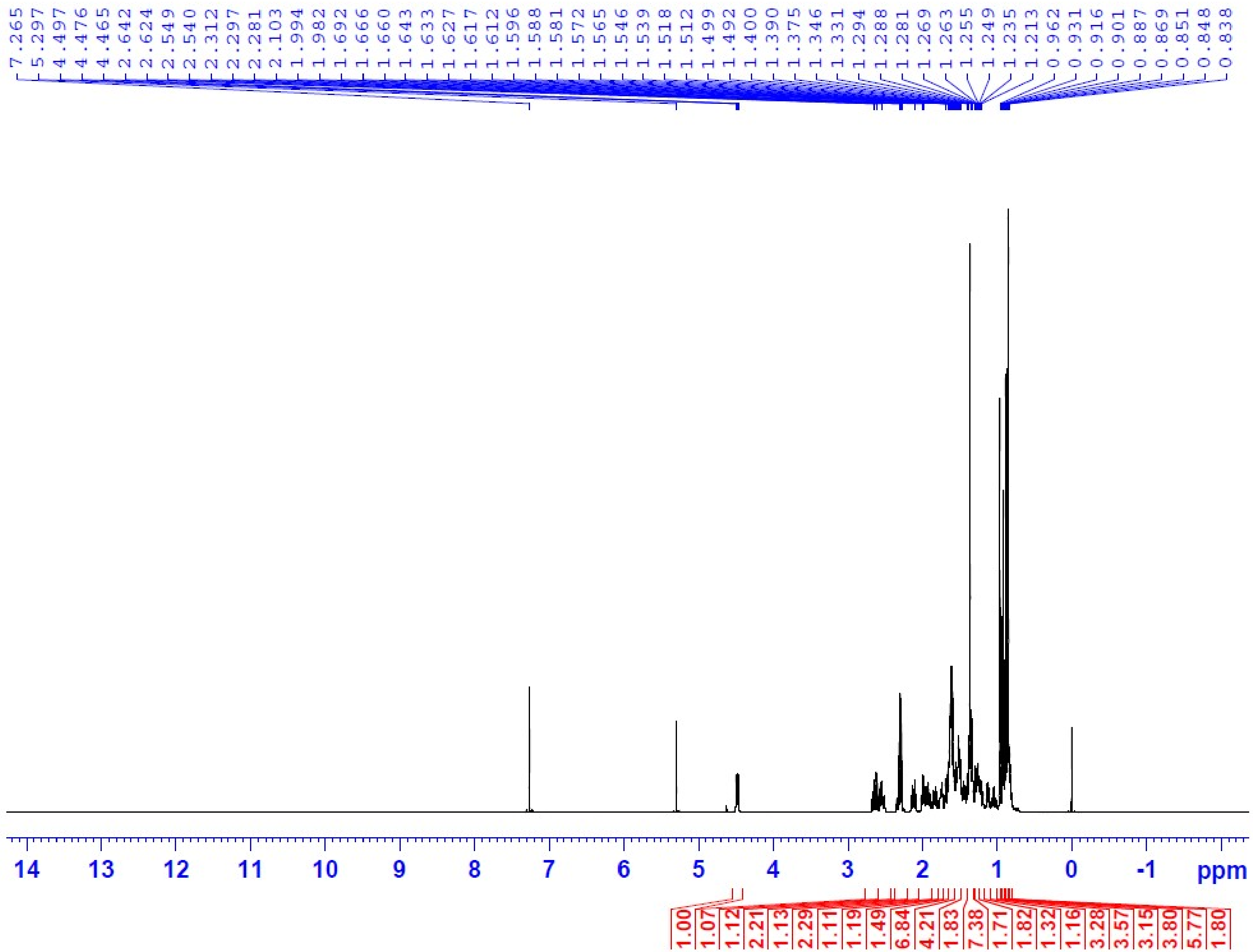

$^{1}$H-NMR spectrum of compund **6b**

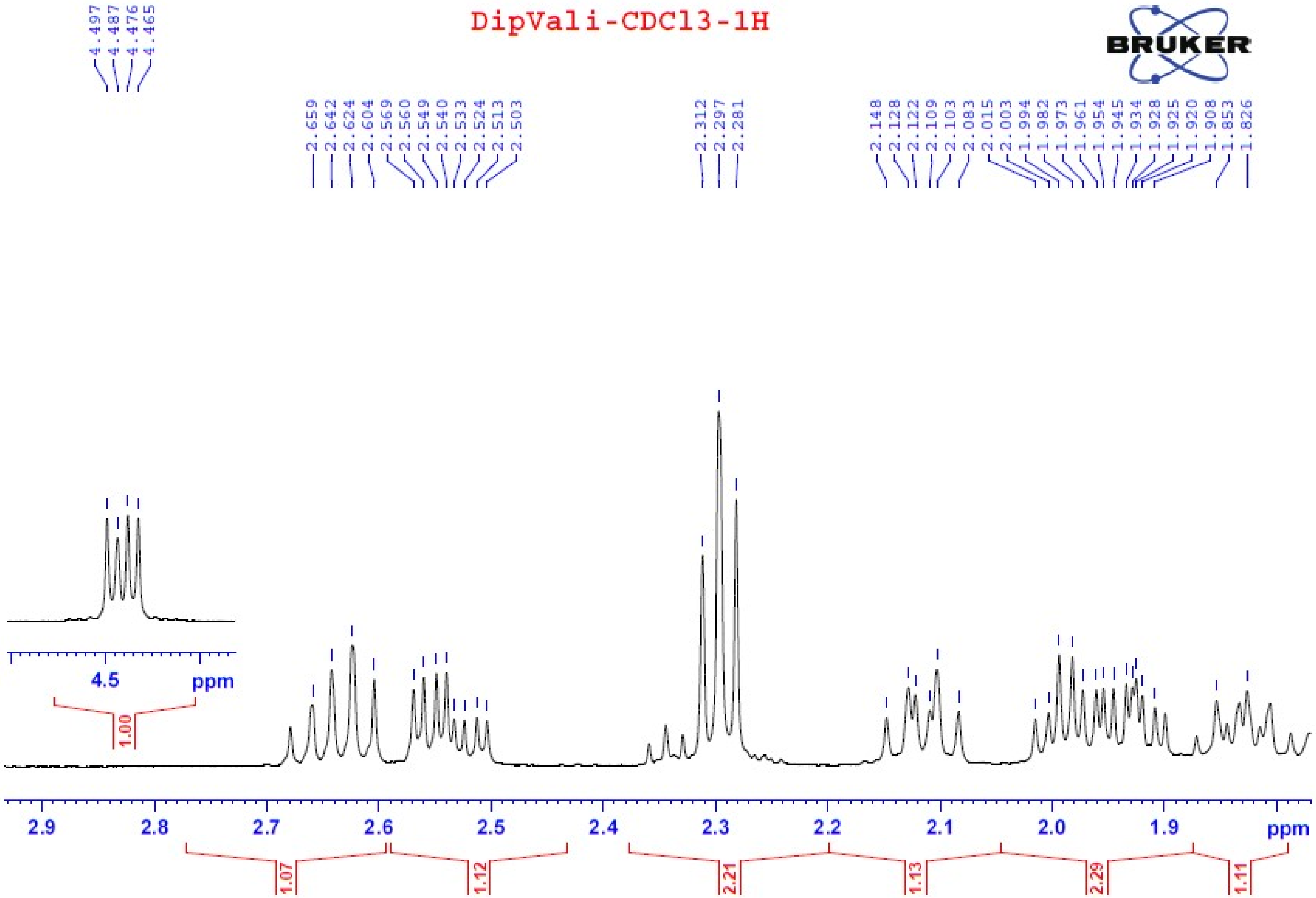
DipVali-CDCl3-1H
BRUKER
4.497
4.487
4.476
4.465
2.659
2.642
2.624
2.604
2.569
2.560
2.549
2.540
2.533
2.524
2.513
2.503
2.312
2.297
2.281
2.148
2.128
2.122
2.109
2.103
2.083
2.015
2.003
1.994
1.982
1.973
1.961
1.954
1.945
1.934
1.928
1.925
1.920
1.908
1.853
1.826
4.5
ppm
1.00
2.9
2.8
2.7
2.6
2.5
2.4
2.3
2.2
2.1
2.0
1.9
ppm
1.07
1.12
2.21
1.13
2.29
1.11

<sup></sup>

$^{1}$H-NMR spectrum of compound **6b** (extension)

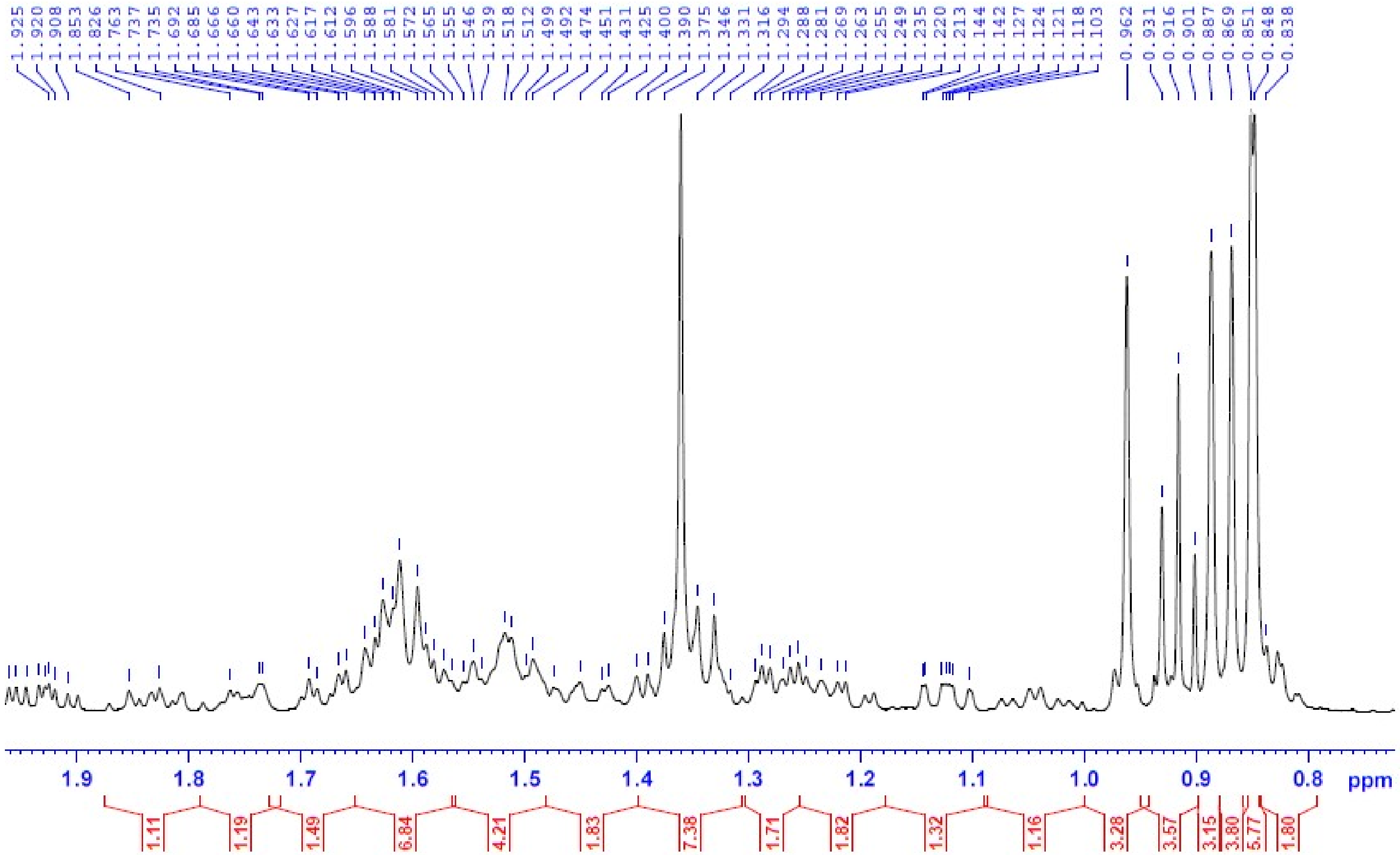
1.925
1.920
1.908
1.853
1.826
1.763
1.737
1.735
1.692
1.685
1.666
1.660
1.643
1.633
1.627
1.617
1.612
1.596
1.588
1.581
1.572
1.565
1.555
1.546
1.539
1.518
1.512
1.499
1.492
1.474
1.451
1.431
1.425
1.400
1.390
1.375
1.346
1.331
1.316
1.294
1.288
1.281
1.269
1.263
1.255
1.249
1.235
1.220
1.213
1.144
1.142
1.127
1.124
1.121
1.118
1.103
0.962
0.931
0.916
0.901
0.887
0.869
0.851
0.848
0.838
1.9
1.8
1.7
1.6
1.5
1.4
1.3
1.2
1.1
1.0
0.9
0.8
ppm
1.11
1.19
1.49
6.84
4.21
1.83
7.38
1.71
1.82
1.32
1.16
3.28
3.57
3.15
3.80
5.77
1.80

$^{1}$H-NMR spectrum of compound **6b** (extension)

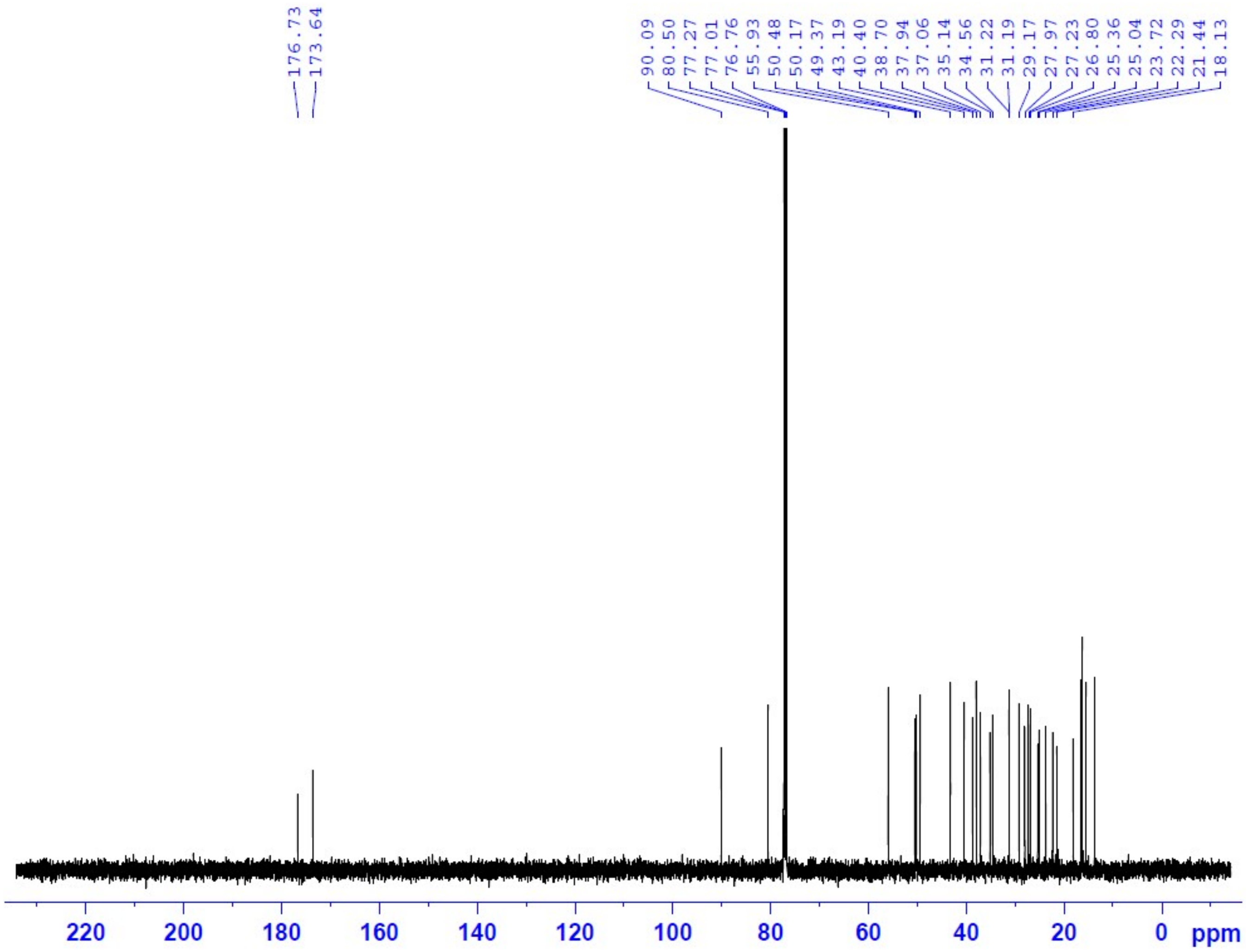


$^{13}$C-NMR spectrum of compound **6b**

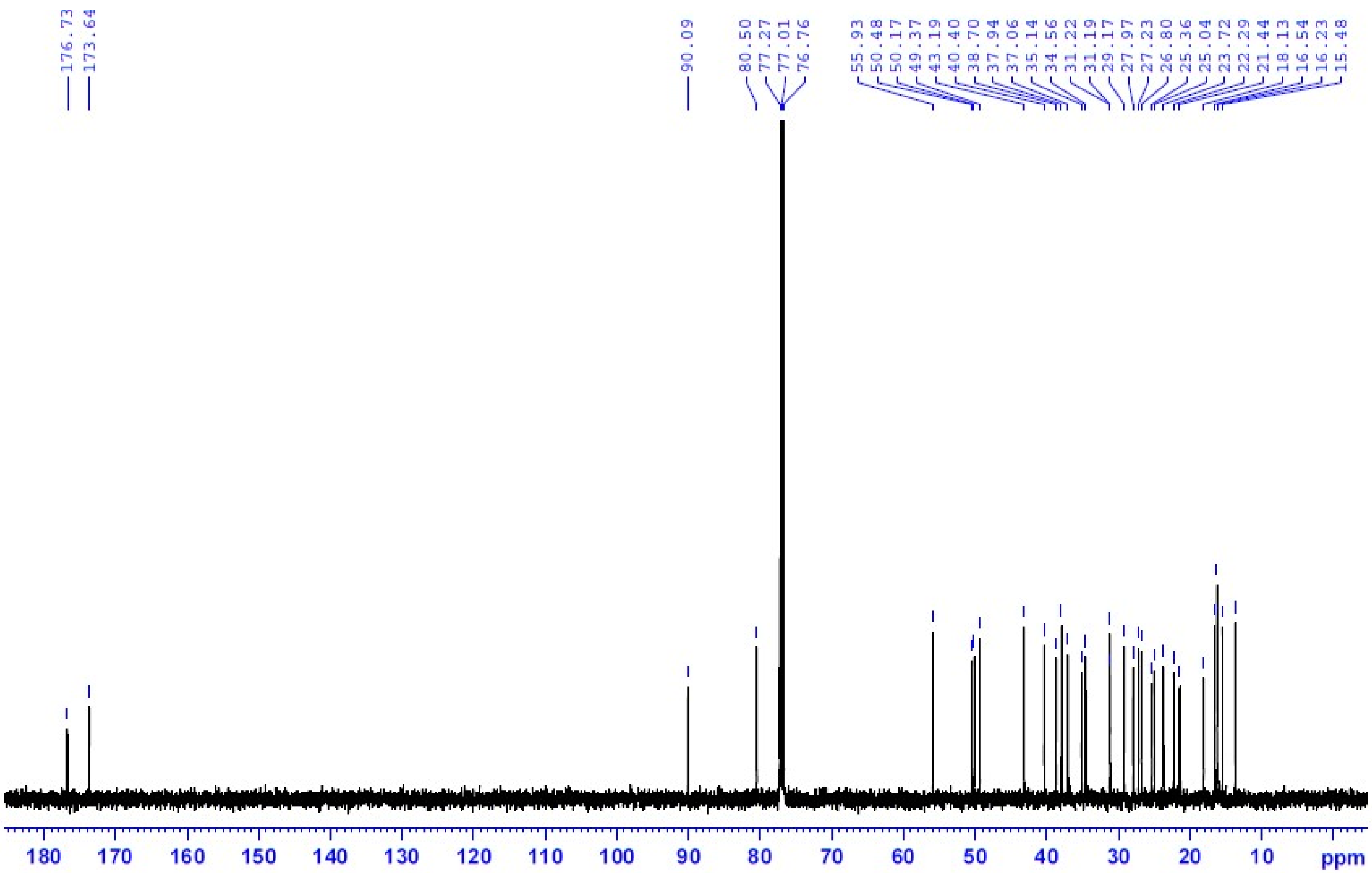
176.73
173.64
90.09
80.50
77.27
77.01
76.76
55.93
50.48
50.17
49.37
43.19
40.40
38.70
37.94
37.06
35.14
34.56
31.22
31.19
29.17
27.97
27.23
26.80
25.36
25.04
23.72
22.29
21.44
18.13
16.54
16.23
15.48
180 170 160 150 140 130 120 110 100 90 80 70 60 50 40 30 20 10 ppm

$^{13}$C-NMR spectrum of compound **6b** (extension)

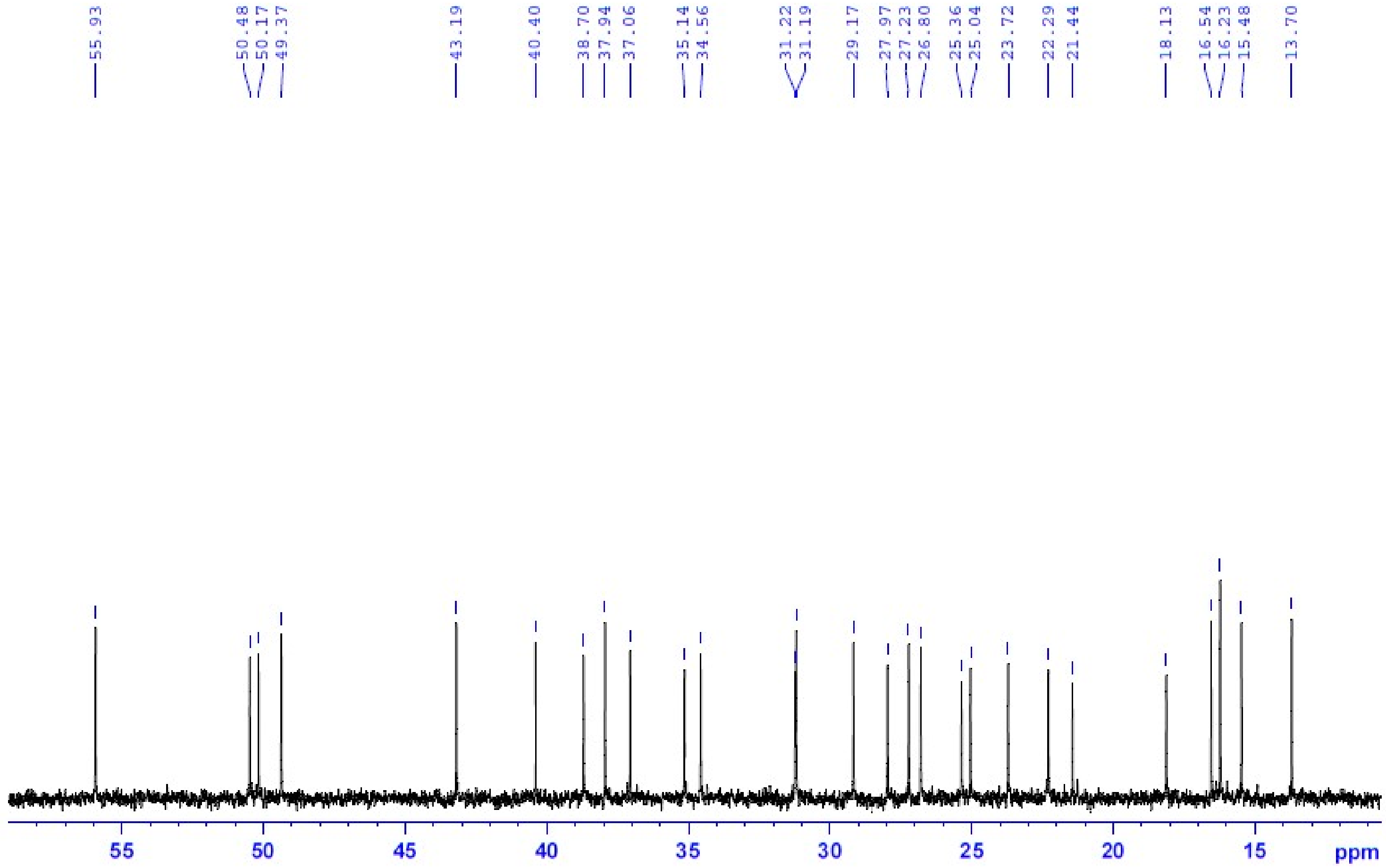

55.93
50.48
50.17
49.37
43.19
40.40
38.70
37.94
37.06
35.14
34.56
31.22
31.19
29.17
27.97
27.23
26.80
25.36
25.04
23.72
22.29
21.44
18.13
16.54
16.23
15.48
13.70
55
50
45
40
35
30
25
20
15
ppm

$^{13}$C-NMR spectrum of compound **6b** (extension)

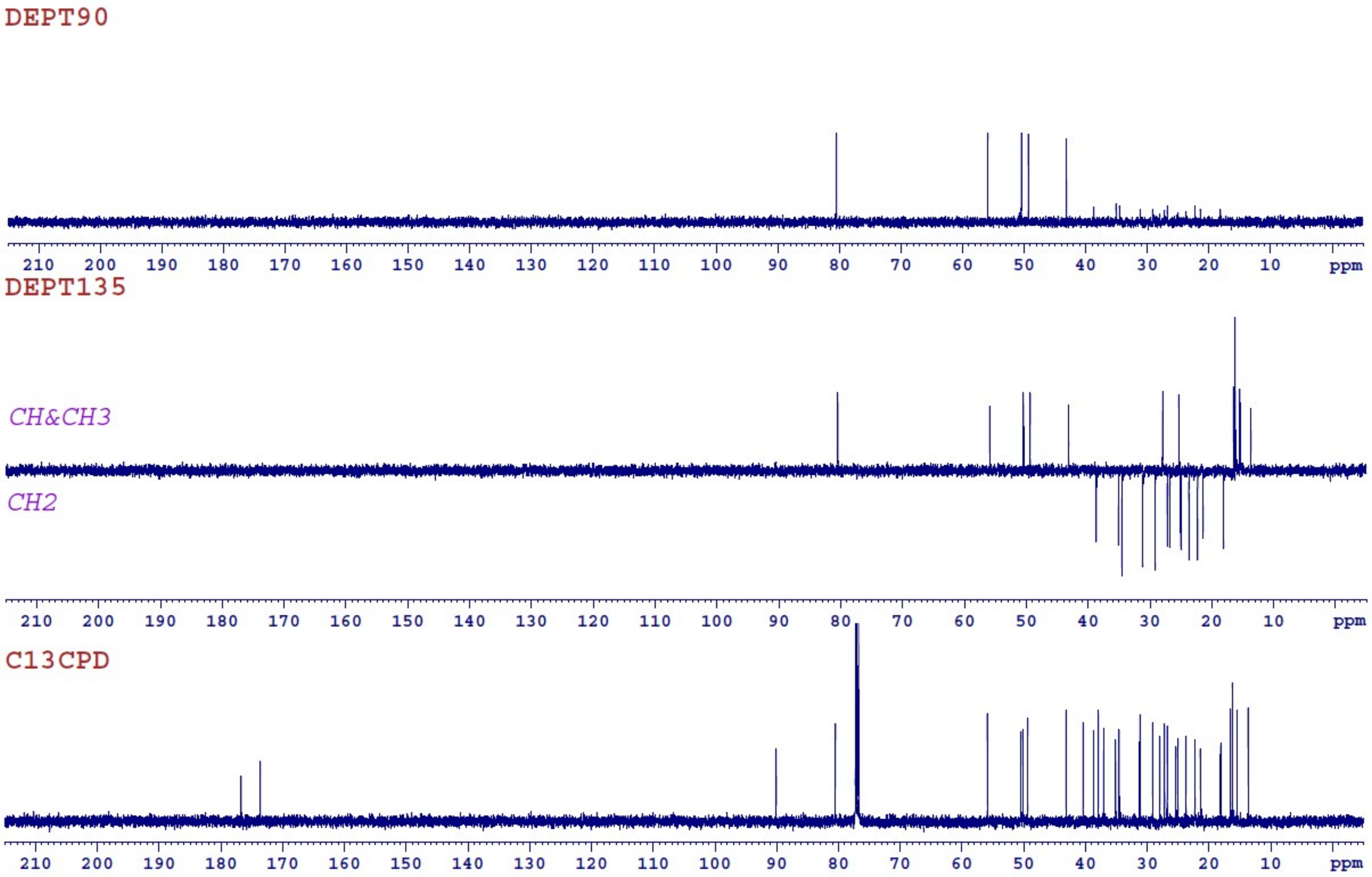


DEPT spectrum of compound **6b**

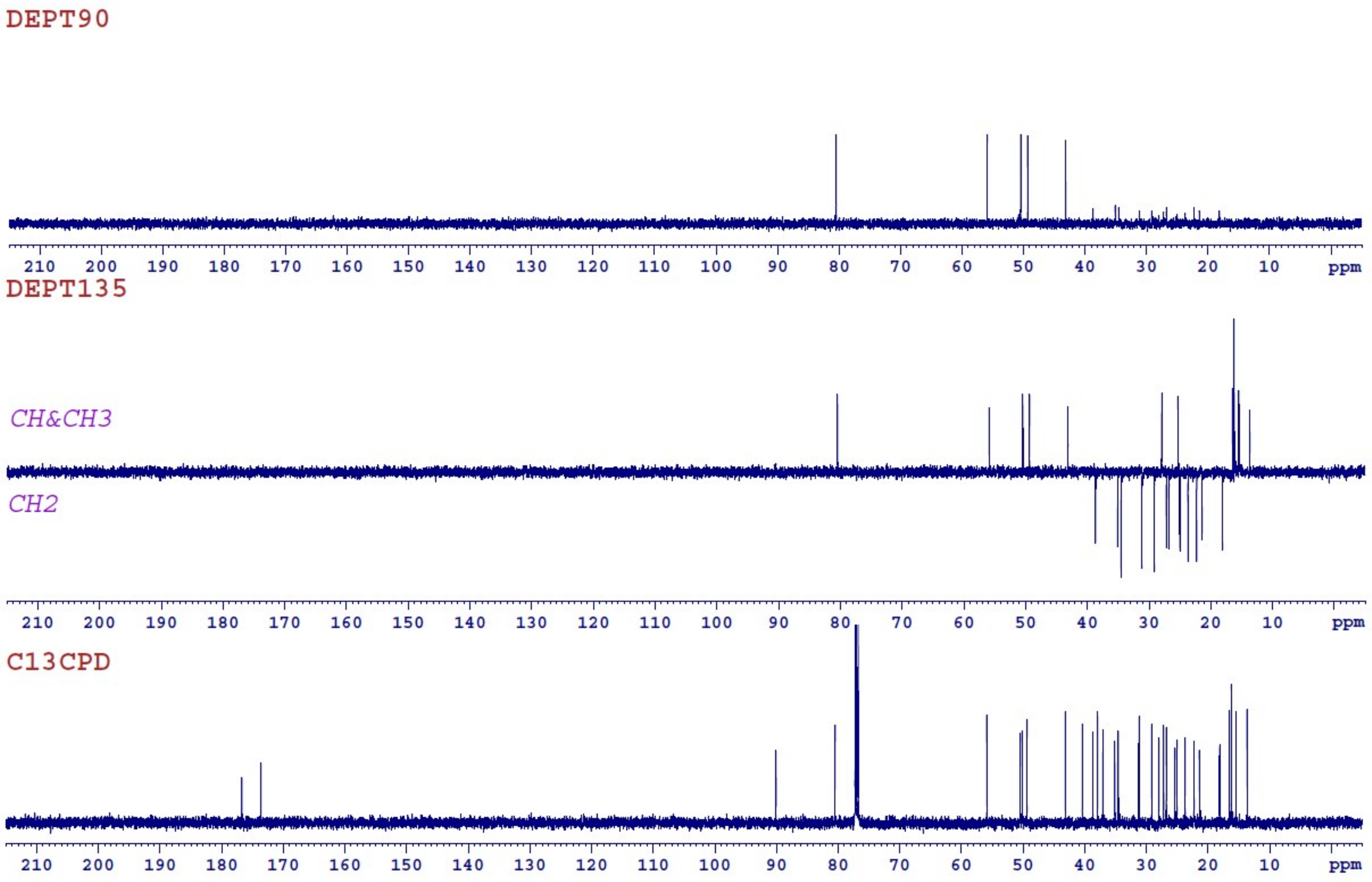


DEPT spectrum of compound **6b** (extension)

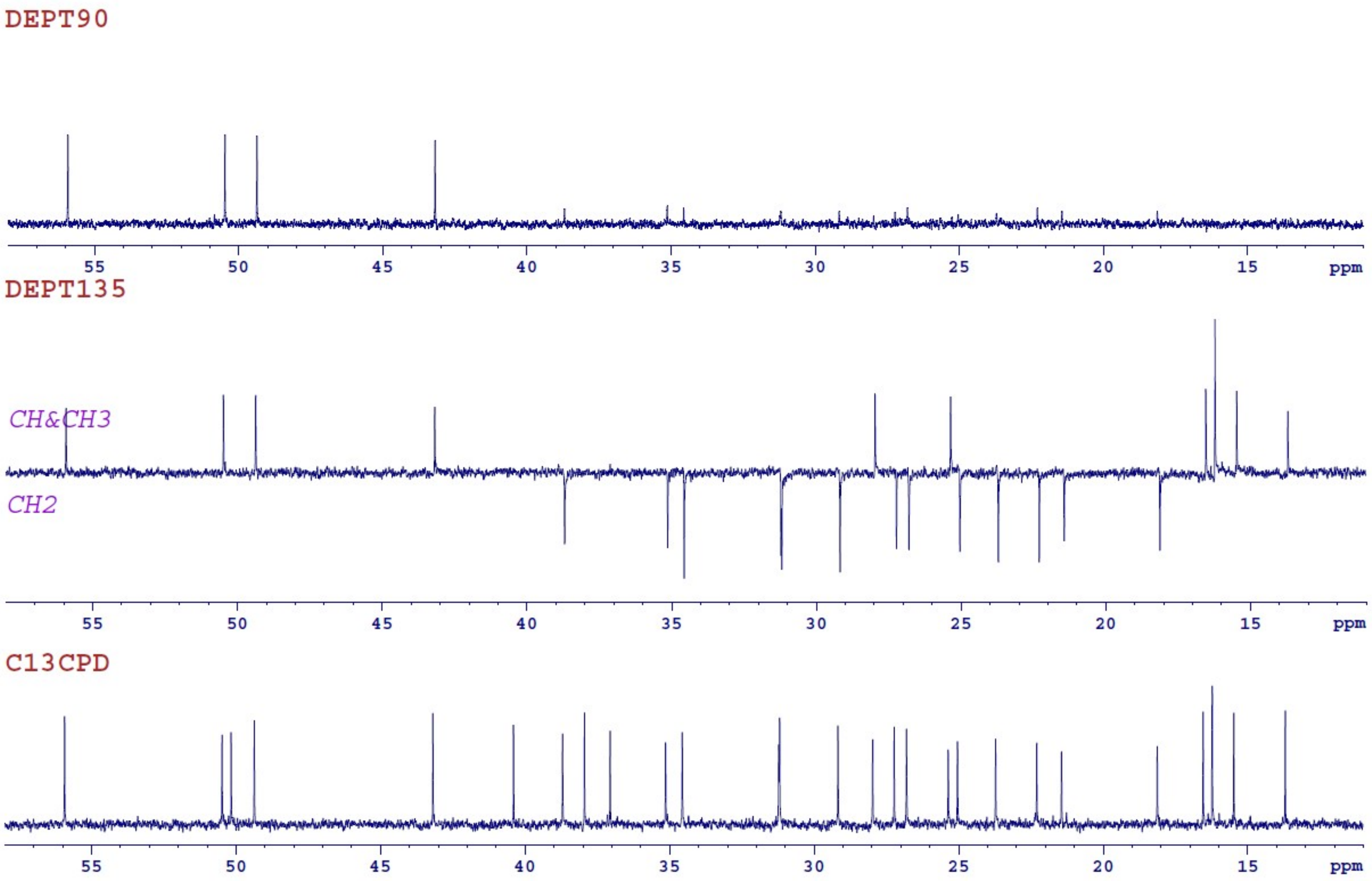


DEPT spectrum of compound **6b** (extension)

**1.19. Compound 6c**

***Sample name: Diplzo***
***Operator: Le Anh VHH***
***Method: +IDA TOF MS/MS***
***Date: 2021.04.23***

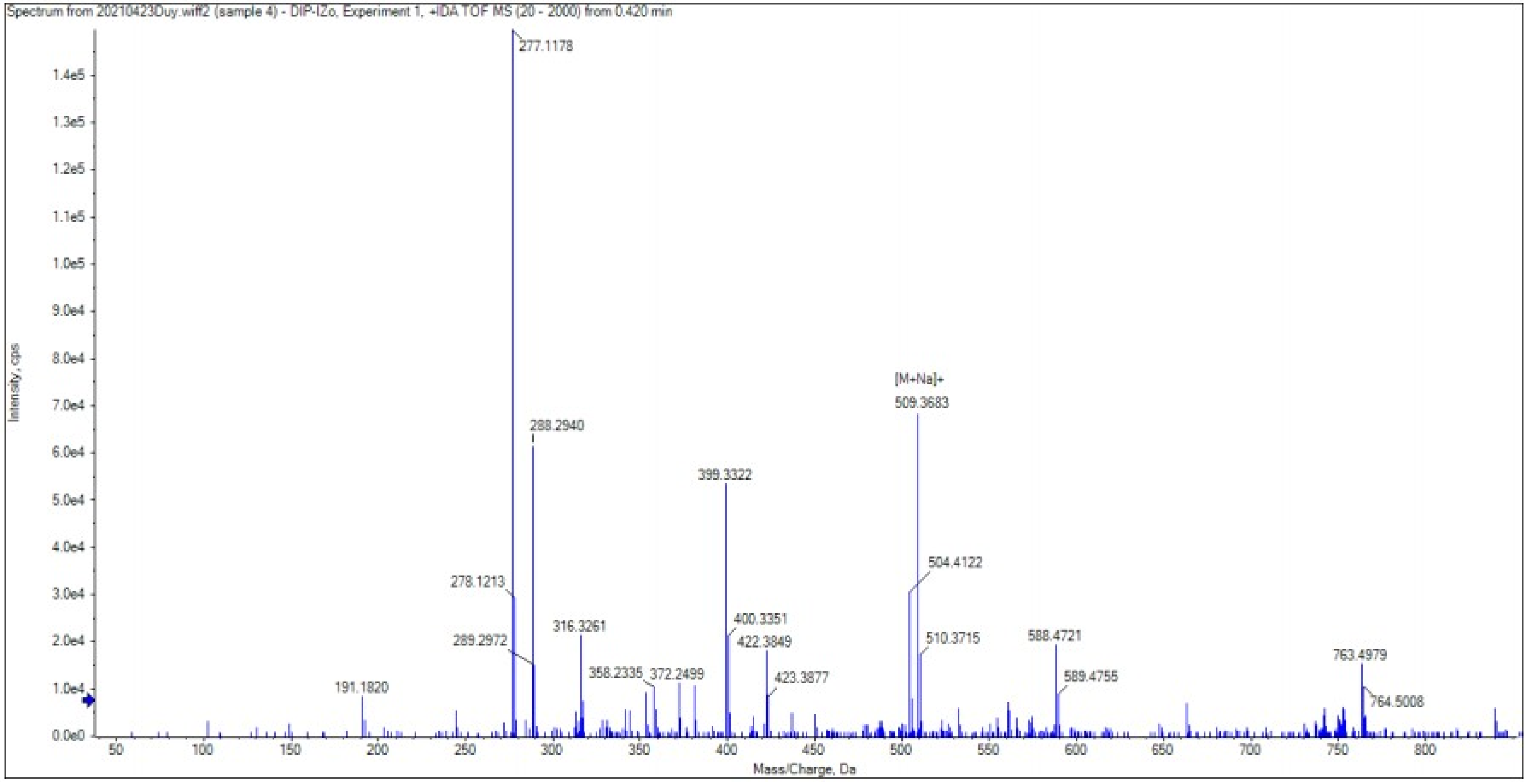


| Hit | Formula | m/z | RDB | ppm | MS Rank | MSMS ppm | MSMS Rank | Found |
|---|---|---|---|---|---|---|---|---|
| 1 | C31H50O4 | 509.36713 | 7.0 | 3.1 | 1 | | | NA/NA |

(+)-HR-ESI-MS spectrum of compound **6c**

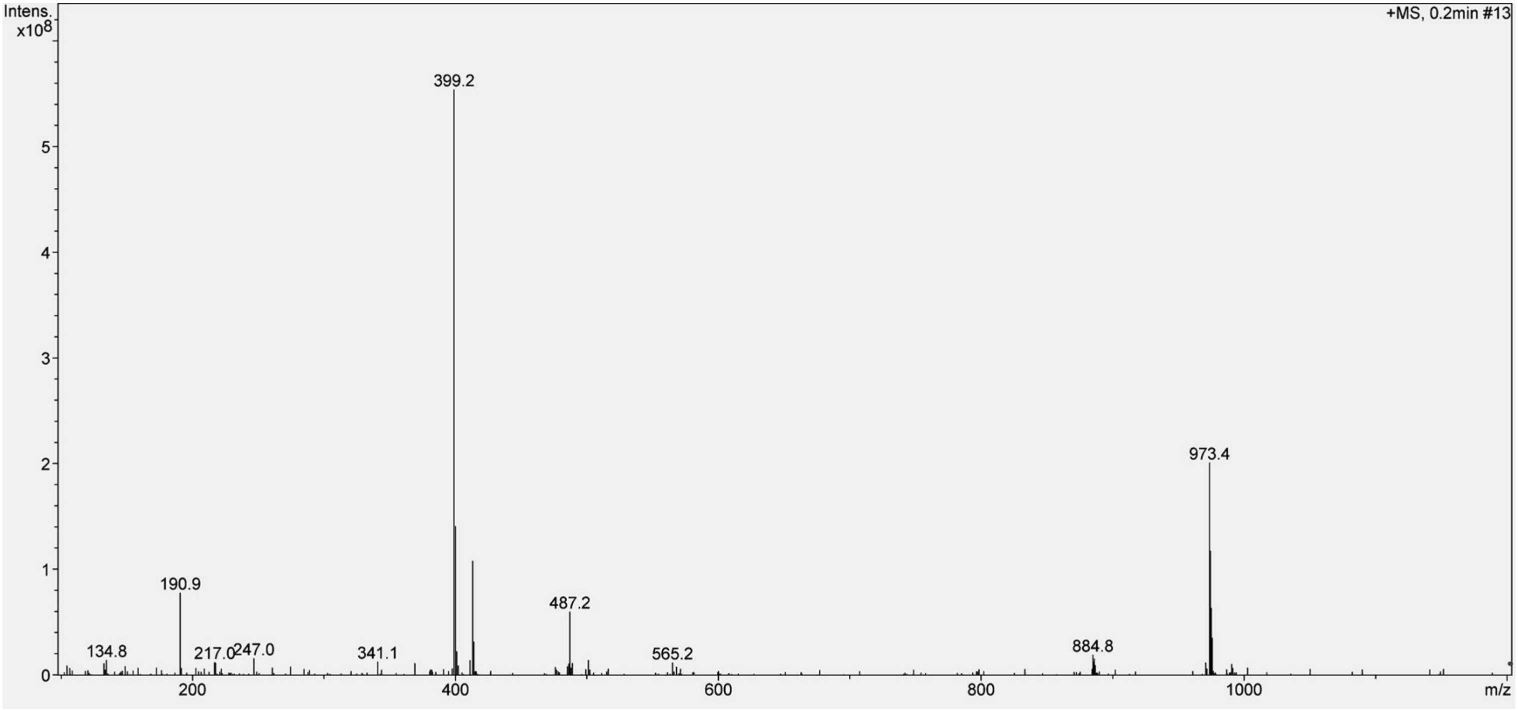


(+)-ESI–MS spectrum of compound **6c**

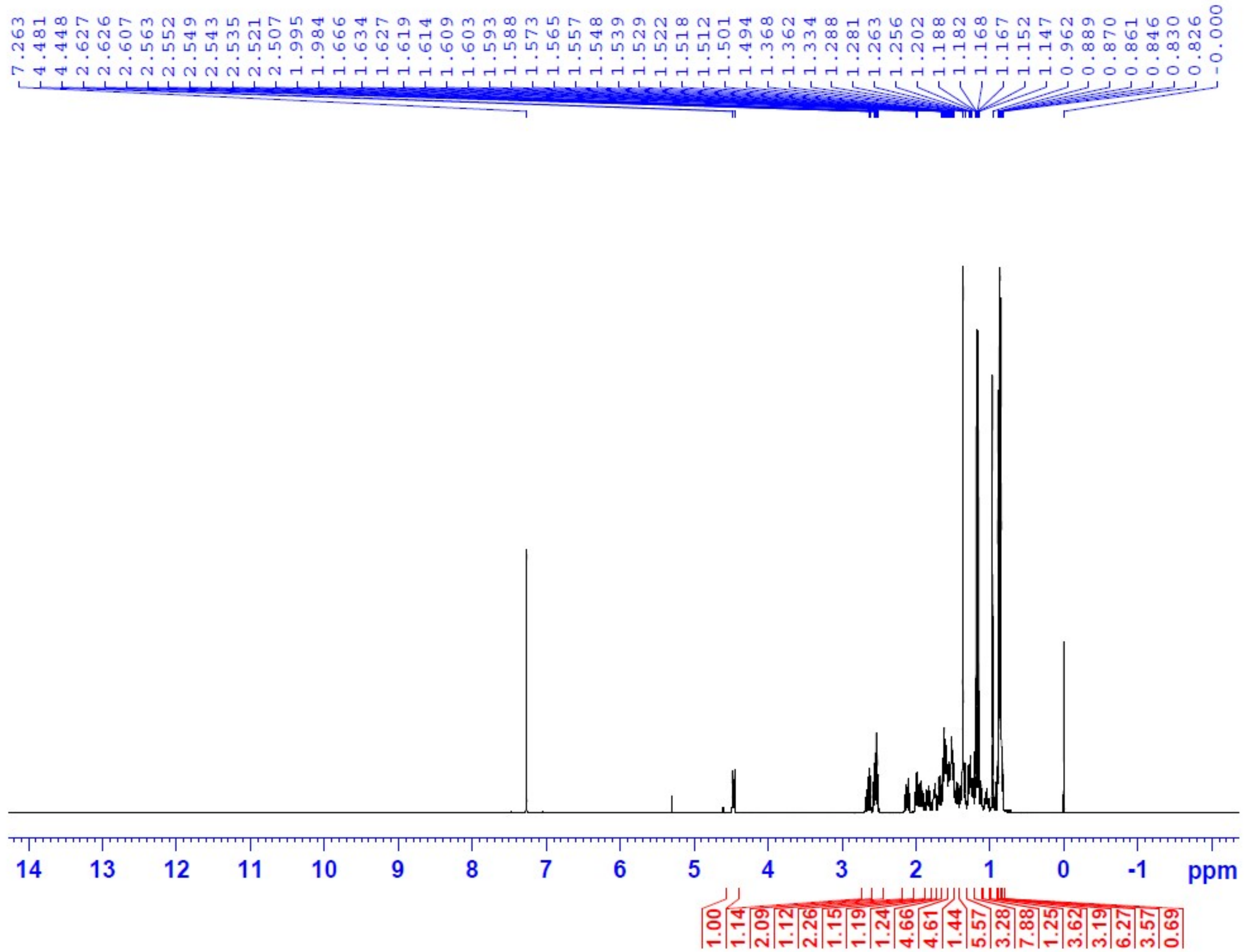

$^{1}$H-NMR spectrum of compound **6c**

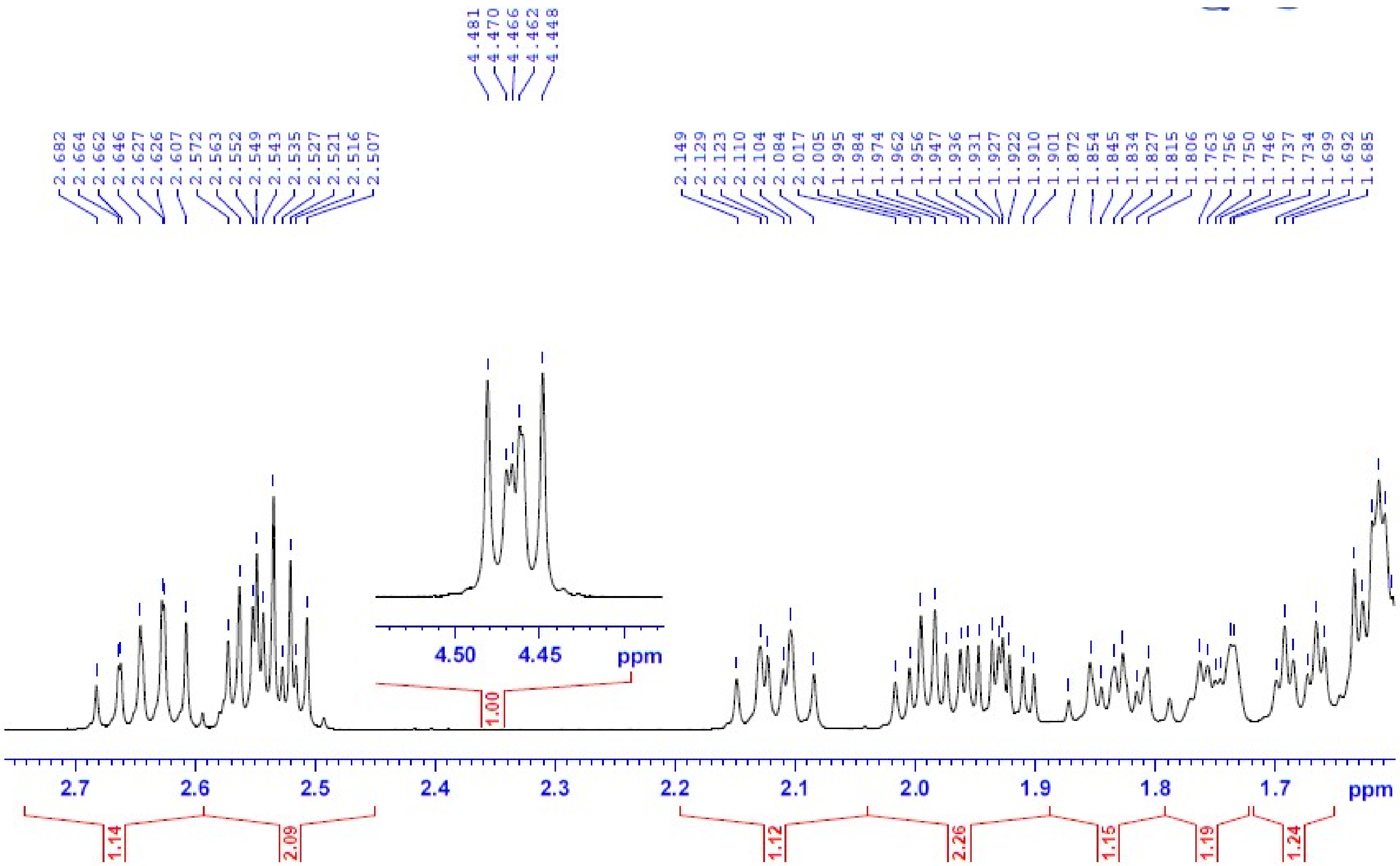
4.50
4.45
ppm
1.00
2.7
2.6
2.5
2.4
2.3
2.2
2.1
2.0
1.9
1.8
1.7
ppm
1.14
2.09
1.12
2.26
1.15
1.19
1.24

$^{1}$H-NMR spectrum of compound **6c** (extension)

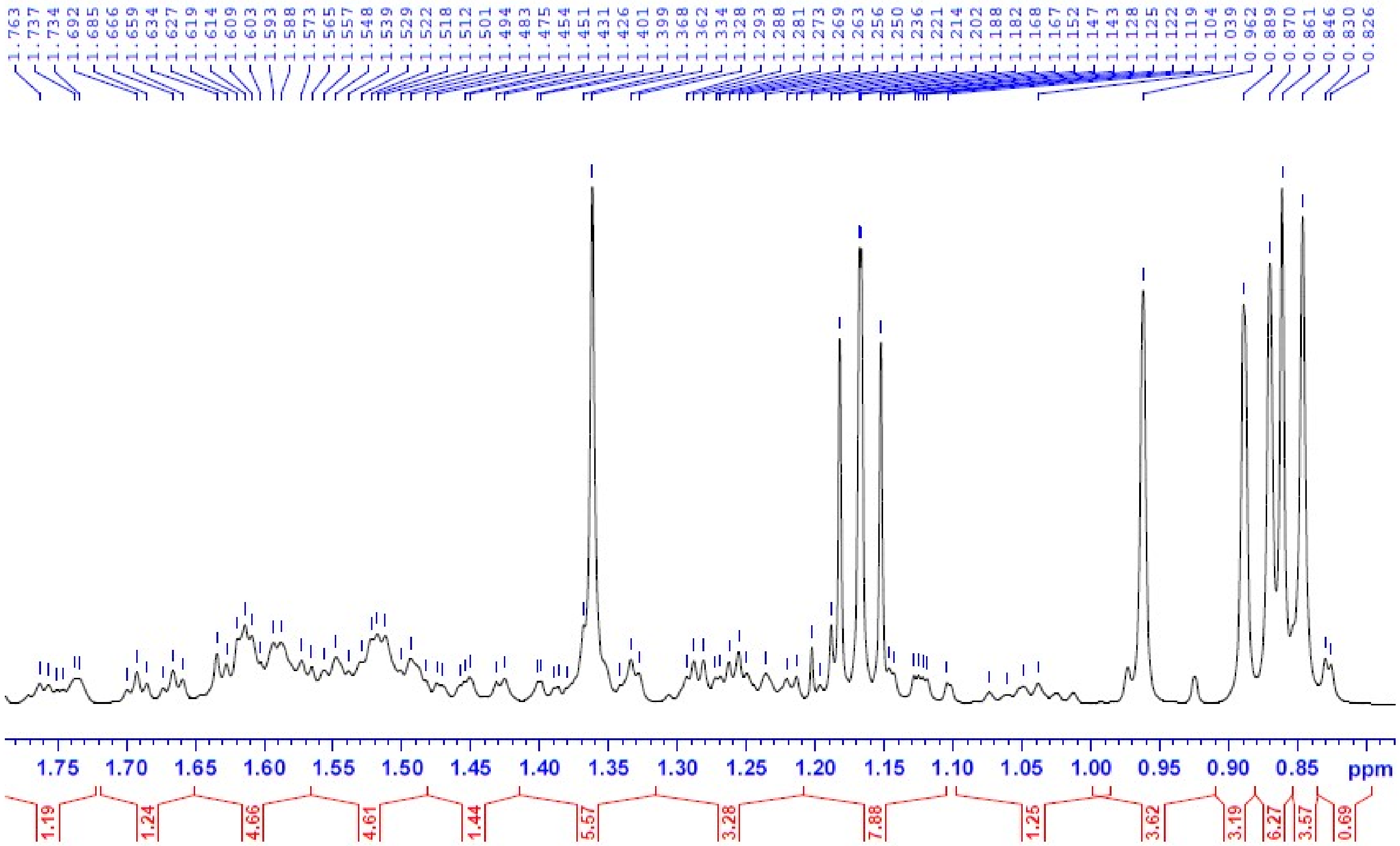

$^{1}$H-NMR spectrum of compound **6c** (extension)

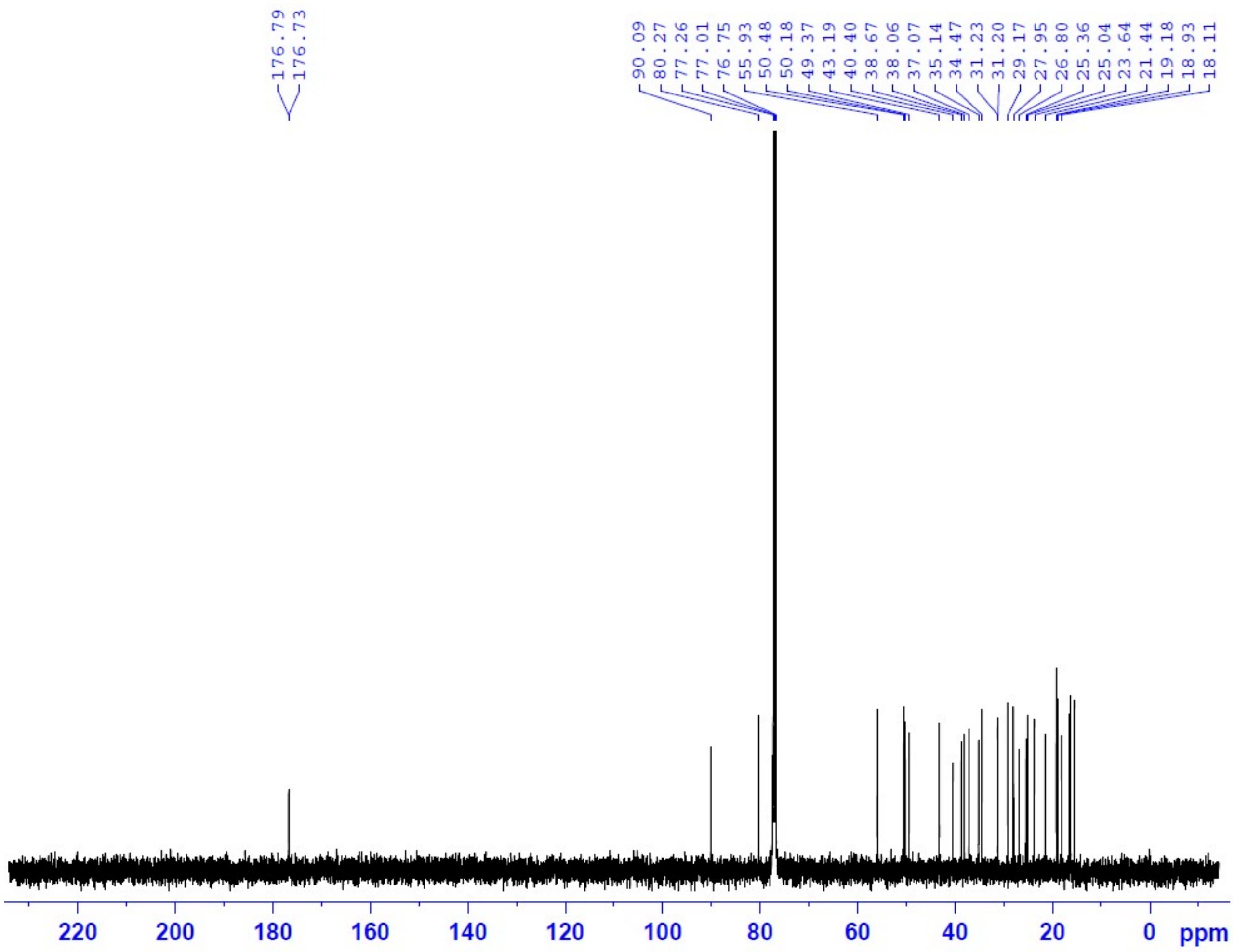


$^{13}$C-NMR spectrum of compound **6c**

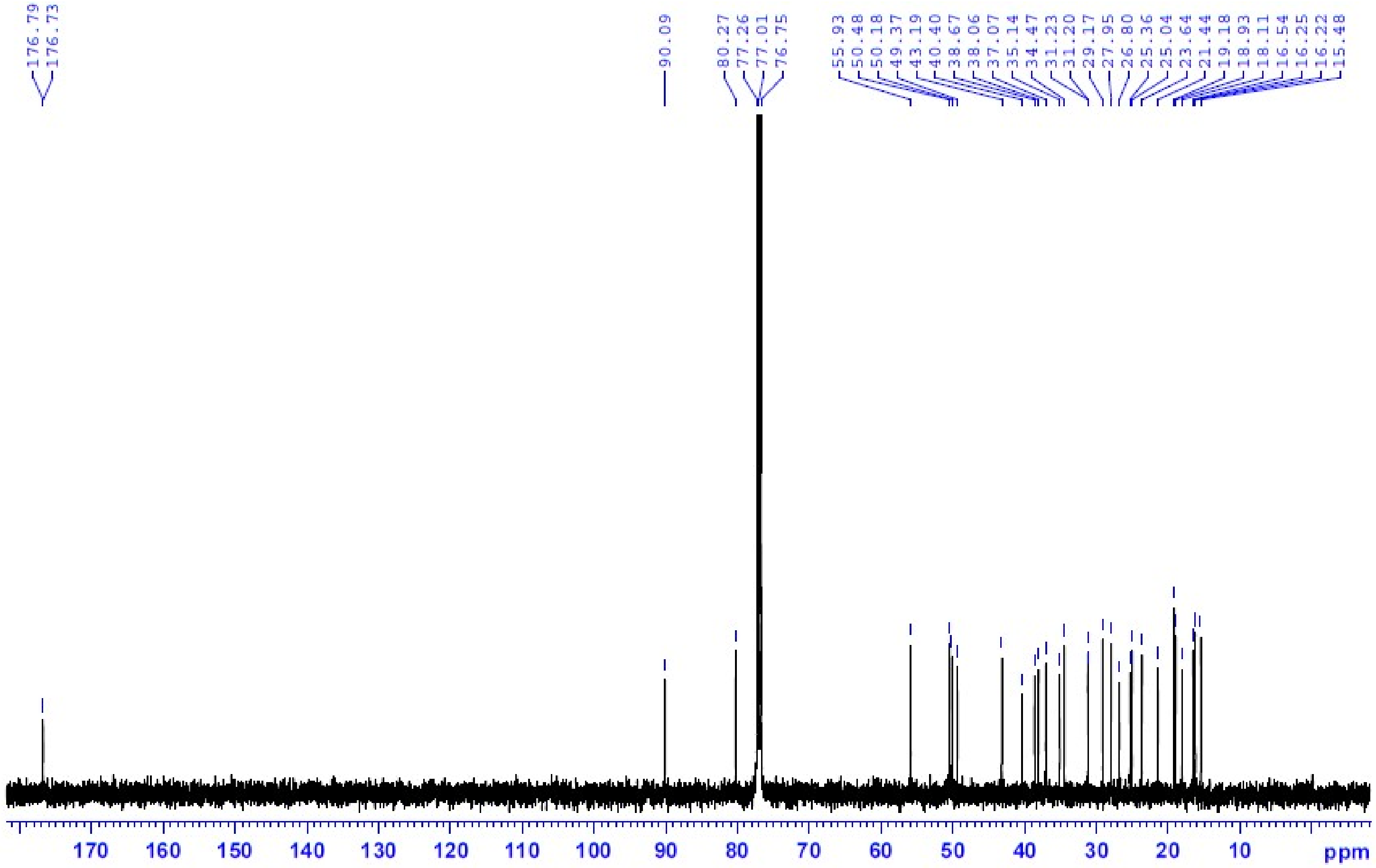
176.79
176.73
90.09
80.27
77.26
77.01
76.75
55.93
50.48
50.18
49.37
43.19
40.40
38.67
38.06
37.07
35.14
34.47
31.23
31.20
29.17
27.95
26.80
25.36
25.04
23.64
21.44
19.18
18.93
18.11
16.54
16.25
16.22
15.48
170 160 150 140 130 120 110 100 90 80 70 60 50 40 30 20 10 ppm

$^{13}$C-NMR spectrum of compound **6c** (extension)

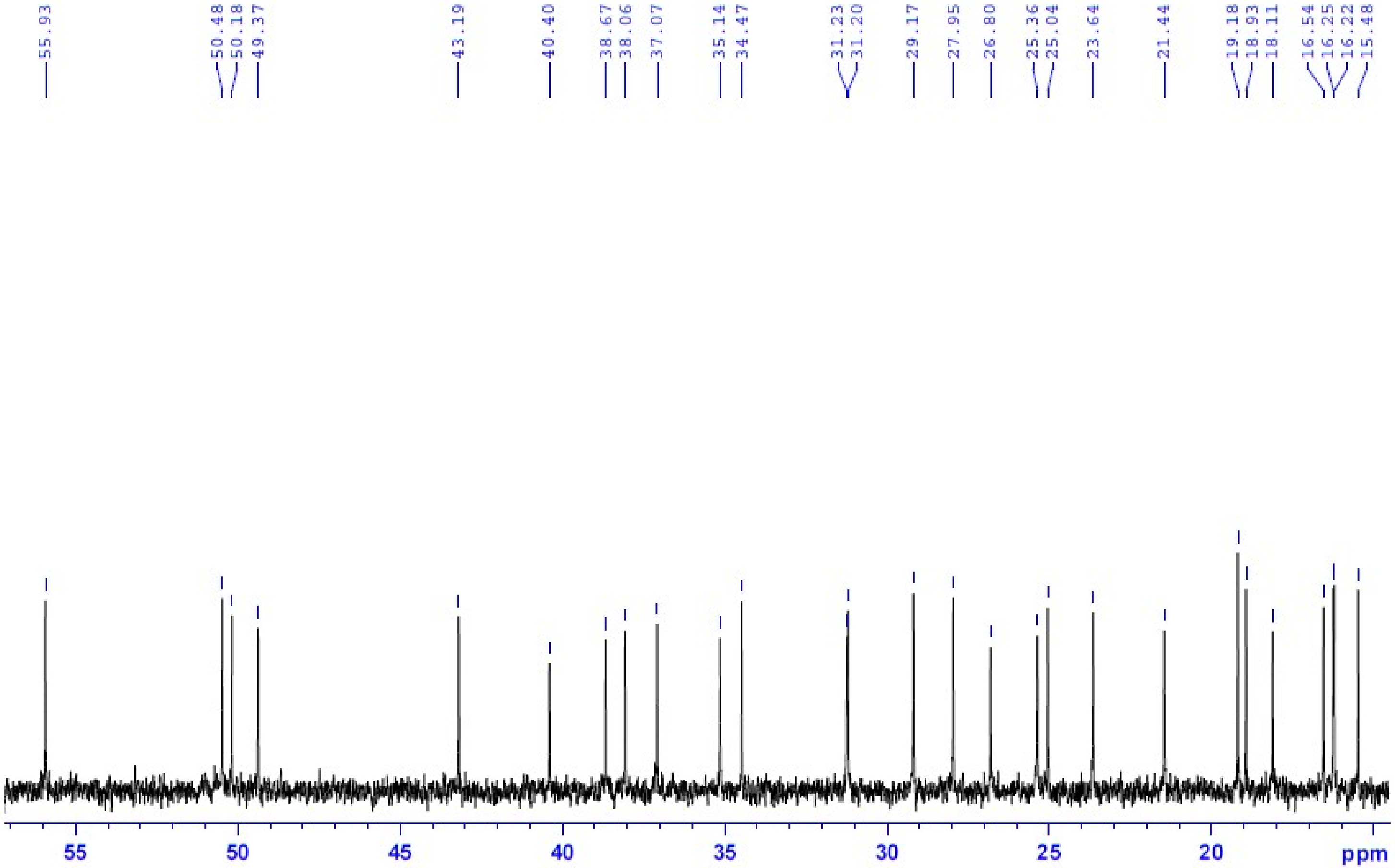
55.93
50.48
50.18
49.37
43.19
40.40
38.67
38.06
37.07
35.14
34.47
31.23
31.20
29.17
27.95
26.80
25.36
25.04
23.64
21.44
19.18
18.93
18.11
16.54
16.25
16.22
15.48
55
50
45
40
35
30
25
20
ppm

$^{13}C$-NMR spectrum of compound **6c** (extension)

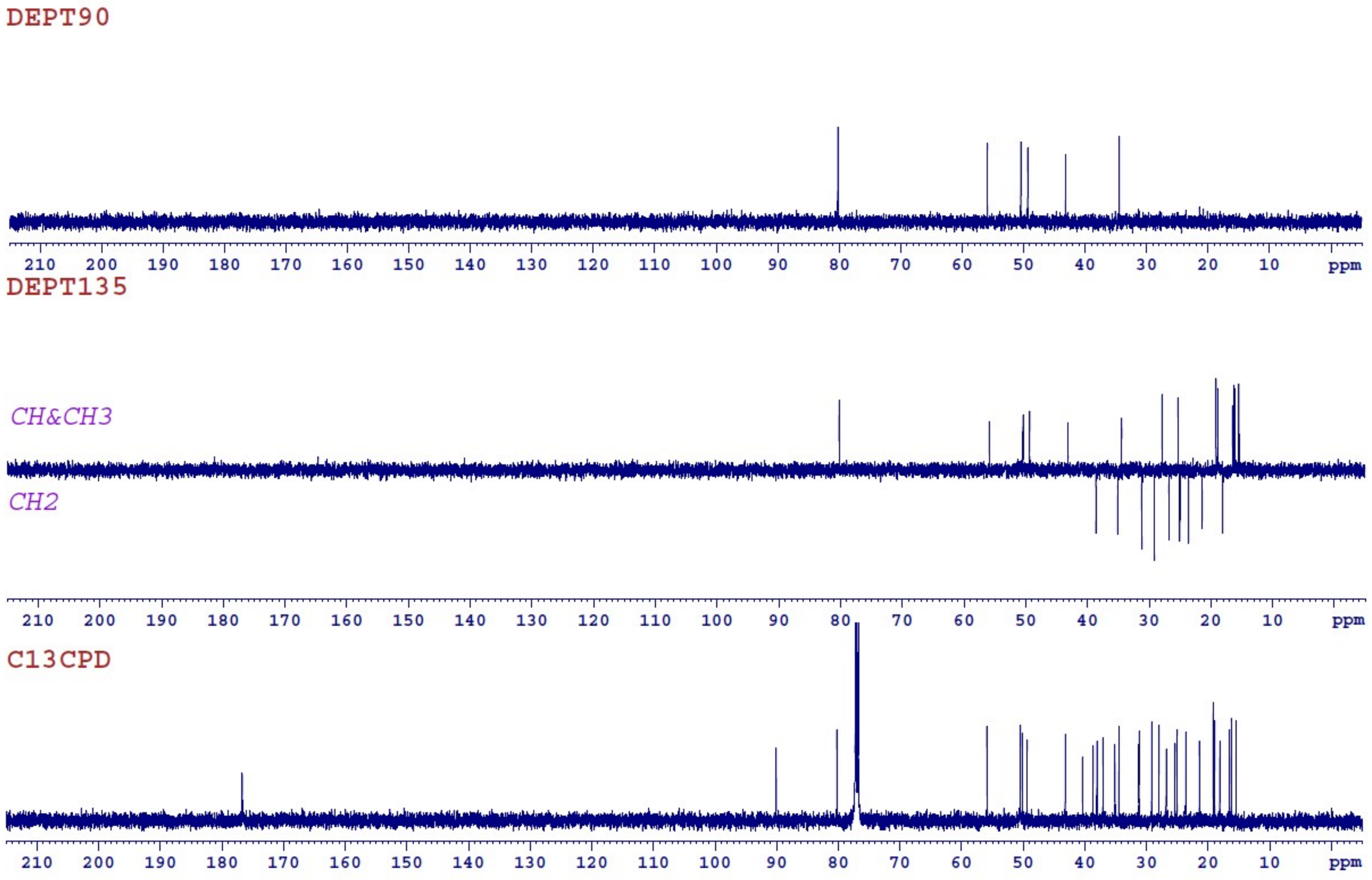


DEPT spectrum of compound **6c**

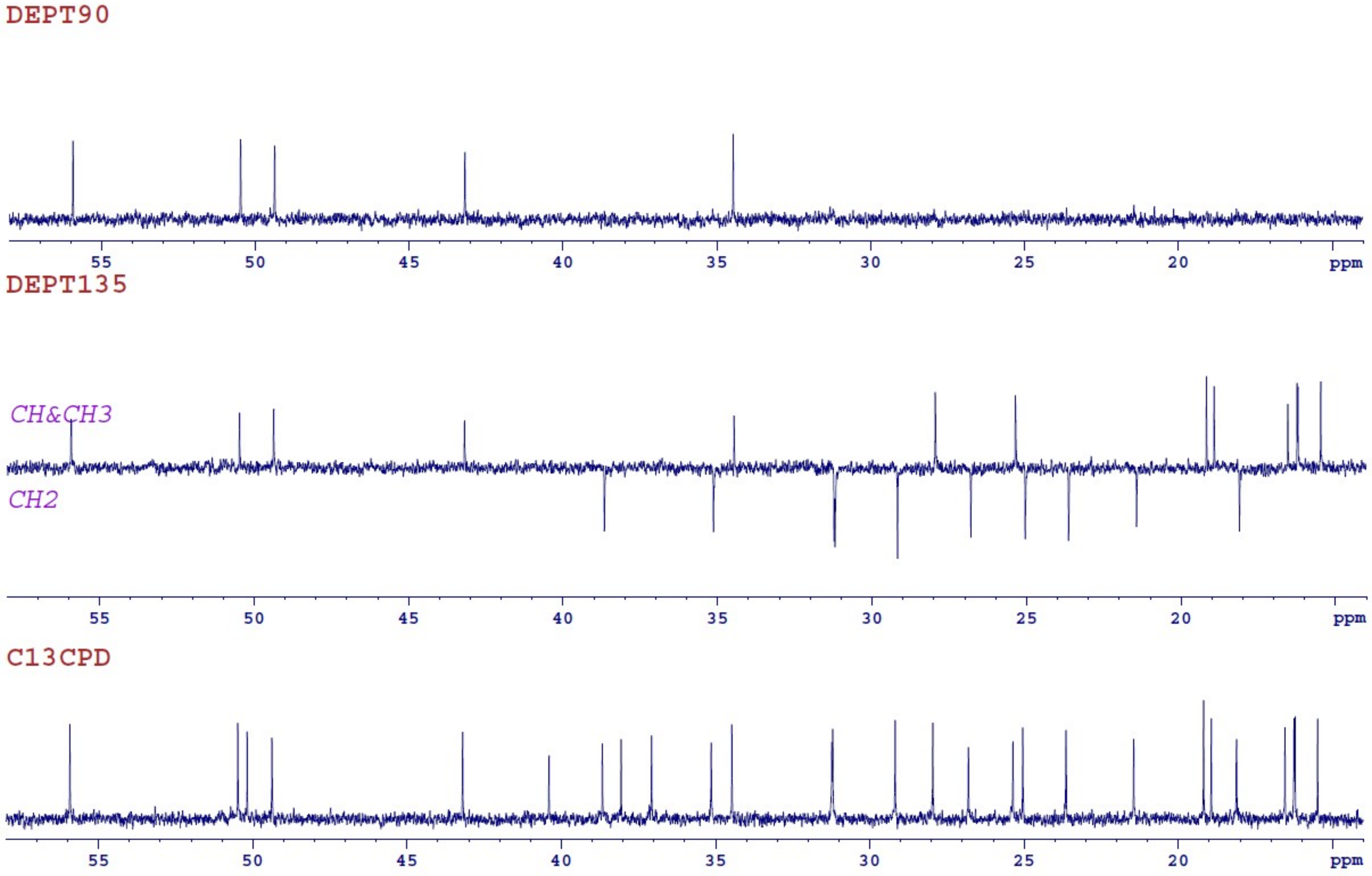

DEPT spectrum of compound **6c** (extension)

### 1.20. Compound 6d

***Sample name: DipSali***
***Operator: Le Anh VHH***
***Method: +IDA TOF MS/MS***
***Date: 2021.04.23***

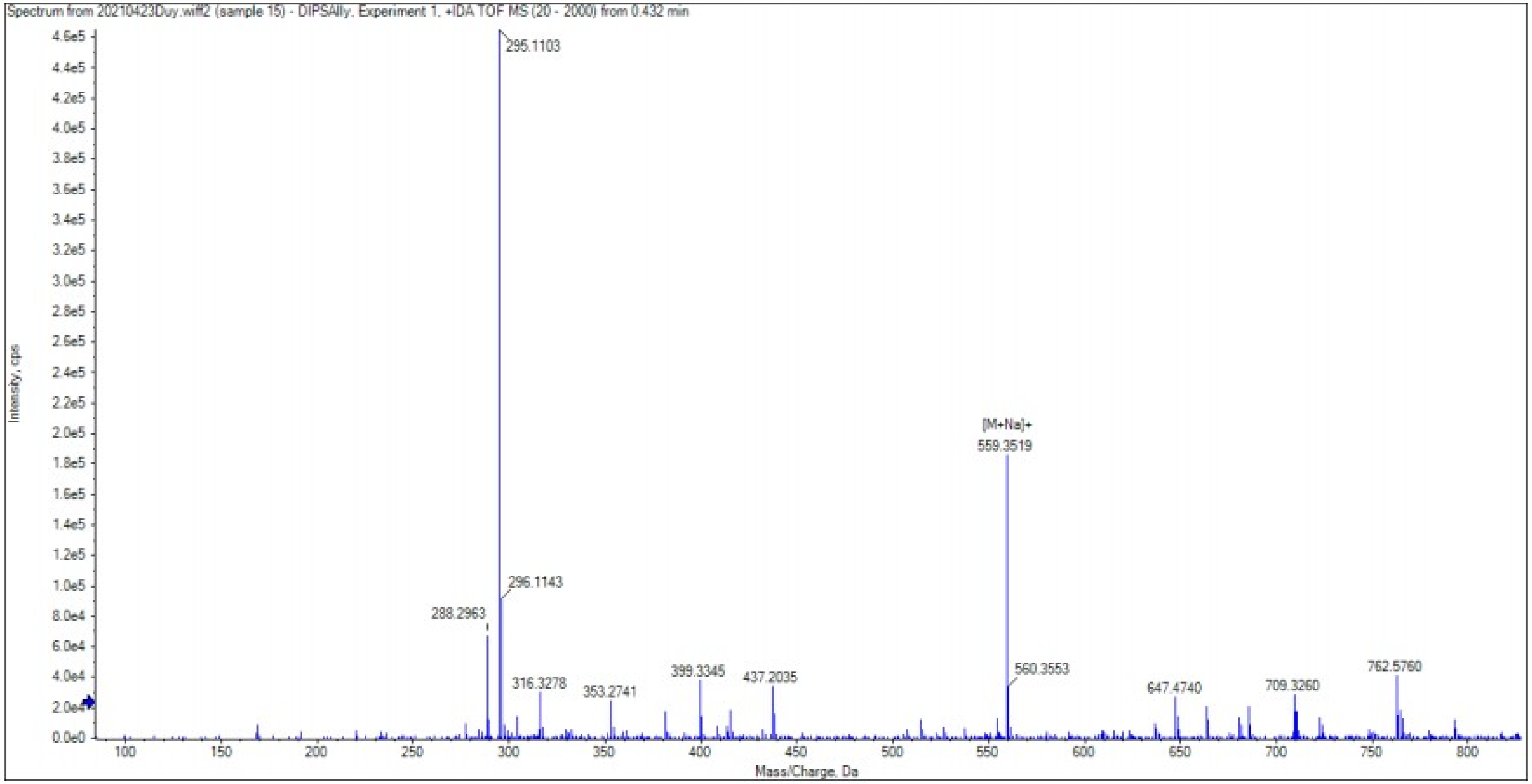


| Hit | Formula | m/z | RDB | ppm | MS Rank | MSMS ppm | MSMS Rank | Found |
|---|---|---|---|---|---|---|---|---|
| 1 | C34H48O5 | 559.34940 | 11.0 | 2.4 | 1 | | | NA/NA |

(+)-HR-ESI-MS spectrum of compound **6d**

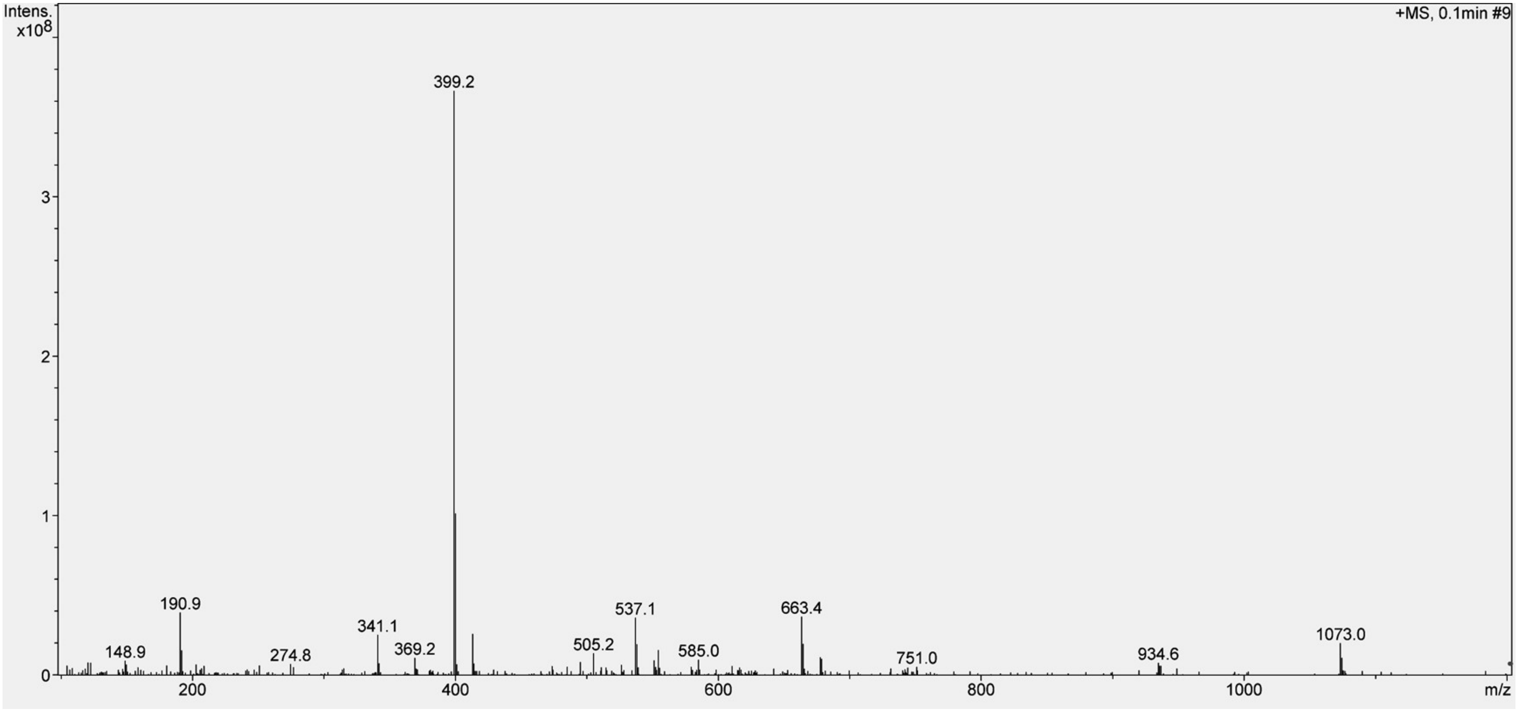


(+)-ESI–MS spectrum of compound **6d**

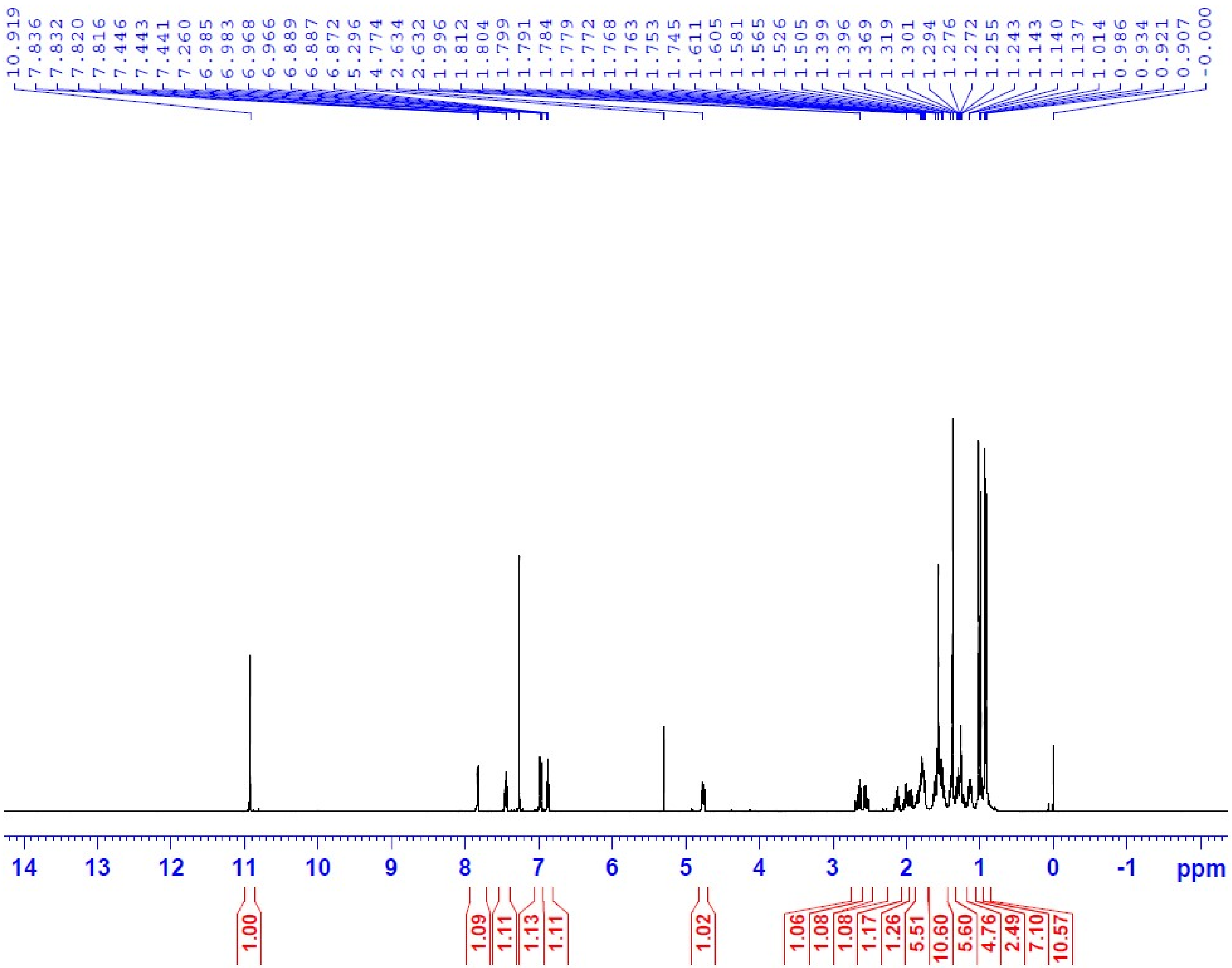

$^{1}$H-NMR spectrum of compound **6d**

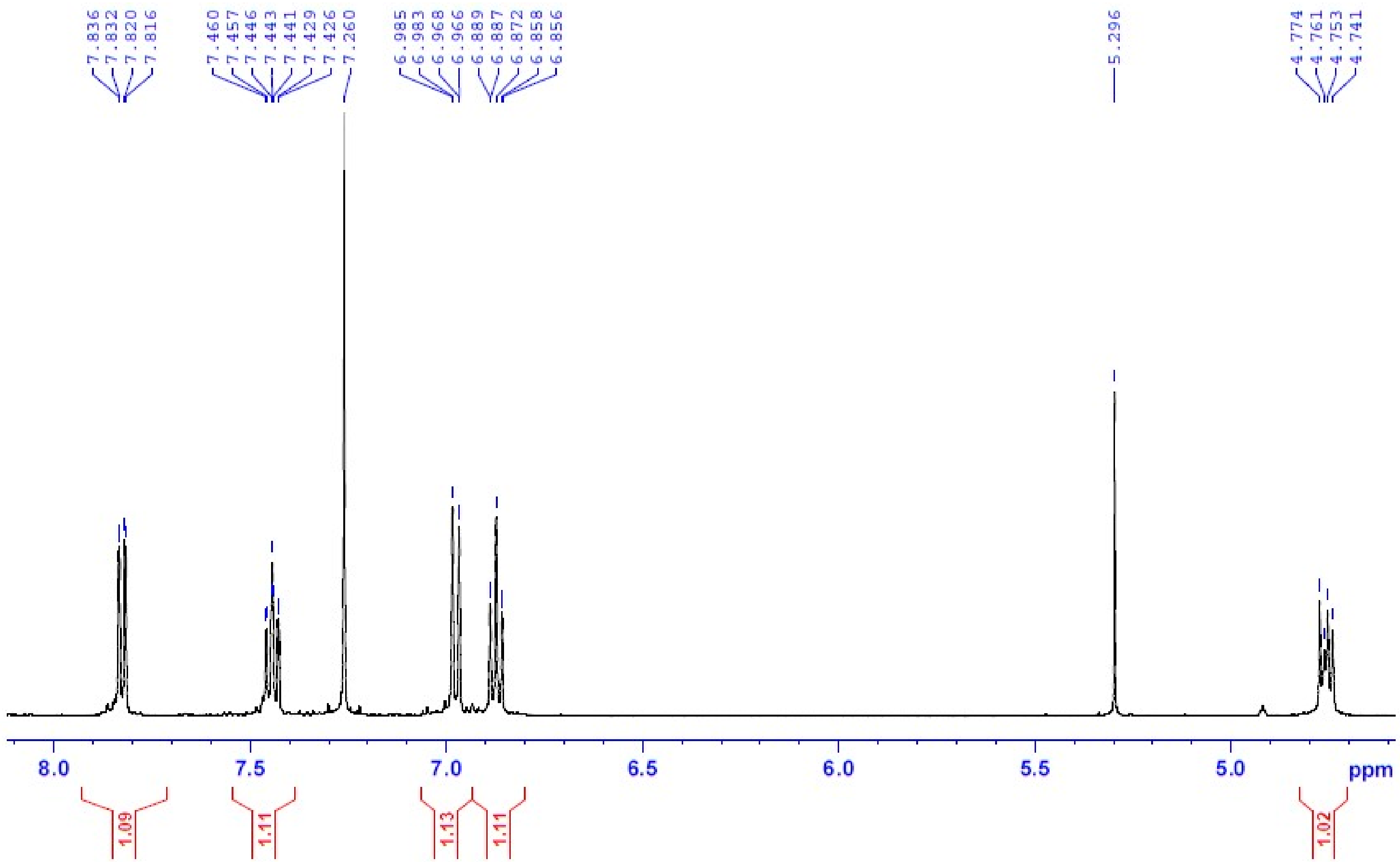
7.836
7.832
7.820
7.816
7.460
7.457
7.446
7.443
7.441
7.429
7.426
7.260
6.985
6.983
6.968
6.966
6.889
6.887
6.872
6.858
6.856
5.296
4.774
4.761
4.753
4.741
8.0
7.5
7.0
6.5
6.0
5.5
5.0
ppm
1.09
1.11
1.13
1.11
1.02

$^{1}$H-NMR spectrum of compound **6d** (extension)

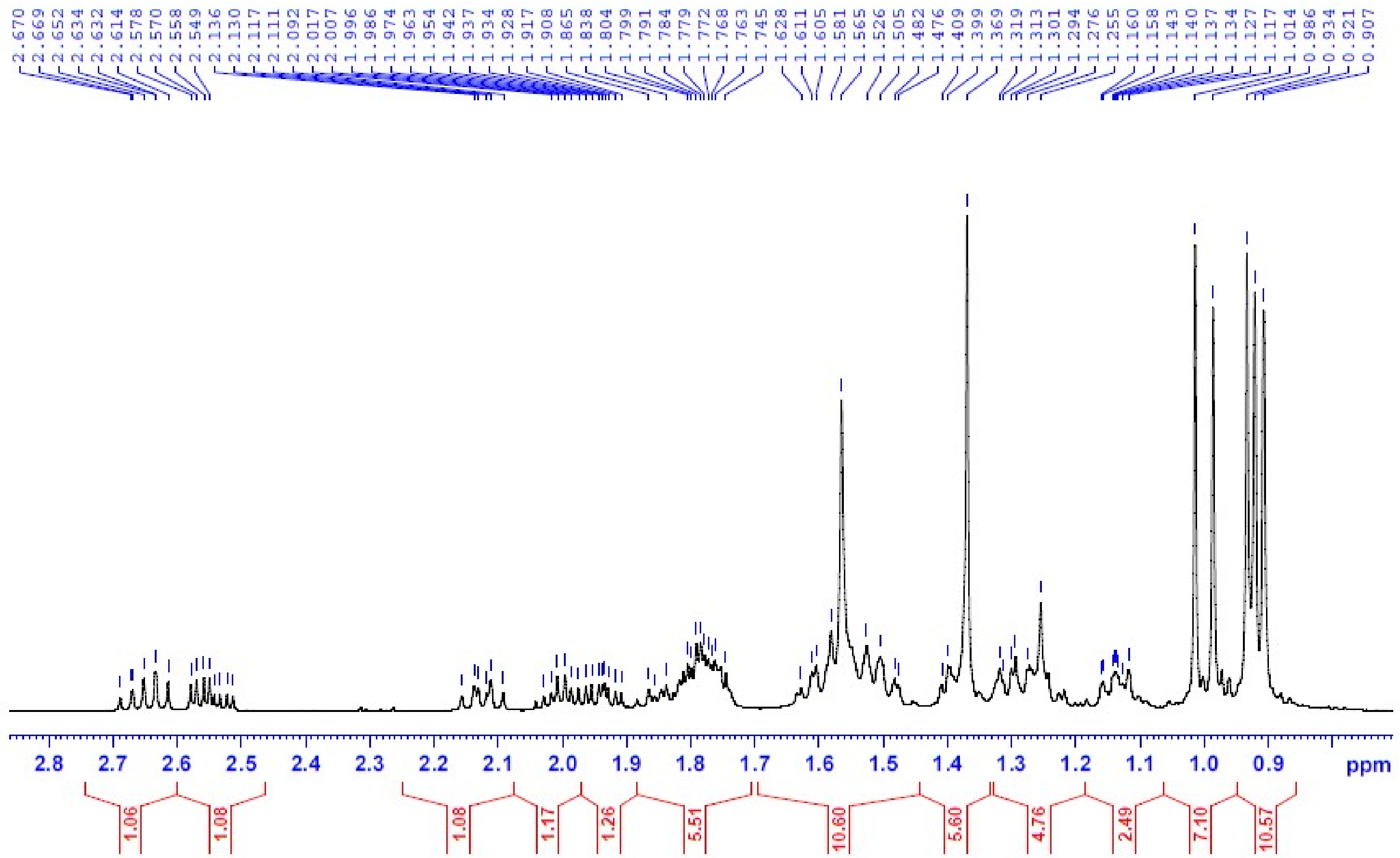

$^{1}$H-NMR spectrum of compound **6d** (extension)

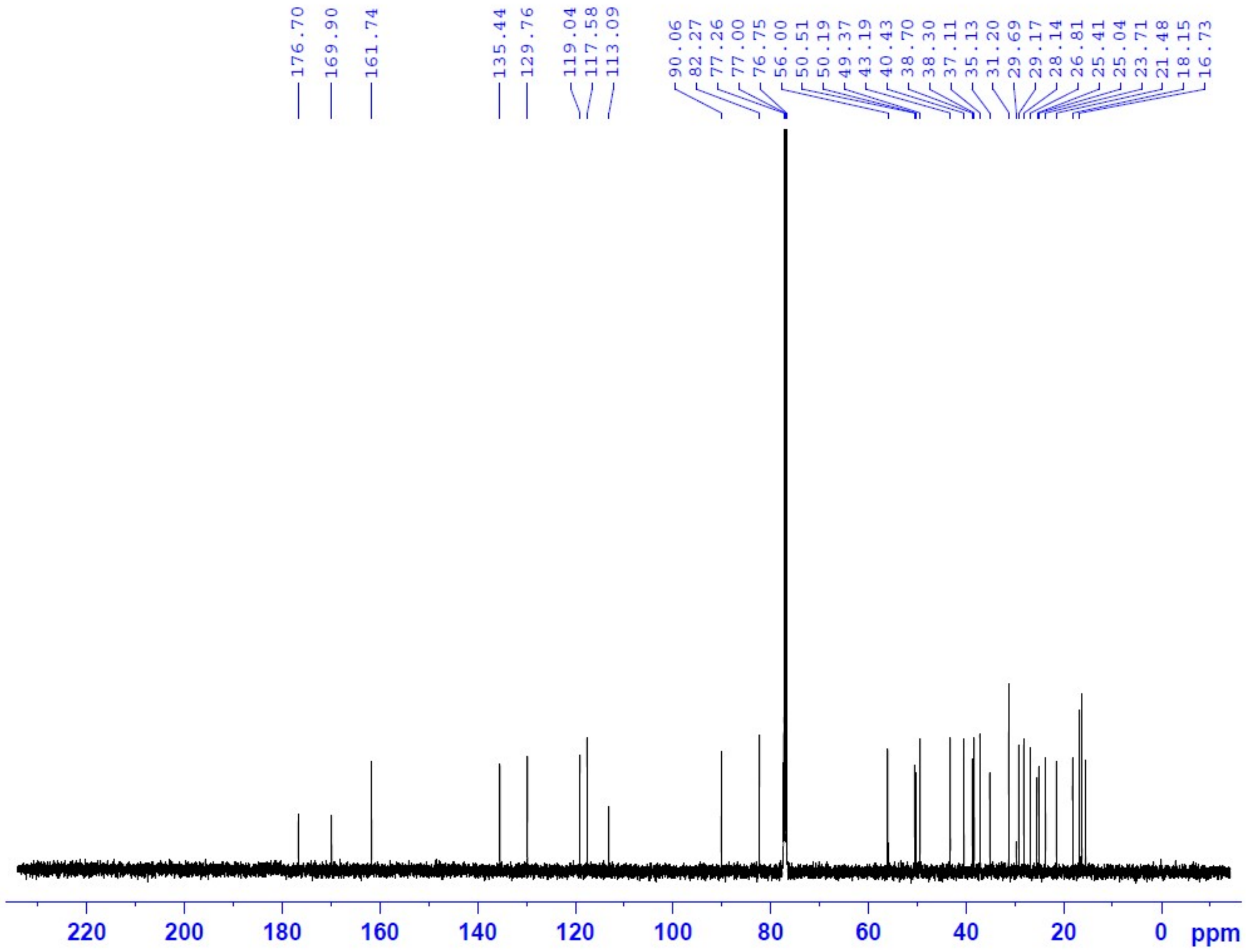


$^{13}$C-NMR spectrum of compound **6d**

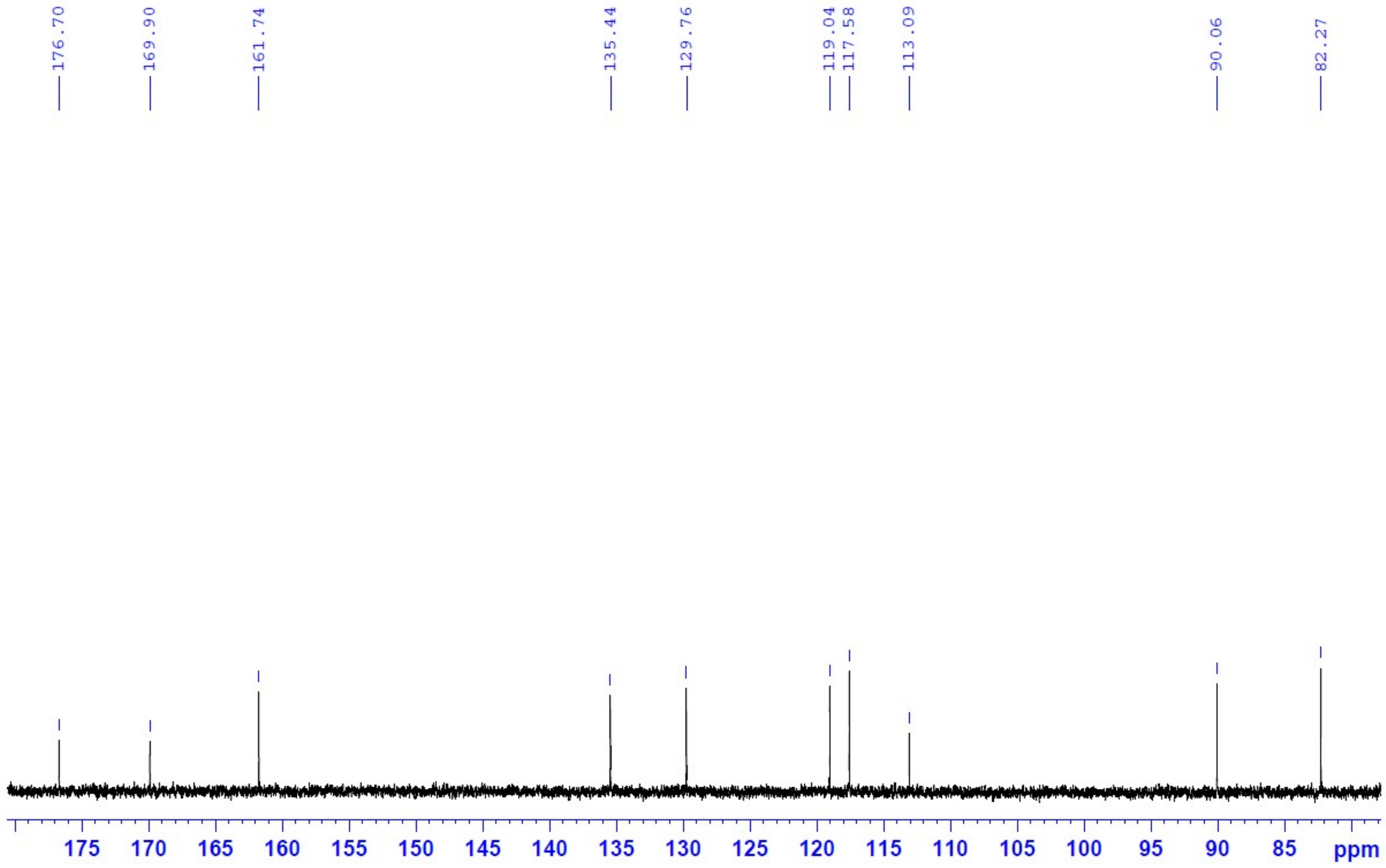


$^{13}$C-NMR spectrum of compound **6d** (extension)

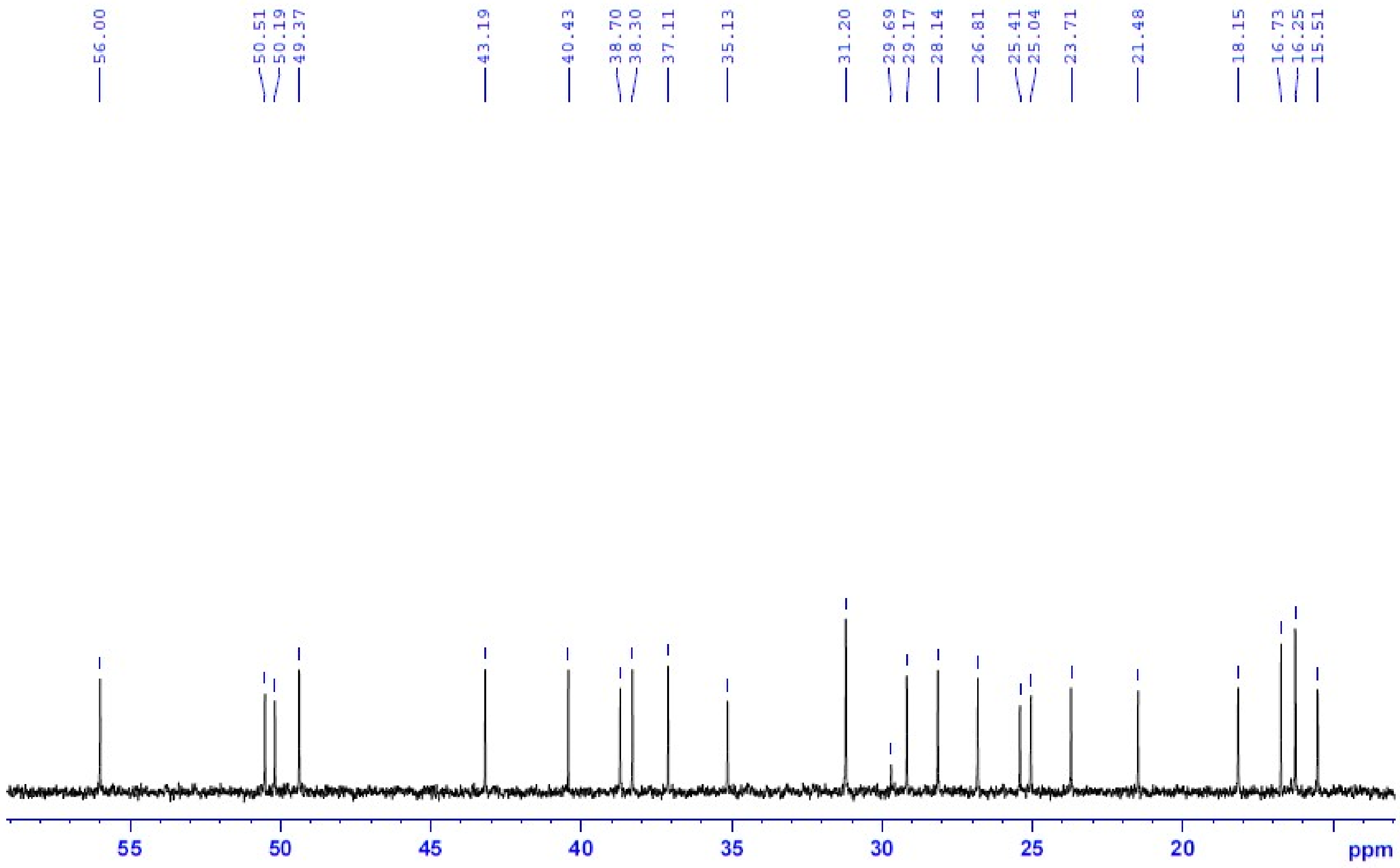
56.00
50.51
50.19
49.37
43.19
40.43
38.70
38.30
37.11
35.13
31.20
29.69
29.17
28.14
26.81
25.41
25.04
23.71
21.48
18.15
16.73
16.25
15.51
55
50
45
40
35
30
25
20
ppm

$^{13}$C-NMR spectrum of compound **6d** (extension)

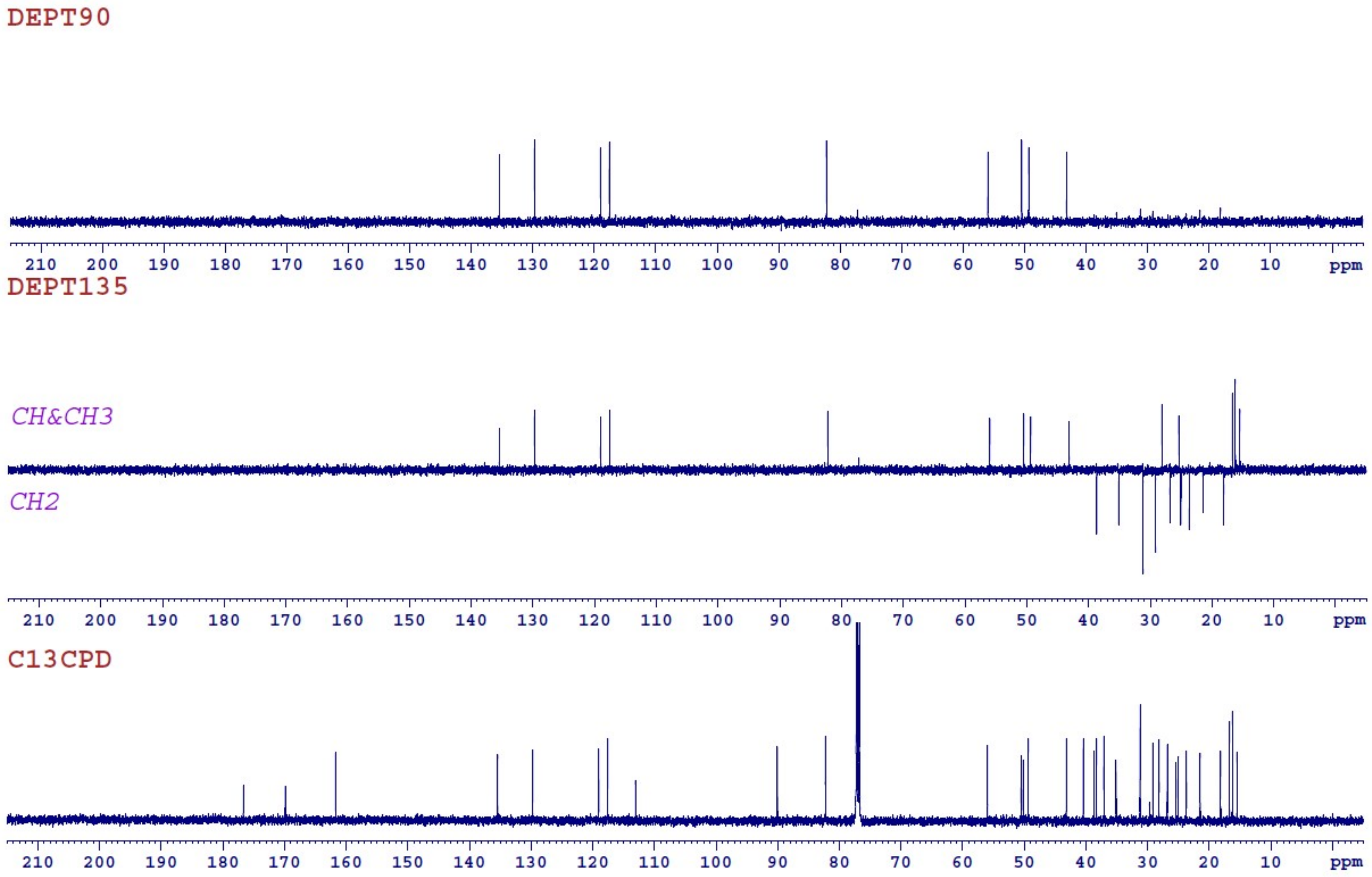


DEPT spectrum of compound **6d**

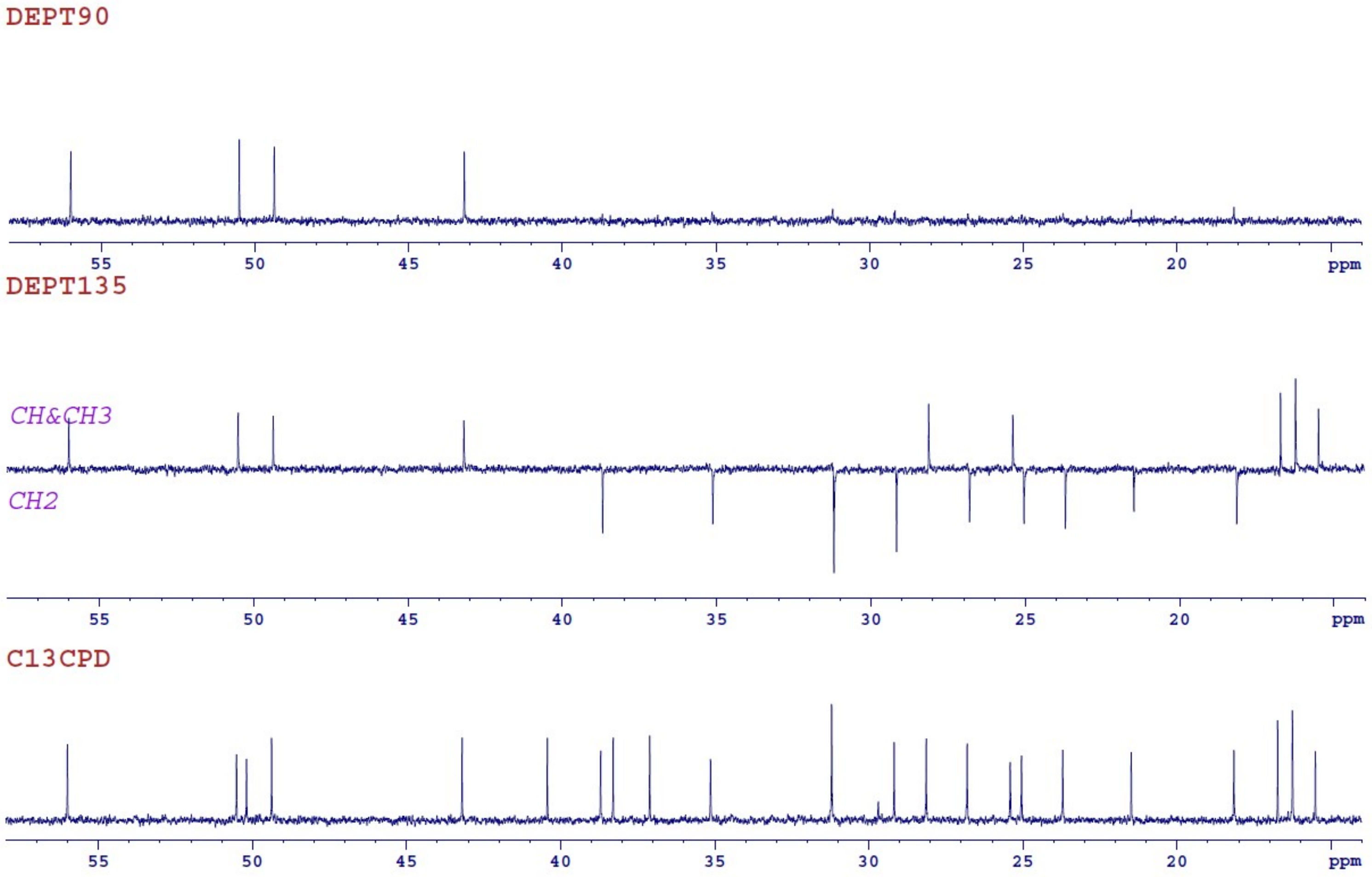


DEPT spectrum of compound **6d** (extension)

### 1.21. Compound 6e

***Sample name: DipCima***
***Operator: Le Anh VHH***
***Method: +IDA TOF MS/MS***
***Date: 2021.04.23***

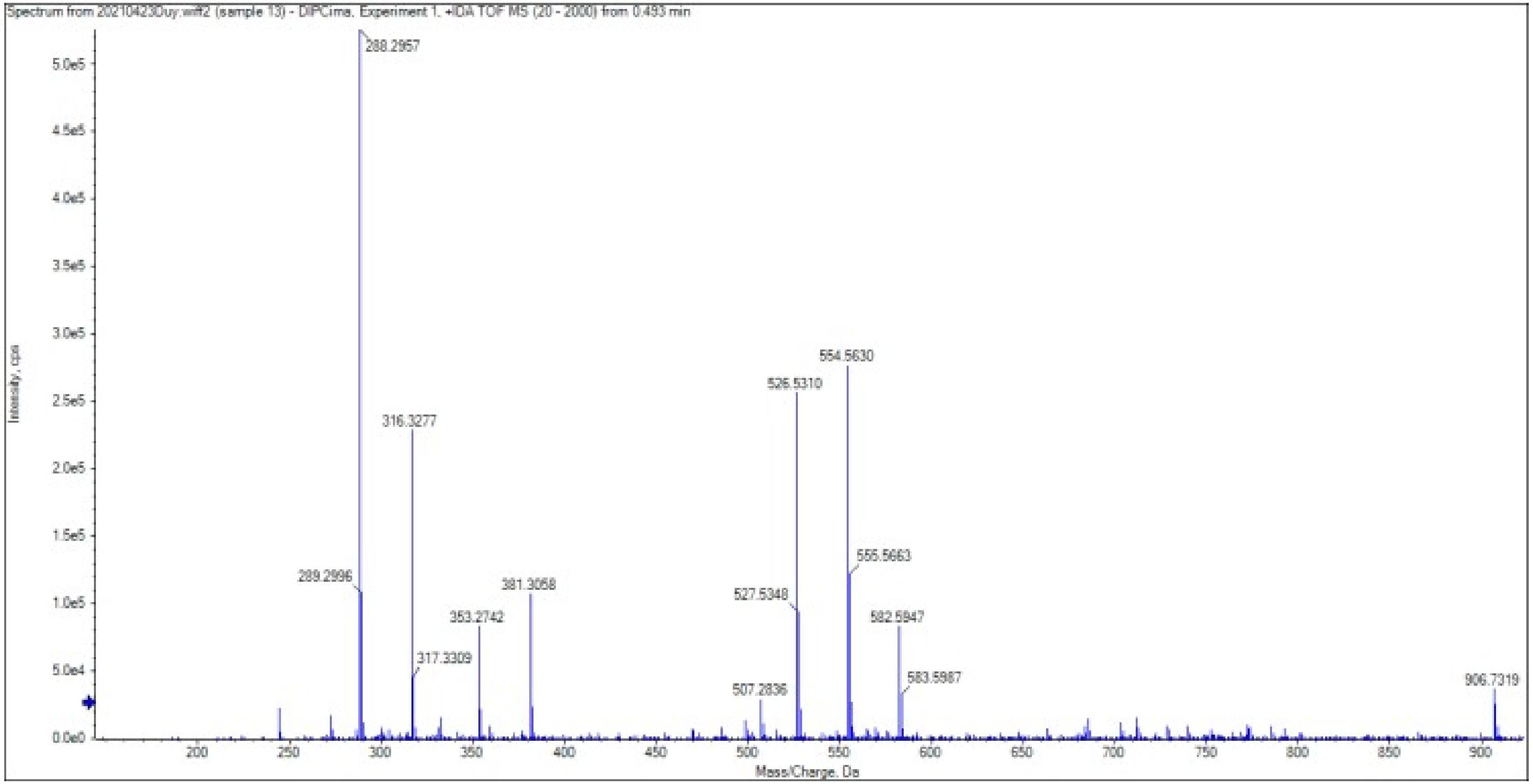


(+)-HR-ESI-MS spectrum of compound **6e**

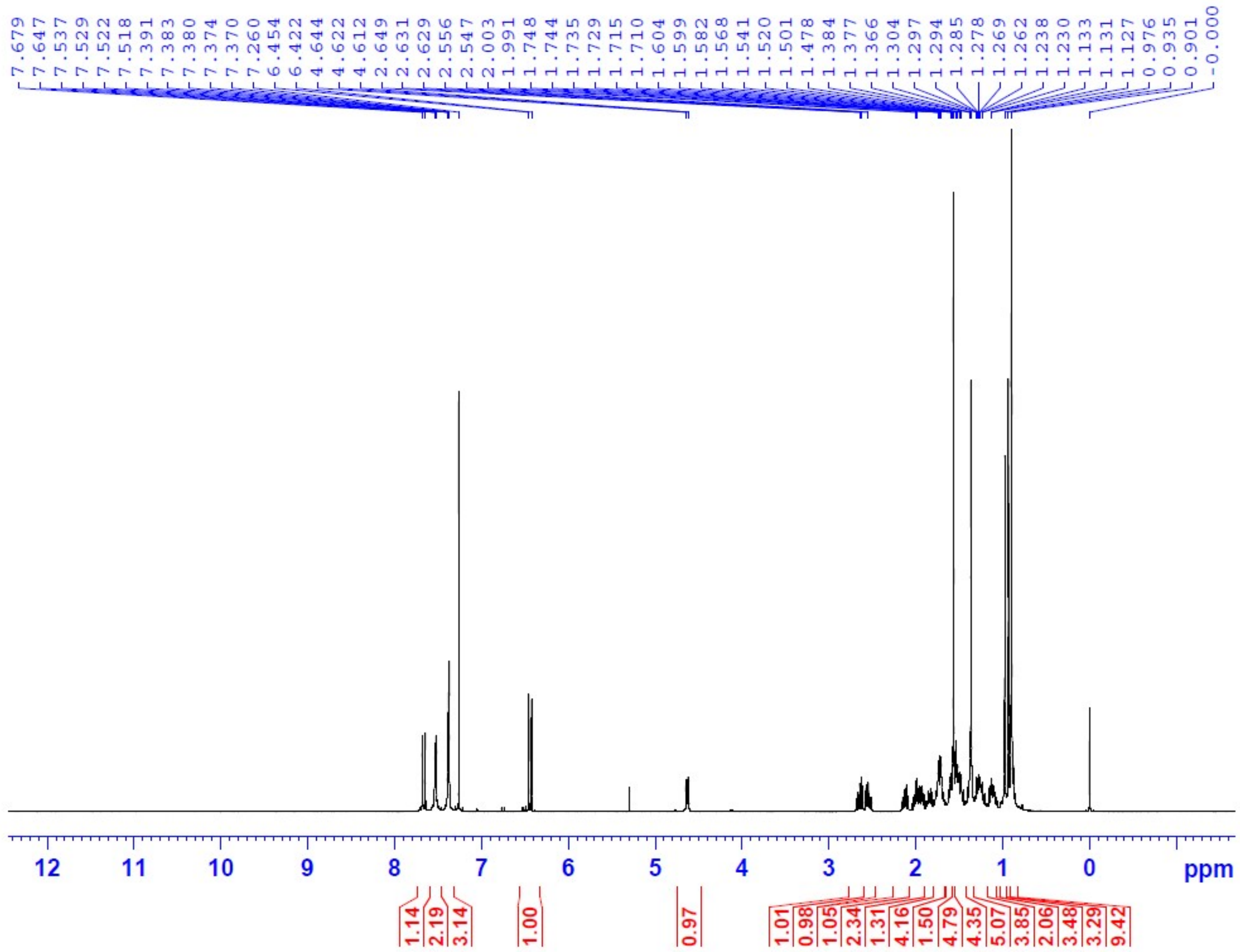

$^{1}$H-NMR spectrum of compound **6e**

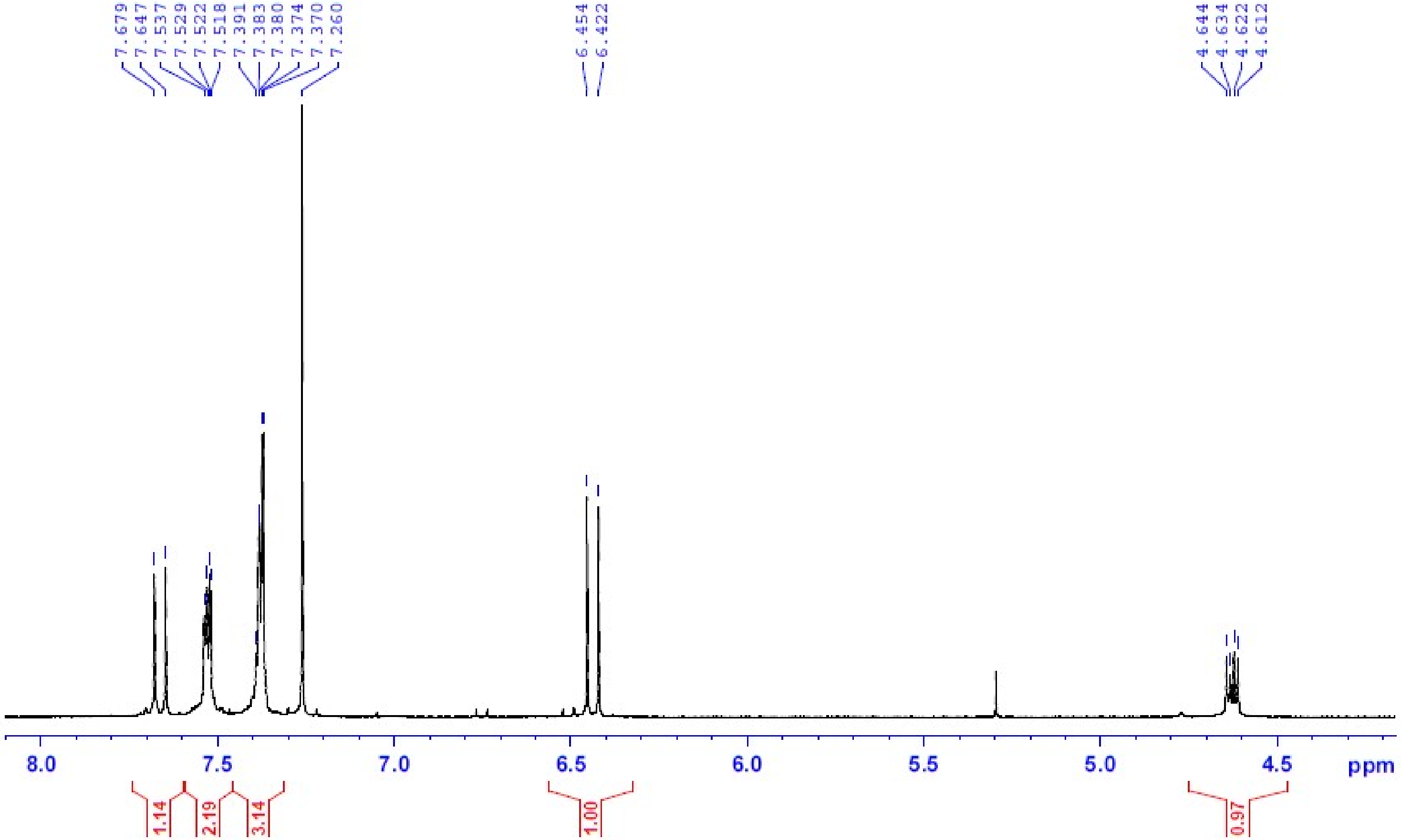
7.679
7.647
7.537
7.529
7.522
7.518
7.391
7.383
7.380
7.374
7.370
7.260
6.454
6.422
4.644
4.634
4.622
4.612
8.0
7.5
7.0
6.5
6.0
5.5
5.0
4.5
ppm
1.14
2.19
3.14
1.00
0.97

$^{1}$H-NMR spectrum of compound **6e** (extension)

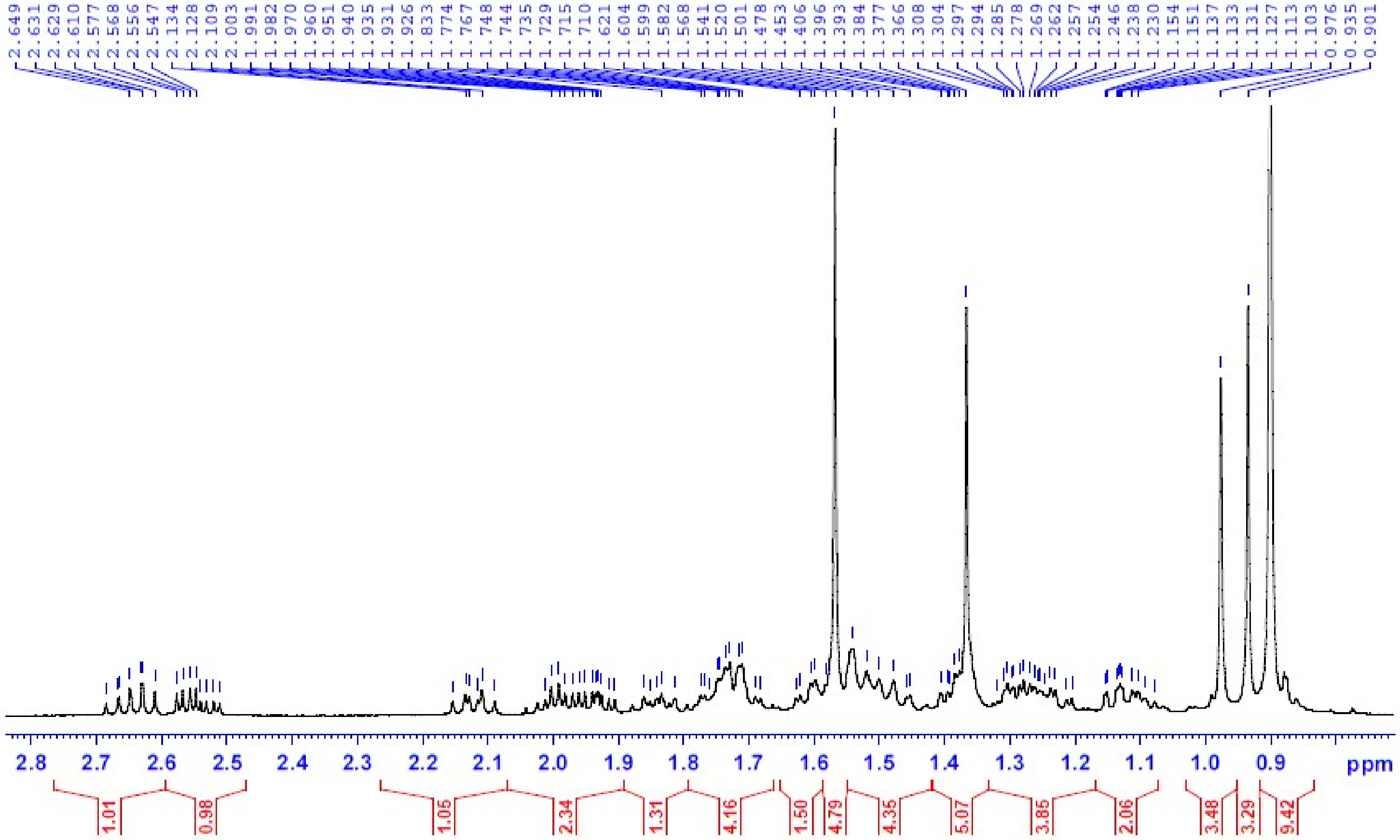

$^{1}$H-NMR spectrum of compound **6e** (extension)

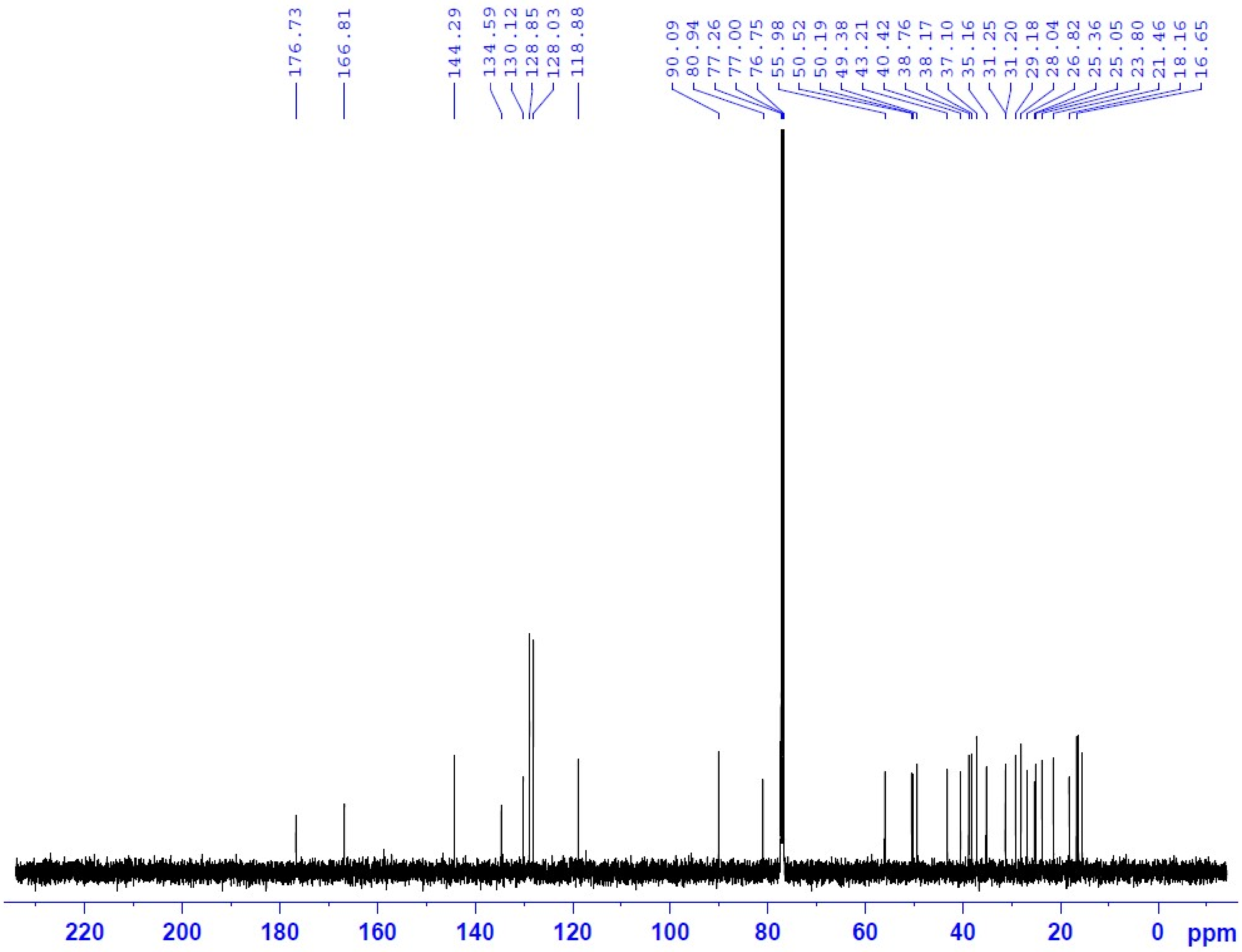


$^{13}$C-NMR spectrum of compound **6e**

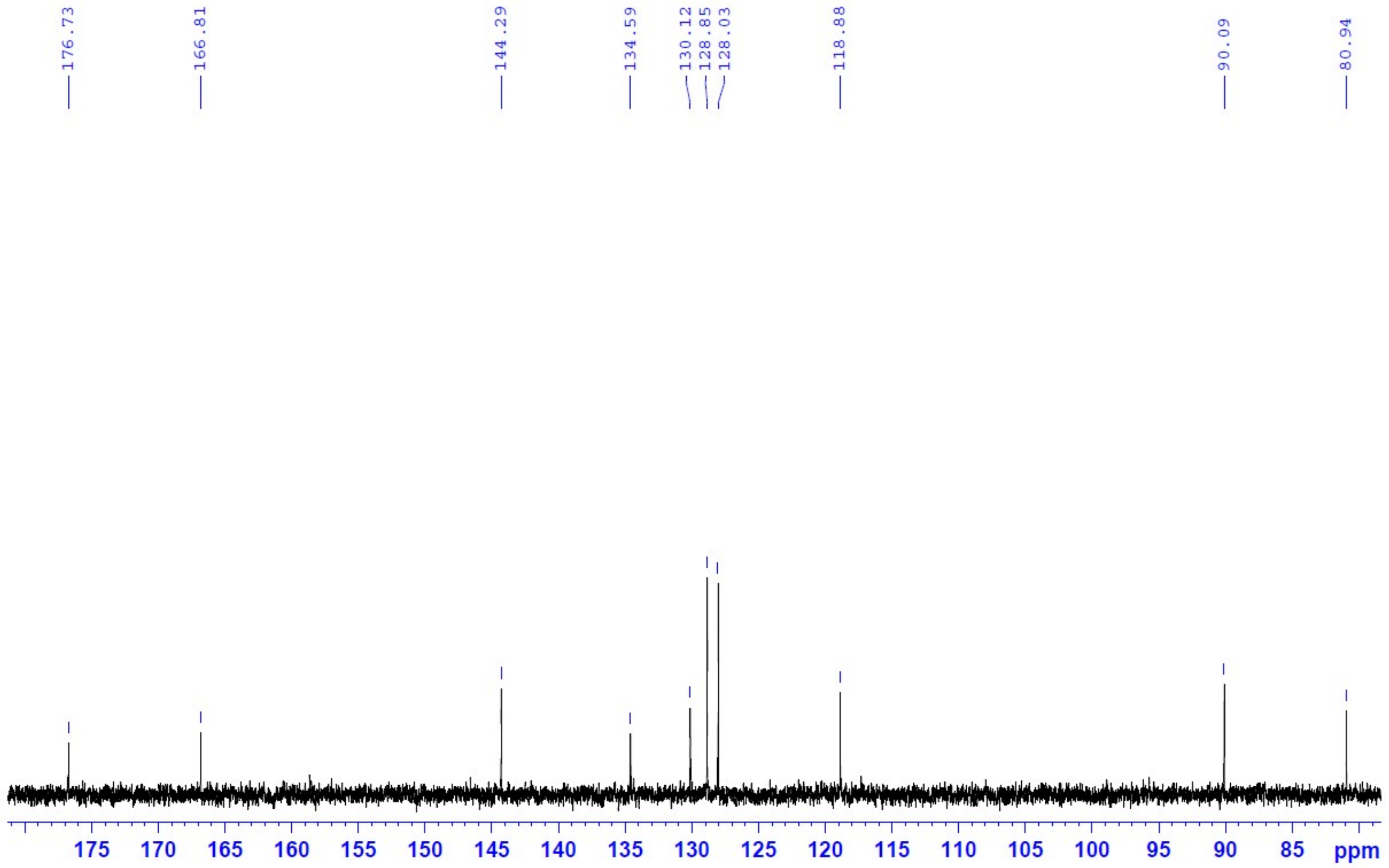

$^{13}$C-NMR spectrum of compound **6e** (extension)

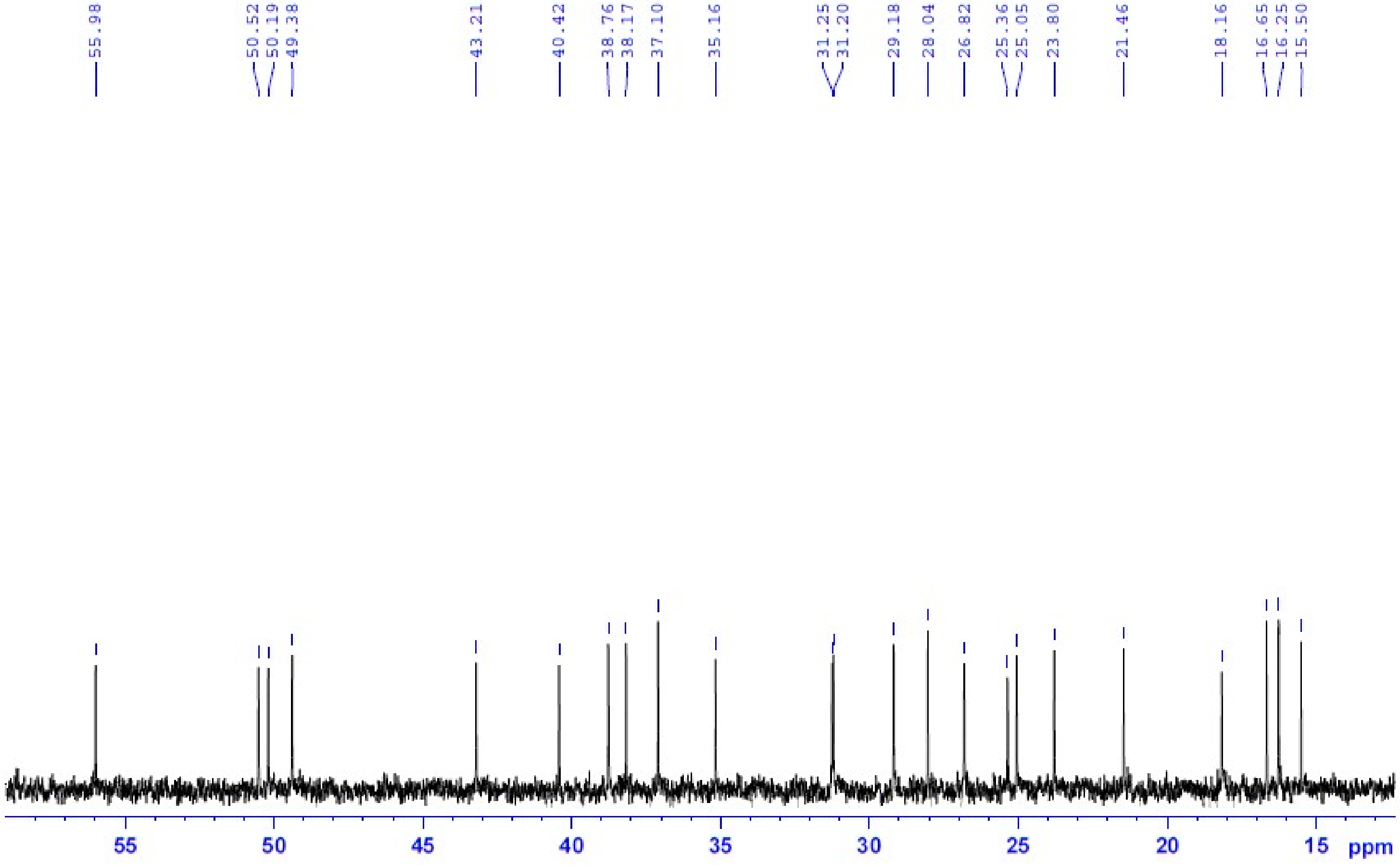
55.98
50.52
50.19
49.38
43.21
40.42
38.76
38.17
37.10
35.16
31.25
31.20
29.18
28.04
26.82
25.36
25.05
23.80
21.46
18.16
16.65
16.25
15.50
55
50
45
40
35
30
25
20
15
ppm

$^{13}$C-NMR spectrum of compound **6e** (extension)

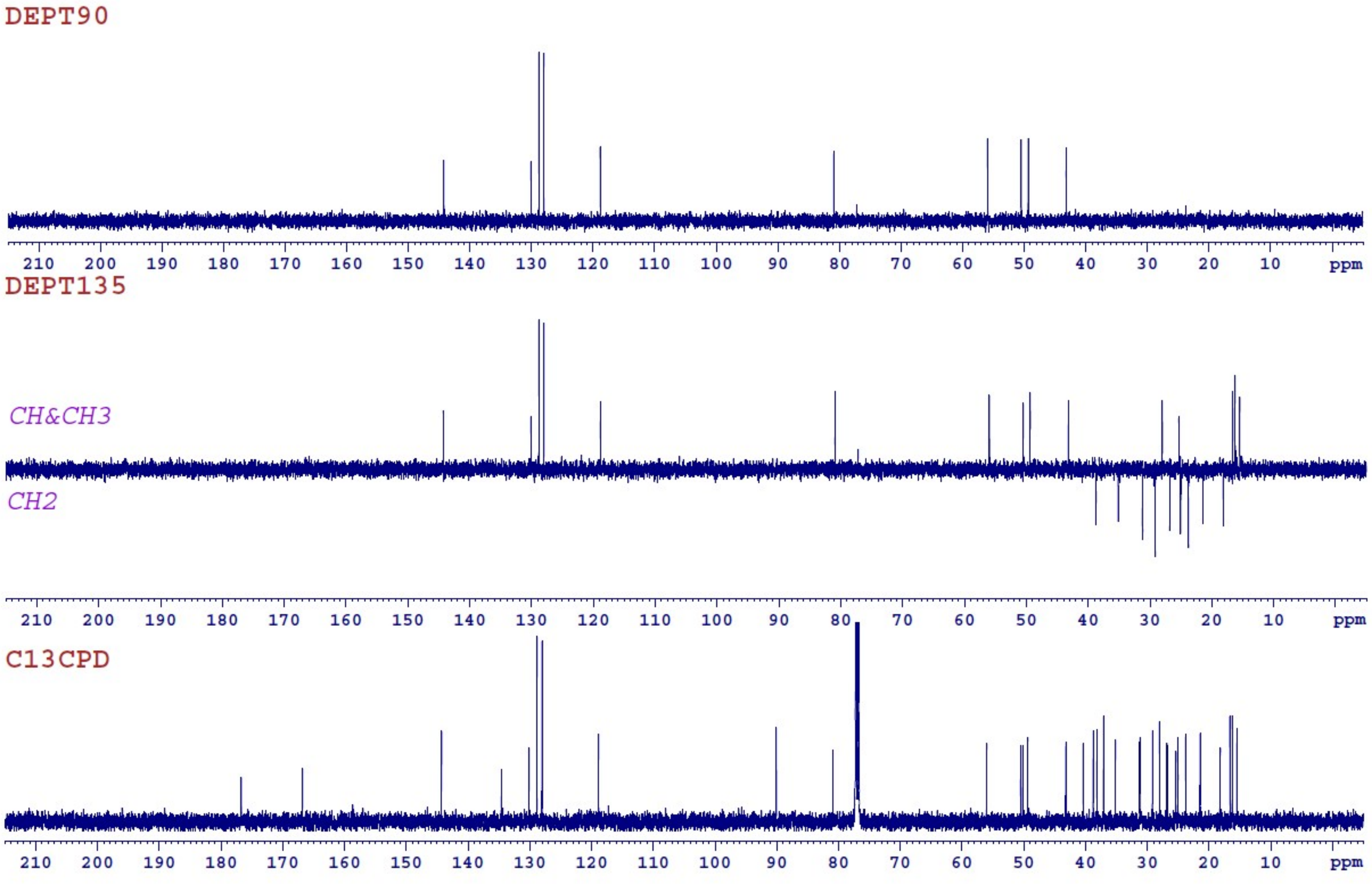


DEPT spectrum of compound **6e**

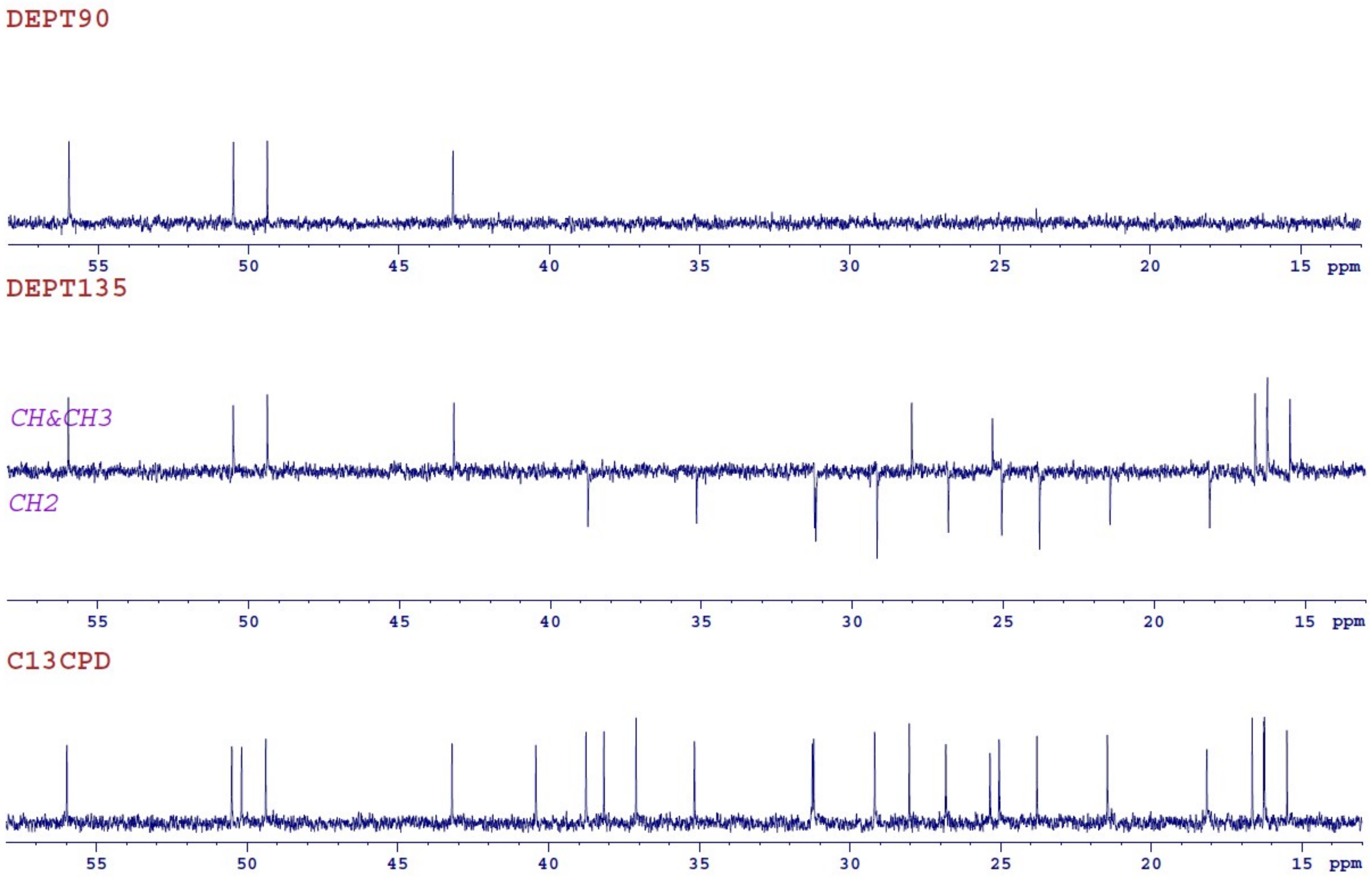


DEPT spectrum of compound **6e** (extension)

## 2. COMPUTATIONAL SIMULATION

### 2.1. In-detail data of ligand-3W37 inhibitory complexes

**Table S1.** Molecular docking simulation results for inhibitory complexes between the compounds and the protein 3W37 with amino acids: **1-3W37**, **2-3W37**, **3a-3W37**, **3b-3W37**, **3c-3W37**, **3d-3W37**, **3e-3W37**, **3f-3W37**, **3g-3W37**, **3h-3W37**, **3i-3W37**, **3k-3W37**, **3l-3W37**, **3m-3W37**, **4-3W37**, **5-3W37**, **6a-3W37**, **6b-3W37**, **6c-3W37**, **6d-3W37**, **6e-3W37**

| Ligand-protein complex | | | Hydrogen bond | | | | | | van de Waals interaction |
|---|---|---|---|---|---|---|---|---|---|
| Name | DS | RMSD | L | P | | T | D | E | |
| **1-3W37** | -10.9 | 1.24 | O | O | Asp666 | H-donor | 2.93 | -0.8 | Arg670, Arg699, Glu792, Ile759. Gly791, Tyr659, Thr790, Val760, Leu663, Asn758, Arg676, Ilr672, Glu302, Met302 |
| | | | O | O | Thr299 | H-acceptor | 2.94 | -1.4 | |
| **2-3W37** | -12.9 | 1.67 | O | O | Thr681 | H-donor | 3.04 | -0.7 | Thr299, Arg670, Arg699, Phe680, Glu301 |
| | | | N | O | Glu792 | H-donor | 2.89 | -7.1 | |
| | | | O | N | Arg814 | H-acceptor | 2.94 | -3.2 | |
| | | | O | N | Arg814 | H-acceptor | 3.22 | -2.2 | |
| **3a-3W37** | -9.7 | 1.89 | C | O | Ile759 | H-donor | 3.00 | -1.0 | Glu301, Ile672, Arg676, Gly791, Thr790, Val760, Pro658, Leu663, Tyr659, Glu792, Thr662, Leu663, Arg699, Asp666, Arg670, Thr681, Phe680 |
| | | | N | N | Ala761 | H-acceptor | 2.94 | -3.5 | |
| **3b-3W37** | -13.4 | 1.78 | N | N | Arg670 | H-acceptor | 3.10 | -0.9 | Glu301, Ile672, Tyr659, Ile759, Leu663, Gly791, Thr662, Arp676, Asp666, Arg699, Asp684, Phe682, Thr681 |
| | | | O | N | Arg670 | H-acceptor | 3.02 | -2.2 | |
| | | | 6-ring | C | Glu792 | π-H | 4.16 | -0.9 | |
| **3c-3W37** | -10.6 | 1.29 | C | O | Asp666 | H-donor | 3.16 | -0.7 | Leu663, Arg670, Gly791, Glu792, Tyr659, Thr662, Thr790, Thr681, Phe680, Arg814, Glu301 |
| | | | O | N | Arg699 | H-acceptor | 2.94 | -1.2 | |
| **3d-3W37** | -12.3 | 1.29 | N | N | Arg670 | H-acceptor | 3.10 | -0.8 | Thr790, Gly791, Thr681, Glu301, Phe680, Pro683, Asp684, Asp666, Leu663, Ile672, Thr662, Tyr659, Glu792 |
| | | | O | N | Arg670 | H-acceptor | 3.13 | -1.2 | |
| | | | O | N | Arg676 | H-acceptor | 3.52 | -0.7 | |
| | | | O | N | Arg699 | ionic | 3.41 | -2.3 | |
| **3e-3W37** | -13.8 | 1.12 | O | S | Met302 | H-donor | 3.86 | -1.1 | Asp305, Glu792, Thr790, Arg670, Asn758, Gly700, Tyr659, Leu663, Asp666, Arg814. Glu301 |
| | | | O | C | Gly791 | H-acceptor | 3.19 | -0.7 | |
| | | | N | C | Arg699 | H-acceptor | 3.65 | -0.6 | |
| | | | O | N | Arg699 | ionic | 3.41 | -2.3 | |

| | | | | | | | | | |
|---|---|---|---|---|---|---|---|---|---|
| | | | N | N | Arg699 | ionic | 3.21 | -3.2 | |
| | | | N | N | Arg699 | ionic | 3.13 | -3.7 | |
| **3f-3W37** | -10.5 | 1.94 | N | N | Arg699 | H-acceptor | 2.91 | -2.4 | Pro683, Arg670, Glu301, Phe680, Asp666, Thr790, Val760, Ile754, Gly791, Ile701, Ile759, Gly700, Gly698, Thr681, Glu792, Arg814, Met302, Thr299 |
| | | | 6-ring | C | Tyr659 | π-H | 3.69 | -0.6 | |
| **3g-3W37** | -9.8 | 2.05 | C | O | Asp666 | H-donor | 3.10 | -1.0 | Pro683, Leu663, Thr790, Glu792, Arg670, Tyr659, Thr662, Gly791, Thr681, Phe680, Glu301, Thr299, Arg298 |
| | | | O | N | Arg699 | H-acceptor | 2.99 | -1.2 | |
| **3h-3W37** | -9.9 | 1.92 | O | O | Asp666 | H-donor | 2.91 | -1.2 | Asp684, Glu301, Arg676, Arg699, Tyr659, Gly698, Leu663, Ile759, Val760, Thr790, Gly791, Glu792, Arg670, Thr299, Pro683 |
| | | | O | N | Arg298 | H-acceptor | 2.98 | -1.5 | |
| **3i-3W37** | -11.3 | 1.81 | N | N | Arg699 | H-acceptor | 3.28 | -1.6 | Met302, Phe680, Thr299, Glu301, Thr681, Gly791, Glu792, Tyr659, Leu663, Asp666, Ile672, Pro683, Arg298 |
| | | | O | N | Arg670 | H-acceptor | 3.25 | -0.7 | |
| | | | O | N | Arg676 | H-acceptor | 3.21 | -1.0 | |
| | | | O | N | Arg676 | H-acceptor | 3.35 | -1.2 | |
| **3k-3W37** | -11.6 | 1.03 | O | O | Thr681 | H-donor | 3.04 | -0.8 | Asp684, Pro683, Phe680, Ile672, Glu301, Arg676, Asp666, Thr790, Arg814, Gly791, Arg670, Glu792 |
| | | | N | N | Arg699 | H-acceptor | 2.86 | -1.0 | |
| | | | O | N | Arg699 | H-acceptor | 3.64 | -0.5 | |
| **3l-3W37** | -10.9 | 1.70 | O | O | Asp666 | H-donor | 2.96 | -1.2 | Arg298, Gly700, Asn758, Val760, Thr790, Tyr659, Arg699, Ile759, Gly791, Arg676, Glu792, Ile672, Glu301, Pro683, Thr299 |
| | | | O | N | Arg670 | H-acceptor | 2.73 | -0.7 | |
| **3m-3W37** | -9.1 | 1.95 | O | N | Arg699 | H-acceptor | 3.19 | -2.3 | Val760, Leu663, Gly698, Tyr659, Glu792, Thr790, Arg670, Arg676, Asp666, Pro683, Thr299, Ile759, Gly791, Asn758 |
| **4-3W37** | -8.5 | 1.87 | O | N | Arg699 | H-acceptor | 3.11 | -1.6 | Thr790, Arg670, Thr299, Met302, Arg814, Glu792, Gly791 |
| **5-3W37** | -14.9 | 1.30 | O | N | Arg670 | H-acceptor | 2.96 | -0.9 | Met302, Thr299, Gly698, Glu792, Asp666, Leu663, Arg699, Ile672, Glu301 |
| | | | O | N | Arg676 | H-acceptor | 2.86 | -4.4 | |
| | | | O | N | Arg676 | H-acceptor | 3.22 | -0.8 | |
| **6a-3W37** | -9.7 | 1.19 | O | N | Arg676 | H-acceptor | 2.83 | -4.6 | Glu792, Gly791, Asp666, Leu663, Ile759, Asn758, Thr662, Val760, Gly700, Gly698, Tyr659, Thr790, Arg670, Arg676. |
| **6b-3W37** | -10.1 | 1.22 | O | N | Arg699 | H-acceptor | 2.99 | -0.8 | Arg670, Arg676, Glu792, Leu663, Asn758, Gly698, Val760, Gly791, Tyr659, Ile701, Ile759, Thr790, Asp666, Arg814 |
| | | | O | N | Gly700 | H-acceptor | 2.82 | -2.8 | |
| **6c-3W37** | -13.2 | 1.12 | O | O | Asn758 | H-donor | 2.60 | -1.2 | Ile759, Glu792, Tyr659, Thr662, Asp666, Leu663, Arg676, Ile672, Arg670, Val760, Gly698, Gly791, Thr790, Gly700 |
| | | | O | N | Arg699 | H-acceptor | 2.96 | -1.6 | |
| **6d-3W37** | -13.7 | 1.31 | C | O | Asp666 | H-donor | 3.33 | -0.8 | Gly698, Ile759, Leu663, Asn758, Gly791, Thr662, Tyr659, Thr790, Arg670 |

| | | | | | | | | | |
|---|---|---|---|---|---|---|---|---|---|
| | | | O | N | Arg676 | H-acceptor | 3.25 | -1.7 | |
| | | | O | N | Arg676 | H-acceptor | 3.35 | -0.7 | |
| | | | 6-ring | C | Glu792 | π-H | 4.34 | -0.7 | |
| **6e-3W37** | -15.2 | 0.31 | O | N | Lý309 | H-acceptor | 3.42 | -1.5 | Arg699, Ile759, Tyr659, Thr662, Gly791, Leu663, Ile672, Asp666, Glu301, Asp305, Met302 |
| | | | O | N | Arg670 | H-acceptor | 3.12 | -2.1 | |
| | | | O | N | Arg676 | H-acceptor | 3.37 | -0.6 | |
| | | | 6-ring | C | Glu792 | π-H | 4.36 | -0.6 | |

**DS**: Docking score energy (kcal.mol$^{-1}$); **RMSD**: Root-mean-square deviation (Å); **L**: Ligand; **P**: Protein; **T**: Type; **D**: Distance (Å); **E**: Energy (kcal.mol$^{-1}$)

## 2.2. In-detail data of ligand-3AJ7 inhibitory complexes

**Table S2.** Molecular docking simulation results for inhibitory complexes between the compounds and the protein 3AJ7 with amino acids: **1-3AJ7**, **2-3AJ7**, **3a-3AJ7**, **3b-3AJ7**, **3c-3AJ7**, **3d-3AJ7**, **3e-3AJ7**, **3f-3AJ7**, **3g-3AJ7**, **3h-3AJ7**, **3i-3AJ7**, **3k-3AJ7**, **3l-3AJ7**, **3m-3AJ7**, **4-3AJ7**, **5-3AJ7**, **6a-3AJ7**, **6b-3AJ7**, **6c-3AJ7**, **6d-3AJ7**, **6e-3AJ7**

| Ligand-protein complex | | | Hydrogen bond | | | | | | van der Waals interaction |
|---|---|---|---|---|---|---|---|---|---|
| Name | DS | RMSD | L | P | | T | D | E | |
| **1-3AJ7** | -10.2 | 1.59 | O | O | Pro312 | H-donor | 2.77 | -2.3 | Phe178, Glu411, Phe159, Gln279, Arg315, Asp242, His280, Leu313, Phe303, Ser311, Phe314, Asp307, Tyr158, Glu277, Asp252 |
| | | | O | N | Arg442 | H-acceptor | 3.29 | -2.0 | |
| **2-3AJ7** | -11.9 | 1.46 | O | O | Asp242 | H-donor | 2.76 | -3.3 | Asp69, Asp307, Glu277, His280, Phe303, Ser240, Phe178, Val216, Tyr158, Ser241, Gln279, Lys156, Pro312, Ser157, Arg315, Phe159, Glu411, Asp242 |
| | | | O | N | Arg442 | H-acceptor | 2.75 | -1.3 | |
| **3a-3AJ7** | -9.5 | 1.77 | O | O | Asp242 | H-donor | 2.63 | -1.2 | His280, Leu246, Tyr158, Asp69, Asp352, Asp215, Tyr72, Arg442, Phe178, Glu411, Phe303, Arg315, Phe159, Gln279, Pro312, Leu313, Asp307, Ph314 |
| | | | C | O | Glu277 | H-donor | 3.05 | -0.9 | |
| | | | O | O | Ser240 | H-acceptor | 2.79 | -1.5 | |
| **3b-3AJ7** | -12.2 | 0.99 | C | O | Glu277 | H-donor | 3.42 | -0.7 | Leu313, Pro312, Gln279, Tyr316, Phe314, His280, Phe303, Glu411, Arg315, Phe178, Tyr158, Asp307, Phe159, Asp215, Asp69, Asp352, Gln353, Arg442, Asp242, Val232 |
| | | | C | O | Ser240 | H-acceptor | 2.68 | -1.5 | |
| | | | C | 6-ring | Tyr72 | H-π | 4.09 | -0.6 | |
| **3c-3AJ7** | -11.8 | 1.45 | O | O | Pro312 | H-donor | 2.64 | -1.9 | Asp242, Tyr158, Ser240, Lys156, Phe303, Ser241, Tyr72, Ser157, His112, Arg442, Glu277, Asp215, Asp352, Gln182, Arg446, Val216, Gln352, Asp69, Asp307, Glu411, Gln279, Arg315, His280, Leu313, Phe314 |
| | | | O | N | His351 | H-acceptor | 3.14 | -1.1 | |
| | | | 6-ring | 6-ring | Phe178 | π-π | 3.96 | -0.0 | |
| **3d-3AJ7** | -10.9 | 1.43 | O | O | Asp352 | H-donor | 3.03 | -0.8 | Tyr72, His112, Asp215, Tyr158, Glu277, Phe303, Phe178, Gln279, Asp307, Val216, Asp242, Pro312, Val232, Leu313, Phe314, Arg315, Glu411, Gln353, Arg442, Asp69 |
| | | | N | O | Ser240 | H-acceptor | 3.07 | -1.1 | |
| | | | O | O | Ser240 | H-acceptor | 2.77 | -1.8 | |
| **3e-3AJ7** | -15.3 | 1.79 | O | O | Asp242 | H-donor | 2.61 | -1.0 | Phe314, Pro312, Ser311, Asp307, Leu313, Phe303, His280, Arg315, Gln279, Phe178, Glu411, Asp215, Phe159, Arg446, Asp69, His351, Asp352. Tyr72, Glu277, Val215, Tyr158, Val 232 |
| | | | O | O | Ser240 | H-acceptor | 2.88 | -0.6 | |
| | | | O | N | Arg442 | ionic | 3.21 | -3.2 | |
| | | | N | N | Arg442 | Ionic | 2.79 | -6.0 | |
| | | | N | N | Arg442 | ionic | 4.00 | -0.5 | |
| **3f-3AJ7** | -12.2 | 1.37 | O | O | Asp242 | H-donor | 2.62 | -1.3 | Leu246, Ser240, Asp69, Tyr72, His112, Phe159, Gln182, Arg442, Phe178, |

| | | | | | | | | | |
|---|---|---|---|---|---|---|---|---|---|
| | | | C | O | Glu277 | H-donor | 3.08 | -1.0 | Asp352, Phe303, Tyr158, Arg315, Glu411, Asp307, Gln279, Ser311, Phe314, Pro312, Leu313, His280. |
| | | | C | O | Asp215 | H-donor | 3.30 | -0.8 | |
| **3g-3AJ7** | -9.9 | 1.14 | O | O | Asp242 | H-donor | 2.81 | -2.6 | Tyr72, His112, Val109, Val216, Tyr158, Phe178, Asp215, Phe303, Phe159, Glu277, Glu411, Gln279, Asp307, His280, Pro312, Arg315, Arg442, Gln182, Gln352, Asp352, Arg446, Asp69 |
| | | | O | N | His351 | H-acceptor | 3.12 | -1.1 | |
| **3h-3AJ7** | -10.4 | 1.43 | I | O | Glu277 | H-donor | 3.48 | -0.2 | Pro312, Ser311, Arg315, Glu411, Asp353, Arg442, Val216, Tyr158, Phe303, Asp242, Gln279, Ser304, Asp307. |
| | | | C | 5-ring | His280 | H-π | 3.55 | -0.9 | |
| **3i-3AJ7** | -11.7 | 1.26 | C | O | Glu277 | H-donor | 3.48 | -0.8 | Asp242, Leu313, Ser241, Lys166, Ser240, Ser157, Leu177, His280, Tyr158, Glu411, Phe178, Phe159, Gln279, Arg442, Tyr72, His112. Asp352, Asp215, Val216, Phe303, Arg315, Phe314, Pro312 |
| | | | C | O | Asp69 | H-donor | 3.58 | -0.6 | |
| **3k-3AJ7** | -12.7 | 1.78 | O | O | Asp242 | H-donor | 2.78 | -3.1 | Ser311, Thr310, Gln279, Pro312, Thr306, Asp307, Phe303, Arg315, Tyr347, Arg213, Glu277, Glu411, His280, Ser240 |
| | | | C | O | Asp352 | H-donor | 3.20 | -0.7 | |
| | | | O | N | Asn350 | H-acceptor | 2.98 | -3.1 | |
| | | | O | N | Gln353 | H-acceptor | 2.91 | -2.2 | |
| | | | C | 6-ring | Tyr158 | H-π | 4.54 | -0.8 | |
| **3l-3AJ7** | -9.6 | 1.83 | C | 6-ring | Tyr158 | H-π | 4.49 | -0.7 | Ser304, Asp307, Gly309, His280, Thr310, val308, Ser241, Asp242, Ser157, Lys156, Ser240, Leu313, Pro312, Phe314 |
| **3m-3AJ7** | -10.3 | 1.79 | C | O | Glu277 | H-donor | 3.15 | -0.8 | Asp242, Pro312, Gln279, Arg315, Asp307, Phe159, Phe178, Thr306, Phe303, Phe301, Arg213, Tyr347, His351, Asp352, Tyr158, Arg442, Glu411, His280, Val232, Ser240, Leu313. |
| | | | O | N | Asn350 | H-acceptor | 2.78 | -2.2 | |
| | | | O | N | Gln353 | H-acceptor | 3.03 | -2.3 | |
| **4-3AJ7** | -10.2 | 1.15 | O | N | Arg442 | H-acceptor | 3.50 | | Gln353, Glu277, Arg315, Phe303, Thr245, Asp242, Ser240, Lys156, Ser157, Asp307, Gln279, Glu411, Asp352, His280 |
| | | | O | N | Ser241 | H-acceptor | 2.87 | | |
| | | | C | 6-ring | Tyr158 | H-π | | | |
| **5-3AJ7** | -15.9 | 1.02 | O | N | Gln353 | H-acceptor | 2.96 | -1.2 | Ser240, Asp352, Thr306, Asp307, Phe303, Glu411, Arg442, Arg315, Pro312, Ser157, tyr316, Lys156, Asp242. |
| | | | O | N | Ser241 | H-acceptor | 3.04 | -0.9 | |
| | | | C | 6-ring | Tyr158 | H-π | 3.96 | -0.7 | |
| | | | C | 6-ring | Tyr158 | H-π | 4.35 | -0.7 | |
| **6a-3AJ7** | -11.6 | 1.67 | O | N | Ser241 | H-acceptor | 2.99 | -2.7 | Val216, Asp352, Arg442, Phe314, glu411, Arg315, Ser240, Gln239, Asp242, Lys156, Asp307, Ser157, Phe159, Phe303, Gln279, His280, Phe178, Glu277, Asp216, Val216 |
| | | | C | 6-ring | Tyr158 | H-π | 4.16 | -0.6 | |
| | | | C | 6-ring | Tyr158 | H-π | 4.22 | -0.6 | |

| | | | | | | | | | |
|---|---|---|---|---|---|---|---|---|---|
| **6b-3AJ7** | -10.6 | 1.22 | C | O | Glu277 | H-donor | 3.39 | -0.7 | Tyr72, Asp352, Gln253, Arg442, Asp242, Pro312, Ser240, Val232, leu313, Asp233, His280, Gln279, Phe303, Glu411, Arg315, Tyr158, Asp215, Phe178, Val216, Arg213, |
| | | | O | N | His351 | H-acceptor | 3.25 | -2.1 | |
| **6c-3AJ7** | -13.7 | 0.97 | O | C | Phe314 | H-acceptor | 3.30 | -0.7 | Gln353, Asp69, Asp352, Gln182, His351, tyr72, His112, Glu277, Gln279, Asp215, Phe178, Phe159, His280, Val216, Glu411, Phe303, Tyr158, Leu313, Pro312, Asp307, Ser311 |
| | | | O | N | Arg315 | H-acceptor | 3.14 | -1.6 | |
| | | | O | N | Arg442 | H-acceptor | 2.91 | -1.9 | |
| | | | | | | | | | |
| **6d-3AJ7** | -12.5 | 0.95 | O | O | Glu277 | H-donor | 2.70 | -6.1 | Asp242, Leu313, Pro312, Asp352, Asp69, Val216, Tyr72, Asp215, Phe178, Tyr158, Glu411, Phe303, His280, Arg315, Gln279, Gln239, Asp233, Val232, Phe159, Trp238. |
| | | | O | N | Lys156 | H-donor | 3.16 | -5.0 | |
| | | | O | N | Ser240 | H-acceptor | 3.16 | -0.8 | |
| | | | O | N | Arg442 | H-acceptor | 3.07 | -1.2 | |
| **6e-3AJ7** | -14.4 | 1.79 | O | N | Ser241 | H-acceptor | 3.33 | -1.0 | Ser240, Lys156, Phe314, Pro312, His280, Leu313, Asp325, Ala329, Ile329, Ile328, Glu332, Asp307, Ser311, Thr310, Ser157, Asp242 |
| | | | C | 6-ring | Tyr158 | H-π | 4.65 | -0.6 | |
| | | | 6-ring | C | Ser304 | π-H | 3.68 | -0.7 | |
| **DS**: Docking score energy (kcal.mol$^{-1}$); **RMSD**: Root-mean-square deviation (Å); **L**: Ligand; **P**: Protein; **T**: Type; **D**: Distance (Å); **E**: Energy (kcal.mol$^{-1}$) | | | | | | | | | |

### 2.3. In-detail data of ligand-PTP1B inhibitory complexes

**Table S3.** Molecular docking simulation results for inhibitory complexes between the compounds and the protein PTP1B with amino acids: **1-PTP1B**, **2-PTP1B**, **3a-PTP1B**, **3b-PTP1B**, **3c-PTP1B**, **3d-PTP1B**, **3e-PTP1B**, **3f-PTP1B**, **3g-PTP1B**, **3h-PTP1B**, **3i-PTP1B**, **3k-PTP1B**, **3l-PTP1B**, **3m-PTP1B**, **4-PTP1B**, **5-PTP1B**, **6a-PTP1B**, **6b-PTP1B**, **6c-PTP1B**, **6d-PTP1B**, **6e-PTP1B**

| Ligand-protein complex | | | Hydrogen bond | | | | | | van der Waals interaction |
|---|---|---|---|---|---|---|---|---|---|
| **Name** | **DS** | **RMSD** | **L** | **P** | | **T** | **D** | **E** | |
| **1-PTP1B** | -10.5 | 1.19 | O | N | Gly259 | H-acceptor | 2.87 | -1.8 | Arg47, Asp29, Ser28, Met258, Gly259, Ala27, Arg254, Ser50, Arg24, Lys36 |
| **2-PTP1B** | -13.8 | 1.17 | O | N | Arg24 | H-acceptor | 3.41 | -0.7 | Lys36, Asp29, Arg254, Gly259, Met258, Asp48, Cys32 |
| | | | O | N | Arg24 | H-acceptor | 2.98 | -2.5 | |
| | | | O | N | Gln262 | H-acceptor | 3.24 | -1.6 | |
| **3a-PTP1B** | -9.3 | 1.25 | C | O | Phe30 | H-acceptor | 3.50 | -0.7 | Tyr46, Asp48, Arg24, Met258, Asp29, Pro31, Phe52, Lys36, Asp181, Lys120, Phe182 |
| | | | N | N | Cys32 | H-acceptor | 3.22 | -0.8 | |
| **3b-PTP1B** | -11.5 | 1.98 | O | O | Gln262 | H-donor | 3.16 | -0.7 | Tyr46, Asp48, Val49, Cys32, Phe30, Asp29, Arg24. Phe182, Ala217, Ile219 |
| | | | 6-ring | C | Pro31 | π-H | 4.74 | -0.6 | |
| **3c-PTP1B** | -9.6 | 1.92 | N | N | Arg24 | H-acceptor | 3.11 | -2.0 | Asp29, Met258, Tyr20, Gly259, Phe182, Arg254 |
| | | | N | N | Gln262 | H-acceptor | 3.03 | -1.4 | |
| **3d-PTP1B** | -13.7 | 1.89 | O | N | Arg24 | H-acceptor | 3.33 | -0.9 | Lys120, Tyr46. Val49, Asp48, Gly259, Lys116, Ser118 |
| | | | O | N | Arg24 | H-acceptor | 2.88 | -1.7 | |
| | | | O | N | Gln262 | H-acceptor | 2.87 | -1.5 | |
| | | | O | N | Arg24 | H-acceptor | 3.31 | -0.7 | |
| | | | O | N | Arg254 | H-acceptor | 3.05 | -7.2 | |
| | | | O | N | Arg24 | ionic | 3.31 | -1.3 | |
| | | | O | N | Arg254 | ionic | 3.05 | -2.5 | |
| | | | 6-ring | C | Met258 | π-H | 4.74 | -0.6 | |
| **3e-PTP1B** | -12.9 | 1.43 | O | N | Arg24 | H-acceptor | 3.29 | -1.8 | Met258, Gln262, Ile219, Gly250, Cys32, Asp29, Asp48, Val49 |
| | | | O | N | Lys36 | H-acceptor | 3.12 | -2.8 | |
| | | | O | N | Lys36 | ionic | 3.12 | -2.1 | |
| | | | 6-ring | C | Lys36 | π-H | 3.55 | -0.7 | |

| | | | | | | | | | |
|---|---|---|---|---|---|---|---|---|---|
| **3f-PTP1B** | -10.1 | 1.75 | O | N | Cys32 | H-acceptor | 3.14 | -1.8 | Pro31, Arg33, Lys36, Met258, Asp48, Val49, Ile219, Asp29 |
| | | | O | N | Arg24 | H-acceptor | 3.11 | -2.4 | |
| | | | O | N | Gln262 | H-acceptor | 3.57 | -0.6 | |
| **3g-PTP1B** | -9.0 | 1.90 | O | N | Lys36 | H-acceptor | 3.23 | -3.9 | Cys32, Asp29, Asp48, Ile219, Val49, Phe182, Met258, Gly259 |
| | | | O | N | Arg24 | H-acceptor | 3.32 | -0.9 | |
| | | | O | N | Gln262 | H-acceptor | 3.20 | -2.0 | |
| **3h-PTP1B** | -9.4 | 1.36 | I | S | Met258 | H-donor | 3.66 | -0.7 | Val49. Ile219, Asp48, Lys36, Asp29 |
| | | | O | N | Arg24 | H-acceptor | 2.91 | -2.8 | |
| | | | O | N | Gln262 | H-acceptor | 3.07 | -2,9 | |
| **3i-PTP1B** | -10.3 | 1.14 | N | N | Gln262 | H-acceptor | 3.41 | -0.9 | Phe30, Arg33, Pro31, Asp29, Lys36, Met258, Tyr20, Asp48, Cys32 |
| | | | O | N | Arg24 | H-acceptor | 2.87 | -2.0 | |
| | | | O | N | Gln262 | H-acceptor | 2.85 | -0.7 | |
| **3k-PTP1B** | -9.7 | 1.35 | O | O | Arg24 | H-donor | 3.26 | -0.8 | Asp29, Ser28, Arg254, His25, Ala27, Gln21, Met258, |
| | | | O | N | Lys36 | H-acceptor | 3.06 | -3.8 | |
| **3l-PTP1B** | -9.9 | 1.60 | O | N | Lys36 | H-acceptor | 3.36 | -0.9 | Val49, Asp48, Gly259, Met258, Arg33, Cys32, Asp29, Ser28, Arg24, Ille219, Gln262 |
| | | | O | N | Arg254 | H-acceptor | 3.37 | -1.2 | |
| **3m-PTP1B** | -9.3 | 1.34 | C | 6-ring | Tyr46 | H-π | 4.42 | -0.6 | Met258. Asp48, Gly259, Gln262, Val49, Phe182, Arg24, Ser28, Asp29 |
| **4-PTP1B** | -11.2 | 1.23 | C | S | Met258 | H-donor | 3.89 | -0.8 | Phe30, Lys36, Asp29, Gly259, Asp48, Pro31 |
| | | | O | N | Cys32 | H-acceptor | 3.04 | -3.3 | |
| | | | O | N | Arg24 | H-acceptor | 3.13 | -1.8 | |
| | | | O | N | Gln262 | H-acceptor | 3.08 | -1.5 | |
| **5-PTP1B** | -13.4 | 1.37 | O | N | Arg24 | H-acceptor | 2.87 | -1.9 | Asp29, Ser28, Gly259, ZGln262, Tyr20. Met258, Asp48 |
| | | | O | N | Arg254 | H-acceptor | 3.27 | -1.1 | |
| | | | O | N | Arg254 | H-acceptor | 3.05 | -3.1 | |
| | | | O | N | Lys36 | H-acceptor | 3.25 | -1.0 | |
| **6a-PTP1B** | -9.8 | 1.40 | O | N | Arg254 | H-acceptor | 2.85 | -2.0 | |
| | | | O | N | Cys32 | H-acceptor | 3.13 | -1.0 | Pro31, Arg33, Phe30, Lys36, Asp29, Met258, Gly259, Arg24 |
| **6b-PTP1B** | -11.5 | 1.85 | C | S | Met258 | H-donor | 4.16 | -0.7 | Pro31, cys32, Lys36, Gly259, Arg254, tyr20, Asp48, Asp29, Phe30 |
| | | | O | N | Arg24 | H-acceptor | 3.38 | -0.9 | |
| | | | O | N | Arg24 | H-acceptor | 3.18 | -0.9 | |

| | | | | | | | | | |
|---|---|---|---|---|---|---|---|---|---|
| | | | O | N | Gln262 | H-acceptor | 3.13 | -1.0 | |
| **6c-PTP1B** | -15.8 | 1.99 | O | N | His25 | H-acceptor | 3.03 | -2.0 | Asp46, Ile219, Val49, Met258, Gly259, Asp29, Ala27, Arg254, Ser28 |
| | | | O | N | Arg24 | H-acceptor | 2.96 | -3.7 | |
| | | | O | N | Gln62 | H-acceptor | 3.49 | -0.7 | |
| | | | C | 5-ring | His25 | H-π | 4.30 | -0.7 | |
| **6d-PTP1B** | -10.7 | 1.70 | O | O | Gln262 | H-donor | 3.08 | -0.9 | Asp29, Met258, Asp48, Phe182, Tyr20 |
| | | | O | N | Arg24 | H-acceptor | 2.87 | -1.8 | |
| | | | O | N | Gln262 | H-acceptor | 3.07 | -1.6 | |
| **6e-PTP1B** | -12.2 | 1.54 | C | O | Ser80 | H-donor | 3.29 | -0.8 | Val211, Gly209, Pro206, His208, Pro206, Leu204, Glu75, Lys73, Arg79, Ser203, Ser205 |
| | | | O | N | Gln78 | H-acceptor | 2.83 | -0.8 | |
| | | | 6-ring | C | Glu76 | π-H | 3.85 | -1.2 | |

**DS**: Docking score energy ($kcal.mol^{-1}$); **RMSD**: Root-mean-square deviation (Å); **L**: Ligand; **P**: Protein; **T**: Type; **D**: Distance (Å); **E**: Energy ($kcal.mol^{-1}$)

### 3. DOSE RESPONSE CURVE OF THE MOST POTENT COMPOUNDS

#### 3.1. Dose response curve of compound 5

| | | | | | | | | |
|---|---|---|---|---|---|---|---|---|
| Compound 5 | Concentration (µg/ml) | 128 | 32 | 8 | 2 | 0.5 | 0.125 | 0.03 |
| | Inhibitory rate (0-1) | 1 | 0.99 | 0.98 | 0.62 | 0.41 | 0.15 | 0.07 |

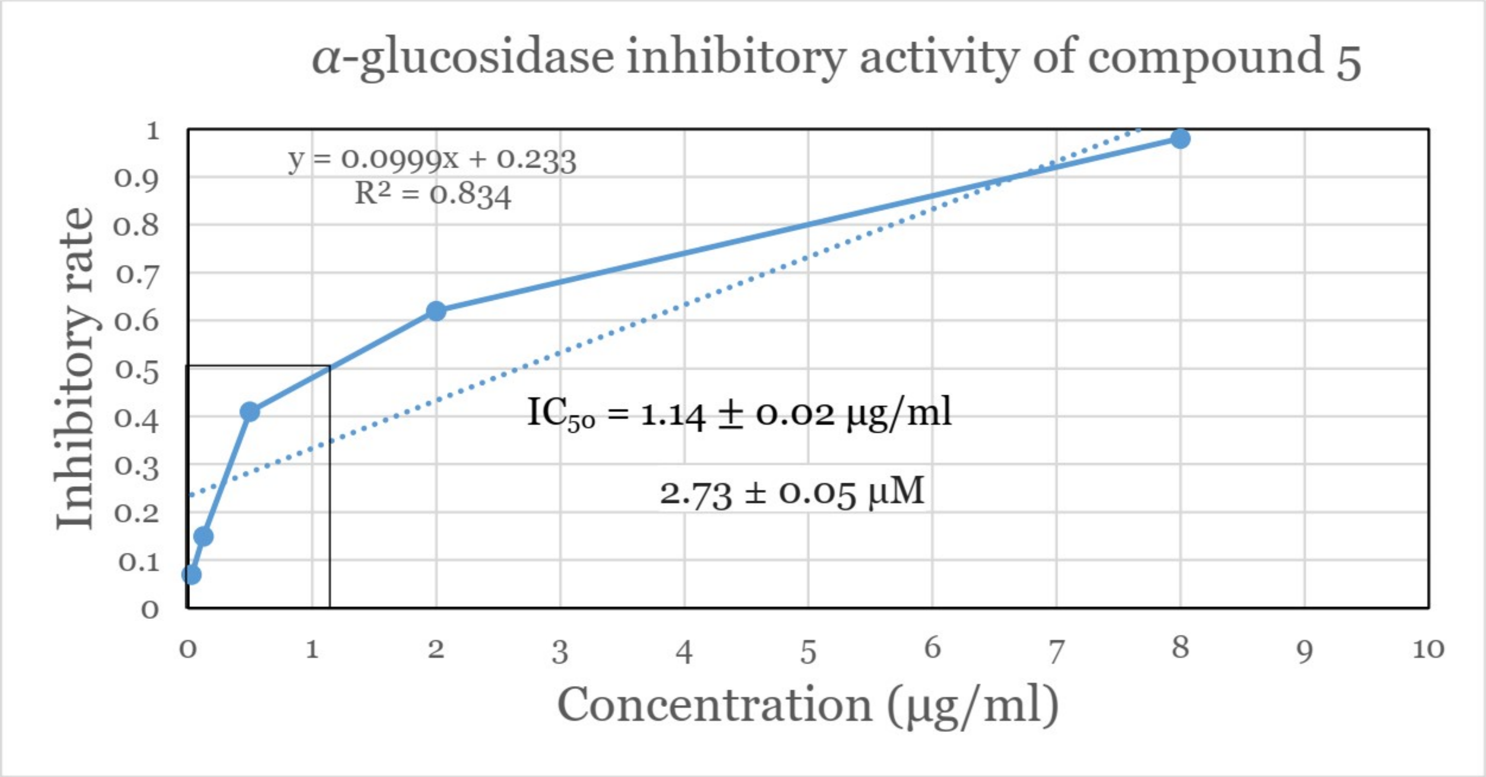


Dose response curve of compound **5**.

### 3.2. Dose response curve of compound 6c

| | | | | | | |
|---|---|---|---|---|---|---|
| Compound 6c | Concentration (μg/ml) | 256 | 64 | 16 | 4 | 1 |
| | Inhibitory rate (0-1) | 0.77 | 0.75 | 0.71 | 0.71 | 0.35 |

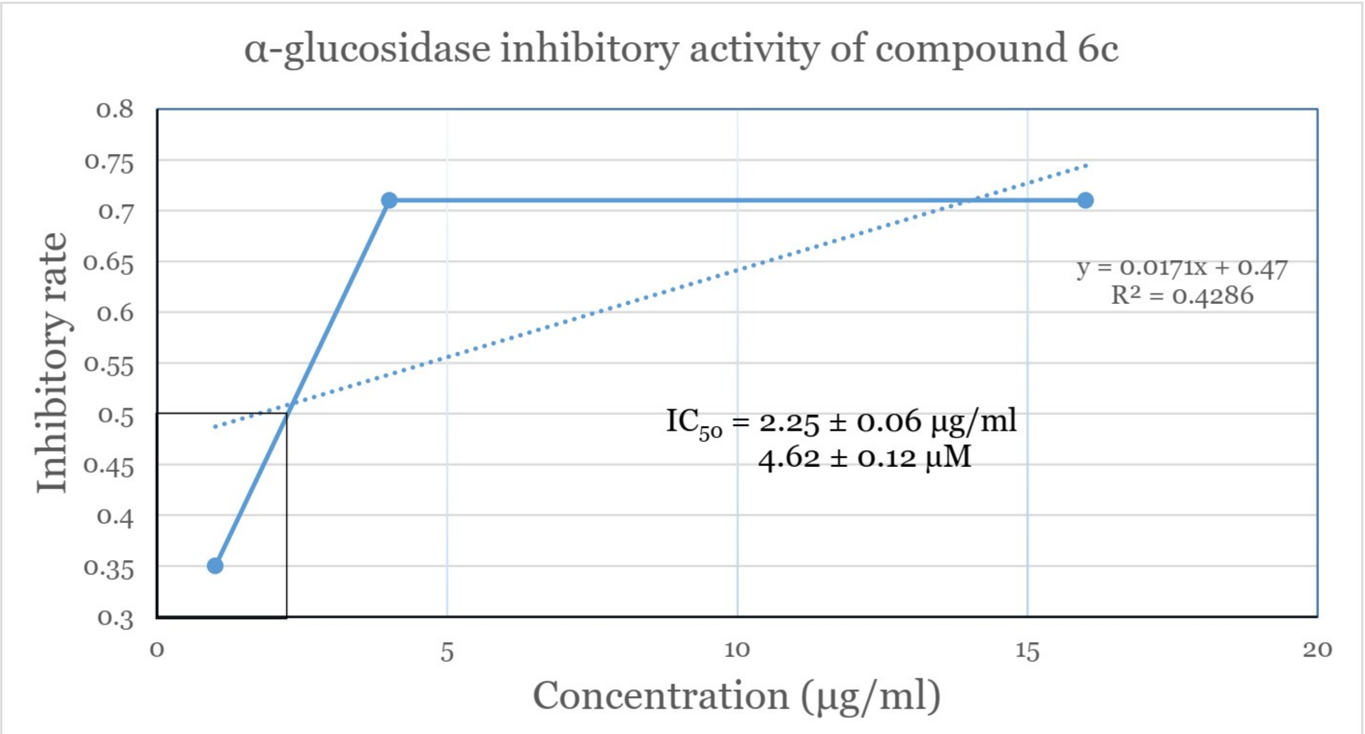


Dose response curve of compound **6c**.

### 3.3. Dose response curve of compound 6e

| | | | | | |
|---|---|---|---|---|---|
| Compound 6e | Concentration (µg/ml) | 256 | 64 | 16 | 4 |
| | Inhibitory rate (0-1) | 0.93 | 0.72 | 0.62 | 0.5 |

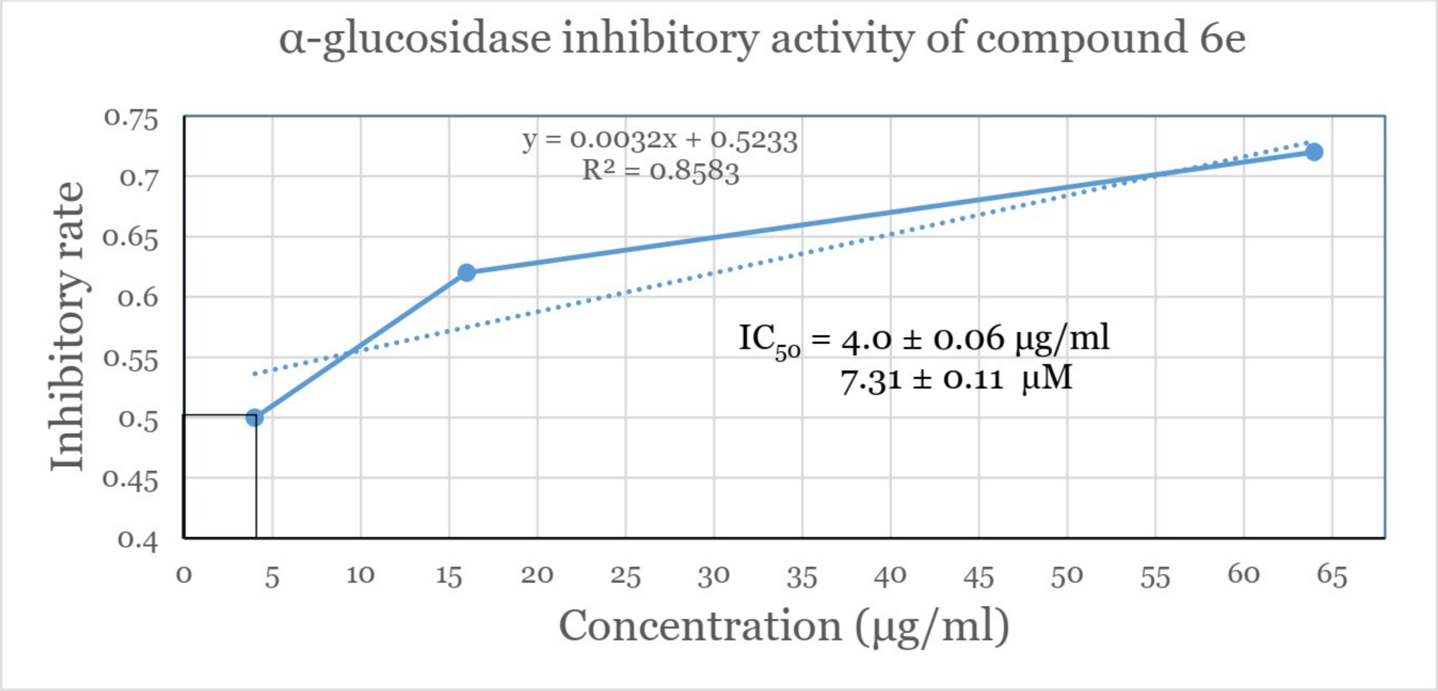


Dose response curve of compound **6e**.
